# Engineered enzymes for enantioselective nucleophilic aromatic substitutions

**DOI:** 10.1038/s41586-025-08611-0

**Published:** 2025-01-15

**Authors:** Thomas M. Lister, George W. Roberts, Euan J. Hossack, Fei Zhao, Ashleigh J. Burke, Linus O. Johannissen, Florence J. Hardy, Alexander A. V. Millman, David Leys, Igor Larrosa, Anthony P. Green

**Affiliations:** 1 Manchester Institute of Biotechnology, The University of Manchester, Manchester, UK; 2 Department of Chemistry, The University of Manchester, Manchester, UK

**Keywords:** Biocatalysis, Biocatalysis, Enzymes

## Abstract

Nucleophilic aromatic substitutions (S_N_Ar) are among the most widely used processes in the pharmaceutical and agrochemical industries^1–4^, allowing convergent assembly of complex molecules through C–C and C–X (X = O, N, S) bond formation. S_N_Ar reactions are typically carried out using forcing conditions, involving polar aprotic solvents, stoichiometric bases and elevated temperatures, which do not allow for control over reaction selectivity. Despite the importance of S_N_Ar chemistry, there are only a handful of selective catalytic methods reported that rely on small organic hydrogen-bonding or phase-transfer catalysts^5–11^. Here we establish a biocatalytic approach to stereoselective S_N_Ar chemistry by uncovering promiscuous S_N_Ar activity in a designed enzyme featuring an activated arginine^12^. This activity was optimized over successive rounds of directed evolution to afford an engineered biocatalyst, S_N_Ar1.3, that is 160-fold more efficient than the parent and promotes the coupling of electron-deficient arenes with carbon nucleophiles with near-perfect stereocontrol (>99% enantiomeric excess (e.e.)). S_N_Ar1.3 can operate at a rate of 0.15 s^−1^, perform more than 4,000 turnovers and can accept a broad range of electrophilic and nucleophilic coupling partners, including those that allow construction of challenging 1,1-diaryl quaternary stereocentres. Biochemical, structural and computational studies provide insights into the catalytic mechanism of S_N_Ar1.3, including the emergence of a halide binding pocket shaped by key catalytic residues Arg124 and Asp125. This study brings a landmark synthetic reaction into the realm of biocatalysis to provide an efficient and versatile platform for catalytic S_N_Ar chemistry.

## Abstract (web summary)

A biocatalytic approach to enantioselective nucleophilic aromatic substitution (S_N_Ar) was achieved through laboratory evolution of highly efficient and selective S_N_Arase biocatalysts.

## Main

**Fig. 1:**
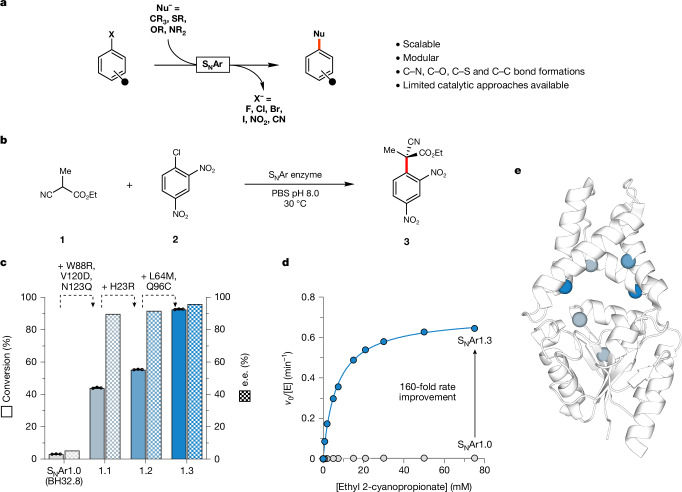
S_N_Ar reactions and directed evolution of an enantioselective S_N_Ar enzyme. **a**, S_N_Ar reactions involve the coupling of aromatic electrophiles with diverse nucleophilic coupling partners. **b**, Chemical scheme showing the target S_N_Ar reaction between ethyl 2-cyanopropionate (**1**) and 2,4-dinitrochlorobenzene (**2**), generating product **3** containing an all-carbon quaternary carbon centre. The S_N_Ar enzymes developed preferentially produce the (*R*)-enantiomer of **3** (Supplementary Information). **c**, Bar chart showing reaction conversion (solid bars) and selectivity (patterned bars) achieved by S_N_Arase variants along the evolutionary trajectory. Reaction conditions: **1** (25 mM), **2** (2.5 mM), S_N_Ar variant (75 μM) in PBS pH 8.0 with 10% v/v DMSO as a co-solvent, 16 h at 30 °C. **d**, Michaelis–Menten kinetic analysis of S_N_Ar1.0 to S_N_Ar1.3 show a 160-fold improvement in *k*_obs_ following evolution (0.0040 ± 0.0002 min^−1^ and 0.65 ± 0.01 min^−1^ for S_N_Ar1.0 and S_N_Ar1.3, respectively; Extended Data Fig. 2). Assays were performed at a fixed concentration of **2** (2.5 mM) and varying concentrations of **1** (3.5–75.0 mM). **e**, Structure showing the six amino acid positions mutated in S_N_Ar1.3 mapped onto the structure of BH32.7 (PDB: 7O1D). Mutations are represented as spheres at the *C*_α_ and coloured according to their order of introduction, corresponding to the variants in Fig. 2b. Data points shown are averages of triplicate measurements, with error bars representing standard deviation. See Supplementary Data for source data.

Nucleophilic aromatic substitutions (S_N_Ar) are fundamental transformations in organic chemistry used to functionalize (hetero)aromatic rings during the synthesis of valuable molecules, including pharmaceuticals and agrochemicals^1,2^. These transformations involve the coupling of electron-deficient (hetero)aryl halide electrophiles with carbon, oxygen, nitrogen or sulfur nucleophiles^3,4,13^ (Fig. 1a). The modularity and operational simplicity of S_N_Ar reactions has led to their widespread use in the synthesis of valuable organic molecules from discovery to manufacturing scales^14^. However, despite their prevalence, these processes still suffer from important limitations that can be attributed to a lack of efficient and general catalysts for mediating S_N_Ar chemistry^15–19^. As a result, established methods of performing S_N_Ar chemistry are incompatible with stereoselective and/or regioselective processes that are highly desirable when constructing complex molecules. To address these limitations, a small number of enantioselective S_N_Ar reactions have recently been developed that make use of small organic hydrogen-bonding or phase-transfer catalysts^5–11^. Although impressive, the efficiency of these organocatalysts is limited and they cannot be easily adapted to operate on new classes of substrates.

We therefore considered alternative catalytic strategies for mediating selective S_N_Ar chemistry that could offer enhanced efficiency and greater flexibility. To this end, our thoughts turned to biocatalysis given the impressive rate accelerations, exacting selectivities and high degree of engineerability associated with enzymes^20–22^. Unfortunately, there are no natural enzymes known that mediate selective and convergent S_N_Ar chemistry. Although the hydrolytic enzymes 4-chlorobenzoyl-CoA dehalogenase^23,24^, 5-nitroanthranilic acid aminohydrolase^25^ and atrazine chlorohydrolase^26^ are thought to operate through S_N_Ar-type pathways, their mechanisms involve metal–hydroxide intermediates or the hydrolysis of covalent aryl esters, meaning that these enzymes cannot be readily adapted to use nucleophiles other than water. Similarly, the promiscuous glutathione arylation activity observed with selected glutathione *S*-transferases probably arises from activation of the glutathione nucleophile^27^ and is not readily adaptable to more valuable substrate classes. In the absence of suitable natural enzymes, here we adopt a ‘bottom-up’ approach to engineer efficient and enantioselective S_N_Ar biocatalysts.

## Engineering an enantioselective S_N_Ar enzyme

To identify a suitable starting template for engineering S_N_Ar enzymes, we considered a family of Morita–Baylis–Hillman (MBH) enzymes recently engineered in our lab that harbour active-site features that could be repurposed to promote the target chemistry^12,28^. These enzymes contain a flexible Arg124 residue as a hydrogen-bond donor that sits adjacent to a binding site for electron-deficient aromatic substrates. Given that hydrogen-bonding catalysts have previously been shown to accelerate S_N_Ar reactions^7^, we evaluated a selection of our in-house MBH enzymes for promiscuous S_N_Ar activity, using a small panel of activated aryl halides and carbon nucleophiles as coupling partners. From this screening, we identified the variant BH32.8 (subsequently referred to as S_N_Ar1.0), which promotes the coupling of ethyl 2-cyanopropionate (**1**) and 2,4-dinitrochlorobenzene (**2**) with modest conversion and stereocontrol (approximately 5% e.e.) (Fig. 1b and Supplementary Fig. 1), as a promising candidate for S_N_Arase engineering. This reaction leads to the generation of product **3** containing an acyclic quaternary carbon stereocentre, a common functional motif in complex organic molecules that is challenging to construct in a stereocontrolled fashion^29^. Furthermore, α-cyano esters serve as precursors to useful chiral motifs, including β-amino acids^30^, β-lactams^31^ and oxindoles^5^.

**Extended Data Fig. 1:**
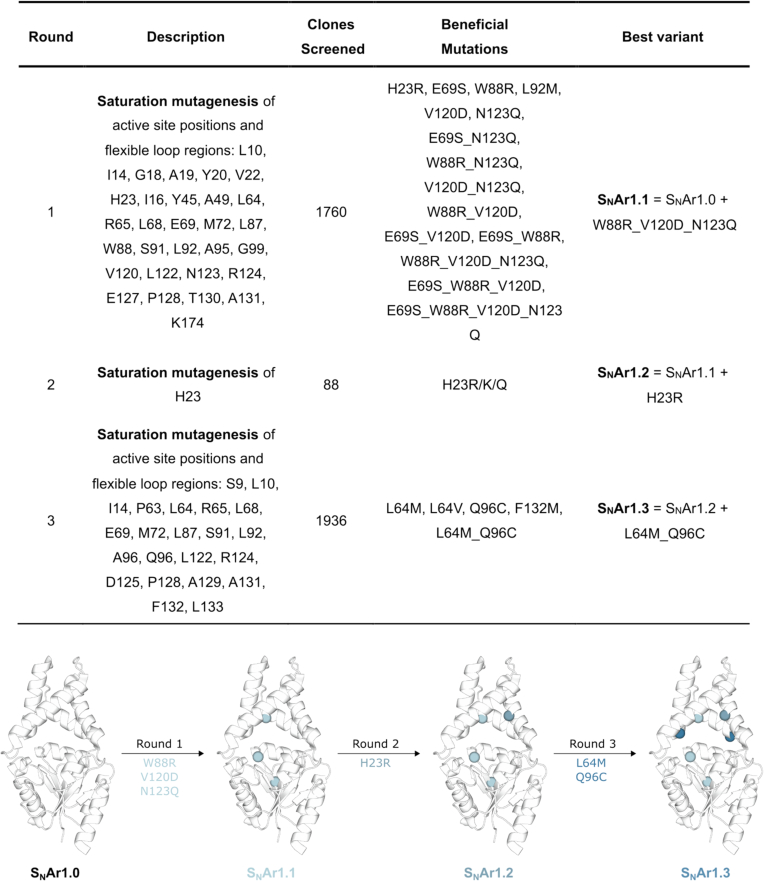
Directed evolution of an efficient and enantioselective S_N_Ar enzyme. Schematic showing the trajectory from S_N_Ar1.0 to S_N_Ar1.3. Mutations introduced are represented as CPK spheres at the *C*_β_. Three rounds of evolution afforded S_N_Ar1.3, which contains six mutations. Library generation method, positions targeted, the number of clones evaluated, beneficial mutations and the most improved variant for each round are given in the table.

To improve activity and selectivity, S_N_Ar1.0 was subjected to successive rounds of laboratory evolution (Fig. 1c and Extended Data Fig. 1). In total, 41 residues, located within the putative active site and secondary coordination sphere, were individually randomized using NNK degenerate codons. Individual library variants were arrayed in 96-well plates and evaluated as clarified cell lysate using an ultra performance liquid chromatography (UPLC) assay monitoring the conversion of **1** and **2** to **3** (Supplementary Fig. 2). The most active (about 1%) clones from each round were selected for further evaluation as purified proteins and screened. Beneficial mutations identified in each round were subsequently combined by DNA shuffling.

**Extended Data Fig. 2:**
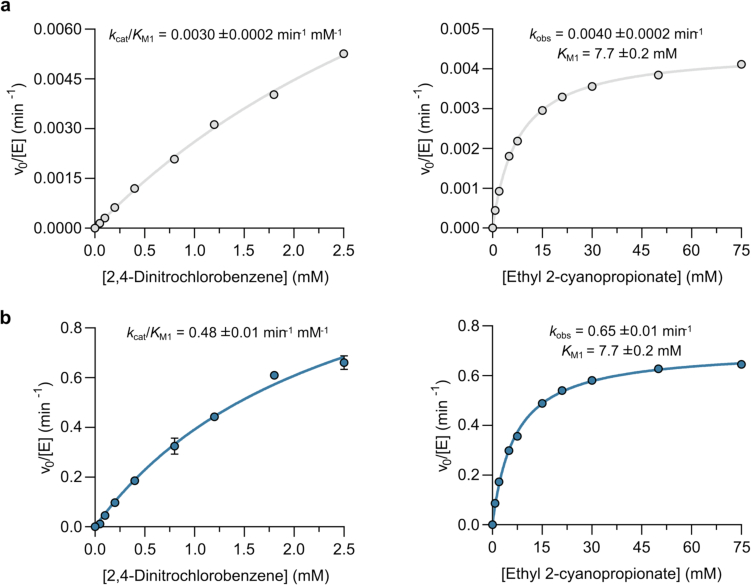
Kinetic characterization of S_N_Ar1.0 and S_N_Ar1.3. Michaelis–Menten plots for the S_N_Ar reaction between **1** and **2** catalysed by either S_N_Ar1.0 (**a**) or S_N_Ar1.3 (**b**). Assays were performed at a fixed concentration of **1** (75 mM) and varying concentrations of **2**, or a fixed concentration of **2** (2.5 mM) and varying concentrations of **1**. The plots show the averaged initial rates that were fitted to the Michaelis–Menten equation using GraphPad Prism software. Error bars represent the standard deviation of measurements made in triplicate. See Supplementary Data for source data.

**Extended Data Fig. 3:**
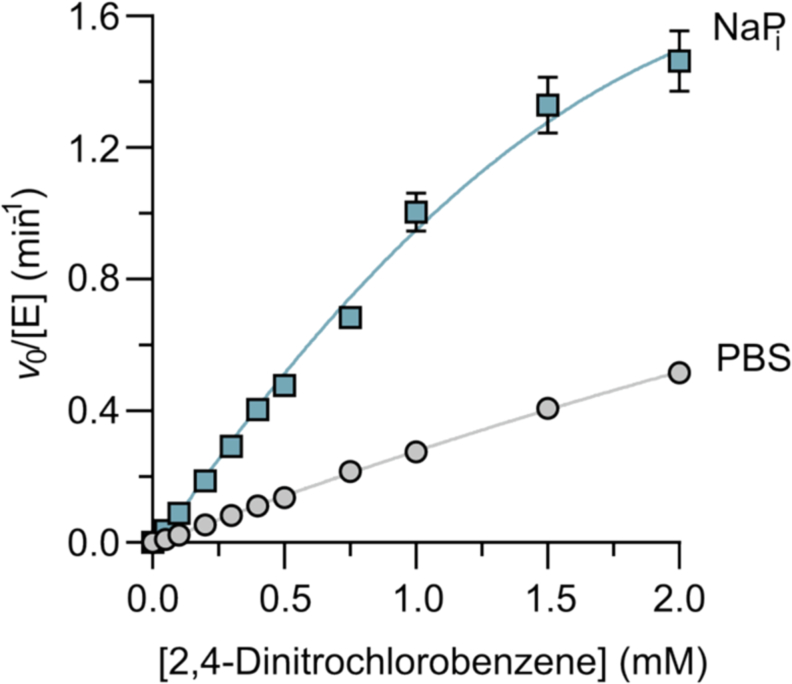
The effect of buffer composition on the Michaelis–Menten kinetic profile of S_N_Ar1.3. Michaelis–Menten plots for the S_N_Ar reaction between **1** and **2** catalysed by S_N_Ar1.3, acquired with S_N_Ar1.3 in either PBS (10 mM Na_2_HPO_4_, 1.8 mM KH_2_PO_4_, 137 mM NaCl, 2.7 mM KCl) or sodium phosphate (NaP_i_) (46.4 mM Na_2_HPO_4_, 3.6 mM NaH_2_PO_4_). Assays were performed at a fixed concentration of **1** (75 mM) and varying concentrations of **2** in PBS pH 8.0 (grey circle markers) or NaP_i_ pH 8.0 (blue square markers). The plots show the averaged initial rates that were fitted to the second-order polynomial (quadratic) using GraphPad Prism software, as saturation of **2** was not achieved. Error bars represent the standard deviation of measurements made in triplicate. See Supplementary Data for source data.

**Extended Data Fig. 4:**
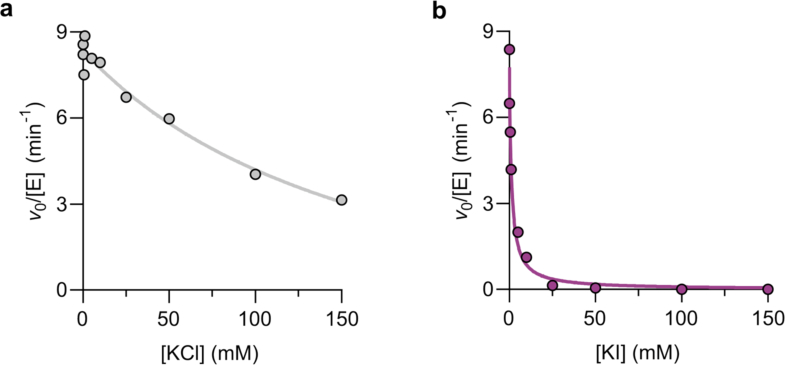
Relationship between S_N_Ar1.3 initial rate and concentration of chloride and iodide. Assays were performed at a fixed concentration of **1** (75 mM) and **5** (1 mM) with varying concentrations of either KCl (**a**) or KI (**b**). Linear fits of conversion versus time allowed determination of *v*_0_, and the *v*_0_ versus halide concentration steady-state kinetic data were fitted to the ‘[inhibitor] versus response’ equation (Y = Bottom + (Top − Bottom)/(1 + (X/IC_50_))) using GraphPad Prism, allowing for the calculation of IC_50_ values. Data points shown are averages of triplicate measurements, with error bars representing standard deviation. See Supplementary Data for source data.

Following the evaluation of approximately 4,000 clones, an S_N_Ar1.3 variant emerged containing six mutations (Fig. 1c,e and Extended Data Fig. 1). Notably, during evolution, His23, which is a key catalytic nucleophile in MBH catalysis, was mutated—excluding the possibility of this residue promoting S_N_Ar chemistry through nucleophilic catalysis^32^. Under assay conditions used during evolution, S_N_Ar1.3 affords **3** as the sole product with 93% conversion, compared with 3% conversion using S_N_Ar1.0. This improvement in catalytic performance also correlates with improvements in enantioselectivity, with S_N_Ar1.3 delivering the (*R*)-enantiomer of **3** in 96% e.e. compared with the modest 5% e.e. observed with the parent template. The absolute configuration of **3** was assigned by X-ray diffraction of optically enriched **3** obtained from a preparative-scale biotransformation (51 mg of **2**, 90% conversion, 70% isolated yield, 98% e.e. after recrystallization; Supplementary Fig. 3 and Supplementary Table 11). To further quantify the improvements in catalytic performance following evolution, we performed more detailed kinetic analysis. Assays performed at fixed concentration of **2** (2.5 mM) and variable concentrations of **1** reveal a substantial 160-fold improvement in *k*_obs_ (0.0040 ± 0.0002 and 0.65 ± 0.01 min^−1^ for S_N_Ar1.0 and S_N_Ar1.3, respectively) with minimal changes in *K*_M**1**_ (6.8 ± 0.2 and 7.7 ± 0.2 mM for S_N_Ar1.3 and S_N_Ar1.0, respectively; Extended Data Fig. 2). Subsequent assays performed under saturating concentrations of **1** also reveal a 160-fold enhancement in *k*_cat_/*K*_M**2**_ (0.0030 ± 0.0002 and 0.48 ± 0.01 min^−1^ mM^−1^ for S_N_Ar1.0 and S_N_Ar1.3 respectively; Extended Data Fig. 2). Notably, switching from phosphate-buffered saline (PBS) buffer (10 mM Na_2_HPO_4_, 1.8 mM KH_2_PO_4_, 137 mM NaCl, 2.7 mM KCl) to sodium phosphate (46.4 mM Na_2_HPO_4_, 3.6 mM NaH_2_PO_4_) leads to a further threefold increase in S_N_Ar1.3 activity (Extended Data Fig. 3), which can be attributed to enzyme inhibition at elevated concentrations of chloride (IC_50_ = 147 ± 32 mM; Extended Data Fig. 4a). Notably, iodide was found to be a more potent inhibitor of S_N_Ar1.3, with an IC_50_ value of 1.20 ± 0.05 mM (Extended Data Fig. 4b).

**Fig. 2:**
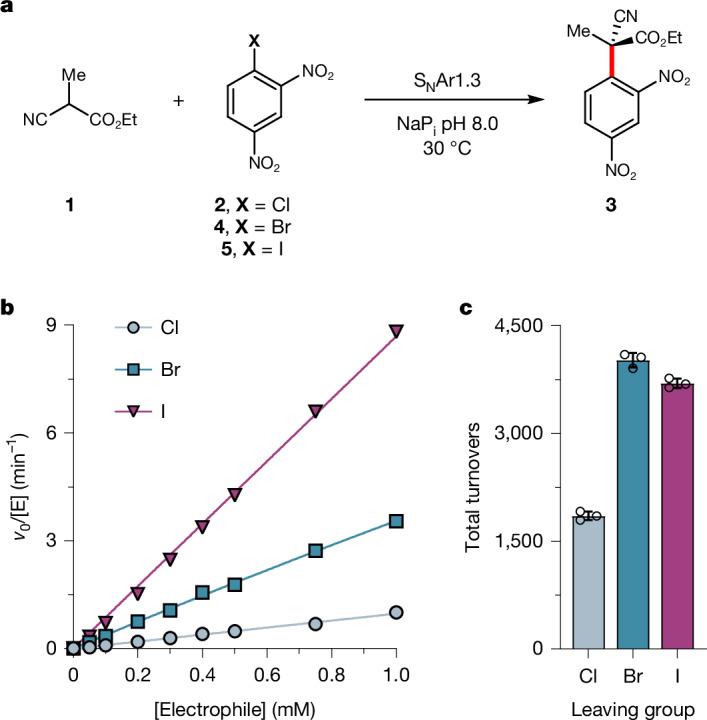
Impact of the halide leaving group on S_N_Ar1.3 activity. **a**, Chemical scheme of the S_N_Ar reaction between **1** and 2,4-dinitrohalobenzene electrophiles (**2**, **4** or **5**) catalysed by S_N_Ar1.3. **b**, Michaelis–Menten kinetic analysis of S_N_Ar1.3 at varying concentrations of **2**, **4** or **5** and saturating concentrations of nucleophile **1** (75 mM). A linear fit of *v*_0_ versus [S] was used to derive *k*_cat_/*K*_M**2**_ values of 0.97 ± 0.04, 3.67 ± 0.04 and 8.34 ± 0.11 mM^−1^ min^−1^ for **2**, **4** and **5**, respectively. Owing to solubility limits with substrates **4** and **5**, it was not possible to perform assays at higher substrate concentrations required to reach saturation. **c**, S_N_Ar1.3 can perform roughly 4,000 turnovers using **4** or **5** as substrates (Supplementary Fig. 4a). Reaction conditions: **1** (10 equiv.), 2,4-dinitrohalobenzene (**2**, 2.5 mM; **4**, 1.5 mM; **5**, 1.0 mM), S_N_Ar1.3 (0.001 mol%) in NaP_i_ pH 8.0 with 10% v/v DMSO and 0.1% w/v Pluronic F-127 at 30 °C. Data points shown are averages of triplicate measurements, with error bars representing standard deviation. See Supplementary Data for source data.

We next explored the effect of varying the halide leaving group on S_N_Ar1.3 activity and selectivity. Notably, despite performing evolution with aryl chloride **2**, enzyme activity is improved by 3.8-fold and 8.6-fold using the bromide-containing (**4**) and iodide-containing (**5**) analogues of **2**, respectively (3.67 ± 0.04 and 8.34 ± 0.11 mM^−1^ min^−1^) (Fig. 2b). This trend differs from that observed in the analogous uncatalysed background reactions, in which reactions with **5** are markedly slower than with **2** or **4**. Using its preferred aryl iodide substrate **5**, S_N_Ar1.3 can operate at a rate of 8.81 ± 0.11 mM^−1^ min^−1^ (using 1 mM of **5**; Fig. 2b) and affords product **3** in greater than 99% e.e. (Supplementary Fig. 4b). Furthermore, the enzyme is able to achieve more than 4,000 turnovers (Fig. 2c and Supplementary Fig. 4a). To demonstrate synthetic utility, we performed a preparative-scale biotransformation to produce 150 mg of **(*R*)-3** (>99% conversion, 91% isolated yield, 99% e.e.) using only 0.5 mol% of S_N_Ar1.3 (Supplementary Fig. 5). We also explored the potential of S_N_Ar1.3 to discriminate between regioisomeric aryl halide substrates. As expected, the enzyme shows no observable activity towards 3,5-dinitrobromobenzene, which is typically poorly reactive as an S_N_Ar substrate. By contrast, S_N_Ar1.3 promotes the coupling of **1** with 2,6-dinitrochlorobenzene (**6**) with high levels of stereocontrol (99% e.e.; Supplementary Fig. 6b). However, activity towards this substrate is approximately 170-fold lower than with the 2,4-dinitrochlorobenzene regioisomer **2** used for enzyme engineering (Supplementary Fig. 6c). The regioselective nature of this S_N_Ar process is further demonstrated through the reaction of **1** with an equimolar mixture of **2** and **6** as substrates, affording product **3** with high yield (97%) and regioselectivity (r.r. 71:1; Supplementary Fig. 6d).

**Fig. 3:**
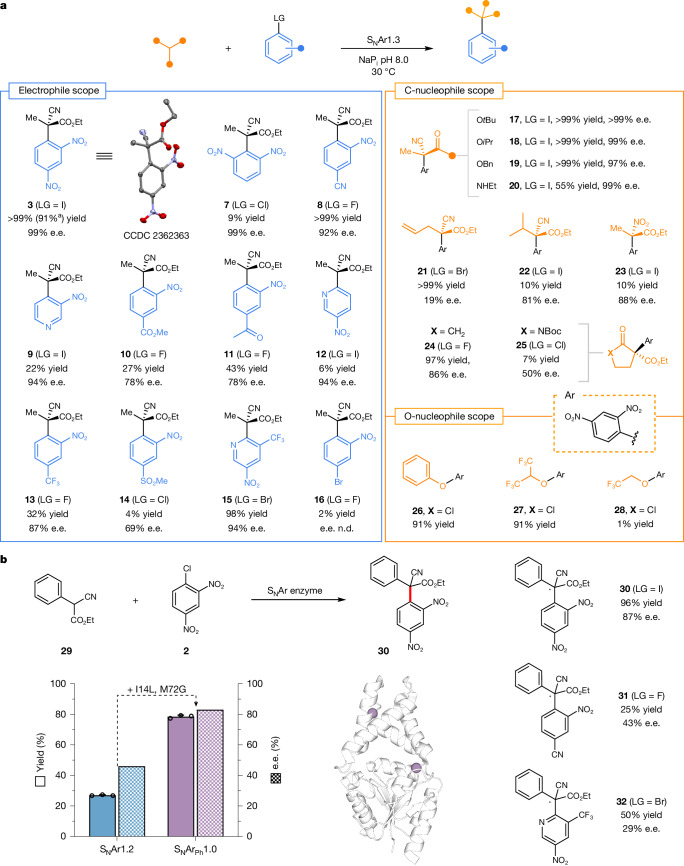
Substrate scope of S_N_Ar1.3. **a**, S_N_Ar1.3 is compatible with a variety of different electrophile (left) and nucleophile (right) coupling partners to form diverse products containing all-carbon quaternary stereocentres or nitrogen-containing quaternary stereocentres (**23**). S_N_Ar1.3 also promotes C–O bond construction using phenols or activated alcohols as nucleophiles. With the exception of **24**, the stereochemistry of the major enantiomer formed in the biotransformations was assigned by analogy to S_N_Ar1.3-derived **(*R*)-3**. The stereochemistry of **24** was assigned on the basis of previously reported chiral HPLC data^5^ (Supplementary Information). The identity of the halide leaving group on the electrophilic coupling partner was selected on the basis of commercial availability, as well as assessment of reactivity and selectivity when several substrates were available. **b**, 1,1-Diaryl quaternary carbon centres can be accessed using ethyl 2-cyano-2-phenylacetate (**29**) as the nucleophile. The most enantioselective variant from the evolutionary trajectory, S_N_Ar1.2, was further engineered to afford a double mutant S_N_Ar_Ph_1.0 with improved selectivity for the production of **30** (Extended Data Fig. 5). Structure showing the six amino acid positions mutated in S_N_Ar_Ph_1.0 mapped onto the structure of BH32.7 (PDB: 7O1D). Mutations are represented as spheres at the *C*_α_. This variant also enables the synthesis of optically enriched **31** and **32**, albeit with reduced selectivity. Reactions were performed using 0.5–5.0 mol% enzyme and 10–20 equivalents of nucleophile. Specific conditions for all reactions are presented in Supplementary Tables 1 and 2. Product yields were determined by HPLC analysis by comparison of product peak areas against standard curves of authentic racemic standards. ^a^Isolated yield from a preparative-scale reaction using S_N_Ar1.3 (0.5 mol%) with electrophile **5** to produce optically pure **(*R*)-3** (152 mg). n.d., not determined. Data points shown are averages of triplicate measurements, with error bars representing standard deviation. See Supplementary Data for source data.

To evaluate the range of transformations accessible with S_N_Ar1.3, we explored the scope towards diverse electrophile and nucleophile coupling partners (Fig. 3a, Supplementary Table 1 for further details and Supplementary Fig. 7). Notably, the enzyme tolerates a wide range of nitroarene substrates, including those containing nitrile, trifluoromethyl, ester, ketone and sulfone substituents, as well as pyridine rings (Fig. 3a) to afford S_N_Ar products with good to excellent e.e. In all cases, the desired S_N_Ar adducts were formed exclusively, with no side products observed. For selected transformations (those leading to products **8**, **9** or **15**), we assessed S_N_Ar variants from across the evolutionary trajectory. In all cases, S_N_Ar1.3 proved to be the most active and selective biocatalyst, suggesting that the mutations installed during evolution have led to general improvements in S_N_Arase performance on aromatic substrates with different substituent patterns and halide leaving groups (Supplementary Fig. 8). For the synthesis of **8**, we compared the performance of S_N_Ar1.3 with aromatic precursors containing different halide leaving groups and observed a reactivity order of F > I > Br > Cl with minimal changes in reaction selectivity (Supplementary Fig. 9). These observations suggest that the intrinsically higher reactivity of aryl fluoride electrophiles overrides the preference of S_N_Ar1.3 for larger halide leaving groups. As well as its broad electrophile substrate scope, S_N_Ar1.3 also tolerates a variety of carbon nucleophiles. Analogues of **1** containing larger ester groups (**17**, **18**, **19**), amide motifs (**20**) and 2-alkyl substituents (**21**, **22**) are well tolerated. The enzyme also accepts a cyclic β-ketoester as a substrate to afford the *C*-arylated species **24** as the sole product with high conversion and selectivity (Supplementary Fig. 10). This is in contrast with the mixtures of *O*-arylated and *C*-arylated products generated in analogous chemical transformations using stoichiometric base or small organic catalysts^5^. Beyond synthesis of all-carbon quaternary stereocentres, S_N_Ar1.3 also promotes the formation of optically enriched nitrogen-containing quaternary stereocentres (**23**) using ethyl 2-nitropropionate as a nucleophile. The resulting α-nitro ester products can be elaborated into valuable chiral motifs, including α-amino acids^33^ and α-tertiary amines^34^. Furthermore, S_N_Ar1.3 can be used for C–O bond construction using phenols or activated alcohols as nucleophiles (p*K*_a_ < 12.4) to generate biaryl ethers (**26**) or aryl alkyl ethers (**27** and **28**), respectively.

**Extended Data Fig. 5:**
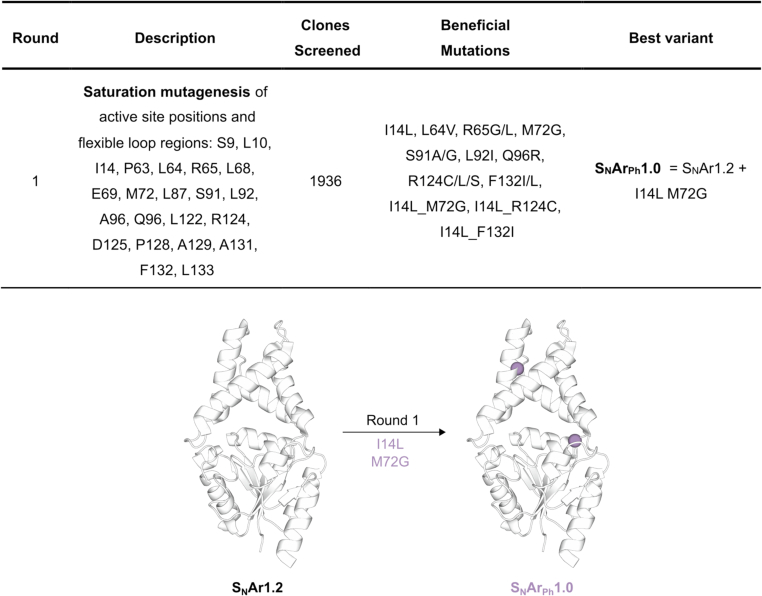
Directed evolution of S_N_Ar_Ph_1.0, an S_N_Arase compatible with nucleophile **20** that generated products with optically 1,1-diaryl quaternary carbon centres. Schematic showing the trajectory from S_N_Ar1.2 to S_N_Ar_Ph_1.0. Mutations introduced are represented as CPK spheres at the *C*_α_. One round of evolution afforded S_N_Ar_Ph_1.0, which contains two mutations. Library generation method, positions targeted, the number of clones evaluated, beneficial mutations and the most improved variant for the single round are given in the table.

Finally, we recognized the potential to apply our S_N_Ar biocatalysts for the construction of 1,1-diaryl quaternary motifs, a common structural feature in bioactive molecules^35,36^ that is challenging to synthesize in a stereocontrolled manner^37^. To explore this possibility, we evaluated a selection of our S_N_Ar variants as biocatalysts for the conversion of **2** and ethyl 2-cyano-2-phenylacetate (**29**) to product **30** (Fig. 3b and Supplementary Fig. 11). The engineered S_N_Ar1.2 variant was able to promote this transformation with modest conversion (27%) and stereocontrol (46% e.e.). To enhance activity and selectivity, we subjected S_N_Ar1.2 to an extra round of directed evolution (Extended Data Fig. 5) to afford a double mutant (S_N_Ar_Ph_1.0), which is threefold more active than the parent template and produces **30** in 84% e.e. Reaction conversion and selectivity can be further improved using aryl iodide **5** as a substrate in place of aryl chloride **2**, with product **30** formed in 96% conversion and 87% e.e. using **5** (Fig. 3b). This enzyme is also able to produce the 1,1-di(hetero)arylated products **31** and **32** (Fig. 3b and Supplementary Table 2 for further details), albeit with reduced selectivity, suggesting that S_N_Ar_Ph_1.0 will serve as a valuable template for engineering biocatalysts for the stereocontrolled synthesis of diverse 1,1-diarylated products (Supplementary Fig. 12).

## S_N_Ar1.3 structure and mechanism

To gain insights into the S_N_Ar1.3 catalytic mechanism and the origins of enhanced performance across evolution, a series of biochemical, structural and computational studies were undertaken. We first considered the possibility that S_N_Ar catalysis could proceed through the formation of enzyme–substrate covalent intermediates, as proposed for 4-chlorobenzoyl-CoA dehalogenase^24^. To explore this hypothesis, we incubated S_N_Ar1.3 and selected variants with aryl halide **5** in the absence of a nucleophilic coupling partner and monitored both halide release^38^ and changes in protein mass over time (Supplementary Fig. 13). We note that no phenolic product arising from aryl-halide hydrolysis is observed under the assay conditions, meaning that we would expect any covalent intermediates to accumulate. Using either assay, there is no evidence for the formation of covalent adducts over catalytically relevant time frames (rate of 8.8 min^−1^), suggesting that aryl-enzyme intermediates are unlikely to be involved in the S_N_Ar1.3 catalytic mechanism. We note that the Cys96 residue, which was introduced in the final round of engineering and gave a modest 2.4-fold activity increase, undergoes slow arylation on incubation of S_N_Ar1.3 with electrophile **5**, with approximately 60% of the protein modified after 10 min (Supplementary Fig. 13). A Cys96Gln mutation in S_N_Ar1.3 leads to a modest 2.7-fold activity reduction, showing that Cys96 is beneficial but not critical to S_N_Ar catalysis (Supplementary Fig. 16).

**Fig. 4:**
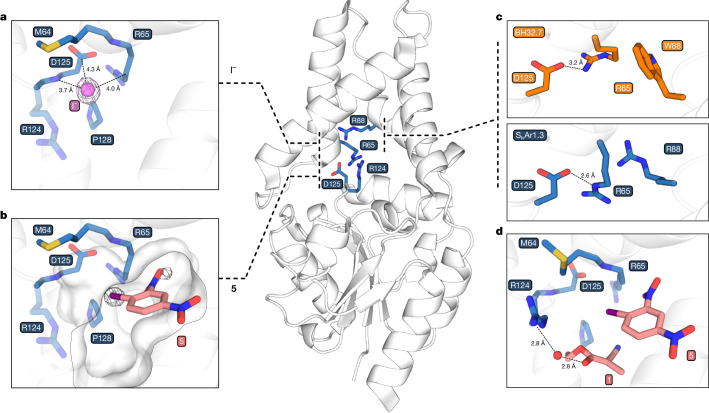
Structural and mechanistic studies of S_N_Ar 1.3. **a**, S_N_Ar1.3 crystals soaked with iodide (pink sphere) reveal a halide binding site composed of side chain and backbone interactions with residues M64, R65, R124, D125 and P128 (blue sticks). Anomalous map contoured at 14*σ* (pink mesh). Arg124 is shown as transparent, as it was not visible in the electron density owing to side chain conformational heterogeneity. **b**, S_N_Ar1.3 crystals soaked with **5** show two anomalous signals, one of which is adjacent to the iodide binding site. The major conformation of **5** binding is shown (salmon), with placement inferred from anomalous density (Extended Data Fig. 6). Although the approach of **1** from ‘above’ the plane of **5** would be occluded by the protein scaffold, a putative nucleophile binding pocket could allow for approach from ‘below’. Solvent-accessible surface shown in transparent grey. Anomalous map contoured at 9*σ* (grey mesh). **c**, Comparison of residues in S_N_Ar1.3 (blue) and BH32.7 (orange; PDB: 7O1D), which is the closest available structure to S_N_Ar1.0 and contains a single Y20A mutation remote from the catalytic site, shows how evolution has modulated polar interactions in the halide binding cavity. **d**, Representative structure from molecular dynamics simulations places **1** in the putative nucleophile binding pocket and suggests that H-bonding interactions between Arg124 and the enolate of **1** may help to position the nucleophile for selective catalysis. A water molecule is shown as a red sphere.

**Extended Data Fig. 6:**
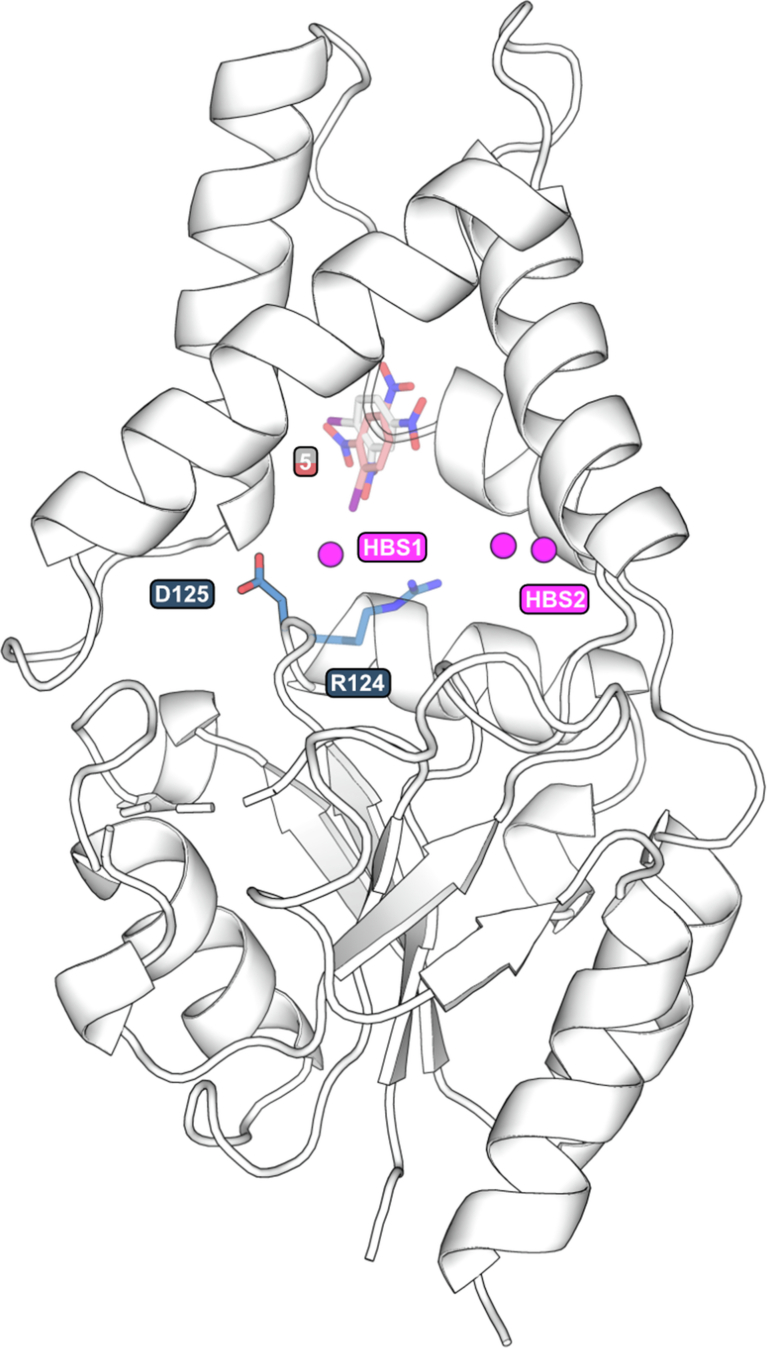
Halide binding sites in S_N_Ar1.3. Soaking S_N_Ar1.3 with iodide (pink spheres) reveals two internal halide binding sites (HBS), with two conformations of the iodide ion presented for HBS2. The most occupied site (HBS1) was further investigated with knockout studies. The two most important residues for catalysis (Arg124 and Asp125) are shown as blue sticks. Arg124 is shown as transparent, as it was not visible in the electron density owing to side chain conformational heterogeneity. The position of electrophile binding from the analogous S_N_Ar1.3 soak with **5** is shown in transparent sticks, with the major position in salmon and the minor in grey.

**Extended Data Fig. 7:**
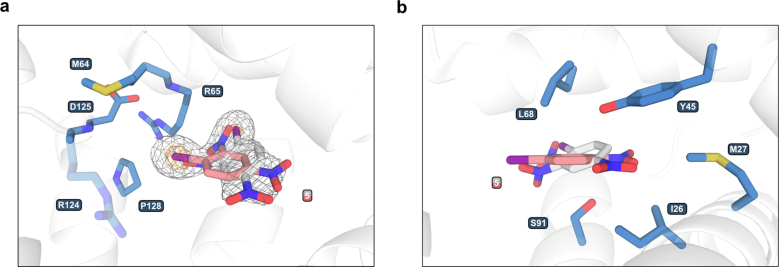
2,4-dinitroiodobenzene binding poses in S_N_Ar1.3. **a**, 2,4-dinitroiodobenzene binds S_N_Ar1.3 in two poses, one major (salmon) and one minor (grey), inferred by anomalous destiny. The major pose is adjacent to the halide binding site. Anomalous map contoured at 9*σ* (orange mesh). 2F_o_–F_c_ map contoured at 1*σ* (grey mesh). **b**, Notable residues forming the aryl halide binding pocket.

To further investigate the mechanism, an X-ray crystal structure of S_N_Ar1.3 was solved to a resolution of 1.8 Å (Supplementary Table 10). We facilitated structural analysis by a K39A mutation at the protein surface, which has negligible impact on activity (Supplementary Fig. 14) and improves data resolution. The structure superimposes well with the S_N_Ar1.0 starting template used for directed evolution, with minimal changes to the overall protein fold (root mean square deviation of 0.89 Å). Given the importance of halide binding cavities in natural dehalogenases^39^, S_N_Ar1.3 crystals were soaked with 100 mM KI before freezing. The resulting structure reveals two internal iodide binding sites (Extended Data Fig. 6), with the most occupied site (about 85%) shaped by Met64, Arg65, Arg124, Asp125 and Pro128 (Fig. 4a). Notably, Arg and Asp residues are a common feature of halide binding sites in natural proteins, despite the latter being negatively charged^40^. To explore the potential importance of the halide binding site, S_N_Ar1.3 crystals were soaked with substrate **5**. The corresponding structure revealed two distinct anomalous signals in close proximity, associated with the iodide substituent, suggesting that the substrate binds with considerable conformational heterogeneity. Notably, the major **5** pose places the halide substituent directly adjacent to the aforementioned halide binding cavity (Fig. 4b and Extended Data Fig. 7). In this pose, there is a vacant cavity below the aromatic substrate that can likely accommodate nucleophilic coupling partners (Extended Data Fig. 7 and Supplementary Fig. 15).

**Extended Data Fig. 8:**
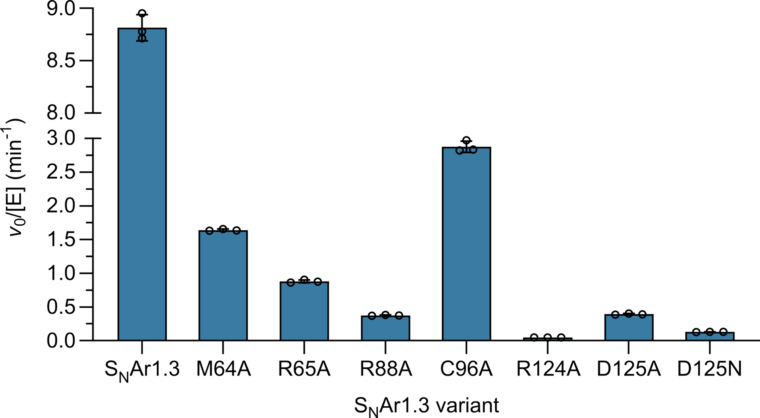
The effect of S_N_Ar1.3 point mutations on the reaction rate with substrate **5**. Bar chart comparison of *v*_0_/[E] (min^−1^) values for point mutants of S_N_Ar1.3 at residues that line the active site and halide binding cavity. Reaction conditions: **1** (75 mM), **5** (1.0 mM) and S_N_Ar1.3 variant in NaP_i_ pH 8.0 with 10% v/v DMSO at 30 °C. Enzyme concentrations: S_N_Ar1.3 (1 μΜ); M64A, R65A, R88A, C96A (1 μΜ); R124A (25 μΜ); D125A (5 μΜ); D125N (40 μΜ). Error bars represent the standard deviation of measurements made in triplicate. See Supplementary Data for source data.

Guided by the structural analysis, we performed site-directed mutagenesis of residues lining the halide binding site (Extended Data Fig. 8 and Supplementary Fig. 16). R124A and D125N/A mutations led to substantial 180-fold and 68-fold/22-fold reductions in rate, respectively, with more modest 5.4-fold and 10-fold rate reductions observed with M64A and R65A (Extended Data Fig. 8 and Supplementary Fig. 16). These assays further underscore the importance of the halide binding motif to efficient catalysis, although the large contribution made by Arg124 could also be ascribed to its role in nucleophile activation (vide infra). Comparison of the S_N_Ar1.0 and S_N_Ar1.3 structures shows how the halide binding pocket has been modulated through directed evolution. In particular, the W88R mutation results in repositioning of Arg65 to optimize its electrostatic interactions with Asp125 (Fig. 4c). Substitution of Arg88 by alanine leads to a 24-fold reduction in rate (Extended Data Fig. 8 and Supplementary Fig. 16), highlighting the importance of this extended polar network. Notably, although high levels of selectivity were preserved with the M64A, R65A and D125A halide cavity mutations, the R124A mutation led to a substantial loss of enantioselectivity (Supplementary Fig. 17), suggesting that Arg124 may also play a role in positioning and/or activating the nucleophile **1** for selective catalysis (Supplementary Fig. 18). This hypothesis is supported by molecular dynamics simulations that reveal productive conformations with the enolate of **1** forming hydrogen bonding interactions with Arg124 (Supplementary Fig. 19). In these simulations, a bridging water between the enolate and Arg124 is present in 48% of the structures and a direct Arg124-enolate hydrogen bond is observed in 28% of the frames.

## Conclusion

In summary, we have established a biocatalytic solution to S_N_Ar chemistry, one of the most important classes of transformations in the chemical industry. Here we have focused on the development of enzymes to enable stereocontrolled construction of carbon quaternary centres. Notably, we have shown that our methods can be extended to the synthesis of nitrogen-containing quaternary stereocentres, the construction of C–O bonds and to regioselective S_N_Ar processes, highlighting the broad synthetic utility of our engineered biocatalysts.

The S_N_Ar enzymes developed in this study already show impressive activities, selectivities and substrate scope, despite only sampling about 4,000 variants across the evolutionary trajectory. Deeper exploration of protein sequence space will undoubtedly deliver more potent S_N_Ar biocatalysts in the future, including those that operate on poorly activated electrophile and nucleophile coupling partners. Crucially, the structural and mechanistic studies described herein provide insights into the active-site features of S_N_Ar1.3 responsible for efficient and selective catalysis. This analysis provides an important blueprint for the de novo design of customized S_N_Ar biocatalysts with active-site geometries and arrangements of functional components required for a target transformation^41^. By combining modern protein design methods^42–44^ with high-throughput laboratory evolution, we are optimistic about the prospects of developing biocatalysts for a wide variety of valuable S_N_Ar processes, including those that are beyond the reach of existing methodologies.

## Methods

### Materials

All chemicals and biological materials were obtained from commercial suppliers. Lysozyme, DNase I and chloramphenicol were purchased from Sigma-Aldrich; polymyxin B sulfate from Apollo Scientific; LB agar, 2×YT media and l-arabinose from Formedium; *Escherichia coli* 5α, Q5 DNA polymerase, T4 DNA ligase and restriction enzymes from New England Biolabs; and oligonucleotides were synthesized by Integrated DNA Technologies.

### pBbE8k_S_N_Ar constructs

The construction of the vector pBbE8k_S_N_Ar1.0 (pBbE8k_BH32.8) is described elsewhere^12^. The gene was subcloned using *NdeI* and *XhoI* restriction sites into a pBbE8k vector, modified to include a 6×His tag following the *XhoI* restriction site.

### Protein expression and purification

For expression of S_N_Ar1.0 and variants, chemically competent *E. coli* 5α cells were transformed with the requisite pBbE8k_S_N_Ar construct. Single colonies of freshly transformed cells were cultured (18 h at 37 °C, 200 r.p.m.) in 2×YT medium (5 ml) containing kanamycin sulfate (25 µg ml^−1^). Starter cultures (500 µl) were used to inoculate 2×YT medium (50 ml) supplemented with kanamycin sulfate (25 µg ml^−1^). Cultures were grown (37 °C, 200 r.p.m.) to an optical density at 600 nm (OD_600_) of about 0.6. Protein expression was induced with the addition of l-arabinose (10 mM final concentration). Induced cultures were incubated (20 h at 25 °C) and the cells were subsequently collected by centrifugation (3,220 × *g* for 10 min). Pelleted cells were resuspended in lysis buffer (50 mM HEPES, 300 mM NaCl, 20 mM imidazole, pH 7.5) and lysed by sonication. Cell lysates were cleared by centrifugation (27,216 × *g* for 30 min) and supernatants were subjected to affinity chromatography using Ni-NTA agarose (QIAGEN). Purified protein was eluted using elution buffer (50 mM HEPES, 300 mM NaCl, 250 mM imidazole, pH 7.5). Proteins were desalted using 10DG desalting columns (Bio-Rad) with the requisite storage buffer and analysed by sodium dodecyl sulfate–polyacrylamide gel electrophoresis. Proteins were aliquoted, flash-frozen in liquid nitrogen and stored at −80 °C. Protein concentrations were determined by measuring the absorbance at 280 nm using calculated extinction coefficients (ExPASy ProtParam); extinction coefficient of 27,390 M^−1^ cm^−1^ for S_N_Ar1.0 and 21,890 M^−1^ cm^−1^ for S_N_Ar1.1 to 1.3 and S_N_Ar_Ph_1.0.

### Protein mass spectrometry

Purified protein samples were buffer-exchanged into 0.1% acetic acid using a 10 k MWCO Vivaspin unit (Sartorius) and diluted to a final concentration of 0.5 mg ml^−1^. Mass spectrometry was performed using a 1200 series Agilent LC system, with a 5 µl injection into 5% acetonitrile (with 0.1% formic acid) and desalted inline for 1 min. Protein was eluted over 1 min using 95% MeCN with 5% H_2_O. The resulting multiply charged spectrum was analysed using an Agilent 6510 Q-TOF instrument and deconvoluted using Agilent MassHunter software.

To prepare protein with the Cys96 residue arylated (Supplementary Fig. 13c), S_N_Ar1.3 (200 µM final concentration) was incubated with **5** (1 mM) for 1 h at 30 °C. As a control, an S_N_Ar1.3 C96A variant was incubated under the same conditions. Samples were then characterized by mass spectrometry as described above.

### Library construction

Rounds 1, 2 and 3: saturation mutagenesis. Positions were individually randomized using degenerate NNK codons. DNA libraries were constructed by overlap extension polymerase chain reaction (PCR). Primers for library generation are given in Supplementary Table 8. Assembled genes and pBbE8k vector were digested using *NdeI* and *XhoI* endonucleases, gel-purified and subsequently ligated using T4 DNA ligase in a 4:1 ratio, respectively. Ligations were transformed into *E. coli* 5α cells, the resulting colonies were pooled and plasmid DNA was extracted using a Miniprep Kit (QIAGEN) to yield plasmid DNA for each library. Sequencing was performed by Source BioScience.

### Shuffling by overlap extension PCR

After each round of screening, beneficial mutations were combined by DNA shuffling of fragments generated by overlap extension PCR. Primers were designed that encoded either the parent amino acid or the identified mutation. These primers were used to generate short fragments that were gel-purified and mixed for assembly of the full-length gene by overlap extension PCR. Final full-length genes contain all possible combinations of mutations at specified positions. Genes were cloned as described above.

### Library screening

For protein expression and screening, all transfer and aliquoting steps were performed using a Hamilton liquid-handling robot. Chemically competent *E. coli* 5α cells were transformed with the appropriate library plasmids. Freshly transformed colonies were used to inoculate 2×YT medium (150 μl) supplemented with kanamycin sulfate (25 μg ml^−1^) in Corning Costar 96-well microtitre round-bottom plates. Each plate also contained six freshly transformed clones of the parent template and two clones of pBbE8k_RFP as an internal reference. Plates were incubated overnight (30 °C, 80% humidity, 850 r.p.m.), then an aliquot of overnight culture (20 µl) was used to inoculate 2×YT medium (480 μl) supplemented with kanamycin sulfate (25 μg ml^−1^). The cultures were incubated (30 °C, 80% humidity, 850 r.p.m.) until an OD_600_ of about 0.6 was reached and l-arabinose was added (10 mM final concentration). Induced plates were incubated (20 h, 30 °C, 80% humidity, 850 r.p.m.). Cells were collected by centrifugation (2,900 × *g* for 10 min). The supernatant was discarded and the pelleted cells were resuspended in lysis buffer (400 μl: PBS buffer at requisite pH supplemented with lysozyme (1.0 mg ml^−1^), polymyxin B (0.5 mg ml^−1^) and DNase I (10 μg ml^−1^)) and incubated (2 h, 30 °C, 80% humidity) with shaking (850 r.p.m.). Cell debris was removed by centrifugation (2,900 × *g*, 10 min).

#### Rounds 1 and 2

Clarified lysate (50 μl) was added to a 96-well microtitre plate and then the reaction initiated by the addition of assay mix (50 μl) containing 2,4-dinitrochlorobenzene (2.5 mM final concentration), ethyl-2-cyanopropionate (25 mM final concentration) in PBS pH 8.0 with dimethyl sulfoxide (DMSO) (10% v/v final concentration). Reactions were heat-sealed and incubated overnight (30 °C, 850 r.p.m., 80% humidity) and then quenched with the addition of MeCN (100 μl), heat-sealed and incubated (1 h, 30 °C, 80% humidity, 850 r.p.m.). Precipitated proteins were removed by centrifugation (2,900 × *g*, 10 min). An aliquot of the clarified, quenched reaction mixture (100 µl) was transferred to a Greiner 96-well polypropylene microtitre plate and heat-sealed with pierceable foil. Reactions were evaluated by high-performance liquid chromatography (HPLC) analysis as described below.

#### Round 3

Clarified lysate (50 μl) was added to a 96-well microtitre plate and then the reaction initiated by the addition of assay mix (50 μl) containing 2,4-dinitrochlorobenzene (2.5 mM final concentration), ethyl-2-cyanopropionate (5 mM final concentration) in PBS pH 8.0 with DMSO (10% v/v final concentration). Reactions were heat-sealed and incubated overnight (30 °C, 850 r.p.m., 80% humidity) and then quenched with the addition of MeCN (100 μl), heat-sealed and incubated (1 h, 30 °C, 80% humidity, 850 r.p.m.). Precipitated proteins were removed by centrifugation (2,900 × *g*, 10 min). An aliquot of the clarified, quenched reaction mixture (100 µl) was transferred to a Greiner 96-well polypropylene microtitre plate and heat-sealed with pierceable foil. Reactions were evaluated by HPLC analysis as described below.

#### S_N_Ar_Ph_1.0 evolution

Clarified lysate (50 μl) was added to a 96-well microtitre plate and then the reaction initiated by the addition of assay mix (50 μl) containing 2,4-dinitrochlorobenzene (1.0 mM final concentration), ethyl 2-cyano-2-phenylacetate (2.0 mM final concentration) in PBS pH 6.0 with DMSO (20% v/v final concentration). Reactions were heat-sealed and incubated overnight (30 °C, 850 r.p.m., 80% humidity) and then quenched with the addition of MeCN (100 μl), heat-sealed and incubated (1 h, 30 °C, 80% humidity, 850 r.p.m.). Precipitated proteins were removed by centrifugation (2,900 × *g*, 10 min). An aliquot of the clarified, quenched reaction mixture (100 µl) was transferred to a Greiner 96-well polypropylene microtitre plate and heat-sealed with pierceable foil. Reactions were evaluated by HPLC analysis as described below.

Following each round, the most active variants (about 1%) were rescreened as purified proteins using the HPLC assay. Proteins were produced and purified as described above. However, starter cultures were inoculated from glycerol stocks prepared from the original overnight cultures.

### General procedure for analytical-scale biotransformations

Analytical-scale biotransformations (typically 100 μl) were performed in 96-well microtitre plates or microcentrifuge tubes (1.5 ml) using **1** (10, 15 or 25 mM), **2**, **4** or **5** (1.0, 1.5 or 2.5 mM) and the relevant S_N_Ar variant at the specified concentration in PBS or NaP_i_ (pH 8.0) with 10% v/v DMSO co-solvent at 30 °C for the specified time period. For analysis of conversion by reverse-phase UPLC, reactions were quenched with MeCN (1 volume). Quenched reactions were shaken (850 r.p.m.) for 30 min. Precipitated protein was removed by centrifugation (2,900 × *g* or 14,000 × *g* for 15 min, for reactions in 96-well microtitre plates or microcentrifuge tubes, respectively) and supernatants were transferred to a fresh plate for UPLC analysis (see ‘Chromatographic analysis’ section). For normal-phase chiral HPLC analysis, the substrates and products were extracted with methyl *tert*-butyl ether (MTBE; 2 volumes). Precipitated protein was removed by centrifugation (14,000 × *g* for 10 min), the organic phase was obtained and directly injected onto the normal-phase HPLC.

### General procedure for substrate scope biotransformations

Analytical-scale biotransformations (typically 100 μl) for the substrate profile (Fig. 4) were performed in 96-well microtitre plates or microcentrifuge tubes (1.5 ml) using the specified electrophile and nucleophile with S_N_Ar1.3 or S_N_Ar_Ph_1.0 in NaP_i_ pH 8.0 with 10% v/v DMSO as co-solvent or PBS pH 6.0 with 20% v/v at 30 °C, respectively (Supplementary Tables 1 and 2). For analysis of conversion by reverse-phase UPLC, reactions were quenched with MeCN (1 volume). Quenched reactions were shaken (850 r.p.m.) for 30 min. Precipitated protein was removed by centrifugation (2,900 × *g* or 14,000 × *g* for 15 min, for reactions in 96-well microtitre plates or microcentrifuge tubes, respectively) and supernatants were transferred to a fresh plate for UPLC analysis (see ‘Chromatographic analysis’ section). For normal-phase chiral HPLC analysis, the substrates and products were extracted with MTBE (2 volumes). Precipitated protein was removed by centrifugation (14,000 × *g* for 10 min), the organic phase was separated and directly injected onto the normal-phase HPLC.

### Competition experiment between regioisomers **2** and **6**

The biotransformations (500 μl) were performed in microcentrifuge tubes (1.5 ml) using **1** (10 mM), **2** and **6**, (1.0 mM) and S_N_Ar1.3 (50 μM) in NaP_i_ (pH 8.0) with 10% v/v DMSO co-solvent at 30 °C for 3 h. For analysis of conversion by reverse-phase UPLC, the reactions were quenched with MeCN (1 volume). Quenched reactions were shaken (850 r.p.m.) for 30 min. Precipitated protein was removed by centrifugation (14,000 × *g*) for 15 min and the supernatant was transferred to a fresh plate for UPLC analysis (see ‘Chromatographic analysis’ section).

### Chromatographic analysis

We performed UPLC analysis on a 1290 Infinity II LC system (Agilent) with a Kinetex 5 µm XB-C18 100 Å LC Column, 50 × 2.1 mm (Phenomenex). The separation methods for all substrates/products and extinction coefficients used to calculate the conversion are reported in Supplementary Tables 3 and 4. Analytical-scale biotransformations, substrate standards and chemically synthesized product standards (**3** to **32**) were characterized using an injection volume of 3 μl and eluted over 15 min using a gradient of 5–95% MeCN in MQ H_2_O with 0.1% TFA at 1 ml min^−1^. Peaks were assigned by comparison with chemically synthesized standards and the peak areas were integrated using Agilent’s OpenLab software. We performed chiral analysis using a HPLC 1260 system (Agilent). For all products, the major stereoisomer formed in the biotransformations was assigned on the basis of an analogy to S_N_Ar1.3-derived **(*R*)-3**. Peaks were assigned by comparison with chemically synthesized standards and peak areas were integrated using Agilent’s OpenLab software. Separation methods for all product enantiomers used to determine e.e. are reported in Supplementary Table 5.

### Kinetic characterization

Initial velocity (*v*_0_) versus [ethyl 2-cyanopropionate] kinetic data were measured using His_6_-tagged purified S_N_Ar1.0 (125 μM) and S_N_Ar1.3 (10 μΜ), a fixed concentration of **2** (2.5 mM) and varying concentrations of **1** (3.5–75.0 mM). Reactions were performed in PBS or NaP_i_ pH 8.0 with 10% v/v DMSO co-solvent and were incubated at 30 °C with shaking (850 r.p.m.). S_N_Ar1.0 was sampled at 60-min intervals from 1 to 6 h and S_N_Ar1.3 was sampled every 10 min from 10 to 60 min. Samples were quenched with MeCN (2 volumes) and analysed by UPLC as described above (see ‘Chromatographic analysis’ section). *v*_0_ versus [2,4-dinitrochlorobenzene] kinetic data in PBS pH 8.0 were measured using a fixed concentration of **1** (75 mM) and varying concentrations of **2** (0.05–2.50 mM) as described above. *v*_0_ versus [2,4-dinitrochlorobenzene], [2,4-dinitrobromobenzene] and [2,4-dinitroiodobenzene] kinetic data in NaP_i_ pH 8.0 were measured using a fixed concentration of **1** (75 mM) and varying concentrations of electrophile (0.05–2.50 mM) and S_N_Ar1.3 (1 μM) as described above. The plots of the averaged initial rates were fitted to the Michaelis–Menten equation using Origin software (equation (1)):

1 $${\rm{Y}}={V}_{\max }\times {\rm{X}}/({K}_{{\rm{M}}}+{\rm{X}})$$

### Halide inhibition characterization

Initial velocity (*v*_0_) versus [halide] kinetic data were measured using His_6_-tagged purified S_N_Ar1.3 (1 μΜ) and a fixed concentration of **5** (1 mM) and **1** (75 mM), with varying concentrations of either KI or KCl (0.1–150.0 mM). Reactions were performed in 50 mM NaP_i_ pH 8.0 with 10% v/v DMSO co-solvent and were incubated at 30 °C with shaking (850 r.p.m.). Reactions were sampled at 10-min intervals for 40 min. Samples were quenched with MeCN (2 volumes) and analysed by UPLC as described above (see ‘Chromatographic analysis’ section). Linear fits of conversion versus time allowed determination of *v*_0_, and the *v*_0_ versus halide concentration steady-state kinetic data were fitted using the ‘[inhibitor] versus response’ equation using GraphPad Prism (equation (2)) allowing for the calculation of IC_50_ values:

2 $${\rm{Y}}={\rm{Bottom}}+({\rm{Top}}-{\rm{Bottom}})/(1+({\rm{X}}/{{\rm{IC}}}_{50}))$$

### Total turnover numbers

S_N_Ar1.3-catalysed (0.001 mol%) biotransformations were performed in microcentrifuge tubes (1.5 ml) using **1** (10 equiv) and **2**, **4** or **5** (2.5, 1.5 or 1.0 mM) in NaP_i_ pH 8.0 with 10% v/v DMSO and 0.1% w/v Pluronic F-127 (Fig. 3b and Supplementary Fig. 4b). Reactions were incubated at 30 °C with shaking (850 r.p.m.) and samples were taken at 13, 37, 60, 84, 111 and 140 h. For UPLC analysis, reactions were quenched at the stated time points with the addition of MeCN (1 volume). Samples were vortexed and precipitated proteins were removed by centrifugation (14,000 × *g* for 10 min), followed by UPLC analysis.

### Iodide quantification assay

The details of the enzymatic iodide sensing assay have been reported previously^38^. A *Curvularia inaequalis* vanadium-dependent chloroperoxidase (*Ci*VCPO) variant^45^ was used to oxidize I^−^ to HOI, resulting in a sequential oxidation of the chromogen 3,3′,5,5′-tetramethylbenzidine (TMB) and an increase in absorbance at 570 nm. Procedures for *Ci*VCPO expression, purification and activity determination were as described in the referenced study. S_N_Ar variants (200 µM) were incubated with **5** (1 mM) for 10 min and an aliquot (12 µl) of these reactions or KI standards was then added to iodide assay reagent (100 μl) in a transparent 96-well polystyrene microtitre plate. Absorbance at 570 nm was monitored spectrophotometrically over 10 min using a CLARIOstar plate reader and the initial rate was calculated. The iodide assay reagent contained *Ci*VCPO (26 U ml^−1^), TMB (2 mM), H_2_O_2_ (2 mM) and sodium orthovanadate (1 mM) in NaP_i_ (20 mM, pH 6.0) with 10% v/v DMSO as a co-solvent.

### Preparative-scale biotransformation with **2**

A 500-ml Erlenmeyer flask was charged sequentially with **2** (50.6 mg, 0.25 mmol, 1.00 equiv), 15 ml DMSO, **1** (153 μl, 1.25 mmol, 5 equiv) and PBS pH 8.0 (35 ml). The solution was swirled to reach homogeneity before the addition of a stock solution of 100 μM of S_N_Ar1.3 in PBS pH 8.0 (50 ml, 0.005 mmol, 2 mol%). The flask was sealed with a foam bung and foil and then incubated (30 °C, 120 r.p.m.) for 40 h. After this time, an aliquot (50 μl) of the reaction mixture was analysed by UPLC, showing 90% conversion. The reaction mixture was transferred to a separating funnel and extracted with EtOAc (3 × 100 ml), the organic layers washed with brine and dried over MgSO_4_, followed by concentration in vacuo. The crude product was purified by flash column chromatography (SiO_2_, celite dry loading, 10–35% EtOAc in hexane) to afford (*R*)-ethyl 2-cyano-2-(2,4-dinitrophenyl)propanoate (**3**) as a pale yellow solid (50 mg, 70% isolated yield, 94% e.e.). The product was recrystallized from EtOAc/hexane to give pale yellow needles (25 mg, 98% e.e.), which were used for X-ray diffraction to determine the absolute configuration of **3**. Spectral data were consistent with the synthetic standard. ${[\alpha ]}_{D}^{24.3}=+\,113.0^\circ$ (*c* = 0.46, CHCl_3_). *R*_*f*_ 0.28 (35% EtOAc in hexane) [UV]. Melting point: 116 °C (hexane). ^1^H NMR (500 MHz, CDCl_3_): δ 9.02 (d, *J* = 2.5 Hz, 1H), 8.61 (dd, *J* = 8.7, 2.4 Hz, 1H), 8.07 (d, *J* = 8.7 Hz, 1H), 4.42–4.29 (m, 2H), 2.24 (s, 3H), 1.37 (t, *J* = 7.1 Hz, 3H). ^13^C{^1^H} NMR (126 MHz, CDCl_3_): δ 166.1, 148.3, 136.9, 131.25, 128.3, 121.9, 117.4, 64.4, 47.4, 24.8, 14.0. HRMS: (ESI^+^) calcd for C_12_H_12_O_3_N_6_ ([M + H]^+^): 294.0721, found: 294.0720.

### Preparative-scale biotransformation with **5**

A 1-l Erlenmeyer flask was charged sequentially with **5** (161.7 mg, 0.55 mmol, 1.00 equiv) in DMSO (30 ml), **1** (699 μl, 5.5 mmol, 10 equiv) in DMSO (25 ml) and S_N_Ar1.3 in NaP_i_ pH 8.0 (495 ml at 5.5 μM, 5 μM final concentration). The flask was sealed with a foam bung and foil and then shaken in an incubator (30 °C, 200 r.p.m.) for 20 h. After this time, an aliquot (50 μl) of the reaction mixture was taken, quenched and analysed by UPLC, showing 100% conversion of **5**. The reaction mixture was transferred to a separating funnel and extracted with EtOAc (3 × 500 ml), the organic layers were combined and washed with water (3.0 200 ml) and brine and then dried over MgSO_4_, followed by concentration in vacuo. The crude product was purified by flash column chromatography (SiO_2_, loaded in CH_2_Cl_2_, 10–35% EtOAc in hexane) to afford (*R*)-ethyl 2-cyano-2-(2,4-dinitrophenyl)propanoate (**3**) as a pale yellow solid (148 mg, 91% yield, 99% e.e.). Spectral data were consistent with the synthetic standard. ${[\alpha ]}_{D}^{\,24.3}=+117.5^\circ$ (*c* = 0.46, CHCl_3_). *R*_*f*_ 0.28 (35% EtOAc in hexane) [UV]. Melting point: 116 °C (hexane). ^1^H NMR (400 MHz, CDCl_3_): δ 9.00 (d, *J* = 2.4 Hz, 1H), 8.59 (dd, *J* = 8.7, 2.5 Hz, 1H), 8.05 (d, *J* = 8.7 Hz, 1H), 4.33 (qq, *J* = 6.8, 3.6 Hz, 2H), 2.21 (s, 3H), 1.34 (t, *J* = 7.1 Hz, 3H). ^13^C{^1^H} NMR (101 MHz, CDCl_3_): δ 166.1, 148.3, 148.0, 136.8, 131.2, 128.3, 121.9, 117.4, 64.4, 47.4, 24.8, 14.0. HRMS: (ESI^+^) calcd for C_12_H_12_O_3_N_6_ ([M + H]^+^): 294.0721, found: 294.0723.

### Protein crystallization, refinement and model building

As we had observed, halide inhibition for S_N_Ar1.3 samples for crystallization were purified in the absence of Cl^−^ by removing NaCl from all buffers in the protein purification procedures described above. Two of the structures (ligand free and **5** bound) contained the extra K39A mutation, as this improved the resolution of the corresponding diffraction data. K39A had a minimal effect on S_N_Ar1.3 rate or e.e. values (Supplementary Fig. 14). Crystals of S_N_Ar1.3 or S_N_Ar1.3(K39A) were prepared by mixing 300 nl PACT condition E11 (0.2 M sodium citrate tribasic dihydrate and 20% w/v PEG 3350) with 20 nl S_N_Ar1.3 seed stock and 280 nl of 20 mg ml^−1^ S_N_Ar1.3 in 50 mM NaP_i_. All trials were conducted by sitting vapour drop diffusion with incubation at 4 °C. Crystals were cryoprotected in reservoir solution supplemented with 10% w/v PEG 200 and flash-cooled in liquid N_2_. For the I^−^ soak, KI was added to the drop at a final concentration of 100 mM before freezing. To obtain the complex with **5**, the **5** solid was added to the drop and incubated overnight before crystal harvesting and freezing. The data were collected from single crystals at Diamond Light Source and subsequently scaled and reduced with Xia2 (ref. ^46^). Preliminary phasing was performed by molecular replacement in Phaser using BH32.7 (PDB: 7O1D) as a search model. Iterative cycles of rebuilding and refinement were performed in COOT^47^ and Phenix.refine^48^, respectively. The complete data collection and refinement statistics are provided in Supplementary Table 10. Coordinates and structure factors have been deposited in the Protein Data Bank (PDB) under accession numbers 9FUG, 9FUL and 9FUO.

### **(*R*)-3** data collection

Crystals suitable for diffraction were isolated by recrystallization from EtOAc/hexane. Single-crystal X-ray diffraction data for compound **3** were collected at 100 K on a Rigaku XtaLAB AFC-11 four-circle goniometer equipped with a HyPix-6000HE detector and Oxford Cryosystems. Data were collected with a dual-source Rigaku FR-X rotating anode using Cu Kα (*λ* = 1.54184 Å) radiation. Data were collected using the CrysAlisPro program.

### **(*R*)-3** crystal structure determination and refinements

Data processing and reduction was performed with CrysAlisPro. Empirical absorption correction was applied using spherical harmonics, implemented with the SCALE3 ABSPACK algorithm. The crystal structure was solved and refined using the SHELX suite of programs in Olex2 (refs. ^49,50^). All non-hydrogen atoms were refined anisotropically. Hydrogen atom positions were calculated and refined with fixed isotropic displacement parameters. The complete data collection and refinement statistics are provided in Supplementary Table 10. Absolute structure determination was based on anomalous dispersion. The absolute configuration of compound **3** was found to be (*R*).

Crystallographic data has been deposited with the CCDC number 2362363.

### Molecular docking

Putative binding poses for substrate **5** were generated by molecular docking performed with AutoDock Vina^51^, using AutoDockTools^52^ to assign hydrogen atoms to the crystal structure and generate input files. Residues R124 and L68 were flexible during docking, whereas the rest of the enzyme was kept rigid. 20 binding modes were generated with an exhaustiveness of 50, with a 20 Å × 20 Å × 20 Å search volume centred on the reacting carbon atom in the reactive pose of the crystal structure. There is very little difference in computed binding energy for the predicted binding poses, with the first 14 poses all within 1 kcal mol^−1^. Pose 7 was selected as it places the iodide in a very similar position as in the crystal structure (Fig. 4d), with a computed binding energy of −5.8 kcal mol^−1^ (0.6 kcal mol^−1^ higher than the first binding pose). Nucleophile **1** was then docked into the crystal structure with pose 7 of substrate **5** and the corresponding coordinates of Arg124 and Leu68 added, using the same parameters, keeping Arg124 flexible. There is only a 0.9 kcal mol^−1^ difference between the 20 identified binding poses. Pose 15, with a computed binding energy of −4.0 kcal mol^−1^ (0.7 kcal mol^−1^ higher than the first binding pose) is consistent with nucleophilic attack with the observed stereochemistry and overlaps with one of the ethylene glycol molecules in the active site of the crystal structure. The docking poses are shown in Supplementary Fig. 15.

### Molecular dynamics simulations

Bonding parameters for nucleophile **1** and substrate **5** were generated using the AmberTools antechamber module^53^ with charges parameterized by RESP fitting to the HF/6-31 G(d,p) electron density of a B3LYP/6-31 + G(d,p) structure optimized in Gaussian 16 Revision C.01 (ref. ^54^). Molecular dynamics simulations were then carried out using GROMACS 2018 (refs. ^55,56^) with the AMBER14 force field^57^ with a solvation box with a minimum 10 Å buffering distance around the protein and counterions generated using AmberTools, retaining crystallographic waters, for a total of 61,696 atoms.

A 3.0 Å harmonic restraint with force constant 4 kJ mol^−1^ Å^−2^ was applied to the donor–acceptor distance to hold the nucleophile and electrophile close to a reactive conformation during the molecular dynamics simulation. Simulations were performed using constant temperature (velocity-rescaling thermostat^58^, 300 K) and pressure (Parrinello–Rahman barostat^59^, 1 bar), 10 Å van der Waals and electrostatic cut-offs, particle mesh Ewald for long-range electrostatics, LINCS bond constraints^60^, periodic boundary conditions and a 2-fs time step. The protocol was as follows: (1) energy minimization with (a) 10 kJ mol^−1^ Å^−2^ constraints on the protein heavy atoms (not hydrogens), (b) 1 kJ mol^−1^ Å^−2^ constraints on the protein heavy atoms, (c) no constraints; (2) 1 ns constant volume (NVT) equilibration of the solvent with 10 kJ mol^−1^ Å^−2^ constraints on the protein heavy atoms; (3) three 1 ns constant pressure (NPT) equilibration stages with the same decreasing position constraints as for energy minimization; (4) 500 ns of production run. Root mean square deviations for the production run as well as the distance between Arg124 and enolate O^−^ are shown in Supplementary Fig. 19. For the hydrogen bonding, direct hydrogen bonds were identified using the GROMACS hbond tool and bridging hydrogen bonds defined as having a water molecule with the oxygen atom within 2.4 Å of the R124 hydrogen at the same time as one hydrogen atom within 2.4 Å of the enolate O^−^. We carried out hydrogen bonding analysis with CPPTRAJ^61^ in AMBER18 (ref. ^62^).

## Online content

Any methods, additional references, Nature Portfolio reporting summaries, source data, extended data, supplementary information, acknowledgements, peer review information; details of author contributions and competing interests; and statements of data and code availability are available at 10.1038/s41586-025-08611-0.

## Supplementary information

Supplementary Information Supplementary Information, including Supplementary Figs. 1–19, Supplementary Tables 1–11 and further references.

Supplementary Data Source data for Figs. 1–3, Extended Data Figs. 2–4 and 8, and Supplementary Figs. 1, 3, 4, 6, 8, 9, 11–14, 16 and 17.

## Data Availability

Coordinates and structure factors have been deposited in the Protein Data Bank under accession numbers 9FUG, 9FUL and 9FUO. Crystallographic data for the structure of **3** reported in this article have been deposited at the Cambridge Crystallographic Data Centre, under deposition number CCDC 2362363. Copies of the data can be obtained free of charge at https://www.ccdc.cam.ac.uk/structures. The data supporting the findings of this study are available in the paper and its Supplementary Information files.
