# Supplementary material for: Engineered enzymes for enantioselective nucleophilic aromatic substitutions
Source: Nature. 2025 Jan 15;639(8054):375–81. doi: 10.1038/s41586-025-08611-0 (PMC11903332; doi:10.1038/s41586-025-08611-0)
Supplement: Supplementary file 1 — Supplementary Information, including Supplementary Figs. 1–19, Supplementary Tables 1–11 and further references. [file 41586_2025_8611_MOESM1_ESM.pdf]

nature portfolio

https://doi.org/10.1038/s41586-025-08611-0

Supplementary information

Engineered enzymes for enantioselective nucleophilic aromatic substitutions

In the format provided by the authors and unedited

Engineered Enzymes for Enantioselective Nucleophilic Aromatic Substitutions

Thomas M. Lister, George W. Roberts, Euan J. Hossack, Fei Zhao, Ashleigh J. Burke, Linus O. Johannissen, Florence J. Hardy, Alexander A. V. Millman, David Leys, Igor Larrosa*, Anthony P. Green*

Table of Contents

Supplementary Figures 4

Supplementary Fig. 1 | Identification of a suitable starting point for evolution 4

Supplementary Fig. 2 | UPLC chromatograms of the reaction profile across the evolutionary trajectory 5

Supplementary Fig. 3 | NMR data of (*R*)-3 produced from a preparative-scale biotransformation using 2,4-dinitrochlorobenzene 6

Supplementary Fig. 4 | The effect of halide leaving group on turnover, activity and selectivity for $S_NAr1.3$. 7

Supplementary Fig. 5 | NMR data of (*R*)-3 produced from a preparative-scale biotransformation using 2,4-dinitroiodobenzene 8

Supplementary Fig. 6 | $S_NAr1.3$ selectivity and reaction rate with regioisomeric electrophile substrates 2 and 6 9

Supplementary Fig. 7 | Electrophiles and nucleophiles that are not tolerated as substrates by $S_NAr1.3$. 10

Supplementary Fig. 8 | Yield and selectivity for different electrophiles improves across evolution. 11

Supplementary Fig. 9 | The effect of leaving group on the activity and selectivity for the 4-halo-3-nitrobenzonitrile electrophile. 12

Supplementary Fig. 10 | $S_NAr1.3$ does not suffer competing *C*- and *O*-arylation with β-ketoester nucleophiles. 13

Supplementary Fig. 11 | Identification of a suitable starting point for evolving towards 1,1-diaryl products. 14

Supplementary Fig. 12 | $S_NAr_{Ph}1.0$ is more active and selective than other S_NAr variants for a different electrophile 15

Supplementary Fig. 13 | Investigating the formation of an enzyme-substrate covalent intermediate using an iodide release assay. 16

Supplementary Fig. 14 | The K39A mutation installed to improve the resolution of diffraction data leads to minimal effects on rate and selectivity. 17

Supplementary Fig. 15 | Molecular docking of substrate 5 and nucleophile 1. 18

Supplementary Fig. 16 | The effect of $S_NAr1.3$ point mutations on the reaction rate with substrate 5. 20

Supplementary Fig. 17 | The effect of mutating $S_NAr1.3$ active site residues on conversion and selectivity. 21

Supplementary Fig. 18 | Proposed substrate activation mode in $S_NAr1.3$ 22

Supplementary Fig. 19 | Molecular dynamics simulations. 23

Supplementary Tables 24

Supplementary Table 1 | Substrate scope of $S_NAr1.3$. 24

Supplementary Table 2 | Substrate scope of $S_NAr_{Ph}1.0$ 26

Supplementary Table 3 | Extinction coefficients of electrophiles and products 27

Supplementary Table 4 | Reverse-phase UPLC analysis methods 28

Supplementary Table 5 | Normal-phase chiral HPLC analysis methods 29

Supplementary Table 6 | Experimental and calculated masses of apo enzymes used to investigate the formation of a covalent aryl-enzyme intermediate. 30

Supplementary Table 7 | Experimental and calculated masses of apo enzymes used in this study 31

Supplementary Table 8 | Primer sequences used to generate DNA libraries 32

Supplementary Table 9 | Primer sequences used to generate point mutants of $S_NAr1.3$ 36

Supplementary Table 10 | Data collection and refinement statistics 37

Supplementary Table 11 | Crystallographic data for compound 3 (CCDC 2362363) 38

Chemical Synthesis 40

Protein and DNA sequences for $S_NAr1.3$ and $S_NAr_{Ph}1.0$ 74

NMR spectra of chemically synthesised substrates and product standards 75

Chiral HPLC chromatograms 111

References 134

Supplementary Figures

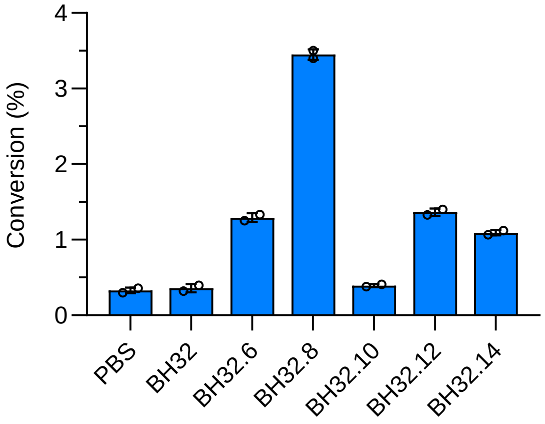

Supplementary Fig. 1 | Identification of a suitable starting point for evolution. Conversion to product **3** from ethyl 2-cyanopropionate (**1**) and 2,4-dinitrochlorobenzene (**2**) achieved using a selected number of BH32 evolutionary variants. Reaction conditions: **1** (25 mM), **2** (2.5 mM), BH32 variant (75 µM) in PBS pH 8.0 with 10% *v*/*v* DMSO at 30 °C for 16 h. Error bars represent the standard deviation of measurements made in duplicate. Source Data are provided as a Source Data file.

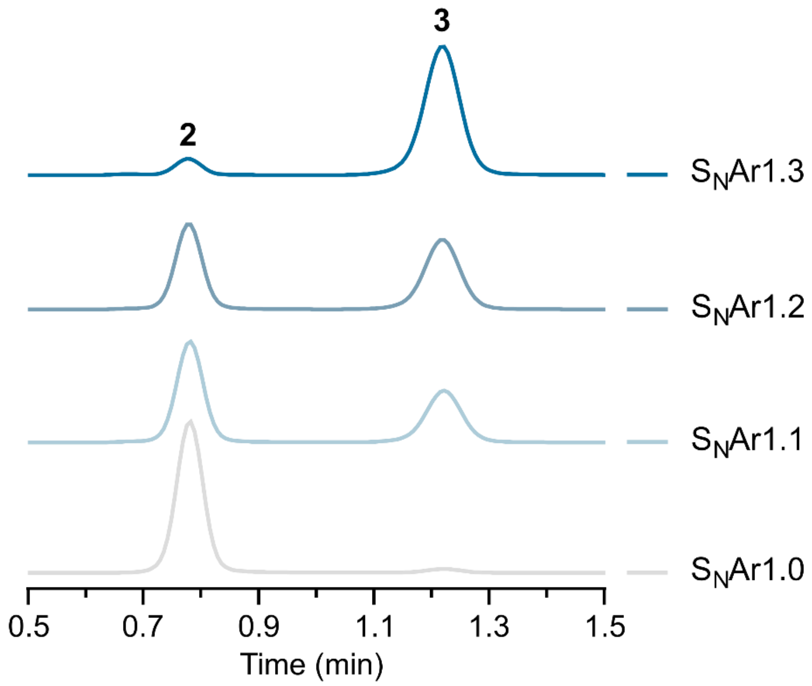

Supplementary Fig. 2 | UPLC chromatograms of the reaction profile across the evolutionary trajectory. Ultra-high performance chromatography (UPLC) absorbance chromatograms (260 nm) for the S_NAr reaction between ethyl 2-cyanopropionate (**1**) and 2,4-dinitrochlorobenzene (**2**) to give product **3**, using S_NAr enzymes from along the evolutionary trajectory. Reaction conditions: **1** (25 mM), **2** (2.5 mM), S_NAr variant (75 µM) in PBS pH 8.0 with 10% *v*/*v* DMSO as a co-solvent, 16 h at 30 °C.

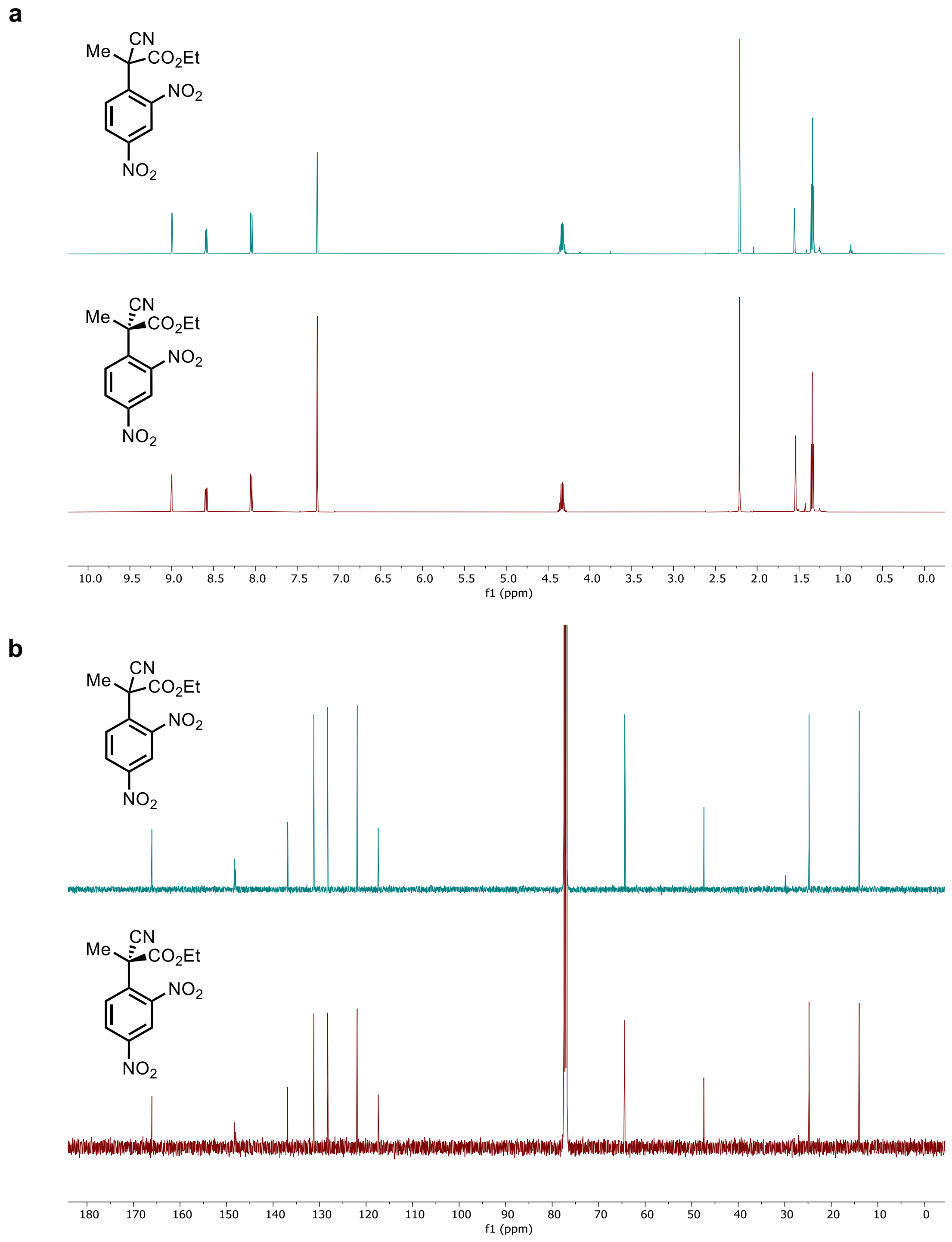

Supplementary Fig. 3 | NMR data of (*R*)-3 produced from a preparative-scale biotransformation using 2,4-dinitrochlorobenzene. **a**, Comparison of ^{1}H NMR data (500 MHz, $CDCl_3$) for the synthetic standard of **3** (top) and **(*R*)-3** (bottom) produced in the preparative-scale biotransformation using $S_NAr1.3$. **b**, Comparison of ^{13}C NMR data (126 MHz, $CDCl_3$) for the synthetic standard of **3** (top) and **(*R*)-3** (bottom) produced in the preparative-scale biotransformation using $S_NAr1.3$. Preparative-scale reaction conditions: **1** (25 mM), **2** (2.5 mM), $S_NAr1.3$ (50 µM) in PBS pH 8.0 with 10% *v*/*v* DMSO at 30 °C for 40 h.

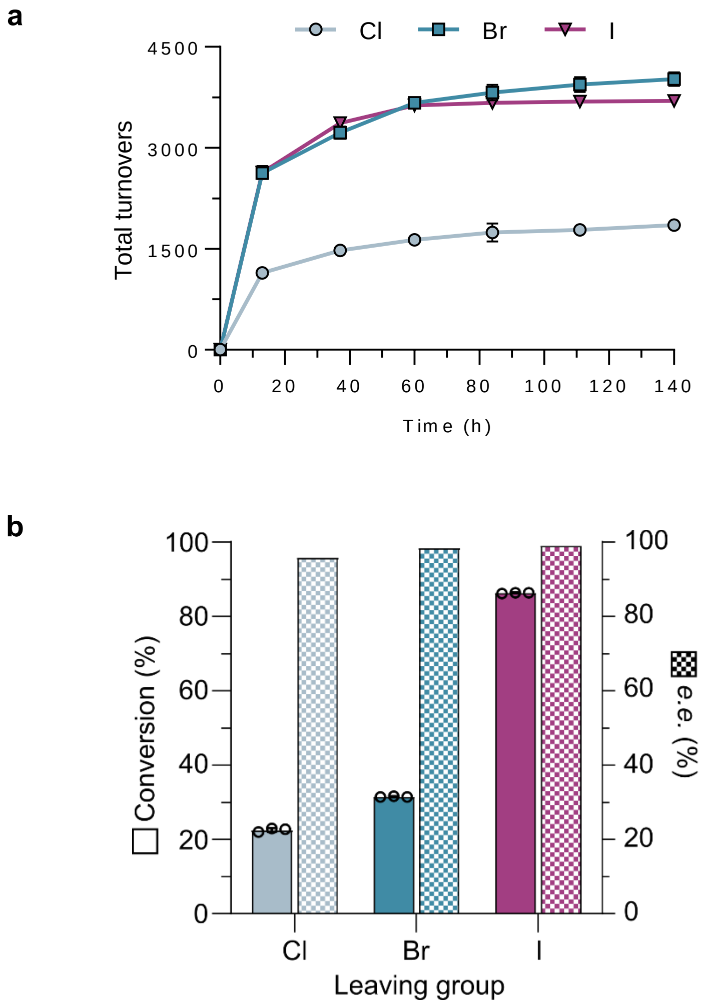

Supplementary Fig. 4 | The effect of halide leaving group on turnover, activity and selectivity for S$_N$Ar1.3. **a**, Time-course to the determine the total turnover number of S$_N$Ar1.3 using different 2,4-dinitrohalobenzene electrophiles. Reaction conditions: **1** (10 equiv), electrophile (**2**, 2.5 mM; **4**, 1.5 mM; **5**, 1.0 mM), S$_N$Ar1.3 (0.001 mol%) in NaP$_i$ pH 8.0 with 10% *v*/*v* DMSO and 0.1% *w*/*v* Pluronic F-127 at 30 °C. **b**, Bar chart showing the effect on reaction conversion (solid bars) and selectivity (patterned bars) achieved by S$_N$Ar1.3 with different 2,4-dinitrohalobenzene electrophiles. Reaction conditions: **1** (10 mM), 2,4-dinitrohalobenzene electrophile (1 mM), S$_N$Ar1.3 (5 μM) in NaP$_i$ pH 8.0 with 10% *v*/*v* DMSO at 30 °C for 16 h. Error bars represent the standard deviation of measurements made in triplicate. Source Data are provided as a Source Data file.

a

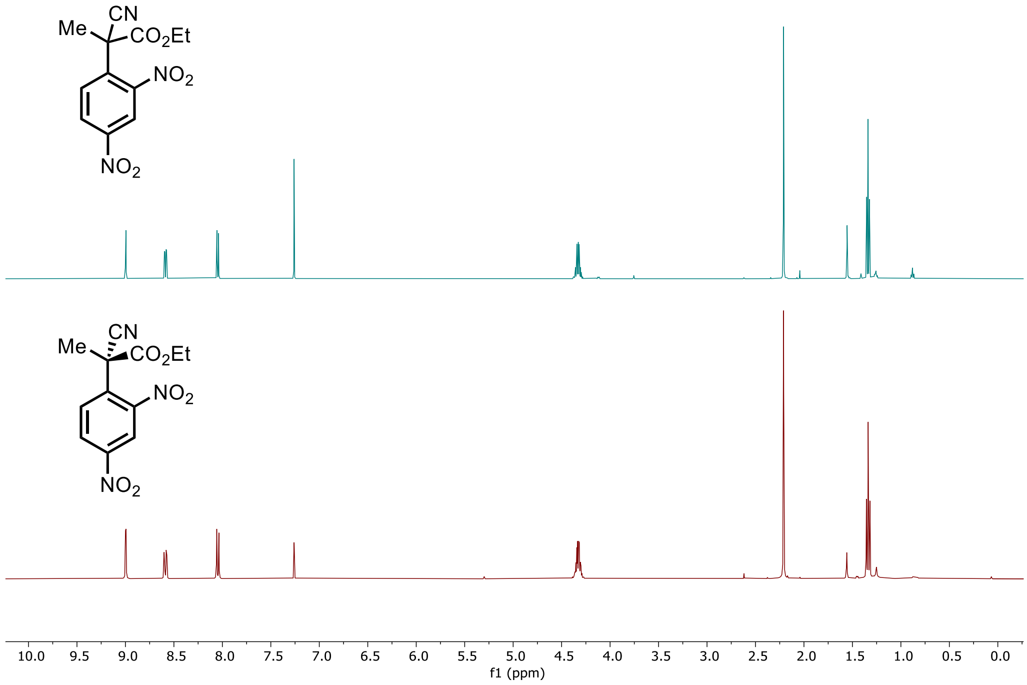

b

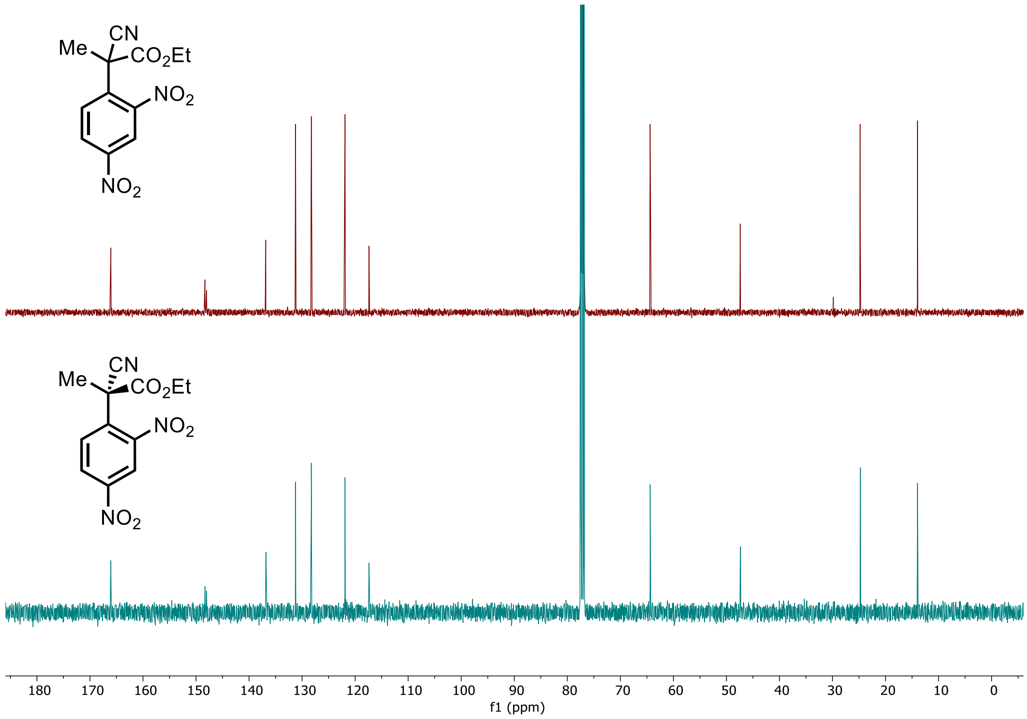

Supplementary Fig. 5 | NMR data of (*R*)-3 produced from a preparative-scale biotransformation using 2,4-dinitroiodobenzene. **a**, Comparison of ^{1}H NMR data (500 MHz, $CDCl_3$) for the synthetic standard of **3** (top) and **(*R*)-3** (bottom) produced in the preparative-scale biotransformation using S$_N$Ar1.3. **b**, Comparison of ^{13}C NMR data (126 MHz, $CDCl_3$) for the synthetic standard of **3** (top) and **(*R*)-3** (bottom) produced in the preparative-scale biotransformation using S$_N$Ar1.3. Preparative-scale reaction conditions: **1** (10 mM), **2** (1.0 mM), S$_N$Ar1.3 (50 µM) in NaP$_i$ pH 8.0 with 10% *v*/*v* DMSO at 30 °C for 20 h.

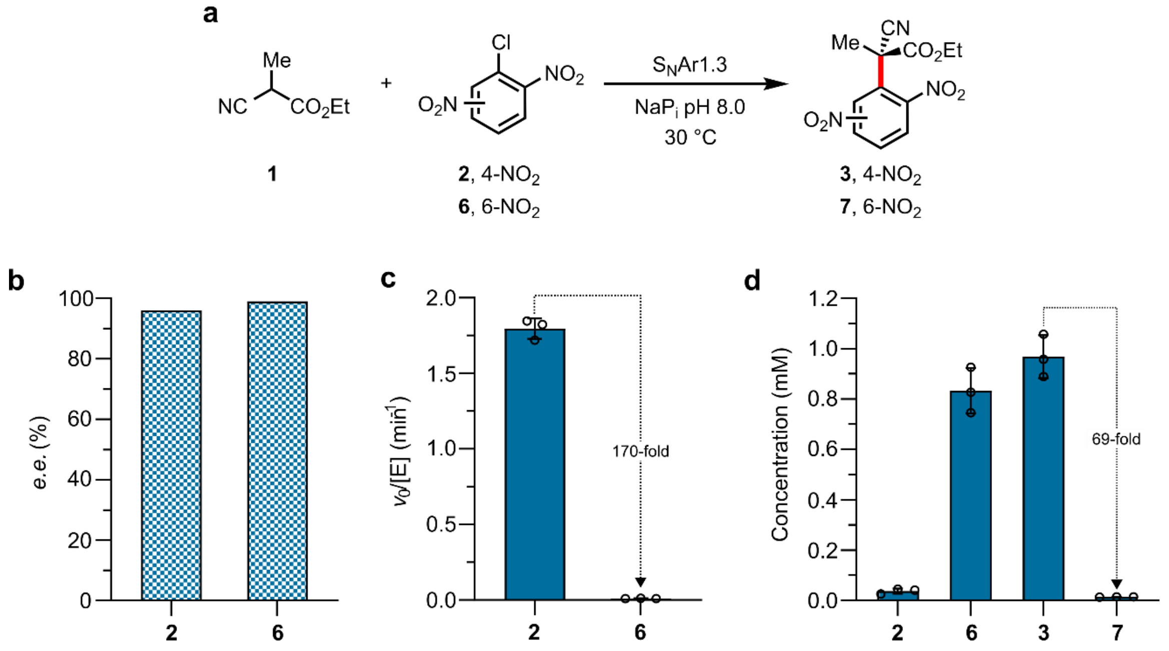

Supplementary Fig. 6 | SNAr1.3 selectivity and reaction rate with regioisomeric electrophile substrates 2 and 6. **a**, Chemical scheme showing the SNAr reaction between ethyl 2-cyanopropionate (**1**) and regioisomers of dinitrochlorobenzene (2,4-dinitrochlorobenzene (**2**) or 2,6-dinitrochlorobenzene (**6**)) to generate products **3** or **7**, respectively. **b**, Bar chart showing the stereoselectivity achieved with SNAr1.3 using **2** or **6** electrophiles. Reaction conditions: **1** (25 mM), **2** or **6** (2.5 mM), SNAr1.3 (75 µM for **2**; 125 µM for **6**) in NaP$_i$ pH 8.0 with 10% *v*/*v* DMSO at 30 °C for 20 h. **c**, Bar chart showing the rate achieved with SNAr1.3 using 2,4-dinitrochlorobenzene (**2**) or 2,6-dinitrochlorobenzene (**6**) electrophiles. **1** (25 mM), **2** or **6** (2.5 mM), SNAr1.3 (1 µM for **2**; 70 µM for **6**) in NaP$_i$ pH 8.0 with 10% *v*/*v* DMSO at 30 °C. **d**, Bar chart showing the concentrations of **2** and **6** and their respective products following incubation with SNAr1.3 and **1**. Reaction conditions: **1** (10 mM), **2** and **6,** (1.0 mM) and SNAr1.3 (50 µM) in NaP$_i$ pH 8.0 with 10% *v*/*v* DMSO at 30 °C for 3 h. Error bars represent the standard deviation of measurements made in triplicate. Source Data are provided as a Source Data file.

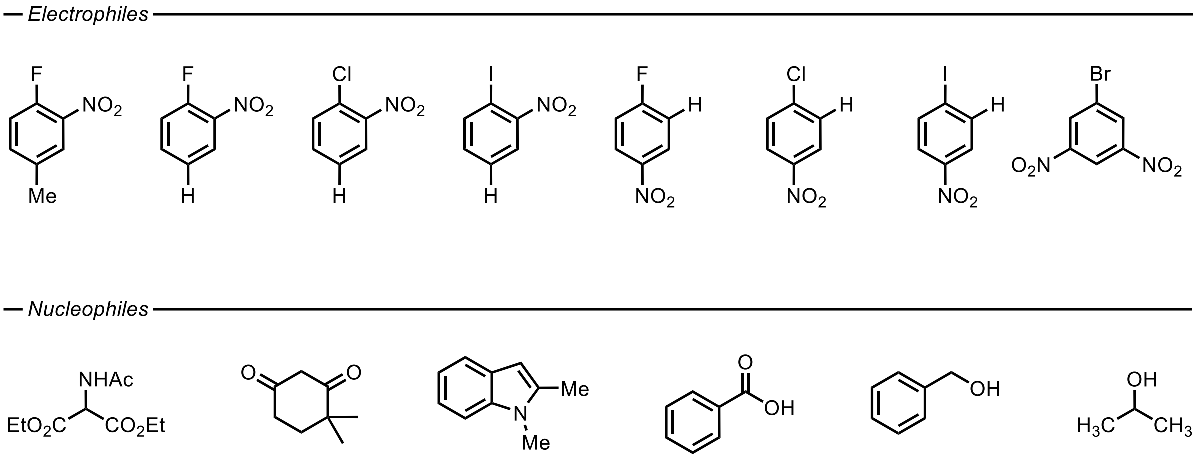

Supplementary Fig. 7 | Electrophiles and nucleophiles that are not tolerated as substrates by S$_N$Ar1.3. Top, Chemical structures of electrophile coupling partners that were incompatible with S$_N$Ar1.3 when employing ethyl 2-cyanopropionate (**1**) as the nucleophile. **Bottom,** Chemical structures of *C*- and *O*-nucleophile coupling partners that were incompatible with S$_N$Ar1.3 when employing 2,4-dinitrofluoro-, chloro-, bromo- or iodonitrobenzene as the electrophile.

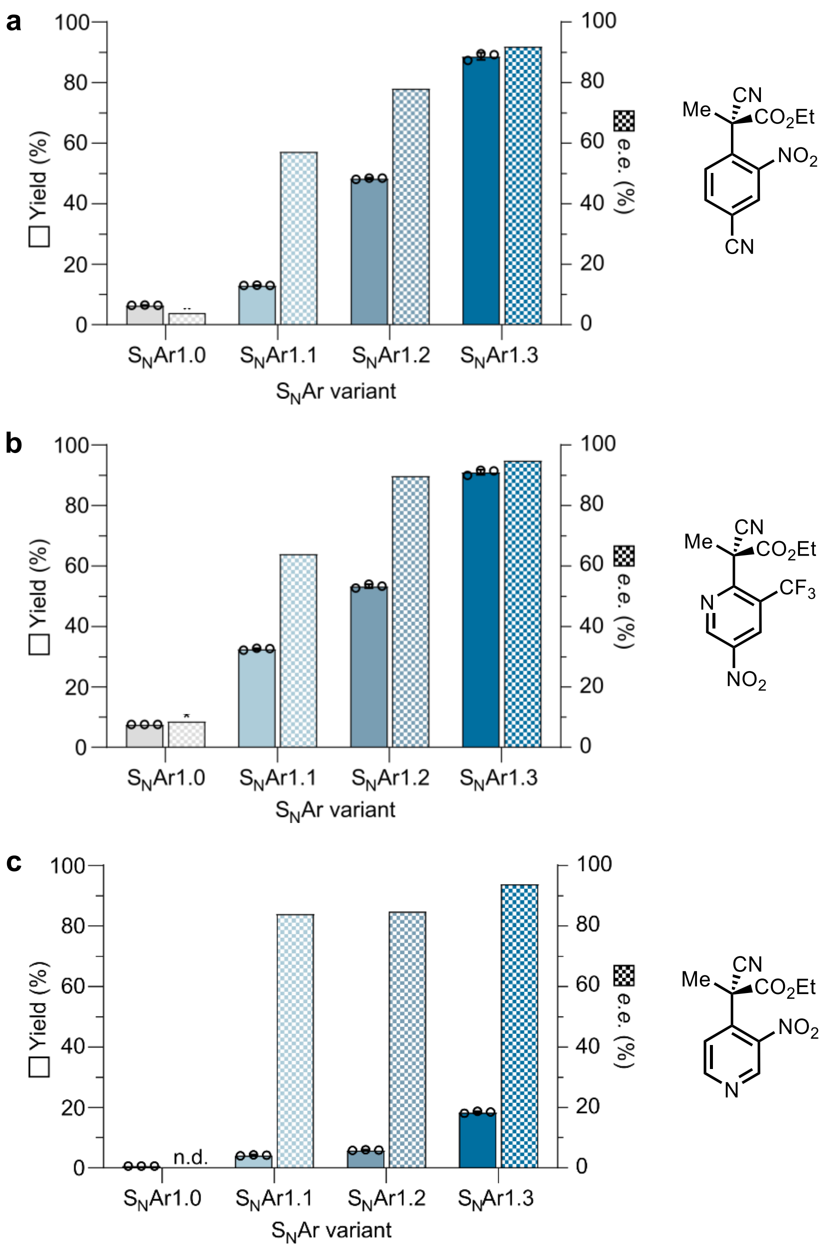

Supplementary Fig. 8 | Yield and selectivity for different electrophiles improves across evolution. Bar chart showing the yield (solid bars) and selectivity (patterned bars) for different electrophiles used in the substrate scope, that contain different halide leaving groups to that used during enzyme engineering, achieved with S_NAr enzymes from across the evolutionary trajectory. Reaction conditions: **a**, **1** (25 mM), 4-fluoro-3-nitrobenzonitrile (2.5 mM), S_NAr enzyme (75 µM) in NaP_i pH 8.0 with 10% *v*/*v* DMSO at 30 °C for 18 h. **b**, **1** (15 mM), 2-bromo-5-nitro-3-(trifluoromethyl)pyridine (1.5mM), S_NAr enzyme (50 µM) in NaP_i pH 8.0 with 10% *v*/*v* DMSO at 30 °C for 18 h. **c**, **1** (10 mM), 4-iodo-3-nitropyridine (1.0 mM), S_NAr enzyme (33 µM) in NaP_i pH 8.0 with 10% *v*/*v* DMSO at 30 °C for 18 h. ***N.b.*** The enantioselectivity for the reaction with 4-iodo-3-nitrobenzene using $S_NAr1.0$ could not be accurately determined due to low conversion. Error bars represent the standard deviation of measurements made in triplicate. Source Data are provided as a Source Data file.

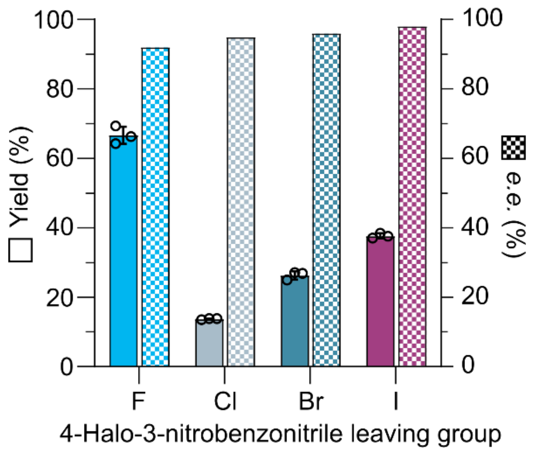

Supplementary Fig. 9 | The effect of leaving group on the activity and selectivity for the 4-halo-3-nitrobenzonitrile electrophile. Bar chart showing the yield (solid bar) and selectivity (patterned bar) achieved by $S_NAr1.3$ with different halide leaving groups on the 4-halo-3-nitrobenzonitrile electrophile normalized to 1.0 mM. Reaction conditions: **1** (10 mM), 4-halo-3-nitrobenzonitrile electrophile (1.0 mM), $S_NAr1.3$ (50 µM) in NaP_i pH 8.0 with 10% *v*/*v* DMSO at 30 °C for 16 h. Error bars represent the standard deviation of measurements made in triplicate. Source Data are provided as a Source Data file.

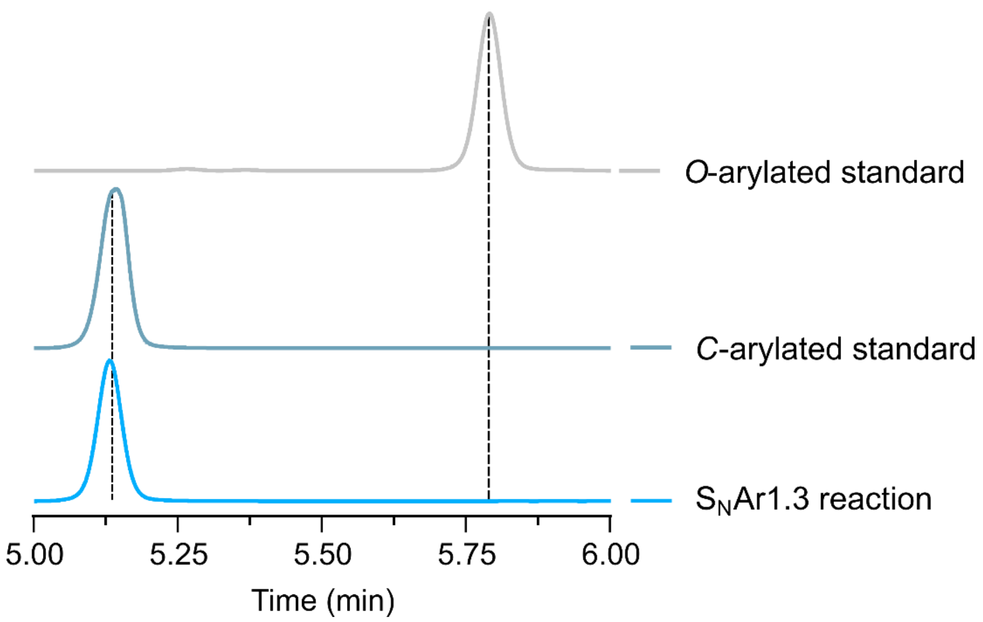

Supplementary Fig. 10 | S$_N$Ar1.3 does not suffer competing *C*- and *O*-arylation with β-ketoester nucleophiles. UPLC chromatograms (260 nm) comparing chemically synthesized standards of **24a** (*C*-arylation), **24b** (*O*-arylation) and the biotransformation using S$_N$Ar1.3 with 2,4-dinitrofluorobenzene and ethyl 2-oxocyclopentane-1-carboxylate, which shows there is exclusively *C*-arylation when using S$_N$Ar1.3. Reaction conditions can be found in Supplementary Table 1.

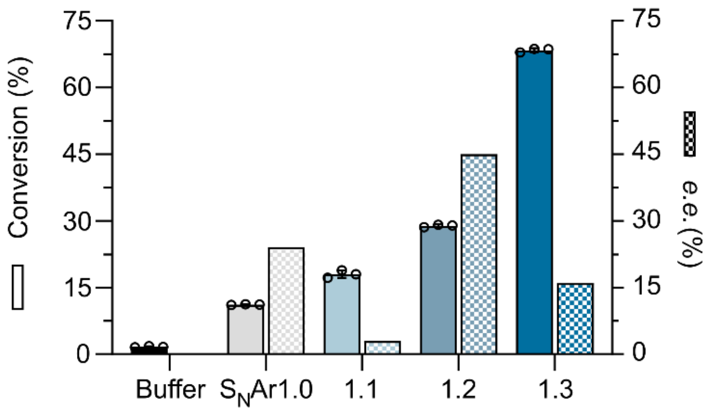

Supplementary Fig. 11 | Identification of a suitable starting point for evolving towards 1,1-diaryl products. Bar chart showing conversion (solid bars) and selectivity (patterned bars) to 1,1-diaryl product **30** achieved with S_NAr enzymes across the previous evolutionary trajectory using 2,4-dinitrochlorobenzene (**2**) and ethyl 2-cyano-2-phenylacetate (**29**). Reaction conditions: **29** (2 mM), **2**, (1 mM), S_NAr enzyme (50 µM) in PBS pH 6.0 with 20% *v*/*v* DMSO at 30 °C for 16 h. Error bars represent the standard deviation of measurements made in triplicate. ***N.b.*** Substrate concentration and co-solvent loading were adjusted due to reduced solubility of **29**; buffer pH was adjusted to decrease the level of background reactivity. Error bars represent the standard deviation of measurements made in triplicate. Source Data are provided as a Source Data file.

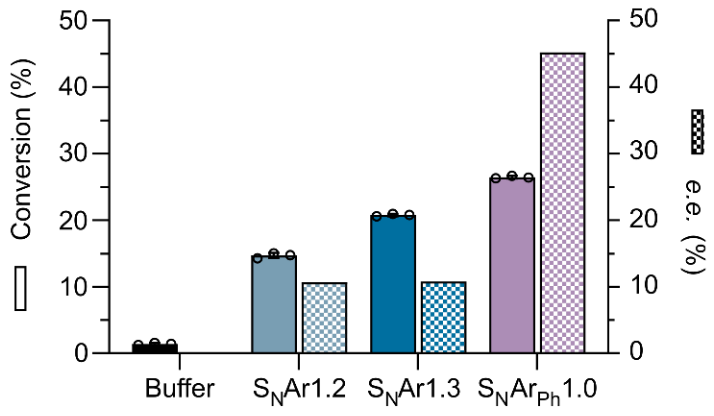

Supplementary Fig. 12 | $S_NAr_{Ph}1.0$ is more active and selective than other S_NAr variants for a different electrophile. The mutations installed from $S_NAr1.2$ to give $S_NAr_{Ph}1.0$ confer improved activity (solid bar) and selectivity (patterned bar) with a different substrate (4-fluoro-3-nitrobenzonitrile, affording product **31**) to that used during evolution. Reaction conditions: **29** (2 mM), 4-fluoro-3-nitrobenzonitrile (1 mM), S_NAr enzyme (50 μM) in PBS pH 7.0 with 20% *v*/*v* DMSO at 30 °C for 6 h. Error bars represent the standard deviation of measurements made in triplicate. Source Data are provided as a Source Data file.

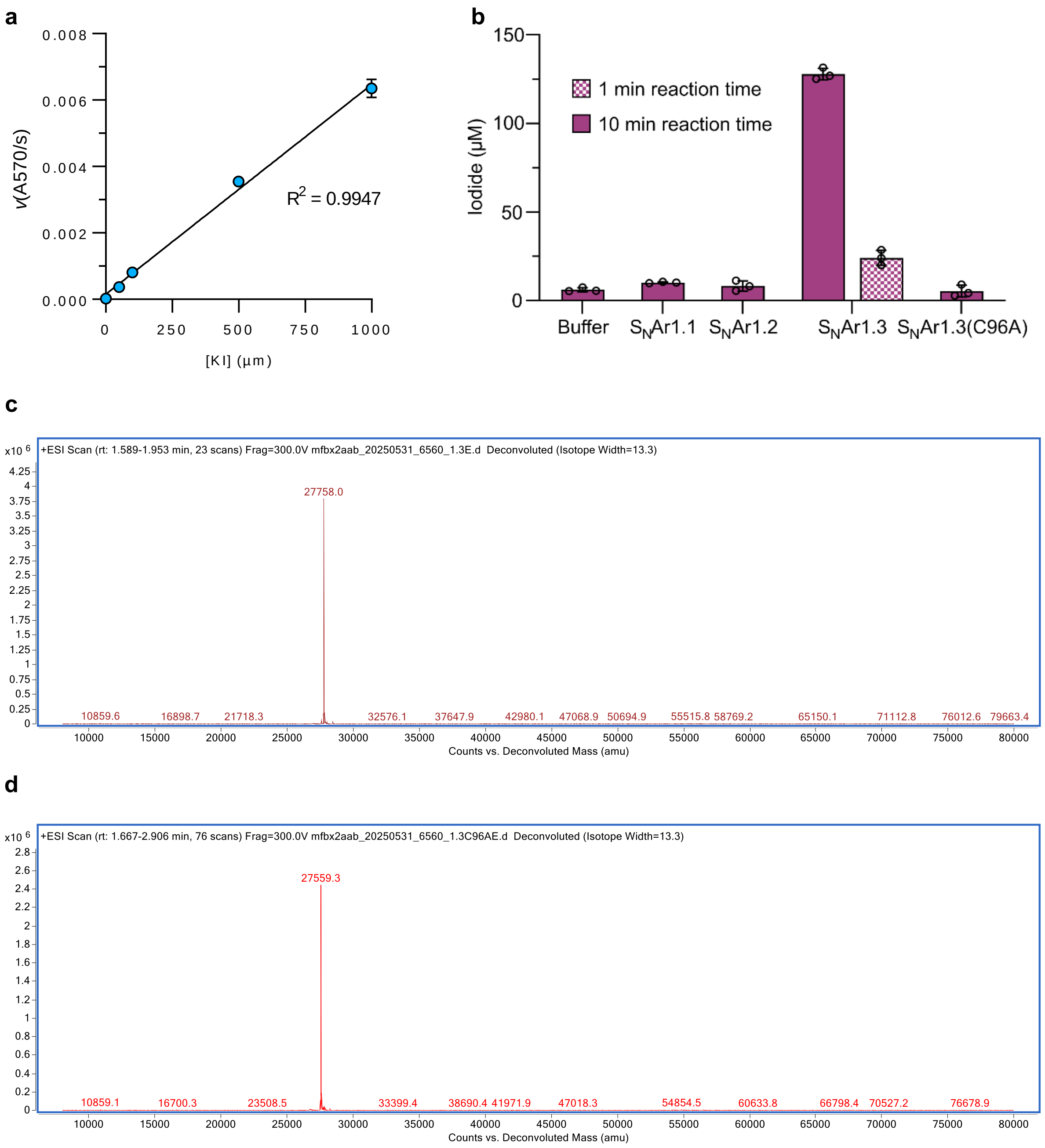

Supplementary Fig. 13 | Investigating the formation of an enzyme-substrate covalent intermediate using an iodide release assay. a, Calibration curve for the iodide release assay. The concentration of potassium iodide is plotted against the initial velocity of the increase in absorbance at 570 nm. **b**, Incubation of S$_N$Ar variants along the evolutionary trajectory with **5** prior to determination of iodide concentration using the iodide assay. As S$_N$Ar1.3 contained a single Cys residue introduced during evolution (Cys96), we suspected this residue underwent nucleophilic attack on **5**, leading to the release of iodide. Hence, S$_N$Ar1.3(C96A), a catalytically active and selective mutant (Extended Data Fig. 7, Supplementary Fig. 12 and 13), was assayed alongside. Timepoints of 1 and 10 minutes for S$_N$Ar1.3 indicate a slow arylation of Cys96. **c**, Protein mass spectrum acquired following incubation of S$_N$Ar1.3 with **5**, showing a single arylation (+167). **d**, Protein mass spectrum acquired following incubation of S$_N$Ar1.3(C96A) with **5**, showing no arylation. Protein mass spectrometry data for all variants can be found in Supplementary Table 6.

a

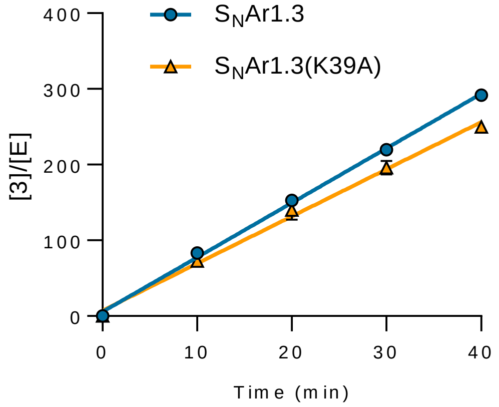

b

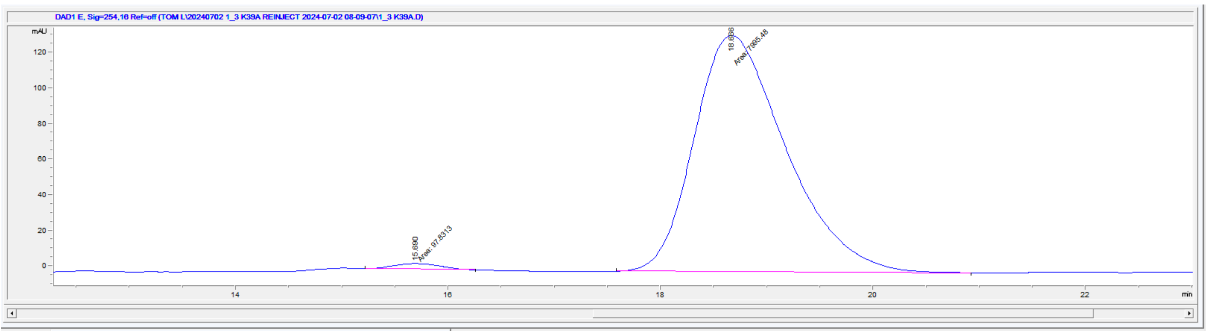

#	Time	Area	Height	Width	Area%	Symmetry
1	15.69	97.8	3.2	0.5051	1.209	0.804
2	18.666	7995.5	132.9	1.0029	98.791	0.667

Supplementary Fig. 14 | The K39A mutation installed to improve the resolution of diffraction data leads to minimal effects on rate and selectivity. a, Line graphs of [**3**]/[E] against time for point S_NAr1.3 and S_NAr1.3(K39A). Data were fitted using Linear Regression equation in GraphPad Prism. Reaction conditions: **1** (75 mM), **5** (1.0 mM) and S_NAr1.3 or S_NAr1.3(K39A) variant in NaPi pH 8.0 with 10% *v*/*v* DMSO at 30 °C. **b**, Chiral HPLC data from a reaction using S_NAr1.3(K39A). Reaction conditions: **1** (10 mM), **5** (1.0 mM) and S_NAr1.3(K39A) variant in NaPi pH 8.0 with 10% *v*/*v* DMSO at 30 °C for 14 h.

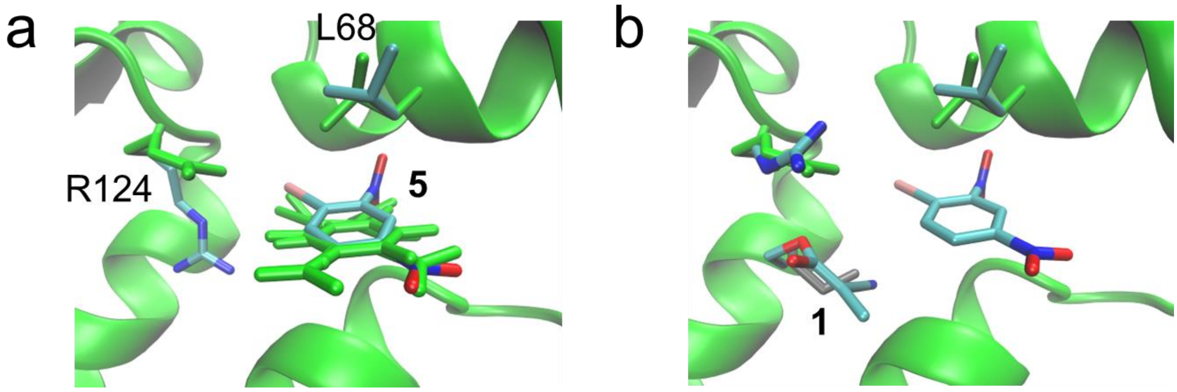

Supplementary Fig. 15 | Molecular docking of substrate 5 and nucleophile 1. a, Selected docking pose of **5** (blue sticks) with the corresponding conformations of Leu68 and Arg124 (blue sticks). The conformations of Leu68, Arg124 and the two orientations of **5** observed in the crystal structure (PDB: 9FUG, green backbone) are overlaid, depicted as green sticks. **b**, Selected pose from subsequent docking of **1** with the corresponding conformation of Arg124 (blue sticks), overlaid with the active site ethylene glycol (grey sticks) and conformations of Leu68 and Arg124 (green sticks) observed in the crystal structure (PDB: 9FUG).

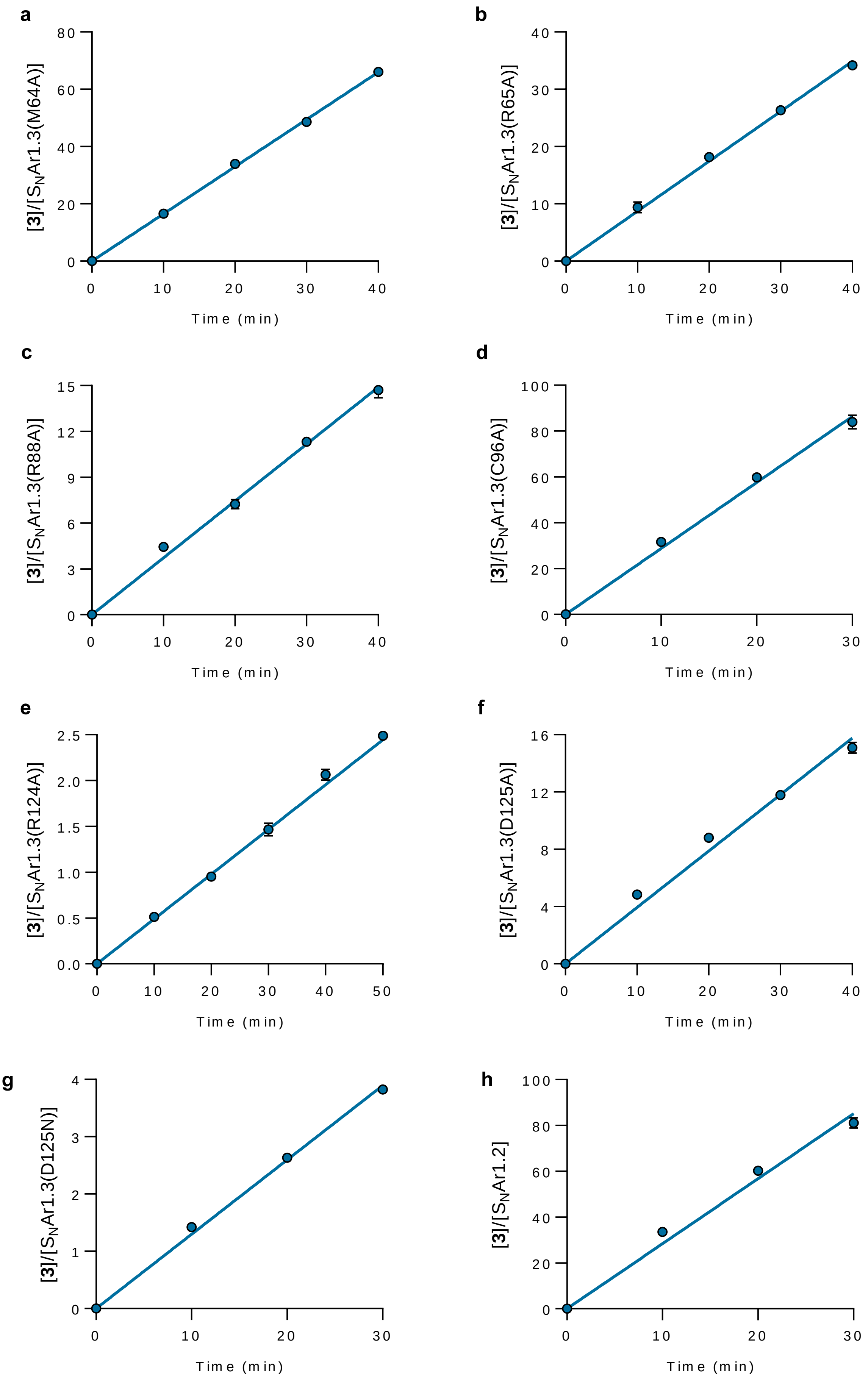
a
[3]/[SNAr1.3(M64A)]
Time (min)
b
[3]/[SNAr1.3(R65A)]
Time (min)
c
[3]/[SNAr1.3(R88A)]
Time (min)
d
[3]/[SNAr1.3(C96A)]
Time (min)
e
[3]/[SNAr1.3(R124A)]
Time (min)
f
[3]/[SNAr1.3(D125A)]
Time (min)
g
[3]/[SNAr1.3(D125N)]
Time (min)
h
[3]/[SNAr1.2]
Time (min)

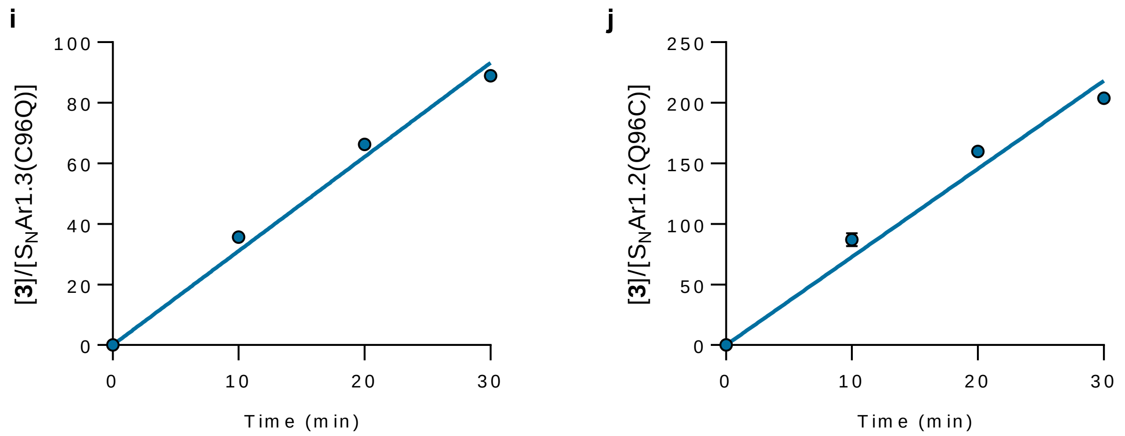

Supplementary Fig. 16 | The effect of SNAr1.3 point mutations on the reaction rate with substrate 5. Line graphs of [**3**]/[E] against time for point mutations of SNAr1.3 of residues that line the active site and halide binding cavity: **a**, SNAr1.3(M64A); **b**, SNAr1.3(R65A); **c**, SNAr1.3(R88A); **d**, SNAr1.3(C96A); **e**, SNAr1.3(R124A); **f**, SNAr1.3(D125A); **g**, SNAr1.3(D125N); **h**, SNAr1.2; **i**, SNAr1.3(C96Q); **j**, SNAr1.2(Q96C). Data were fitted using Linear Regression equation in GraphPad Prism. Reaction conditions: **1** (75 mM), **5** (1.0 mM) and SNAr1.3 variant in NaP_i pH 8.0 with 10% *v*/*v* DMSO at 30 °C. Enzyme concentrations: SNAr1.3 (1 µM); M64A, R65A, R88A, C96A (2 µM); SNAr1.2, SNAr1.3(C96Q), SNAr1.2(Q96C) (3 µM); R124A (25 µM); D125A (5 µM); D125N (40 µM). Error bars represent the standard deviation of measurements made in triplicate. Source Data are provided as a Source Data file.

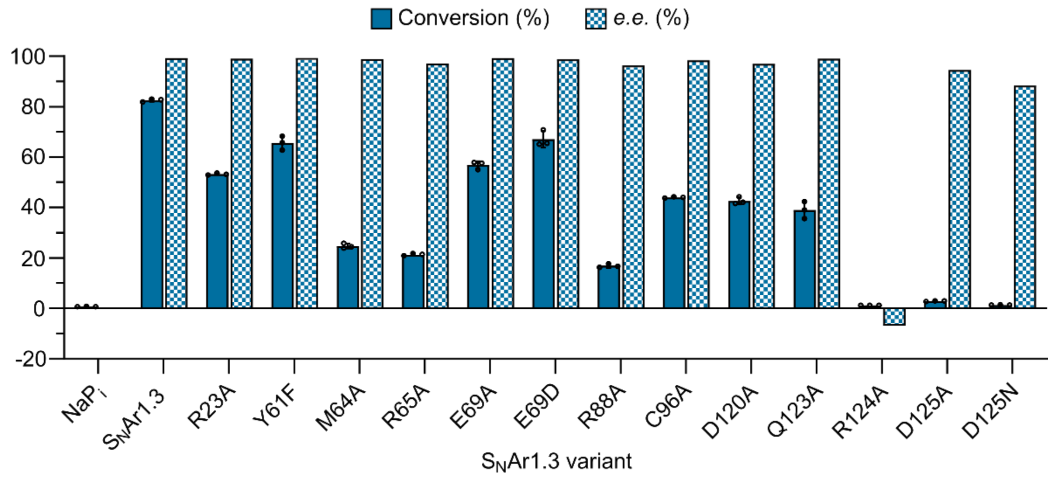

Supplementary Fig. 17 | The effect of mutating $S_NAr1.3$ active site residues on conversion and selectivity. Residues lining the halide binding site and secondary coordination sphere were mutated to understand their relative contributions to catalysis. The effects of mutating these residues on conversion (solid bars) and stereoselectivity (patterned bars) were measured using 2,4-dinitroiodobenzene (**5**) as the electrophile. Reaction conditions: **5** (1 mM), **1** (10 mM) and $S_NAr1.3$ variant (0.2 mol%) in NaP_i pH 8.0 with 10% *v*/*v* DMSO for 18 h at 30 °C. Error bars represent the standard deviation of measurements made in triplicate.

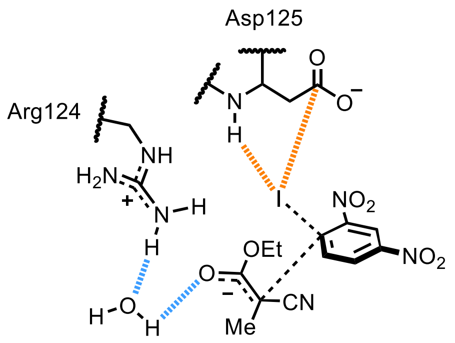

Supplementary Fig. 18 | Proposed substrate activation mode in S_NAr1.3. Schematic of the proposed substrate activation mode of S_NAr1.3 using **1** and **5** as substrates. H-bonding interactions involving Arg124, a water molecule and the enolate of **1** are shown in blue. Electrostatic interactions involving Asp125 and the halide leaving group are shown in orange.

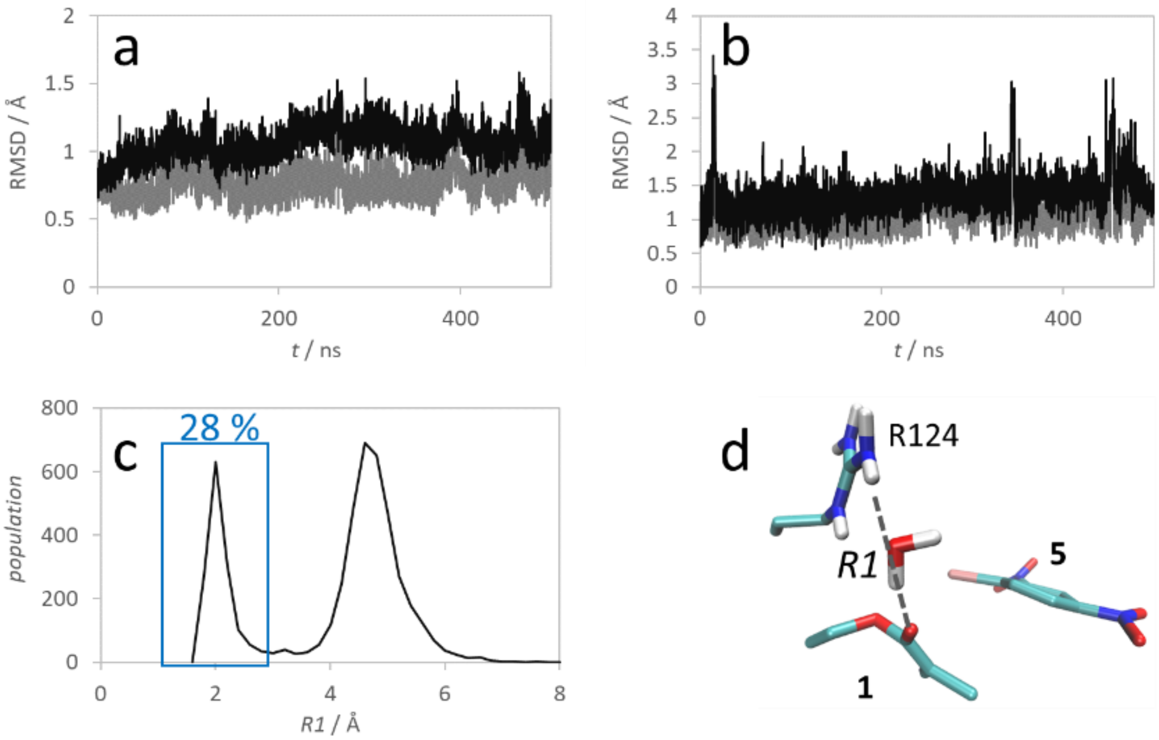

Supplementary Fig. 19 | Molecular dynamics simulations. **a**, Backbone RMSD and **b**, heavy atom RMSD for R124, substrate **5** and nucleophile **1** following least-squares fitting to the enzyme backbone; RMSDs were calculated relative to first structure of the 500 ns production run (black) and the average structure of the 500 ns production run (grey). The RMSDs relative to the average structure show that there is minimal structural drift and that the simulation is fluctuating about an average. **c**, distribution of O–H distance R1 shown in **d** which suggests that R124 is directly hydrogen-bonded to the enolate for 28% of the simulation (blue box), which agrees with hydrogen-bond analysis which identified a direct hydrogen bond in 26% of frames and a bridging water as in **d** in 48% of frames.

Supplementary Tables

Supplementary Table 1 | Substrate scope of S_NAr1.3. Reaction conditions for the synthesis of **3** and **7-28**. Electrophile substrates were chosen on the basis of commercial availability. Reactions were performed in NaP_i pH 8.0 with 10% *v*/*v* DMSO unless stated otherwise. Standard deviations are given for measurements in triplicate. Reaction conversions were determined based on UPLC calibration curves of authentic standards of starting materials and products. Extinction coefficients of electrophiles and products are given in Supplementary Table 3 and UPLC analysis methods are given in Supplementary Table 4. For products **7-28**, reactions were analysed on a 15 min gradient method (3 µL injection volume, 5-95% MeCN in MQ H_2O with 0.1% TFA). The *e.e.* values were determined using chiral HPLC analysis, chiral HPLC methods are given in Supplementary Table 5. Chromatograms of product standards and biotransformations are provided at the end of the Supplementary Information.

Product	Electrophile, conc. (mM)	Nucleophile, conc. (mM)	Catalyst loading (mol%)	Time (h)	Yield (%)	S.D.	*e.e.* (%)
**3**	**5,** 1.0	**1**, 10	0.5	20	>99	0	99
**7**	**6**, 2.5	**1**, 25	5	20	9	0.04	99
**8**	4-Fluoro-3-nitrobenzonitrile, 2.5	**1**, 25	5	20	>99	0	92
**8**	4-Chloro-3-nitrobenzonitrile, 2.5	**1**, 25	3	20	38	0.58	95
**8**	4-Bromo-3-nitrobenzonitrile, 1.5	**1**, 15	3	20	46	0.74	96
**8**	4-Iodo-3-nitrobenzonitrile, 1.0	**1**, 10	3	20	36	0.11	98
**9**	4-Iodo-3-nitropyridine (**SM1**), 1.0	**1**, 10	5	20	22	0.39	94
**10**	Methyl 4-fluoro-3-nitrobenzoate, 2.5	**1**, 25	5	20	27	0.15	78
**11**	1-(4-fluoro-3-nitrophenyl)ethan-1-one, 2.5	**1**, 50	5	20	43	0.15	78
**12**	2-Iodo-5-nitropyridine, 1.0	**1**, 10	3	20	6	0.19	94
**13**	1-Fluoro-2-nitro-4-(trifluoromethyl)benzene, 2.5	**1**, 25	5	20	32	1.54	87
**14**	Chloro-4-(methylsulfonyl)-2-nitrobenzene, 2.5	**1**, 25	5	20	4	0.16	69
**15**	2-Bromo-5-nitro-3-(trifluoromethyl)pyridine, 1.5	**1**, 15	3	20	98	0.25	94
**16**	4-Bromo-1-fluoro-2-nitrobenzene	**1**, 25	5	20	2	0.03	n.d.
**17** [a]	**5**, 1.0	*t*-Butyl 2-cyanopropanoate (**SM2**), 10	3	6	>99	0	>99
**18**	**5**, 1.0	*i*-Propyl 2-cyanopropanoate (**SM3**), 10	3	20	>99	0	99
**19**	**5**, 1.0	Benzyl 2-cyanopropanoate (**SM4**), 10	3	20	>99	0	97
**20**	5, 1.0	2-Cyano-*N*-ethylpropanamide (**SM5**), 20	5	20	55	0.76	99
**21**	**5**, 1.0	Ethyl 2-cyanopent-4-enoate (**SM6**), 10	5	20	>99	0	19

**22**	**5**, 1.0	Ethyl 2-cyano-3-methylbutanoate (**SM7**), 10	5	20	10	0.23	81
**23**	**5,** 1.0	Ethyl 2-nitropropanoate, 10	5	20	10	0.12	88
**24a**	2,4-Dinitrofluorobenzene, 2.5	Ethyl 2-oxocyclopentane-1-carboxylate, 25	5	1	>99	0	86
**25**	**2**, 2.5	1-(*tert*-butyl) 3-ethyl 2-oxopyrrolidine-1,3-dicarboxylate, 25	5	20	7	0.22	50
**26 †**	**2**, 2.5	Phenol, 25	5	20	91	0.66	–
**27 †**	**2**, 2.5	1,1,1,3,3,3-Hexafluoropropan-2-ol, 25	5	20	91	0.56	–
**28 †**	**2**, 2.5	2,2,2-Trifluoroethan-1-ol, 25	5	20	1	0.04	–

[a] Reaction run for shorter duration due to product decomposition under the reaction conditions. [b] Reaction run for shorter duration, with 5% *v*/*v* MeCN co-solvent and higher catalyst loading than is required due to product decomposition under the reaction conditions. n.d. = not determined. † No background reactivity was observed with 1,1,1,3,3,3-hexafluoropropan-2-ol or 2,2,2-trifluoroethan-1-ol nucleophiles and only low conversion (>3%) for the phenol nucleophile.

Supplementary Table 2 | Substrate scope of S_NAr_{Ph}1.0. Reaction conditions for the synthesis of **30**-**32**. Reactions were performed in PBS pH 6.0 with 20% *v*/*v* DMSO unless stated otherwise. Standard deviations are given for measurements in triplicate. Reaction conversions were determined based on UPLC calibration curves of authentic standards of starting materials and products. Extinction coefficients of electrophiles and products are given in Supplementary Table 3 and UPLC methods are given in Supplementary Table 4. For products **31** and **32**, reactions were analysed on a 15 min gradient method (3 µL injection volume, 5-95% MeCN in MQ H_2O with 0.1% TFA). The *e.e.* values were determined using chiral HPLC analysis, chiral HPLC methods are given in Supplementary Table 5. Chromatograms of product standards and biotransformations are provided at the end of the Supplementary Information.

Product	Electrophile, conc. (mM)	Nucleophile, conc. (mM)	Catalyst loading (mol%)	Time (h)	Yield (%)	S.D.	*e.e.* (%)
**30**	**5,** 1.0	**29**, 2.0	5	19	96	0.12	87
**31** [a]	4-Fluoro-3-nitrobenzonitrile, 1.0	**29**, 2.0	5	6	25	0.24	43
**32** [b]	2-Bromo-5-nitro-3-(trifluoromethyl)pyridine, 1.0	**29**, 2.0	5	19	50	0.07	29

[a] Reaction was performed in PBS pH 7.0 buffer

[b] Reaction was performed in PBS pH 8.0 buffer

Supplementary Table 3 | Extinction coefficients of electrophiles and products

Compound	Extinction coefficient (mM^{-1} cm^{-1}) [a]
**2**	1620
**3**	1450
**4**	1600
**5**	960
**6**	680
**7**	720
4-X-3-nitrobenzonitrile	810 (X = F); 610 (X = Cl); 800 (X = Br); 780 (X = I)
**8**	770
4-Iodo-3-nitropyridine **(SM1)**	770
**9**	650
Methyl 4-fluoro-3-nitrobenzoate	800
**10**	800
1-(4-Fluoro-3-nitrophenyl)ethan-1-one	1000
**11**	860
2-Iodo-5-nitropyridine	770
**12**	920
1-Fluoro-2-nitro-4-(trifluoromethyl)benzene	860
**13**	640
1-Chloro-4-(methylsulfonyl)-2-nitrobenzene	500
**14**	760
2-Bromo-5-nitro-3-(trifluoromethyl)pyridine	1550
**15**	900
4-Bromo-1-fluoro-2-nitrobenzene	840
**16**	670
**17**	1805
**18**	1820
**19**	1835
**20**	1720
**21**	1750
**22**	1780
**23**	1900
**24a**	1930
**25**	2140
**26**	1200
**27**	1460
**28**	1350
**30**	2030
**31**	840
**32**	800

[a] Detector wavelength was set at 260 nm

Supplementary Table 4 | Reverse-phase UPLC analysis methods

Product	Electrophile	Flow rate (mL min^{-1})	Mobile phase (% MeCN in MQ H_2O with 0.1% TFA)	Run time (min)
**3** [a]	**2**	1.2	35	2
	**4**	1.2	25	5
	**5**	1.2	25	5
**30** [b]	**2**, **4** or **5**	1	30 (0.1 min)<br>30-70% (2.25 min)<br>70-30% (2.3 min)<br>30% (2.5 min)	2.5

[a] Column temperature set to 20 °C

[b] Column temperature set to 30 °C

Supplementary Table 5 | Normal-phase chiral HPLC analysis methods

Product	Flow rate (mL min^{-1})	Mobile phase (% *i*PrOH in hexane)	Run time (min)
**3** [a]	1	20	25
**7** [a]	1	15	35
**8** [a]	1	10	35
**9** [a]	0.5	5	60
**10** [b]	1	10	25
**11** [a]	1	10	40
**12** [c]	1	5	25
**13** [b]	0.8	2	25
**14** [d]	1	20	45
**15** [b]	1	20	20
**16** [a]	1	10	15
**17** [b]	1	8	30
**19** [a]	1	10	50
**20** [a]	1	18	25
**21** [a]	1	20	15
**18** [a]	1	10	25
**22** [a]	0.7	5	35
**23** [a]	0.5	5	60
**24a** [a]	1	20	20
**25** [e]	1	3	10
**30** [a]	1	10	25
**31** [b]	1	10	30
**32** [b]	0.8	18	30

[a] Daicel CHIRALPAK® AS (25 cm, 0.46 cmØ)

[b] Daicel CHIRALPAK® OD-H (25 cm, 0.40 cmØ)

[c] Daicel CHIRALPAK® AD (25 cm, 0.46 cmØ)

[d] Daicel CHIRALPAK® IC-3 (5 cm, 0.21 cmØ)

[e] Daicel CHIRALPAK® IB-N (5 cm, 0.21 cmØ)

Supplementary Table 6 | Experimental and calculated masses of apo enzymes used to investigate the formation of a covalent aryl-enzyme intermediate.

Reaction	Expected mass (Da)	Observed mass (Da)
S_NAr1.0	27580	27579
S_NAr1.0 + **5**	27580	27579
S_NAr1.1	27579	27578
S_NAr1.1 + **5**	27579	27578
S_NAr1.2	27598	27597
S_NAr1.2 + **5**	27598	27597
S_NAr1.3	27591	27591
S_NAr1.3 + **5**	27591	27758 (+167)
S_NAr1.3(C96A)	27559	27559
S_NAr1.3(C96A) + **5**	27559	27559

Supplementary Table 7 | Experimental and calculated masses of apo enzymes used in this study

Variant	Expected Mass (Da)	Observed Mass (Da)
$S_NAr1.0$	27579.7	27579.0
$S_NAr1.1$	27579.7	27578.5
$S_NAr1.2$	27598.7	27597.3
$S_NAr1.3$	27591.7	27591.6
$S_NAr_{Ph}1.0$	27524	27524
$S_NAr1.3$(R23A)	27506.6	27506.8
$S_NAr1.3$(K39A)	27534.7	27534.5
$S_NAr1.3$(Y61F)	27575.7	27575.4
$S_NAr1.3$(M64A)	27531.6	27531.1
$S_NAr1.3$(R65A)	27506.6	27506.9
$S_NAr1.3$(E69A)	27553.7	27554.0
$S_NAr1.3$(E69D)	27577.7	27577.4
$S_NAr1.3$(R88A)	27506.6	27506.4
$S_NAr1.3$(C96A)	27559.7	27559.2
$S_NAr1.3$(D120A)	27547.7	27547.6
$S_NAr1.3$(Q123A)	27534.7	27534.9
$S_NAr1.3$(R124A)	27506.6	27506.5
$S_NAr1.3$(D125A)	27547.7	27546.8
$S_NAr1.3$(D125N)	27590.8	27590.2

Supplementary Table 8 | Primer sequences used to generate DNA libraries

**Flanking primers**	
Nde_F	catgcatgCATATGATTCGTGCGGTATTC
Xho_R	catgcatgCTCGAGAGAGCCCTGACC
**Round 1**	
L10x_F	catgcatgcatATGATTCGTGCGGTATTCTTTGATAGCNNKGGTACTCTGATTAG CGTTG
I14x_F	catgcatgcatATGATTCGTGCGGTATTCTTTGATAGCCTGGGTACTCTGNNKAG CGTTGAAGGCGCT
G18x_F	catgcatgcatATGATTCGTGCGGTATTCTTTGATAGCCTGGGTACTCTGATTAGCG TTGAANNKGCTTATAAAGTGCATCTGAAAATTATG
A19x_F	catgcatgcatATGATTCGTGCGGTATTCTTTGATAGCCTGGGTACTCTGATTAGC GTTGAAGGCNNKTATAAAGTGCATCTGAAAATTATGGAGG
Y20x_F	catgcatgcatATGATTCGTGCGGTATTCTTTGATAGCCTGGGTACTCTGATTAGC GTTGAAGGCGCTNNKAAAGTGCATCTGAAAATTATGGAG
V22x_F	GAAGGCGCTTATAAANNKCATCTGAAAATTATGGAGGAAGTG
V22x_R	TATAAGCGCCTTCAACGCT
H23x_F	GGCGCTTATAAAGTGNNKCTGAAAATTATGGAGGAAGTGCTG
H23x_R	CACTTTATAAGCGCCTTCAAC
I26x_F	AAAGTGCATCTGAAANNKATGGAGGAAGTGCTGG
I26x_R	AGATGCACTTTATAAGCGCC
Y45x_F	ACCCTGCTGGACGAANNKGAGAAACTGGCTCGC
Y45x_R	TTCGTCCAGCAGGGT
A49x_F	GAATACGAGAAACTGNNKCGCGAAGCGTTCTCT
A49x_R	CAGTTTCTCGTATTCGTCCAG
L64x_F	AAACCGTATCGTCCG NNKCGTGATATCCTGGAAGAAGTAAT
L64x_R	GGACGATACGGTTTGCC
R65x_F	CCGTATCGTCCGCTG NNK GAT ATC CTG GAA GAA GTA ATG CG
R65x_R	CAGCGGACGATACGG

L68x_F	CCGCTGCGTGATATCNNKGAAGAAGTAATGCGTAAACTGG
L68x_R	GATATCACGCAGCGG
E69x_F	CTGCGTGATATCCTGNNKGAAGTAATGCGTAAACTGGC
E69x_R	CAGGATATCACGCAGCG
M72x_F	GATATCCTGGAAGAAGTA NNK CGT AAA CTG GCG GAA AAG
M72x_R	TACTTCTTCCAGGATATCACGC
L87x_F	AAATACCCTGAAAAC NNK TGG GAA ATC TCC CTG C
L87x_R	GTTTTCAGGGTATTTGAAACCG
W88x_F	TACCCTGAAAACTTG NNK GAA ATC TCC CTG CGT ATG
W88x_R	CAAGTTTTCAGGGTATTTGAAACC
S91x_F	AACTTGTGGGAAATC NNK CTG CGT ATG GCG C
S91x_R	GATTTCCCACAAGTTTTCAGG
L92x_F	TTGTGGGAAATCTCC NNK CGT ATG GCG CAA CG
L92x_R	GGAGATTTCCCACAAGTTTTC
A95x_F	ATCTCCCTGCGTATG NNK CAA CGC TAC GGC GA
A95x_R	CATACGCAGGGAGATTTC
G99x_F	ATGGCGCAACGCTAC NNK GAG CTG TAC CCG GAA
G99x_R	GTAGCGTTGCGCCA
V120x_F	AAATATCACGTTGGC NNK ATC CTG AAT AGG GAT ACC GAG
V120x_R	GCCAACGTGATATTTACCTTTC
L122x_F	CACGTTGGCGTGATC NNK AAT AGG GAT ACC GAG CC
L122x_R	GATCACGCCAACGTGATA
N123x_F	GTTGGCGTGATCCTG NNK AGG GAT ACC GAG CCG
N123x_R	CAGGATCACGCCAAC
R124x_F	GGCGTGATCCTGAAT NNK GAT ACC GAG CCG GC
R124x_R	ATTCAGGATCACGCCAAC

E127x_F	CTGAATAGGGATACC NNK CCG GCC ACG GCAT
E127x_R	GGTATCCCTATTCAGGATCAC
P128x_F	AATAGGGATACCGAG NNK GCC ACG GCA TTC CT
P128x_R	CTCGGTATCCCTATTCAGGA
T130x_F	GATACCGAGCCGGCC NNK GCA TTC CTG GAC GCA
T130x_R	GGCCGGCTCGGTAT
A131x_F	ACCGAGCCGGCCACG NNK TTC CTG GAC GCA CTG
A131x_R	CGTGGCCGGCTC
K174x_F	GGCGTTAAAGGCGAG NNK GCA GTG TAC GTT GGT GAC
K174x_R	CTCGCCTTTAACGCCG
**Round 3**	
S9x_F	catgcatgcatATGATTCGTGCGGTATTCTTTGAT NNK CTGGGTACTCTGATTAGC
P63x_F	GGCAAACCGTATCGT NNK CTGCGTGATATCCTGGA
P63x_R	ACGATACGGTTTGCCC
L87x_F	AAATACCCTGAAAAC NNK CGGGAAATCTCCCTGC
L87x_R	GTTTTCAGGGTATTTGAAACCG
S91x_F	AACTTGCGGGAAATC NNK CTGCGTATGGCGC
S91x_R	GATTTCCCGCAAGTTTTCA
L92x_F	TTGCGGGAAATCTCC NNK CGTATGGCGCAACG
L92x_R	GGAGATTTCCCGCAAG
A95x_F	ATCTCCCTGCGTATG NNK CAACGCTACGGCG
A95x_R	CATACGCAGGGAGATTTC
Q96x_F	TCCCTGCGTATGGCG NNK CGCTACGGCGAG
Q96x_R	CGCCATACGCAGGG
L122x_F	CACGTTGGCGATATC NNK AGAGGGATACCGAGCC
L122x_R	GATATCGCCAACGTGATATTTACC

R124x_F	GGCGATATCCTGCAG NNK GATACCGAGCCGGC
R124x_R	CTGCAGGATATCGCCA
D125x_F	GATATCCTGCAGAGG NNK ACCGAGCCGGCC
D125x_R	CCTCTGCAGGATATCGC
P128x_F	CAGAGGGATACCGAG NNK GCCACGGCATTCCT
P128x_R	CTCGGTATCCCTCTGC
A129x_F	AGGGATACCGAGCCG NNK ACGGCATTCCTGGAC
A129x_R	CGGCTCGGTATCCC
F132x_F	GAGCCGGCCACGGCA NNK CTGGACGCACTGGG
F132x_R	TGCCGTGGCCG
L133x_F	CCGGCCACGGCATTC NNK GACGCACTGGGCAT
L133x_R	GAATGCCGTGGCCG

Supplementary Table 9 | Primer sequences used to generate point mutants of $S_NAr1.3$

Primers for point mutations	
R23A_F	GGCGCTTATAAAGTGGCTCTGAAAATTATGGAGGAAGTGCTG
Y45F_F	ACCCTGCTGGACGAATTTGAGAAACTGGCTCGC
Y61F_F	TATGCGGGCAAACCGTTTCGTCCGATGCGTGATAT
Y61F_R	CGGTTTGCCCGCATA
M64A_F	AAACCGTATCGTCCGGCGCGTGATATCCTGGAAGAA
R65A_F	CCGTATCGTCCGATGGCTGATATCCTGGAAGAAGTAATGCG
Y83F_F	AAGTACGGTTTCAAATTTCCTGAAAACTTGCGGGA
Y83R_F	TTTGAAACCGTACTTTTCCGC
R88A_F	TACCCTGAAAACTTGGCTGAAATCTCCCTGCGTATG
C96A_F	TCCCTGCGTATGGCGGCGCGCTACGGCGAG
D120A_F	AAATATCACGTTGGCGCTATCCTGCAGAGGGATAC
Q123A_F	GTTGGCGATATCCTGGCTAGGGATACCGAGCC
R124A_F	GGCGATATCCTGCAGGCTGATACCGAGCCGGC
D125A_F	GATATCCTGCAGAGGGCTACCGAGCCGGCC
D125E_F	GATATCCTGCAGAGGGAAACCGAGCCGGCC
D125N_F	GATATCCTGCAGAGGAACACCGAGCCGGCC

Supplementary Table 10 | Data collection and refinement statistics. Entries in parentheses refer to statistics in the highest resolution bin.

	$S_NAr1.3$ (K39A)	$S_NAr1.3$ iodide soak	$S_NAr1.3$ **5** soak (K39A)
PDB ascension code	9FUO	9FUL	9FUG
Wavelength (Å)	0.9763	0.9763	0.9763
Resolution range	54.16 – 1.81 (1.84 – 1.81)	61.06 – 1.88 (1.91 – 1.88)	54.29 – 1.71 (1.74 – 1.71)
Space group	P 31 2 1	P 31 2 1	P 31 2 1
Unit cell dimensions a, b, c, (Å) α, β, γ (°)	70.19, 70.19, 119.28 90, 90, 120	70.51, 70.51, 119.36 90, 90, 120	70.36, 70.36, 119.52 90, 90, 120
Total reflections	637040 (31369)	576370 (29523)	760006 (38079)
Unique reflections	31737 (1575)	28636 (1424)	37752 (1857)
Multiplicity	20.1 (19.9)	20.1 (20.7)	20.1 (20.5)
Completeness (%)	100.00 (100.00)	100.00 (99.9)	100.00 (100.00)
Mean I/sigma(I)	12.7 (0.3)	14.5 (0.4)	13.8 (0.3)
Wilson B-factor	30.4	35.0	29.5
R-meas	0.134 (4.680)	0.127 (5.150)	0.118 (5.435)
$CC_{1/2}$	1.0 (0.3)	1.0 (0.3)	1.0 (0.3)
R-work	0.194	0.207	0.204
R-free	0.231	0.230	0.243
Number of non-hydrogen atoms	2192	2008	2191
macromolecules	1914	1850	1956
ligands	31	18	34
solvent	247	140	201
Protein residues	231	226	231
RMS(bonds)	0.006	0.010	0.006
RMS(angles)	0.764	0.887	0.622
Ramachandran favoured (%)	97.38	97.30	98.69
Ramachandran allowed (%)	2.62	2.25	1.31
Ramachandran outliers (%)	0.00	0.45	0
Rotamer outliers (%)	1.46	0.51	2.84
Clashscore	6	5	3
Average B-factor	52.0	53.0	49.0

Supplementary Table 11 | Crystallographic data for compound 3 (CCDC 2362363)

Identification code	lil4
Empirical formula	$C_{12}H_{11}N_3O_6$
Formula weight	293.24
Temperature/K	99.9(4)
Crystal system	orthorhombic
Space group	$P2_12_12_1$
a/Å	8.0316(2)
b/Å	9.8338(2)
c/Å	16.3449(4)
α/°	90
β/°	90
γ/°	90
Volume/Å^3	1290.94(5)
Z	4
ρ_{calc}g/cm^3	1.509
μ/mm^{-1}	1.062
F(000)	608.0
Crystal size/mm^3	0.609 × 0.101 × 0.018
Radiation	CuKα (λ = 1.54184)
2Θ range for data collection/°	10.498 to 151.202
Index ranges	-8 ≤ h ≤ 9, -12 ≤ k ≤ 12, -19 ≤ l ≤ 20
Reflections collected	11687
Independent reflections	2605 [R_{int} = 0.0211, R_{sigma} = 0.0121]
Data/restraints/parameters	2605/0/192
Goodness-of-fit on F^2	1.087
Final R indexes [I>=2σ (I)]	R_1 = 0.0275, wR_2 = 0.0718
Final R indexes [all data]	R_1 = 0.0277, wR_2 = 0.0720
Largest diff. peak/hole / e Å^{-3}	0.14/-0.22
Flack parameter	0.04(8)

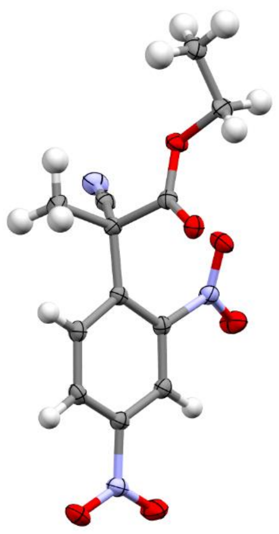

Data collection: Crystals suitable for diffraction were isolated by recrystallisation from EtOAc/hexane. Single crystal X-Ray diffraction data for compound **3** was collected at 100 K on a Rigaku XtaLab AFC-11 four circle goniometer equipped with a Hypix6000HE detector and Oxford cryosystem. Data was collected with a dual source Rigaku FR-X rotating anode using Cu – K_{α} ($\lambda = 1.54184$ Å) radiation. Data was collected using the CrysAlisPro program.

Crystal structure determination and refinements: Data processing and reduction was performed with CrysAlisPro. Empirical absorption correction was applied using spherical harmonics, implemented with the SCALE3 ABSPACK algorithm. The crystal structure was solved and refined using the SHELX suite of programmes in Olex2.[1,2] All non-hydrogen atoms were refined anisotropically. Hydrogen atom positions were calculated and refined with fixed isotropic displacement parameters. Absolute structure determination was based on anomalous dispersion. The absolute configuration of compound **3** was found to be (*R*).

Crystallographic data has been deposited with the CCDC number 2362363.

Chemical Synthesis

Preparation of starting materials

Synthesis of 4-iodo-3-nitropyridine (SM1)

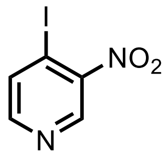

A 50 mL round-bottomed flask equipped with a stirrer bar was charged sequentially with 4-chloro-3-nitropyridine (500 mg, 3.15 mmol, 1.00 equiv) and NaI (4.80 g, 31.5 mmol, 10.0 equiv) and MeCN (16 mL), the resulting suspension sparged with N_2 (15 min) then heated to reflux for 2 days. The reaction mixture was cooled to room temperature, quenched with H_2O (50 mL), transferred into a separating funnel and extracted with EtOAc (3 × 50 mL). The combined organics were washed with brine, dried over $MgSO_4$ then concentrated *in vacuo*. The crude product was triturated with $CHCl_3$ (50 mL), centrifuged (2,900 *g*, 4 °C, 15 min) then the supernatant decanted and concentrated *in vacuo* to afford the title compound as an orange solid (72 mg, 9%) that was used in enzyme assays without further purification. Spectroscopic data matched those previously reported in the literature[3].

^{1}H NMR (500 MHz, $CDCl_3$)
δ 9.04 (s, 1H), 8.35 (d, *J* = 5.2 Hz, 1H), 8.02 (d, *J* = 5.1 Hz, 1H)

^{13}C{^{1}H} NMR (126 MHz, $CDCl_3$)
δ 152.55, 149.7, 146.0, 136.6, 98.5

Synthesis of *tert*-butyl 2-cyanopropanoate (SM2)

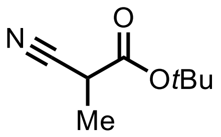

To a solution of *tert*-butyl 2-cyanoacetate (929 µL, 918 mg, 6.50 mmol, 1.30 equiv) and iodomethane (311 µL, 710 mg, 5.00 mmol, 1.00 equiv) in MeCN (25 mL) was added K_2CO_3 (691 mg, 5.00 mmol, 1.00 equiv), and the resulting mixture was stirred at 80 °C for 15 h. After filtration, the filtrate was concentrated and the residue purified by flash column chromatography (SiO_2, hexane/EtOAc: 16/1 to hexane/EtOAc: 8/1) to afford the title compound as a colourless oil (238 mg, 31%). Spectroscopic data matched those previously reported in the literature[4].

^{1}H NMR (400 MHz, $CDCl_3$)
δ 3.44 (q, *J* = 7.4 Hz, 1H), 1.54 (d, *J* = 7.4 Hz, 3H), 1.49 (s, 9H).

^{13}C{^{1}H} NMR (101 MHz, $CDCl_3$)
δ 165.6, 117.9, 84.1, 32.65, 27.9, 15.4.

Synthesis of *iso*-propyl 2-cyanopropanoate (SM3)

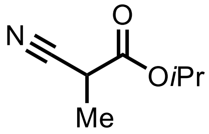

To a solution of *iso*-propyl 2-cyanoacetate (738 μL, 826 mg, 6.50 mmol, 1.30 equiv) and iodomethane (311 μL, 710 mg, 5.00 mmol, 1.00 equiv) in MeCN (25 mL) was added K_2CO_3 (691 mg, 5.00 mmol, 1.00 equiv), and the resulting mixture was stirred at 80 °C for 15 h. After filtration, the filtrate was concentrated and the residue purified by flash column chromatography (SiO_2, hexane/EtOAc: 16/1 to hexane/EtOAc: 8/1) to afford the title compound as a colourless oil (216 mg, 31%). Spectral data matched those previously reported in the literature[4].

^{1}H NMR (400 MHz, $CDCl_3$)
δ 5.14-5.01 (m, 1H), 3.50 (q, *J* = 7.4 Hz, 1H), 1.57 (d, *J* = 7.5 Hz, 3H), 1.30 (d, *J* = 6.3 Hz, 3H), 1.29 (d, *J* = 6.3 Hz, 3H)

^{13}C{^{1}H} NMR (101 MHz, $CDCl_3$)
δ 166.2, 117.6, 71.0, 31.9, 21.7, 21.6, 15.4

Synthesis of benzyl 2-cyanopropanoate (SM4)

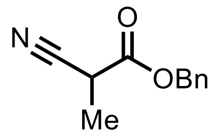

To a solution of benzyl cyanoacetate (995 µL, 1.14 g, 6.50 mmol, 1.30 equiv) and iodomethane (311 µL, 710 mg, 5.00 mmol, 1.00 equiv) in MeCN (25 mL) was added K_2CO_3 (691 mg, 5.00 mmol, 1.00 equiv.), and the resulting mixture was stirred at 80 °C for 15 h. After filtration, the filtrate was concentrated and the residue was purified by flash column chromatography (SiO_2, hexane/EtOAc: 16/1 to hexane/EtOAc: 8/1) to afford the title compound as a colourless oil (509 mg, 54%). Spectral data matched those previously reported in the literature[5].

^{1}H NMR (400 MHz, $CDCl_3$)
δ 7.44-7.32 (m, 5H), 5.24 (s, 2H), 3.59 (q, *J* = 7.4 Hz, 1H), 1.60 (d, *J* = 7.4 Hz, 3H)

^{13}C{^{1}H} NMR (101 MHz, $CDCl_3$)
δ 166.5, 134.65, 128.90, 128.86, 128.5, 117.3, 68.5, 31.7, 15.4

Synthesis of 2-cyano-*N*-ethylpropanamide (SM5)

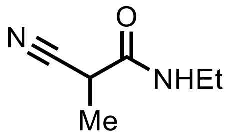

A 25 mL flask equipped with a stirrer bar was charged sequentially with ethyl 2-cyanopropionoate (629 μL, 635 mg, 5.00 mmol, 1.00 equiv) and ethylamine (66-72% aqueous solution, 5 mL) then sealed with a suba seal and the reaction mixture heated to 45 °C for 6 h. The reaction mixture was cooled to room temperature then the solvent removed *in vacuo.* The residue was redissolved in H_2O (10 mL) and extracted with EtOAc (3 × 15 mL), washed with brine, dried over $MgSO_4$ and then concentrated *in vacuo* to afford the title compound as a pale orange solid (488 mg, 77%). ^1H NMR data were consistent with data previously reported in the literature[6].

^1H NMR (500 MHz, $CDCl_3$)
δ 6.11 (s, 1H), 3.43 – 3.31 (m, 3H), 1.60 (d, *J* = 7.5 Hz, 3H), 1.19 (t, *J* = 7.3 Hz, 3H).

$^{13}C\{^1H\}$ NMR (126 MHz, $CDCl_3$)
δ 164.9, 119.3, 35.50, 32.4, 15.8, 14.7

HRMS (ESI$^+$)
Calcd for $C_6H_{10}ON_2Na$ ($[M+Na]^+$): 149.0685, found: 149.0682.

Synthesis of ethyl 2-cyanopent-4-enoate (SM6)

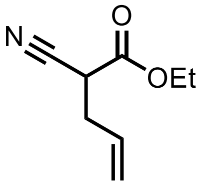

To a solution of ethyl cyanoacetate (1.60 mL, 1.70 g, 15.03 mmol, 3.00 equiv.) in anhydrous THF (25 mL) was added NaH (60% in paraffin oil, 200 mg, 5.00 mmol, 1.00 equiv) portion-wise at 0 °C. The reaction mixture was stirred at 0 °C for 30 min, then allyl bromide (435 μL, 608 mg, 5.00 mmol, 1.00 equiv) was added dropwise at 0 °C. After addition, the mixture was warmed up to room temperature and stirred for 20 h. The reaction was quenched with 1 M HCl (20 mL) and extracted with EtOAc (3 × 15 mL), and the combined organic phase was washed with brine, dried over $MgSO_4$, and concentrated. The residue purified by column chromatography (SiO_2, hexane/EtOAc: 16/1 to hexane/EtOAc: 8/1) to afford the title compound as a pale yellow oil (490 mg, 64%). Spectrosopic data matched those previously reported in the literature[7].

^{1}H NMR (400 MHz, $CDCl_3$)
δ 5.90-5.72 (m, 1H), 5.34-5.17 (m, 2H), 4.26 (q, *J* = 7.1 Hz, 2H), 3.56 (dd, *J* = 7.3, 6.3 Hz, 1H), 2.77-2.60 (m, 2H), 1.32 (t, *J* = 7.1 Hz, 3H)

^{13}C{^{1}H} NMR (101 MHz, $CDCl_3$)
δ 165.6, 131.5, 120.2, 116.2, 63.0, 37.6, 34.0, 14.1

Synthesis of ethyl 2-cyano-3-methylbutanoate (SM7)

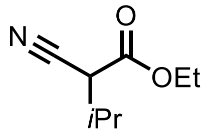

To a solution of ethyl cyanoacetate (534 μL, 566 mg, 5.00 mmol, 1.00 equiv) and 2-iodopropane (750 μL, 1.27 g, 7.50 mmol, 1.50 equiv) in acetone (25 mL) was added K_2CO_3 (2.07 g, 15.0 mmol, 3.00 equiv), and the resulting mixture was stirred at 70 °C for 24 h. After filtration, the filtrate was concentrated and the residue purified by flash column chromatography (SiO_2, hexane/EtOAc: 16/1 to hexane/EtOAc: 8/1) to afford the title product as a pale yellow oil (582 mg, 75%). Spectroscopic data matched those previously reported in the literature[8].

^{1}H NMR (400 MHz, $CDCl_3$)
δ 4.26 (q, *J* = 7.1 Hz, 2H), 3.39 (d, *J* = 5.3 Hz, 1H), 2.47-2.34 (m, 1H), 1.32 (t, *J* = 7.1 Hz, 3H), 1.13 (d, *J* = 6.8 Hz, 3H), 1.10 (d, *J* = 6.7 Hz, 3H)

^{13}C{^{1}H} NMR (101 MHz, $CDCl_3$)
δ 166.0, 115.6, 62.7, 45.45, 30.1, 20.8, 18.95, 14.2

Preparation of synthetic standards

General procedure 1

A round-bottomed flask equipped with a stirrer bar was charged sequentially with NaOEt (1.5 equiv), nucleophile (1.5 equiv), electrophile (1.0 equiv) and EtOH (0.1 M final concentration) then fitted with a reflux condenser and the solution heated to reflux for 18 h. The reaction mixture was cooled to room temperature, concentrated *in vacuo* and the residue taken up in H_2O/EtOAc (15 mL per 1.0 mmol each), transferred into a separating funnel, the organic layer removed then the aqueous extracted with EtOAc (2 × 15 mL per mmol). The combined organic layers were washed with brine, dried over $MgSO_4$ and concentrated *in vacuo*. The crude product was then purified by flash column chromatography.

General procedure 2

A heat gun-dried round-bottomed flask equipped with a stirrer bar was charged sequentially with Na_2CO_3 (2.50 equiv), nucleophile (if solid, 1.05 equiv) and electrophile (if solid, 1.00 equiv) then placed under an atmosphere of N_2. Anhydrous DMF (0.2 M final concentration), nucleophile (if liquid, 1.05 equiv) and electrophile (if liquid, 1.00 equiv) were added sequentially then the reaction mixture heated at 60 °C for the specific time period. The reaction mixture was cooled to room temperature, quenched with 1 M HCl (30 mL per mmol), transferred into a separating funnel and extracted with EtOAc (3 × 15 mL per mmol). The combined organics were washed with H_2O (3 × 15 mL per mmol), brine then dried over $MgSO_4$ and concentrated *in vacuo*. The crude product was then purified by flash column chromatography.

General Procedure 3

A heat gun-dried round-bottomed flask equipped with a stirrer bar was charged with NaH (60% in dispersion oil), flushed with N_2 and then charged with THF (50% of total volume). The stirring suspension was cooled to 0 °C then the requisite nucleophile in THF (30% of total volume) added dropwise. The suspension was stirred at rt for 30 min, cooled to 0 °C and the requisite electrophile in THF (20% of total volume) was added dropwise. The reaction mixture was warmed to rt and stirred for overnight. The reaction mixture was quenched dropwise with 1 M HCl (10 mL per mmol), transferred to a separating funnel and extracted with EtOAc (3 × 10 mL per mmol). The organic phases were combined, washed with brine, dried over $MgSO_4$ and concentrated *in vacuo*. The crude product was then purified by flash column chromatography.

Synthesis of ethyl 2-cyano-2-(2,4-dinitrophenyl)propanoate (3)

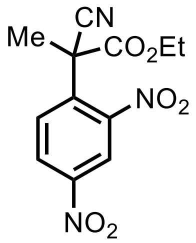

Following General Procedure 1, NaOEt (122 mg, 1.8 mmol, 1.8 equiv), 2,4-dinitrochlorobenzene (202 mg, 1.0 mmol, 1.0 equiv) and ethyl 2-cyanopropanoate (191 mg, 1.5 mmol, 1.5 equiv) were reacted in EtOH (10 mL). Following the work-up as described in General Procedure 1, the crude product was purified by flash column chromatography (SiO_2, Celite dry loading, 10-35% EtOAc in hexane) to afford the title compound as a yellow solid (152 mg, 52%).

R_f	0.28 (35% EtOAc in hexane) [UV]
Melting point	94-95 °C (hexane)
^{1}H NMR	(500 MHz, $CDCl_3$)<br>δ 9.00 (d, J = 2.4 Hz, 1H), 8.59 (dd, J = 8.6, 2.4 Hz, 1H), 8.05 (d, J = 8.6 Hz, 1H), 4.51 – 4.17 (m, 2H), 2.21 (s, 3H), 1.34 (t, J = 7.1 Hz, 3H)
^{13}C{^{1}H} NMR	(126 MHz, $CDCl_3$)<br>δ 166.1, 148.3, 148.0, 136.85, 131.2, 128.3, 121.9, 117.4, 64.4, 47.4, 24.8, 14.0
HRMS	(ESI$^+$)<br>Calcd. for $C_{12}H_{11}N_3O_3$ ([M+H]$^+$): 294.0721, found: 294.0718

Synthesis of ethyl 2-cyano-2-(2,6-dinitrophenyl)propanoate (7)

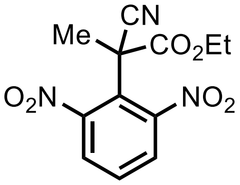

Following General Procedure 2, 1-chloro-2,6-dinitrobenzene (406 mg, 2.00 mmol, 1.00 equiv), (515 mg, 5.00 mmol, 2.50 equiv) and ethyl 2-cyanopropanoate (277 µL, 279 mg, 1.10 mmol, 1.10 equiv) were combined in DMF (10 mL) and stirred at 70 °C for 6 h. Following the work-up as described in General Procedure 2, the crude product was purified by flash column chromatography (SiO_2, 20-30% EtOAc in cyclohexane, Celite dry-loaded) to afford the title compound as a red oil (400 mg, 68%).

R_f	0.36 (30% EtOAc in cyclohexane) [UV]
^{1}H NMR	(400 MHz, $CDCl_3$)<br>δ 8.11 (d, *J* = 8.1 Hz, 2H), 7.83 – 7.69 (m, 1H), 4.39 – 4.15 (m, 2H), 2.40 (s, 3H), 1.30 (t, *J* = 7.1 Hz, 3H)
^{13}C{^{1}H} NMR	(101 MHz, $CDCl_3$)<br>δ 165.0, 151.35, 131.0, 129.5, 124.4, 116.3, 64.7, 46.4, 24.1, 13.7
HRMS	(ESI^+)<br>Calcd. for $C_{12}H_{11}O_6N_3Na$ ($[M+Na]^+$): 316.0531, found: 316.0540

Synthesis of ethyl 2-cyano-2-(4-cyano-2-nitrophenyl)propanoate (8)

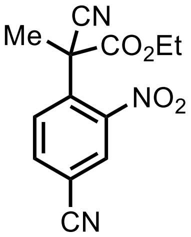

Following General Procedure 2, 4-fluoro-3-nitrobenzonitrile (166 mg, 1.00 mmol, 1.00 equiv), Na_2CO_3 (265 mg, 2.50 mmol, 2.50 equiv) and ethyl 2-cyanopropanoate (132 µL, 133 mg, 1.05 mmol, 1.05 equiv) were reacted in DMF (5 mL) for 1.5 h. Following the work-up as described in General Procedure 2, the crude product was purified by flash column chromatography (SiO_2, loaded in CH_2Cl_2, 20-30% EtOAc in hexane) to afford the title compound as a pale yellow solid (110 mg, 40%).

R_f	0.36 (40% EtOAc in hexane) [UV]
Melting point	93–94 °C (hexane)
^1H NMR	(500 MHz, $CDCl_3$)<br>δ 8.45 (d, *J* = 1.8 Hz, 1H), 8.04 (dd, *J* = 8.2, 1.8 Hz, 1H), 7.96 (d, *J* = 8.2 Hz, 1H), 4.32 (m, 2H), 2.18 (s, 3H), 1.33 (t, *J* = 7.1 Hz, 3H)
$^{13}C\{^1H\}$ NMR	(126 MHz, $CDCl_3$)<br>δ 166.15, 147.9, 137.2, 135.4, 130.8, 130.0, 117.4, 115.8, 115.1, 64.3, 47.4, 24.65, 14.0
HRMS	(ESI^+)<br>Calcd for $C_{13}H_{11}O_4N_3Na$ ($[M+Na]^+$): 296.0640, found: 296.0640

Synthesis of ethyl 2-cyano-2-(3-nitropyridin-4-yl)propanoate (9)

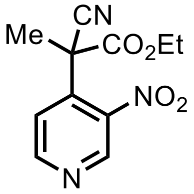

Following General Procedure 2, 4-chloro-3-nitropyridine (158 mg, 1.00 mmol, 1.00 equiv), Na_2CO_3 (265 mg, 2.50 mmol, 2.50 equiv) and ethyl 2-cyanopropanoate (132 µL, 133 mg, 1.05 mmol, 1.05 equiv) were reacted in DMF (5 mL) for 15 h. Following the work-up as described in General Procedure 2, the crude product was purified by flash column chromatography (SiO_2, loaded neat, 0-40% EtOAc in hexane) to afford the title compound as a pale orange solid (120 mg, 48%).

R_f	0.25 (40% EtOAc in hexane) [UV]
Melting point	64–5 °C (hexane)
^{1}H NMR	(500 MHz, $CDCl_3$)<br>δ 9.37 (s, 1H), 8.98 (d, J = 5.2 Hz, 1H), 7.73 (d, J = 5.2 Hz, 1H), 4.32 (m, 2H), 2.16 (s, 3H), 1.33 (t, J = 7.2 Hz, 3H)
^{13}C{^{1}H} NMR	(126 MHz, $CDCl_3$)<br>δ 165. 8, 155.3, 147.5, 143.2, 139.5, 123.3, 117.2, 64.3, 47.05, 24.1, 14.0
HRMS	(APCI$^+$)<br>Calcd. for $C_{11}H_{12}O_4N_3$ ([M+H]$^+$): 250.0822, found: 250.0815

Synthesis of methyl 4-(2-cyano-1-ethoxy-1-oxopropan-2-yl)-3-nitrobenzoate (10)

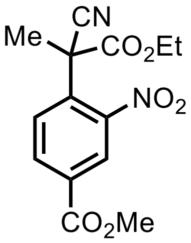

Following General Procedure 2, methyl 4-fluoro-3-nitrobenzoate (199 mg, 1.00 mmol, 1.00 equiv), Na_2CO_3 (265 mg, 2.50 mmol, 2.50 equiv) and ethyl 2-cyanopropanoate (132 µL, 133 mg, 1.05 mmol, 1.05 equiv) were reacted in DMF (5 mL) for 2 h. Following the work-up as described in General Procedure 2, the crude product was purified by flash column chromatography (SiO_2, loaded in CH_2Cl_2, 20% EtOAc in petrol) to afford the title compound as a pale yellow solid (172 mg, 56%).

R_f	0.21 (20% EtOAc in petrol) [UV]
Melting point	70–71 °C (hexane)
^{1}H NMR	(500 MHz, DMSO-d_6)<br>δ 8.60 (d, J = 1.9 Hz, 1H), 8.40 (dd, J = 8.3, 1.9 Hz, 1H), 8.11 (d, J = 8.3 Hz, 1H), 4.29 – 4.14 (m, 2H), 3.94 (s, 3H), 2.19 (s, 3H), 1.20 (t, J = 7.1 Hz, 3H)
^{13}C{^{1}H} NMR	(126 MHz, DMSO-d_6)<br>δ 167.0, 163.9, 147.40, 134.7, 133.2, 132.0, 130.7, 126.45, 117.9, 63.4, 53.0, 45.9, 23.9, 13.65
HRMS	(ESI+)<br>Calcd for $C_{14}H_{14}N_2O_6Na$ ($[M+Na]^+$): 329.0744, found: 329.0750

Synthesis of ethyl 2-(4-acetyl-2-nitrophenyl)-2-cyanopropanoate (11)

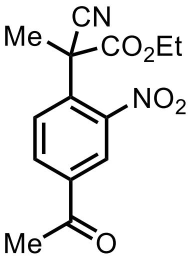

Following General Procedure 2, 1-(4-fluoro-3-nitrophenyl)ethan-1-one (183 mg, 1.00 mmol, 1.00 equiv), Na_2CO_3 (256 mg, 2.50 mmol, 2.50 equiv) and ethyl 2-cyanopropanoate (132 µL, 133 mg, 1.05 mmol, 1.05 equiv) were reacted in DMF (5 mL) for 2 h. Following the work-up as described in General Procedure 2, the crude product was purified by flash column chromatography (SiO_2, loaded in CH_2Cl_2, 30-40% EtOAc in hexane) to afford the title compound as a pale yellow solid (160 mg, 55%).

R_f	0.20 (30% EtOAc in hexane) [UV]
Melting point:	75–76 °C (hexane)
^{1}H NMR	(500 MHz, $CDCl_3$)<br>δ 8.69 (d, J = 1.9 Hz, 1H), 8.29 (dd, J = 8.2, 1.9 Hz, 1H), 7.92 (d, J = 8.2 Hz, 1H), 4.31 (m, 2H), 2.70 (s, 3H), 2.18 (s, 3H), 1.33 (t, J = 7.1 Hz, 3H)
$^{13}C\{^1H\}$ NMR	(126 MHz, $CDCl_3$)<br>δ 194.7, 166.45, 147.7, 138.5, 134.7, 133.1, 130.1, 126.1, 117.8, 63.9, 47.2, 26.7, 24.6, 13.9
HRMS	(ESI$^+$)<br>Calcd. for $C_{14}H_{14}O_5N_2Na$ ([M+Na]$^+$): 313.0795, found: 313.0792

Synthesis of ethyl 2-cyano-2-(5-nitropyridin-2-yl)propanoate (12)

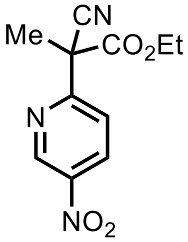

Following General Procedure 2, 2-chloro-5-nitropyridine (158 mg, 1.00 mmol, 1.00 equiv), Na_2CO_3 (265 mg, 2.50 mmol, 2.50 equiv) and ethyl 2-cyanopropanoate (132 µL, 133 mg, 1.05 mmol, 1.05 equiv) were reacted in DMF (5 mL) for 13 h. Following the work-up as described in General Procedure 2, the crude product was purified by flash column chromatography (SiO_2, loaded neat, 10-30% EtOAc in hexane) to afford the title compound as a yellow oil (130 mg, 53%).

R_f	0.56 (35% EtOAc in hexane) [UV]
^{1}H NMR	(500 MHz, $CDCl_3$)<br>δ 9.41 (dd, J = 2.6, 0.7 Hz, 1H), 8.59 (dd, J = 8.6, 2.6 Hz, 1H), 7.88 (dd, J = 8.6, 0.7 Hz, 1H), 4.36 – 4.22 (m, 2H), 2.04 (s, 3H), 1.27 (t, J = 7.1 Hz, 3H)
^{13}C{^{1}H} NMR	(126 MHz, $CDCl_3$)<br>δ 166.0, 160.8, 145.25, 144.0, 133.0, 121.75, 118.3, 64.1, 50.75, 23.8, 13.95
HRMS	(ESI$^+$)<br>Calcd. for $C_{11}H_{12}O_4N_3$ ([M+H]$^+$): 250.0822, found: 250.0830.

Synthesis of ethyl 2-cyano-2-(2-nitro-4-(trifluoromethyl)phenyl)propanoate (13)

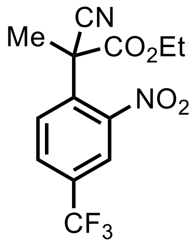

Following General Procedure 2, 1-fluoro-2-nitro-4-(trifluoromethyl)benzene (140 μL, 209 mg, 1.00 mmol, 1.00 equiv), Na_2CO_3 (265 mg, 2.50 mmol, 2.50 equiv) and ethyl 2-cyanopropanoate (132 μL, 133 mg, 1.05 mmol, 1.05 equiv) were reacted in DMF (5 mL) for 1 h. Following the work-up as described in General Procedure 2, the crude product was purified by flash column chromatography (SiO_2, loaded in CH_2Cl_2, 0-20% EtOAc in hexane) to afford the title compound as a yellow solid (150 mg, 47%)

R_f	0.24 (20% EtOAc in hexane) [UV]
Melting point	57 °C (hexane)
^{1}H NMR	(500 MHz, $CDCl_3$)<br>δ 8.44 (d, J = 1.9 Hz, 1H), 8.02 (dd, J = 8.4, 1.9 Hz, 1H), 7.97 (d, J = 8.3 Hz, 1H), 4.39 – 4.26 (m, 2H), 2.19 (s, 3H), 1.34 (t, J = 7.1 Hz, 3H)
^{13}C{^{1}H} NMR	(126 MHz, $CDCl_3$)<br>δ 166.3, 147.65, 134.35, 133.0 (q, J = 34.9 Hz), 130.7 (q, J = 3.5 Hz), 130.5, 123. 8 (q, J = 3.7 Hz), 122.3 (q, J = 273.1 Hz), 117.6, 64.0, 47.1, 24.6, 13.9
^{19}F NMR	(471 MHz, $CDCl_3$)<br>δ –63.2
HRMS	(ESI$^+$)<br>Calcd. for $C_{13}H_{12}O_4N_2F_3$ ([M+Na]$^+$): 317.0744, found: 317.0742.

Synthesis of ethyl 2-cyano-2-(4-(methylsulfonyl)-2-nitrophenyl)propanoate (14)

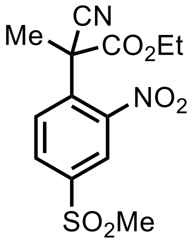

Following General Procedure 2, 1-chloro-4-(methylsulfonyl)-2-nitrobenzene (235 mg, 1.00 mmol, 1.00 equiv), Na_2CO_3 (265 mg, 2.50 mmol, 2.50 equiv) and ethyl 2-cyanopropanoate (132 μL, 133 mg, 1.05 mmol, 1.05 equiv) were reacted in DMF (5 mL) for 1.5 h. Following the work-up as described in General Procedure 2, the crude product was purified by flash column chromatography (SiO_2, loaded with CH_2Cl_2, 50% EtOAc in hexane) to afford the title compound as a pale yellow solid (72 mg, 22%).

R_f	0.3 (50% EtOAc in hexane) [UV]
Melting point	140–141 °C (hexane)
^{1}H NMR	(500 MHz, $CDCl_3$)<br>δ 8.70 (d, J = 2.0 Hz, 1H), 8.31 (dd, J = 8.3, 2.0 Hz, 1H), 8.04 (d, J = 8.2 Hz, 1H), 4.33 (m, 2H), 3.17 (s, 3H), 2.20 (s, 3H), 1.35 (t, J = 7.1 Hz, 3H)
^{13}C{^{1}H} NMR	(126 MHz, $CDCl_3$)<br>δ 166.2, 148.1, 143.15, 136.1, 132.6, 131.2, 125.8, 117.5, 64.3, 47.4, 44.5, 24.8, 14.0
HRMS	(ESI$^-$)<br>Calcd. for $C_{13}H_{14}N_2O_6S$ ([M–H]$^-$): 326.0578, found: 326.0530

Synthesis of ethyl 2-cyano-2-(5-nitro-3-(trifluoromethyl)pyridin-2-yl)propanoate (15)

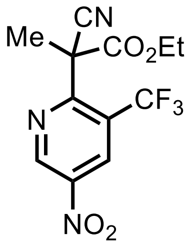

Following General Procedure 2, 2-bromo-5-nitro-3-(trifluoromethyl)pyridine (271 mg, 1.00 mmol, 1.00 equiv), Na_2CO_3 (265 mg, 2.50 mmol, 2.50 equiv) and ethyl 2-cyanopropanoate (132 μL, 133 mg, 1.05 mmol, 1.05 equiv) were reacted in DMF (5 mL) and for 15 h. Following the work-up as described in the General Procedure 2, the crude product was purified by flash column chromatography (Celite dry loading, SiO_2, 20-30% EtOAc in petrol) then by recrystallisation from EtOAc/hexane to afford the title compound as a pale yellow solid (82 mg, 26%).

R_f	0.21 (20% EtOAc in petrol) [UV]
Melting point	85–86 °C (hexane)
^{1}H NMR	(500 MHz, $CDCl_3$)<br>δ 9.58 (d, J = 2.5 Hz, 1H), 8.86 (d, J = 2.5 Hz, 1H), 4.38 – 4.27 (m, 2H), 2.21 (s, 3H), 1.32 (t, J = 7.1 Hz, 3H)
$^{13}C\{^1H\}$ NMR	(126 MHz, $CDCl_3$)<br>δ 166.9, 155.7, 146.2, 143.5, 132.5 (q, J = 5.1 Hz), 126.75 (q, J = 35.1 Hz), 122.1 (q, J = 274.9 Hz), 116.9, 64.1, 50.2, 24.6, 13.8
^{19}F NMR	(471 MHz, $CDCl_3$)<br>δ –57.0
HRMS	($APCI^+$)<br>Calcd. for $C_{12}H_{11}O_4N_3F_3$($[M+H]^+$): 318.0696, found: 318.0691

Synthesis of ethyl 2-(4-bromo-2-nitrophenyl)-2-cyanopropanoate (16)

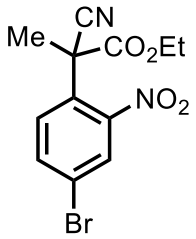

Following General Procedure 3, 4-bromo-1-fluoro-2-nitrobenzene (246 µL, 440 mg, 2.00 mmol, 1.00 equiv), ethyl 2-cyanopropanoate (553 µL, 559 mg, 4.40 mmol, 2.00 equiv) and NaH (60% in dispersion oil, 176 mg, 4.40 mmol, 2.20 equiv) were reacted in THF (10 mL) for 16 h. Following the work-up described in General Procedure 3, the crude product was purified by flash column chromatography (SiO_2, Celite dry-loaded, 20% EtOAc in hexane) to afford the title compound as an off-white solid (270 mg, 41%).

R_f	0.25 (20% EtOAc in hexane) [UV]
^{1}H NMR	(400 MHz, $CDCl_3$)<br>δ 8.31 (d, *J* = 2.1 Hz, 1H), 7.88 (dd, *J* = 8.5, 2.2 Hz, 1H), 7.65 (d, *J* = 8.5 Hz, 1H), 4.38 – 4.22 (m, 2H), 2.13 (s, 3H), 1.32 (t, *J* = 7.1 Hz, 3H)
^{13}C{^{1}H} NMR	(101 MHz, $CDCl_3$)<br>δ 166.75, 147.9, 137.3, 130.75, 129.8, 129.6, 124.0, 118.0, 63.9, 46.9, 24.6, 14.0
HRMS	(ESI$^+$)<br>Calcd. for $C_{12}H_{11}N_2O_4BrNa$ ([M+Na]$^+$): 348.9800, found: 348.9803

Synthesis of *tert*-butyl 2-cyano-2-(2,4-dinitrophenyl)propanoate (17)

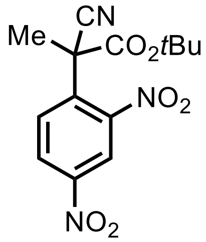

Following General Procedure 2, 2,4-dinitrofluorobenzene (126 µL, 186 mg, 1.00 mmol, 1.00 equiv), *tert*-butyl 2-cyanopropanoate (163 mg, 1.05 mmol, 1.05 equiv) and K_2CO_3 (346 mg, 2.50 mmol, 2.50 equiv) were reacted in DMF (5 mL) for 15 h. Following the work-up as described in General Procedure 2, the crude product was purified by flash column chromatography (SiO_2, hexane/EtOAc: 8/1 to hexane/EtOAc: 4/1) to afford the title compound as a yellow solid (269 mg, 84%).

R_f	0.20 (Hexane/EtOAc: 4/1) [UV]
Melting point	94-95 °C (EtOAc)
^{1}H NMR	(400 MHz, $CDCl_3$)<br>δ 8.96 (d, *J* = 2.4 Hz, 1H), 8.56 (dd, *J* = 8.7, 2.4 Hz, 1H), 8.01 (d, *J* = 8.7 Hz, 1H), 2.18 (s, 3H), 1.50 (s, 9H)
$^{13}C\{^1H\}$ NMR	(101 MHz, $CDCl_3$)<br>δ 164.7, 148.1, 148.1, 137.2, 131.2, 128.1, 121.7, 117.7, 86.4, 48.1, 27.6, 24.7
MS	(ESI$^-$)<br>*m*/*z* = 344 $[M + Na]^+$

Synthesis of *iso*-propyl 2-cyano-2-(2,4-dinitrophenyl)propanoate (18)

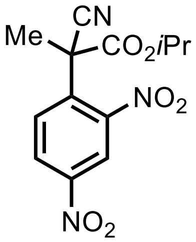

Following General Procedure 2, 2,4-dinitrofluorobenzene (126 µL, 186 mg, 1.00 mmol, 1.00 equiv) and *iso*-propyl 2-cyanopropanoate (148 mg, 1.05 mmol, 1.05 equiv) and K_2CO_3 (346 mg, 2.50 mmol, 2.50 equiv) were reacted in DMF (5 mL) for 15 h. Following the work-up as described in General Procedure 2, the crude product was purified by flash column chromatography (SiO_2, hexane/EtOAc: 8/1 to hexane/EtOAc: 4/1) to afford the title compound as a pale yellow solid (265 mg, 86%).

R_f 0.32 (Hexane/EtOAc: 4/1) [UV]

Melting point 75-76 °C (EtOAc)

^1H NMR (400 MHz, $CDCl_3$)
δ 8.98 (d, *J* = 2.4 Hz, 1H), 8.58 (dd, *J* = 8.7, 2.4 Hz, 1H), 8.03 (d, *J* = 8.7 Hz, 1H), 5.18-5.05 (m, 1H), 2.19 (s, 3H), 1.33 (d, *J* = 6.3 Hz, 3H), 1.30 (d, *J* = 6.3 Hz, 3H)

$^{13}C\{^1H\}$ NMR (101 MHz, $CDCl_3$)
δ 165.6, 148.2, 148.0, 136.9, 131.2, 128.2, 121.8, 117.4, 72.9, 47.4, 24.7, 21.7, 21.4

MS (ESI^-): *m*/*z* = 330 $[M + Na]^+$

Synthesis of benzyl 2-cyano-2-(2,4-dinitrophenyl)propanoate (19)

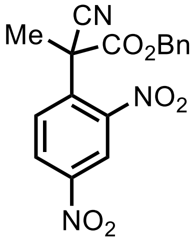

Following General Procedure 2, 2,4-dinitrofluorobenzene (126 µL, 186 mg, 1.00 mmol, 1.00 equiv), benzyl 2-cyanopropanoate (199 mg, 1.05 mmol, 1.05 equiv) and K_2CO_3 (346 mg, 2.50 mmol, 2.50 equiv) were reacted in DMF (5 mL) for 15 h. Following the work-up as described in General Procedure 2, the crude product was purified by flash column chromatography (SiO_2, hexane/EtOAc: 8/1 to hexane/EtOAc: 4/1) to afford the title compound as a yellow solid (108 mg, 30%).

R_f	0.18 (Hexane/EtOAc: 4/1) [UV]
Melting point	136-137 °C (EtOAc)
^{1}H NMR	(400 MHz, $CDCl_3$)<br>δ 8.99 (d, *J* = 2.4 Hz, 1H), 8.57 (dd, *J* = 8.7, 2.4 Hz, 1H), 8.02 (d, *J* = 8.7 Hz, 1H), 7.44-7.31 (m, 5H), 5.34 – 5.20 (m, 2H), 2.19 (s, 3H)
^{13}C{^{1}H} NMR	(101 MHz, $CDCl_3$)<br>δ 166.05, 148.3, 147.9, 136.6, 134.0, 131.2, 129.1, 128.9, 128.75, 128.3, 121.9, 117.1, 69.7, 47.3, 24.6
MS	(ESI$^-$)<br>*m*/*z* = 378 [M + Na]$^+$

Synthesis of 2-cyano-2-(2,4-dinitrophenyl)-*N*-ethylpropanamide (20)

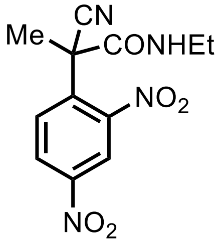

Following General Procedure 3, 1-fluoro-2,4-dinitrobenzene (302 µL, 446 mg, 2.40 mmol, 1.20 equiv), 2-cyano-*N*-ethylpropanamide (252 mg, 2.00 mmol, 1.00 equiv) and NaH (60% in dispersion oil, 100 mg, 2.50 mmol, 1.25 equiv) were reacted in THF (10 mL) for 16 h. Following the work-up described in General Procedure 3, the crude product was purified by flash column chromatography (SiO_2, Celite dry-loaded, 40% EtOAc in cyclohexane) to afford the title compound as an orange solid (380 mg, 65%)

R_f 0.28 (40% EtOAc in cyclohexane) [UV]

^{1}H NMR (400 MHz, $CDCl_3$)
δ 8.88 (d, *J* = 2.5 Hz, 1H), 8.54 (dd, *J* = 8.8, 2.5 Hz, 1H), 7.93 (d, *J* = 8.7 Hz, 1H), 6.46 (t, *J* = 5.7 Hz, 1H), 3.50 – 3.23 (m, 2H), 2.21 (s, 3H), 1.23 (t, *J* = 7.3 Hz, 3H)

^{13}C{^{1}H} NMR (101 MHz, $CDCl_3$)
δ 165.75, 148.8, 148.1, 136.5, 131.15, 127.7, 121.3, 118.4, 46.2, 36.1, 25.9, 14.5

HRMS (ESI$^+$)
Calcd. for $C_{12}H_{12}N_4O_5Na$ ([M+Na]$^+$): 315.0705, found: 315.0712.

Synthesis of ethyl 2-cyano-2-(2,4-dinitrophenyl)pent-4-enoate (21)

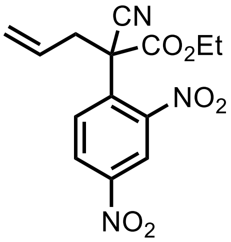

Following General Procedure 2, 2,4-dinitrofluorobenzene (126 μL, 186 mg, 1.00 mmol, 1.00 equiv.), ethyl 2-cyanopent-4-enoate (161 mg, 1.05 mmol, 1.05 equiv.) and K_2CO_3 (346 mg, 2.50 mmol, 2.50 equiv) were reacted in DMF (5 mL) for 15 h. Following the work-up as described in General Procedure 2, the crude product was purified by flash column chromatography (SiO_2, hexane/EtOAc: 8/1 to hexane/EtOAc: 4/1) to afford the title product as a yellow oil (200 mg, 63%).

R_f	0.58 (Hexane/EtOAc: 4/1) [UV]
^{1}H NMR	(400 MHz, $CDCl_3$)<br>δ 8.91 (d, J = 2.4 Hz, 1H), 8.54 (dd, J = 8.7, 2.4 Hz, 1H), 8.02 (d, J = 8.7 Hz, 1H), 5.82-5.63 (m, 1H), 5.29-5.14 (m, 2H), 4.30 (q, J = 7.1 Hz, 2H), 3.37 (dd, J = 14.4, 6.9 Hz, 1H), 3.21 (dd, J = 14.4, 7.7 Hz, 1H), 1.30 (t, J = 7.1 Hz, 3H).
^{13}C{^{1}H} NMR	(101 MHz, $CDCl_3$)<br>δ 164.7, 148.2, 148.1, 135.0, 132.8, 129.5, 127.6, 122.6, 121.7, 116.3, 64.3, 52.7, 40.7, 13.9
MS	(ESI$^-$)<br>m/z = 342 [M + Na]$^+$

Synthesis of ethyl 2-cyano-2-(2,4-dinitrophenyl)-3-methylbutanoate (22)

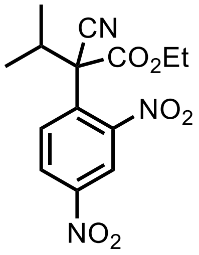

Following General Procedure 2, 2,4-dinitrofluorobenzene (126 µL, 186 mg, 1.00 mmol, 1.00 equiv), ethyl 2-cyano-3-methylbutanoate (163 mg, 1.05 mmol, 1.05 equiv) and K_2CO_3 (346 mg, 2.50 mmol, 2.50 equiv) were reacted in DMF (5 mL) for 15 h. Following the work-up as described in General Procedure 2, the crude product was purified by flash column chromatography (SiO_2, hexane/EtOAc: 8/1 to hexane/EtOAc: 4/1) to afford the title compound as a yellow solid (193 mg, 60%).

R_f	0.26 (Hexane/EtOAc: 4/1) [UV]
Melting point	95-96 °C (EtOAc)
^{1}H NMR	(400 MHz, $CDCl_3$)<br>δ 8.73 (d, *J* = 2.4 Hz, 1H), 8.53 (dd, *J* = 8.8, 2.5 Hz, 1H), 8.00 (d, *J* = 8.9 Hz, 1H), 4.43-4.23 (m, 2H), 3.11-2.94 (m, 1H), 1.34 (t, *J* = 7.1 Hz, 3H), 1.30 (d, *J* = 6.5 Hz, 3H), 1.08 (d, *J* = 6.7 Hz, 3H)
^{13}C{^{1}H} NMR	(101 MHz, $CDCl_3$)<br>δ 165.5, 149.8, 147.8, 134.6, 132.0, 127.0, 121.1, 114.8, 64.0, 57.15, 34.4, 19.2, 19.2, 14.0
MS	(ESI^-)<br>*m*/*z* = 344 $[M + Na]^+$

Synthesis of ethyl 2-(2,4-dinitrophenyl)-2-nitropropanoate (23)

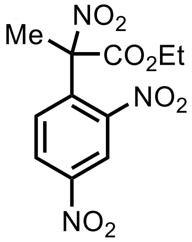

Following General Procedure 3, NaH (60% in paraffin oil, 80 mg, 2.00 mmol, 1.00 equiv), ethyl 2-nitropropanoate (260 µL, 294 mg, 2.00 mmol, 1.00 equiv) and 1-fluoro-2,4-dinitrobenzene (251 µL, 372 mg, 2.00 mmol, 1.00 equiv), were reacted in THF (10 mL) at rt for 20 h. Following the work-up as described in the General Procedure, the crude product was purified by column chromatography (SiO_2, 12-20% EtOAc in petrol, Celite dry-loading) to afford the title compound as a pale yellow oil (505 mg, 81%).

R_f	0.18 (20% EtOAc in petrol) [$KMnO_4$].
^{1}H NMR	(400 MHz, $CDCl_3$)<br>δ 8.93 (d, *J* = 2.4 Hz, 1H), 8.51 (dd, *J* = 8.7, 2.4 Hz, 1H), 7.58 (d, *J* = 8.7 Hz, 1H), 4.39 – 4.21 (m, 2H), 2.38 (s, 3H), 1.27 (t, *J* = 7.1 Hz, 3H).
^{13}C{^{1}H} NMR	(101 MHz, $CDCl_3$)<br>δ 163.9, 148.6, 148.2, 136.6, 130.35, 128.0, 121.7, 95.85, 64.4, 24.5, 13.7
HRMS	(APCI$^-$)<br>Calcd for $C_{11}H_{11}N_2O_6$ ([M–NO_2–H]$^-$): 267.0612, found: 267.0614.

Synthesis of ethyl 1-(2,4-dinitrophenyl)-2-oxocyclopentane-1-carboxylate (24a) and ethyl 2-(2,4-dinitrophenoxy)cyclopent-1-ene-1-carboxylate (24b).

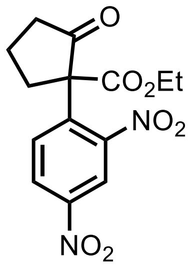

24a

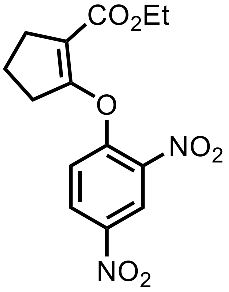

24b

Following General Procedure 3, NaH (60% in dispersion oil, 150 mg, 3.75 mmol, 1.25 equiv), ethyl 2-oxocyclopentane-1-carboxylate (450 μL, 468 mg, 3.00 mmol, 1.00 equiv) and 2,4-dinitrofluorobenzene (456 μL, 670 mg, 3.60 mmol, 1.20 equiv) were reacted at rt for 16 h. Following the work-up as described in the General Procedure, the crude product was purified by flash column chromatography (SiO_2, Celite dry loading, 20-30% EtOAc in hexane) to afford **24b** as an orange oil (136 mg, 14%) and **24a** which was purified further by recrystallisation from hexane/EtOAc to afford a pale yellow crystalline solid (190 mg, 20%). Spectroscopic data for both compounds matched that reported in the literature[9].

24a:

^{1}H NMR (500 MHz, $CDCl_3$)

δ 8.86 (d, *J* = 2.5 Hz, 1H), 8.41 (dd, *J* = 8.7, 2.5 Hz, 1H), 7.48 (d, *J* = 8.7 Hz, 1H), 4.26 – 4.13 (m, 2H), 3.26 – 3.17 (m, 1H), 2.79 – 2.68 (m, 1H), 2.57 – 2.46 (m, 1H), 2.45 – 2.35 (m, 1H), 2.37 – 2.24 (m, 1H), 2.08 – 1.96 (m, 1H), 1.21 (t, *J* = 7.1 Hz, 3H)

^{13}C{^{1}H} NMR (126 MHz, $CDCl_3$)

δ 211.15, 168.0, 149.4, 147.1, 140.5, 131.4, 127.2, 121.4, 65.4, 62.9, 38.9, 36.9, 19.6, 14.0

24b:

^{1}H NMR (500 MHz, $CDCl_3$)

δ 8.84 (d, *J* = 2.8 Hz, 1H), 8.39 (dd, *J* = 9.2, 2.7 Hz, 1H), 7.21 (d, *J* = 9.2 Hz, 1H), 4.08 – 4.01 (m, 2H), 2.80 – 2.72 (m, 2H), 2.72 – 2.65 (m, 2H), 2.11 – 2.01 (m, 2H), 1.08 (t, *J* = 7.1 Hz, 3H)

^{13}C{^{1}H} NMR (126 MHz, $CDCl_3$)

δ 163.0, 160.3, 154.5, 141.9, 139.1, 128.9, 122.25, 119.0, 118.3, 60.6, 33.0, 29.9, 19.0, 14.1

Synthesis of 1-(*tert*-butyl) 3-ethyl 3-(2,4-dinitrophenyl)-2-oxopyrrolidine-1,3-dicarboxylate (25)

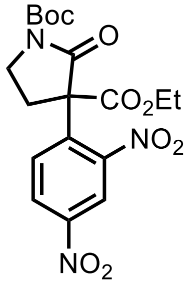

Following General Procedure 3, 1-(*tert*-butyl) 3-ethyl 2-oxopyrrolidine-1,3-dicarboxylate (565 mg, 2.20 mmol, 2.20 equiv), NaH (60% in mineral oil, 88 mg, 2.20 mmol, 2.20 equiv) and 1-fluoro-2,4-dinitrobenzene (126 µL, 186 mg, 1.00 mmol, 1.00 equiv) were reacted in THF (5 mL) and stirred at rt for 15 h. Following the work-up as described in the General Procedure, the crude product was purified by column chromatography (SiO_2, Celite dry-loading, 10-25% EtOAc in hexane) to afford the title compound as a pale yellow solid (134 mg, 32%). Spectroscopic data matched that previously reported in the literature[7].

^{1}H NMR (500 MHz, $CDCl_3$)
δ 8.89 (d, *J* = 2.5 Hz, 1H), 8.46 (dd, *J* = 8.7, 2.4 Hz, 1H), 7.71 (d, *J* = 8.7 Hz, 1H), 4.24 – 4.15 (m, 2H), 4.06 (ddd, *J* = 10.7, 8.4, 6.6 Hz, 1H), 3.73 (ddd, *J* = 10.6, 8.7, 4.9 Hz, 1H), 3.42 (ddd, *J* = 13.6, 8.3, 4.9 Hz, 1H), 2.26 (ddd, *J* = 13.9, 8.7, 6.5 Hz, 1H), 1.58 (s, 9H), 1.54 (s, 0H), 1.20 (t, *J* = 7.2 Hz, 3H)

$^{13}C\{^1H\}$ NMR (126 MHz, $CDCl_3$)
δ 168.8, 167.05, 149.4, 148.95, 147.2, 140.1, 131.8, 127.7, 121.2, 84.6, 63.5, 63.4, 44.0, 31.3, 28.0, 13.7

Synthesis of 2,4-dinitro-1-phenoxybenzene (26)

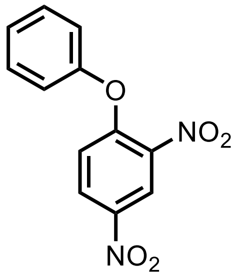

Following General Procedure 2**,** 2,4-dinitrochlorobenzene (202 mg, 1.00 mmol, 1.00 equiv), Na_2CO_3 (265 mg, 2.50 mmol, 2.50 equiv) and phenol (92 µL, 99 mg, 1.05 mmol, 1.05 equiv) were combined in DMF (5 mL) and stirred at 60 °C for 16 h. The work-up as described in the General Procedure, afforded the title compound as an orange solid (173 mg, 66%). Spectroscopic data matched that previously reported in the literature[10].

^{1}H NMR (400 MHz, $CDCl_3$)
δ 8.85 (d, *J* = 2.7 Hz, 1H), 8.31 (dd, *J* = 9.3, 2.8 Hz, 1H), 7.56 – 7.43 (m, 2H), 7.40 – 7.30 (m, 1H), 7.20 – 7.10 (m, 2H), 7.02 (d, *J* = 9.2 Hz, 1H).

^{13}C{^{1}H} NMR (101 MHz, $CDCl_3$)
δ 156.3, 153.6, 141.4, 139.5, 130.8, 128.9, 126.7, 122.2, 120.7, 118.5

Synthesis of 1-((1,1,1,3,3,3-hexafluoropropan-2-yl)oxy)-2,4-dinitrobenzene (27)

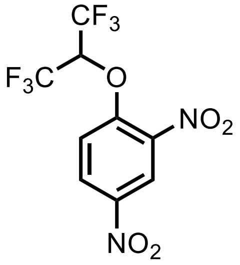

Following General Procedure 2, 2,4-dinitrochlorobenzene (202 mg, 1.00 mmol, 1.00 equiv), Na_2CO_3 (265 mg, 2.50 mmol, 2.50 equiv) and 1,1,1,3,3,3-hexafluoropropan-2-ol (111 µL, 176 mg, 1.05 mmol, 1.05 equiv) were combined in DMF (5 mL) and stirred at 60 °C for 15 h. Following the work-up as described in the General Procedure, the crude product was purified by column chromatography (SiO_2, 20% EtOAc in petrol, Celite dry-loading) to afford the title compound as a colourless solid (133 mg, 40%).

R_f	0.19 (20% EtOAc in petrol) [UV]
^{1}H NMR	(400 MHz, $CDCl_3$)<br>δ 8.83 (d, *J* = 2.7 Hz, 1H), 8.53 (dd, *J* = 9.2, 2.8 Hz, 1H), 7.37 (d, *J* = 9.2 Hz, 1H), 5.19 – 5.07 (m, 1H).
$^{13}C\{^1H\}$ NMR	(126 MHz, $CDCl_3$)<br>δ 153.4, 143.3, 140.6, 129.3, 122.4, 120.1 (q, J = 284.2 Hz), 116.9, 75.75 (hept, J = 34.9 Hz)
^{19}F NMR	(376 MHz, $CDCl_3$)<br>δ -72.7
HRMS	(ESI^+)<br>Calcd. for $C_9H_4F_6N_2O_5$ ($[M–H]^-$): 332.9946, found: 332.9942.

Synthesis of 2,4-dinitro-1-(2,2,2-trifluoroethoxy)benzene (28)

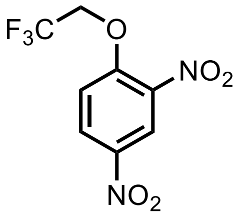

Following General Procedure 2, 2,4-dinitrochlorobenzene (202 mg, 1.00 mmol, 1.00 equiv), Na_2CO_3 (265 mg, 2.50 mmol, 2.50 equiv) and 2,2,2-trifluoroethan-1-ol (80 µL, 110 mg, 1.05 mmol, 1.05 equiv) were combined in DMF (5 mL) and stirred at 60 °C for 15 h. Following the work-up as described in the General Procedure, the crude product was purified by column chromatography (SiO_2, 10-30% EtOAc in petrol, Celite dry-loading) to afford the title compound as a colourless solid (71 mg, 27%)

R_f	0.4 (30% EtOAc in petrol) [UV]
^1H NMR	(400 MHz, $CDCl_3$)<br>δ 8.77 (d, *J* = 2.7 Hz, 1H), 8.49 (dd, *J* = 9.2, 2.8 Hz, 1H), 7.28 (d, *J* = 9.3 Hz, 1H), 4.64 (q, *J* = 7.6 Hz, 2H)
$^{13}C\{^1H\}$ NMR	(101 MHz, $CDCl_3$)<br>δ 154.6, 141. 9, 139.7, 129.3, 122.3 (d, *J* = 278.2 Hz), 122.2, 115.4, 67.25 (q, *J* = 37.3 Hz)
^{19}F NMR	(376 MHz, $CDCl_3$)<br>δ -73.45 (t, *J* = 7.6 Hz)
HRMS	(ESI^+)<br>Calcd for $C_9H_4F_6N_2O_5$ ($[M–H]^-$): 265.1242, found: 265.1240.

Synthesis of ethyl 2-cyano-2-(2,4-dinitrophenyl)-2-phenylacetate (30)

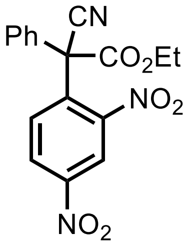

Following General Procedure 1, NaOEt (75 mg, 1.10 mmol, 1.10 equiv), 2,4-dinitrochlorobenzene (203 mg, 1.00 mmol, 1.00 equiv) and ethyl 2-cyano-2-phenylacetate (191 mg, 1.5 mmol, 1.5 equiv) were reacted in EtOH (10 mL). Following the work-up as described in the General Procedure, the crude product was purified by flash column chromatography (SiO_2, Celite dry loading, 15-20% EtOAc in petrol) to afford the title compound as an off-white solid (249 mg, 70%).

R_f	0.34 (20% EtOAc in petrol) [UV]
Melting point	126 °C (petrol)
^{1}H NMR	(500 MHz, $CDCl_3$)<br>δ 9.02 (d, J = 2.4 Hz, 1 H), 8.32 (dd, J = 8.8, 2.5 Hz, 1 H), 7.64 – 7.58 (m, 2 H), 7.58 – 7.52 (m, 3 H), 7.07 (d, J = 8.7 Hz, 1 H), 4.31 (m, 2 H), 1.42 (s, 1 H), 1.30 (t, J = 7.1 Hz, 3 H)
^{13}C{^{1}H} NMR	(126 MHz, $CDCl_3$)<br>δ 165.4, 148. 9, 148.25, 138.3, 133.9, 132.3, 130.6, 130.2, 128.2, 127.6, 121.4, 115.75, 64.6, 56.8, 13.9
HRMS	(ESI$^+$)<br>Calcd. for $C_{17}H_{14}O_6N_3$ ([M+H]$^+$): 356.0877, found: 356.0878.

Synthesis of ethyl 2-cyano-2-(4-cyano-2-nitrophenyl)-2-phenylacetate (31)

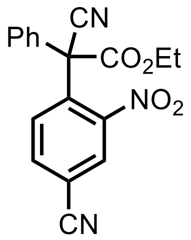

Following General Procedure 2, 4-fluoro-3-nitrobenzonitrile (80 mg, 0.63 mmol, 1.00 equiv), Na_2CO_3 (167 mg, 1.58 mmol, 2.50 equiv) and ethyl 2-cyano-2-phenylacetate (114 μL, 125 mg, 0.66 mmol, 1.05 equiv) were reacted in DMF (2.5 mL) for 2 h. Following the work-up as described in the General Procedure, the crude product was purified by flash column chromatography (SiO_2, loaded in CH_2Cl_2, 20% EtOAc in petrol) to afford the title compound as a pale yellow solid (172 mg, 82%).

R_f 0.21 [UV] (20% EtOAc in petrol)

Melting point 132–133 °C (petrol)

^1H NMR (500 MHz, DMSO-d_6)
δ 8.82 (d, J = 1.8 Hz, 1H), 8.20 (dd, J = 8.3, 1.8 Hz, 1H), 7.68 – 7.57 (m, 3H), 7.54 – 7.48 (m, 2H), 6.97 (d, J = 8.3 Hz, 1H), 4.35 – 4.27 (m, 1H), 4.27 – 4.18 (m, 1H), 1.16 (t, J = 7.1 Hz, 3H)

$^{13}C\{^1H\}$ NMR (126 MHz, DMSO-d_6)
165.3, 148.4, 138.6, 135.15, 133.2, 132.5, 130.9, 130.7, 128.2, 116.7, 116.5, 114.6, 64.7, 56.85, 14.0.

N.b. a ^{13}C environment is missing, attributed to overlapping peaks.

HRMS (APCI$^+$)
Calcd for $C_{18}H_{14}O_4N_3$ ([M+H]$^+$): 336.0979, found: 336.0976.

Synthesis of ethyl 2-cyano-2-(5-nitro-3-(trifluoromethyl)pyridin-2-yl)-2-phenylacetate (32).

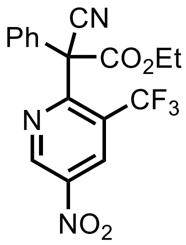

Following General Procedure 2, 2-bromo-5-nitro-3-(trifluoromethyl)pyridine (271 mg, 1.00 mmol, 1.00 equiv), Na_2CO_3 (265 mg, 2.50 mmol, 2.50 equiv) and ethyl 2-cyano-2-phenylacetate (165 µL, 180 mg, 1.05 mmol, 1.05 equiv) were reacted in DMF (5 mL) for 24 h. Following the work-up as described in the General Procedure, the crude product was purified by flash column chromatography (SiO_2, 10% EtOAc in hexane, loaded in CH_2Cl_2) to afford the title product as a pale yellow solid (250 mg, 66%).

R_f	0.44 (15% EtOAc in hexane) [UV]
Melting point	89–90 °C (EtOAc).
^{1}H NMR	(500 MHz, $CDCl_3$)<br>δ 9.48 (d, J = 2.4 Hz, 1H), 8.88 (d, J = 2.5 Hz, 1H), 7.48 – 7.39 (m, 3H), 7.42 – 7.36 (m, 2H), 4.46 – 4.29 (m, 2H), 1.33 (t, J = 7.1 Hz, 3H)
^{13}C{^{1}H} NMR	(126 MHz, $CDCl_3$)<br>δ 165.2, 157.2, 145.9, 143.5, 133.2 (q, J = 5.0 Hz), 132.5, 129.6, 129.0, 128.15, 127.6 (q, J = 35.7 Hz), 121. 9 (q, J = 275.4 Hz), 115.7, 64.5, 61.2, 13.85
^{19}F NMR	(471 MHz, $CDCl_3$)<br>δ –56.3
HRMS	(APCI$^+$)<br>Calcd for $C_{17}H_{13}O_4N_3F_3$ ([M+H]$^+$): 380.0853, found: 380.0845

Protein and DNA sequences for S_NAr1.3 and S_NAr$_{Ph}$1.0

S_NAr1.3

MIRAVFFDSLGTLISVEGAYKVRLKIMEEVLGDYPLNPKTLLDEYEKLAREAFSNYAGKPYRPMRDILE
EVMRKLAEKYGFKYPENLREISLRMACRYGELYPEVVEVLKSLKGKYHVGDILQRDTEPATAFLDALGI
KDLFDSITTSEEAGFFKPHPRIFELALKKAGVKGEKAVYVGDNPVKDAGGSKNLGMTSILLDRKGEKR
EFWDKADFIVSDLREVIKIVDELNGQGSLEHHHHHH

ATGATTCGTGCGGTATTCTTTGATAGCCTGGGTACTCTGATTAGCGTTGAAGGCGCTTATAAAGTG
AGGCTGAAAATTATGGAGGAAGTGCTGGGTGACTATCCGCTGAACCCGAAAACCCTGCTGGACG
AATACGAGAAACTGGCTCGCGAAGCGTTCTCTAACTATGCGGGCAAACCGTATCGTCCGATGCGT
GATATCCTGGAAGAAGTAATGCGTAAACTGGCGGAAAAGTACGGTTTCAAATACCCTGAAAACTTG
CGGGAAATCTCCCTGCGTATGGCGTGTCGCTACGGCGAGCTGTACCCGGAAGTGGTGGAAGTAC
TGAAATCTCTGAAAGGTAAATATCACGTTGGCGATATCCTGCAGAGGGATACCGAGCCGGCCACG
GCATTCCTGGACGCACTGGGCATCAAAGACCTGTTCGATTCCATCACCACGTCTGAAGAAGCTGG
TTTCTTTAAACCGCACCCACGCATCTTCGAACTGGCTCTGAAGAAAGCCGGCGTTAAAGGCGAGA
AAGCAGTGTACGTTGGTGACAACCCGGTCAAAGACGCGGGTGGTTCTAAGAACCTGGGTATGAC
TAGCATCCTGCTGGATCGTAAAGGTGAGAAACGTGAATTCTGGGATAAGGCGGACTTTATCGTCTC
CGACCTGCGCGAAGTTATTAAGATTGTTGACGAACTGAACGGTCAGGGCTCTCTCGAGCACCAC
CACCACCACCAC

S_NAr$_{Ph}$1.0

MIRAVFFDSLGTLLSVEGAYKVRLKIMEEVLGDYPLNPKTLLDEYEKLAREAFSNYAGKPYRPLRDILE
EVGRKLAEKYGFKYPENLREISLRMAQRYGELYPEVVEVLKSLKGKYHVGDILQRDTEPATAFLDALGI
KDLFDSITTSEEAGFFKPHPRIFELALKKAGVKGEKAVYVGDNPVKDAGGSKNLGMTSILLDRKGEKR
EFWDKADFIVSDLREVIKIVDELNGQGSLEHHHHHH

ATGATTCGTGCGGTATTCTTTGATAGCCTGGGTACTCTGTTGAGCGTTGAAGGCGCTTATAAAGTG
AGGCTGAAAATTATGGAGGAAGTGCTGGGTGACTATCCGCTGAACCCGAAAACCCTGCTGGACG
AATACGAGAAACTGGCTCGCGAAGCGTTCTCTAACTATGCGGGCAAACCGTATCGTCCGCTGCGT
GATATCCTGGAAGAAGTAGGTCGTAAACTGGCGGAAAAGTACGGTTTCAAATACCCTGAAAACTTG
CGGGAAATCTCCCTGCGTATGGCGCAACGCTACGGCGAGCTGTACCCGGAAGTGGTGGAAGTAC
TGAAATCTCTGAAAGGTAAATATCACGTTGGCGATATCCTGCAGAGGGATACCGAGCCGGCCACG
GCATTCCTGGACGCACTGGGCATCAAAGACCTGTTCGATTCCATCACCACGTCTGAAGAAGCTGG
TTTCTTTAAACCGCACCCACGCATCTTCGAACTGGCTCTGAAGAAAGCCGGCGTTAAAGGCGAGA
AAGCAGTGTACGTTGGTGACAACCCGGTCAAAGACGCGGGTGGTTCTAAGAACCTGGGTATGAC
TAGCATCCTGCTGGATCGTAAAGGTGAGAAACGTGAATTCTGGGATAAGGCGGACTTTATCGTCTC
CGACCTGCGCGAAGTTATTAAGATTGTTGACGAACTGAACGGTCAGGGCTCTCTCGAGCACCAC
CACCACCACCAC

NMR spectra of chemically synthesised substrates and product standards

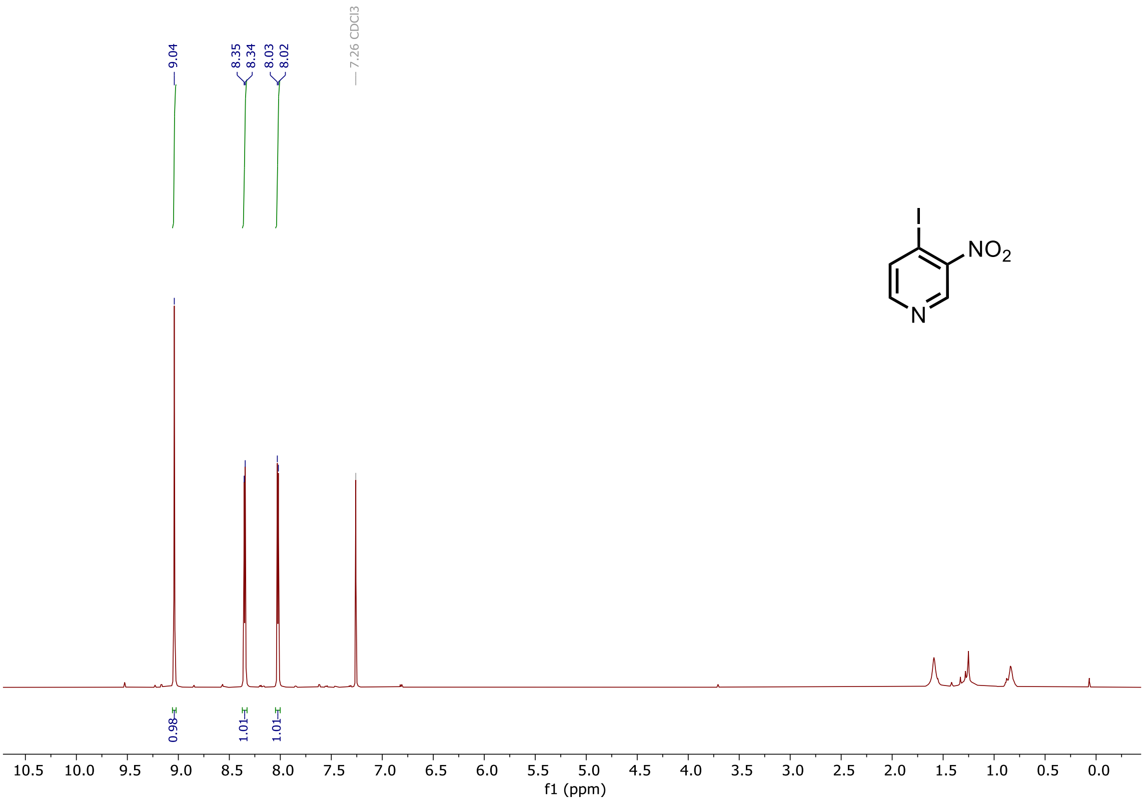

^{1}H NMR (400 MHz, $CDCl_3$) of **SM1**

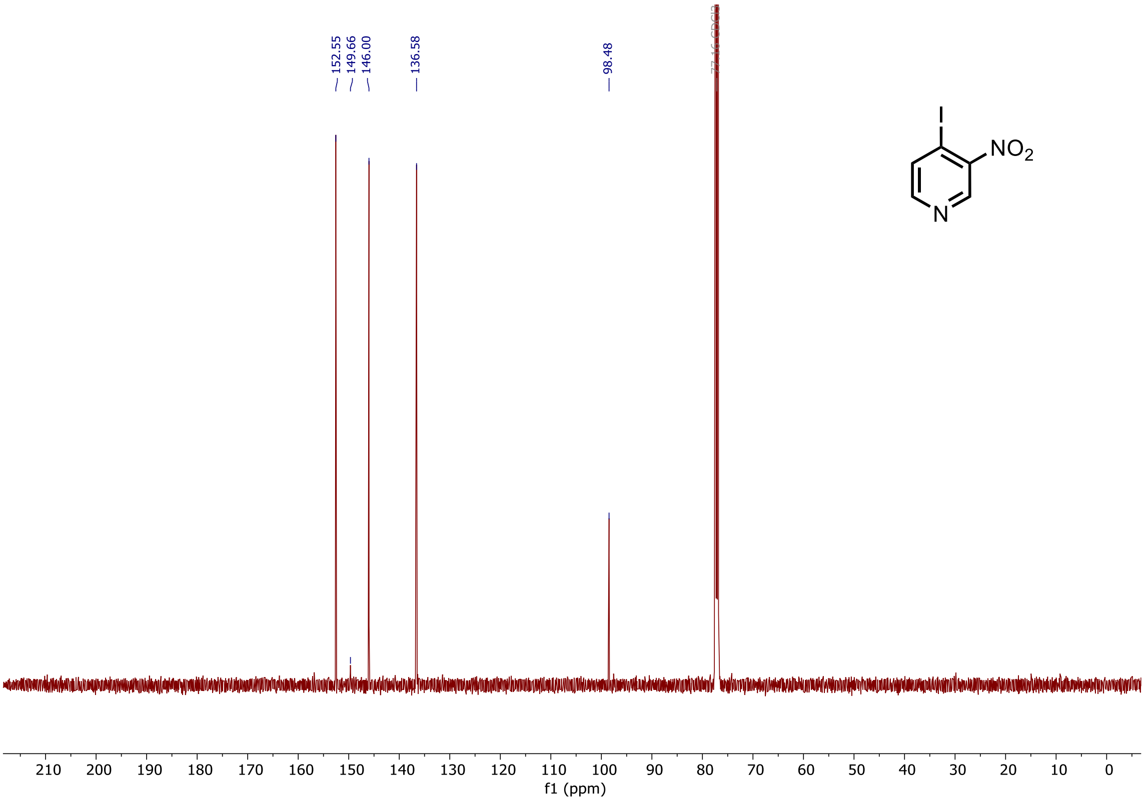

^{13}C{^{1}H} NMR (101 MHz, $CDCl_3$) of **SM1**

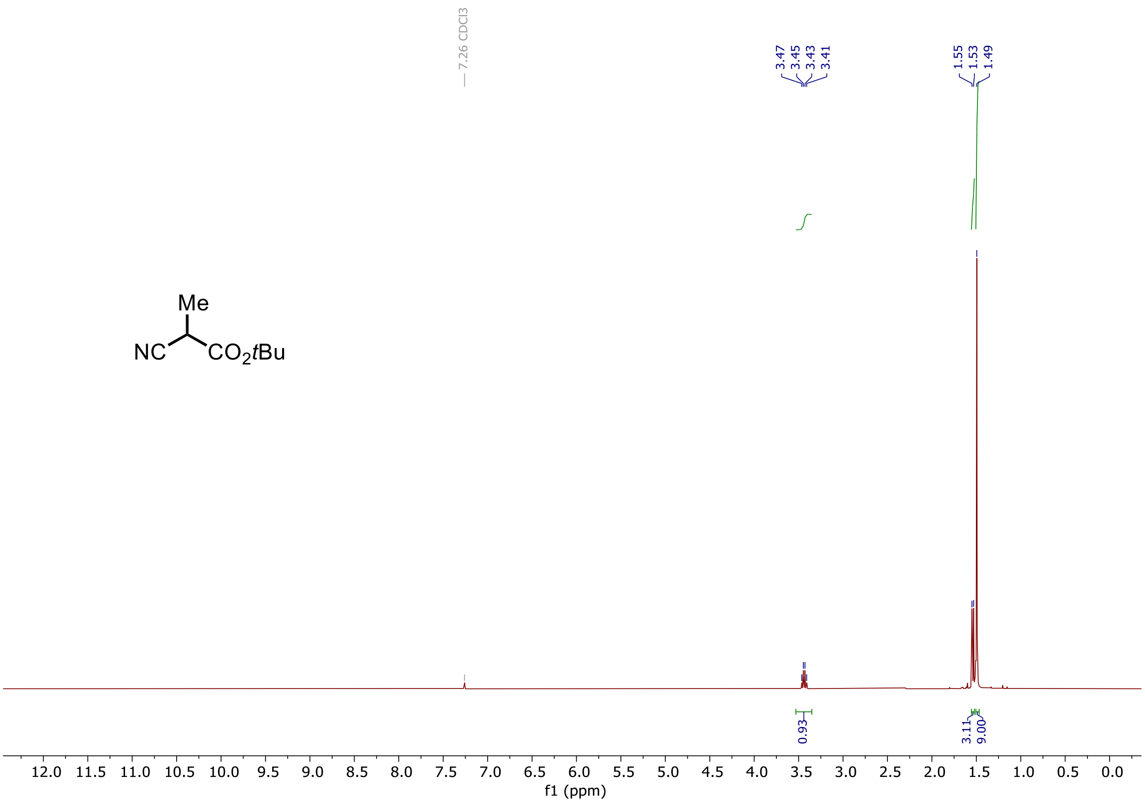

^{1}H NMR (400 MHz, $CDCl_3$) of **SM2**

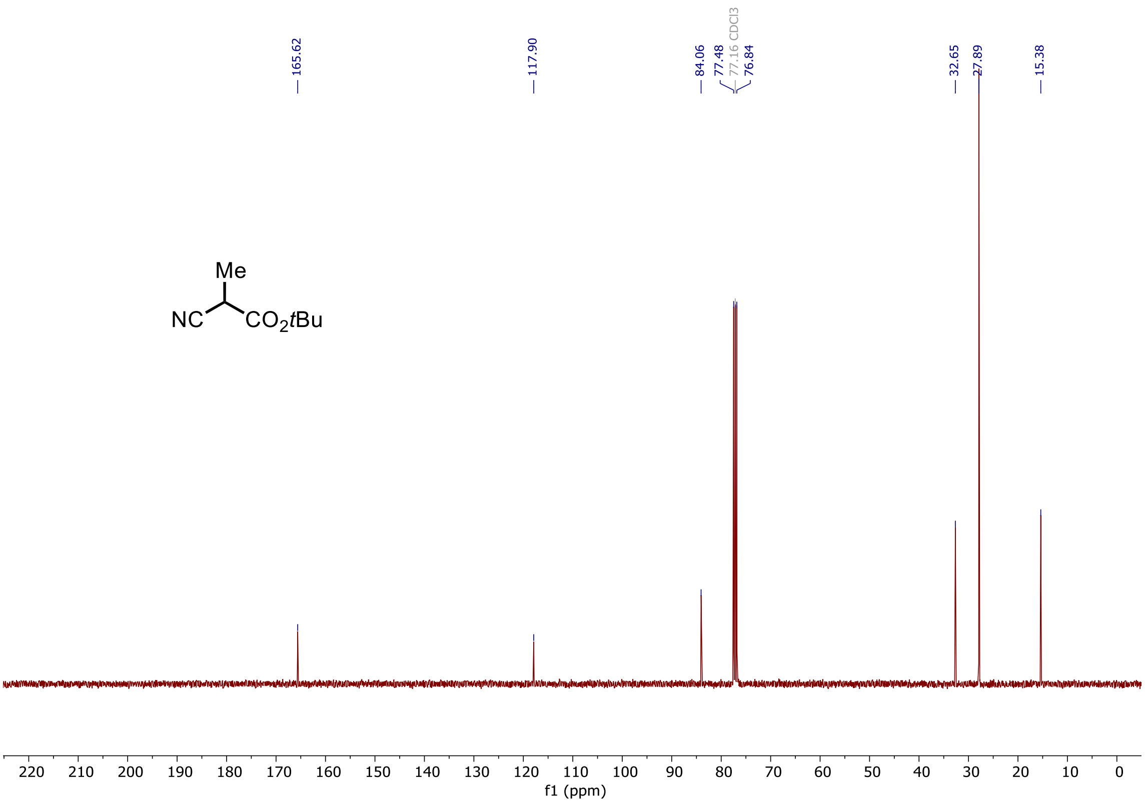

$^{13}C\{^1H\}$ NMR (101 MHz, $CDCl_3$) of **SM2**

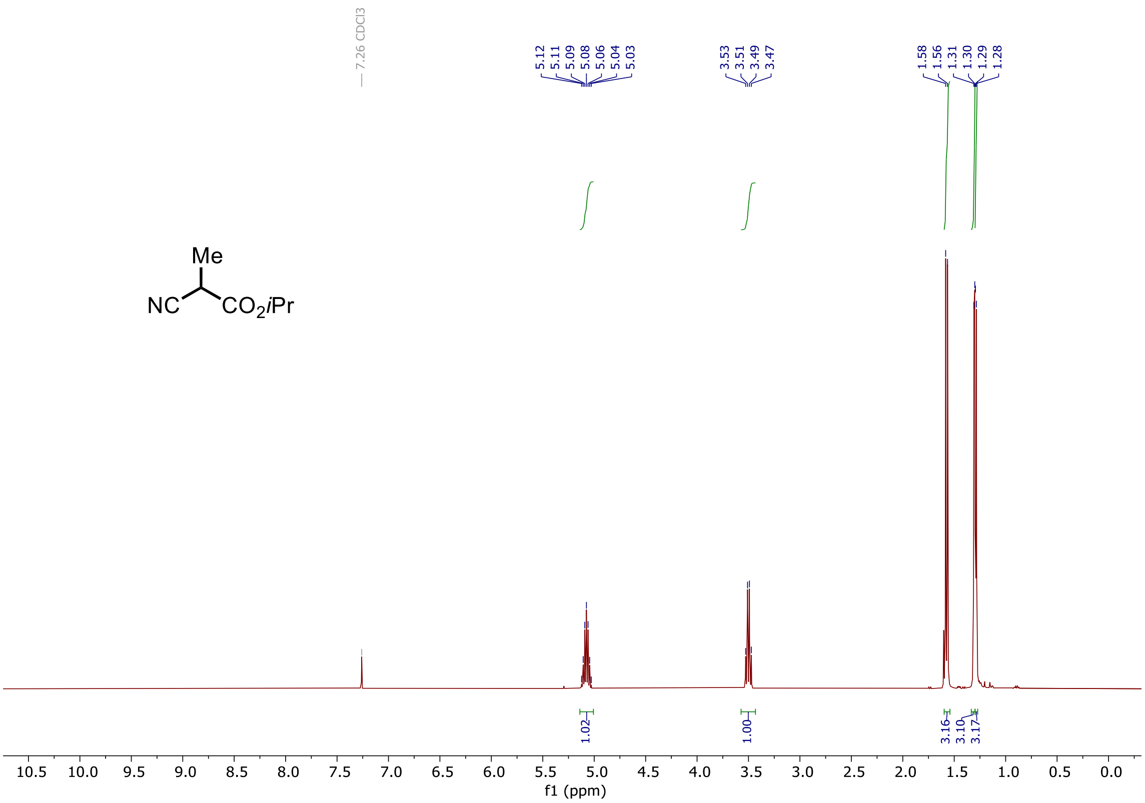

^{1}H NMR (400 MHz, $CDCl_3$) of **SM3**

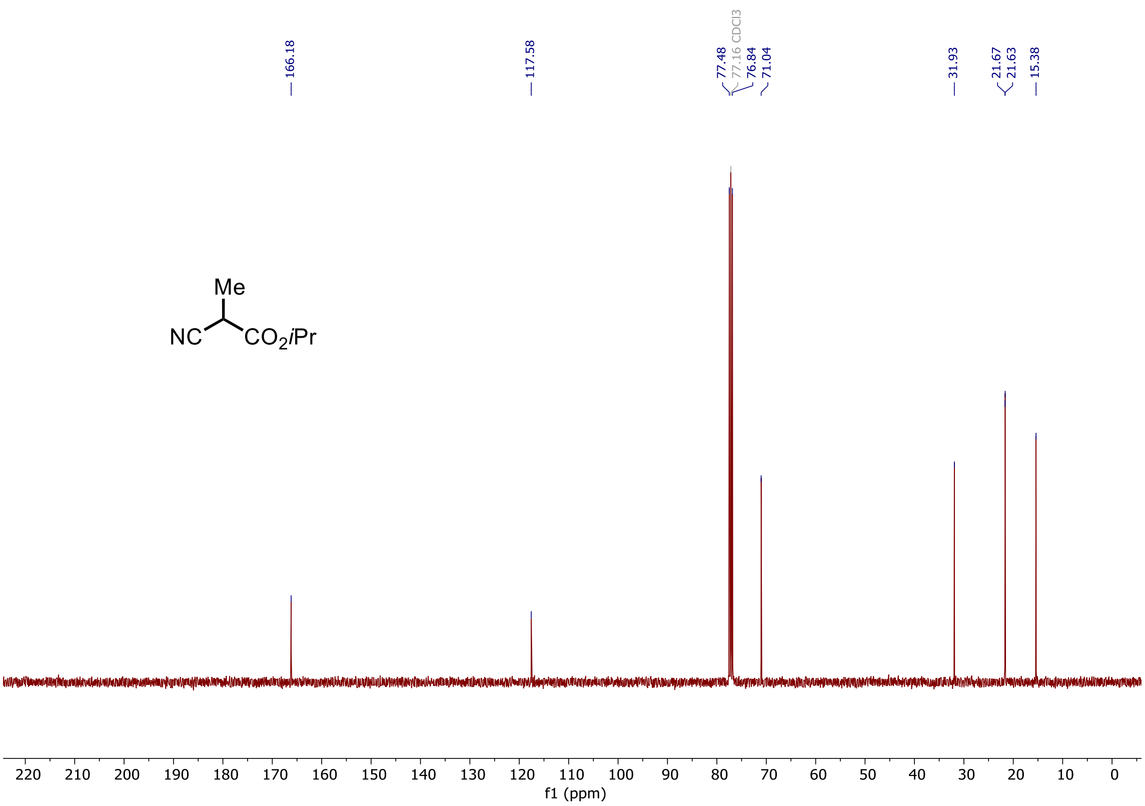

^{13}C{^{1}H} NMR (101 MHz, $CDCl_3$) of **SM3**

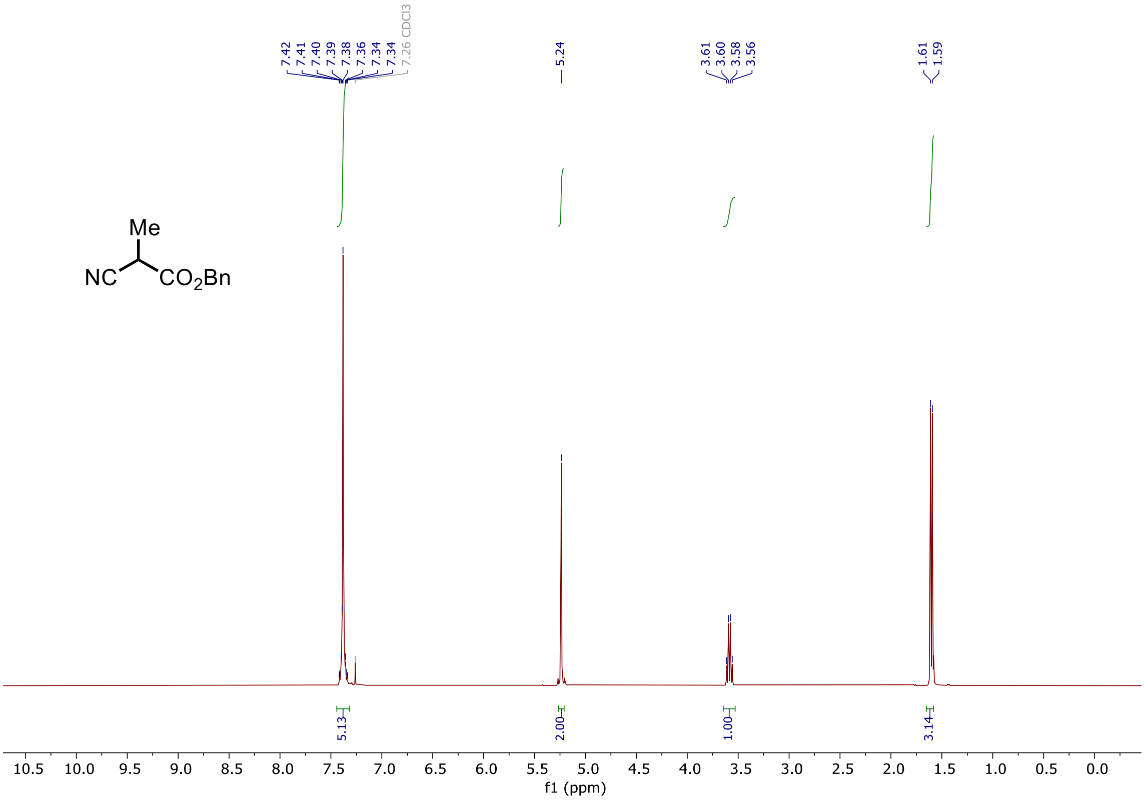

^{1}H NMR (400 MHz, $CDCl_3$) of **SM4**

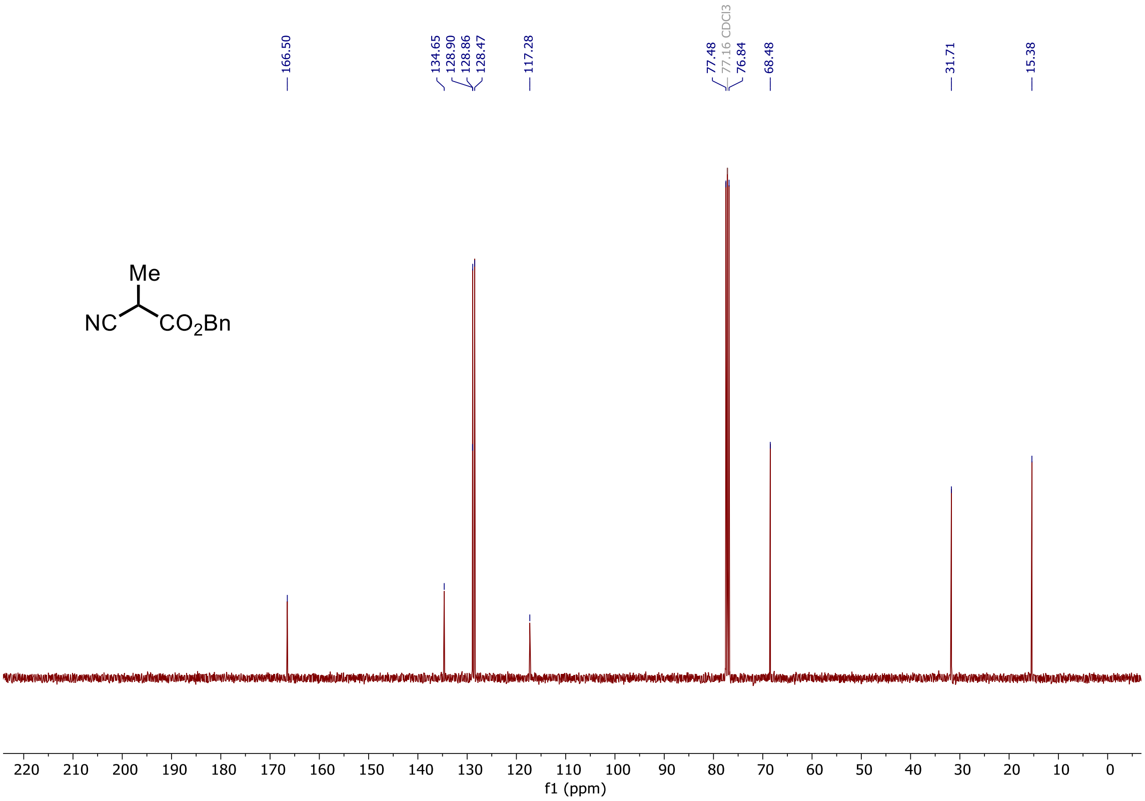

$^{13}C\{^{1}H\}$ NMR (101 MHz, $CDCl_3$) of **SM4**

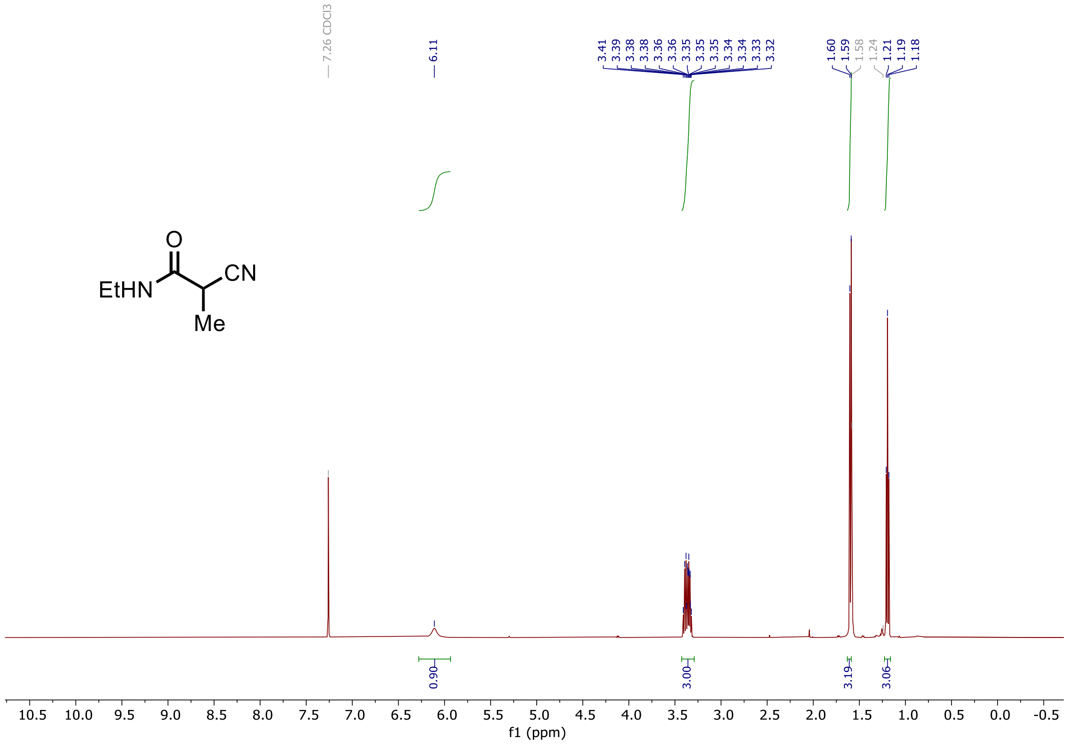

^{1}H NMR (500 MHz, $CDCl_3$) of **SM5**

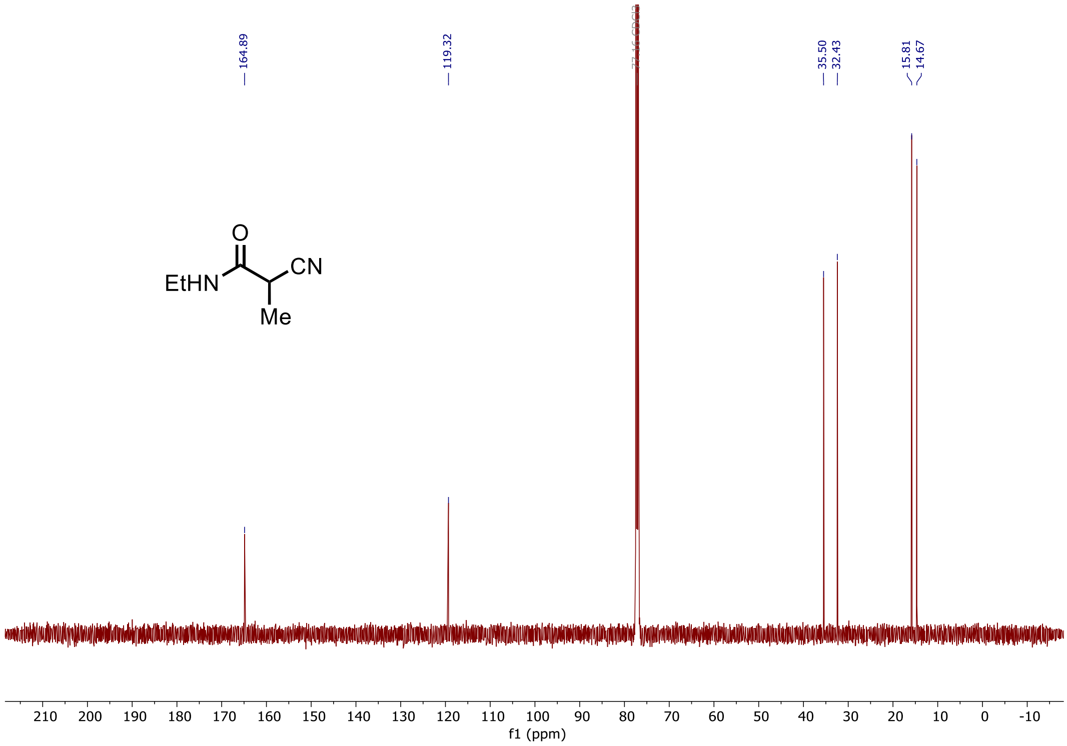

^{13}C NMR (126 MHz, $CDCl_3$) of **SM5**

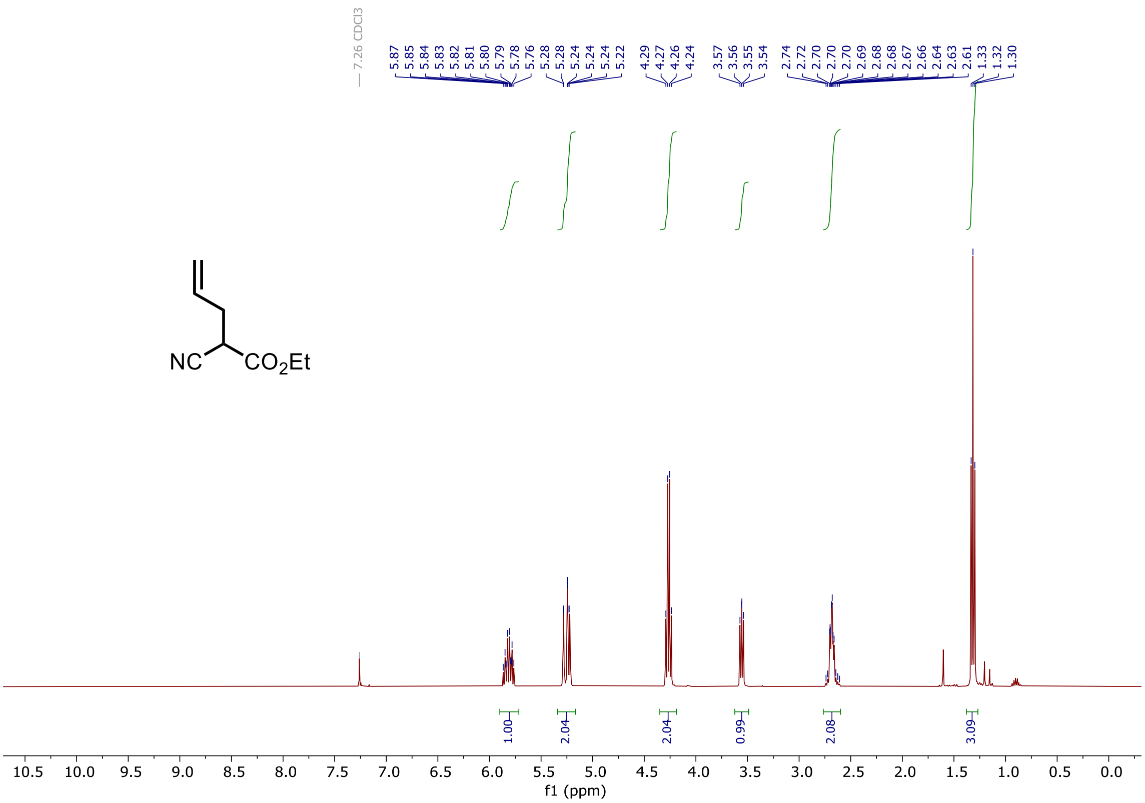

^1H NMR (400 MHz, $CDCl_3$) of **SM6**

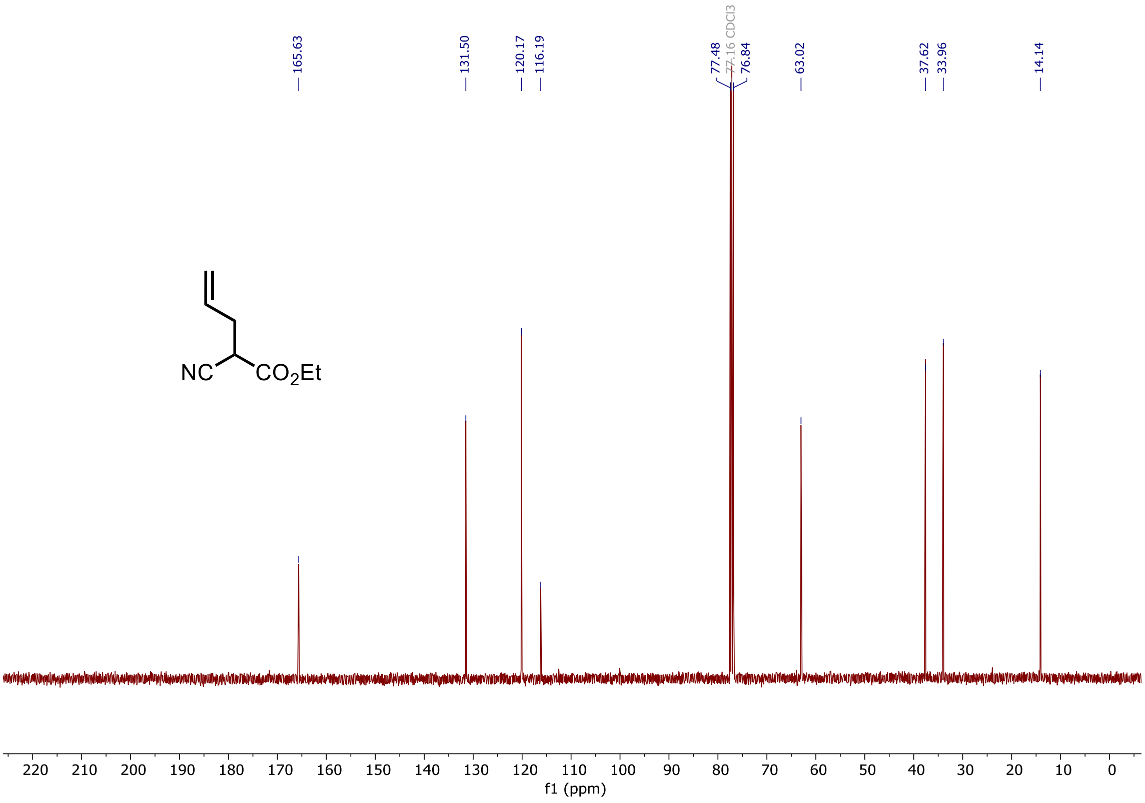

$^{13}C\{^1H\}$ NMR (101 MHz, $CDCl_3$) of **SM6**

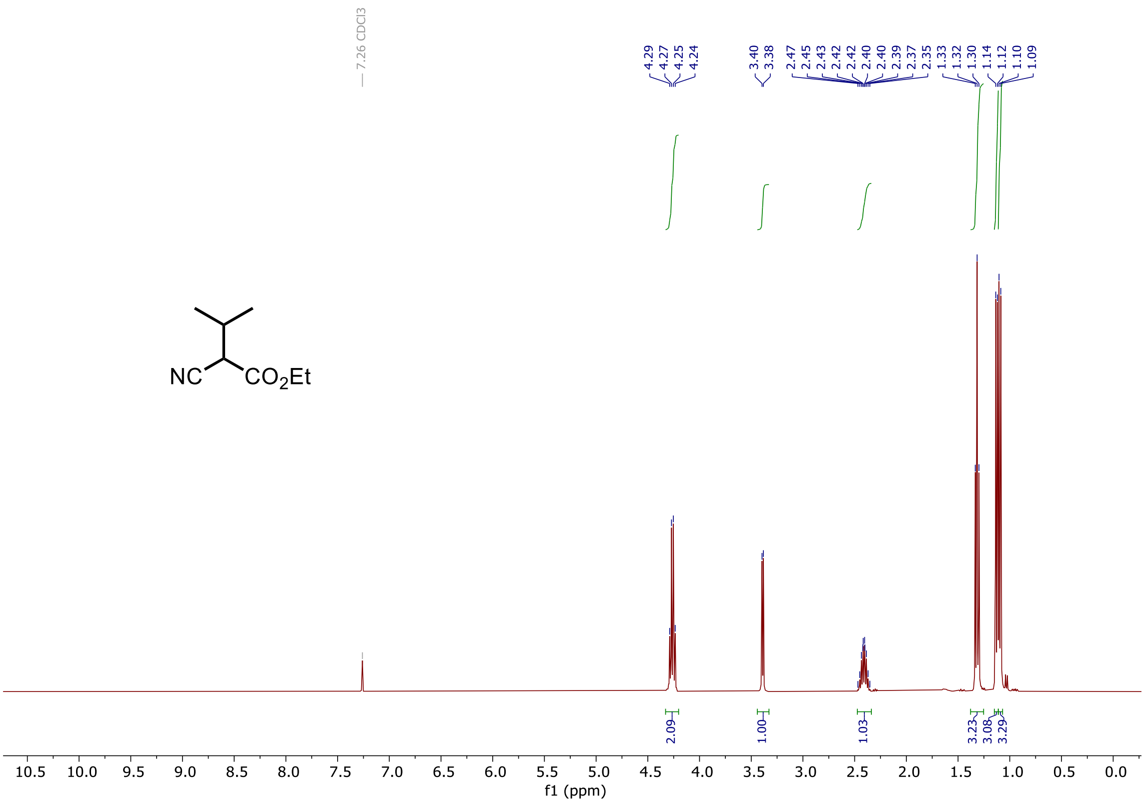

^{1}H NMR (400 MHz, $CDCl_3$) of **SM7**

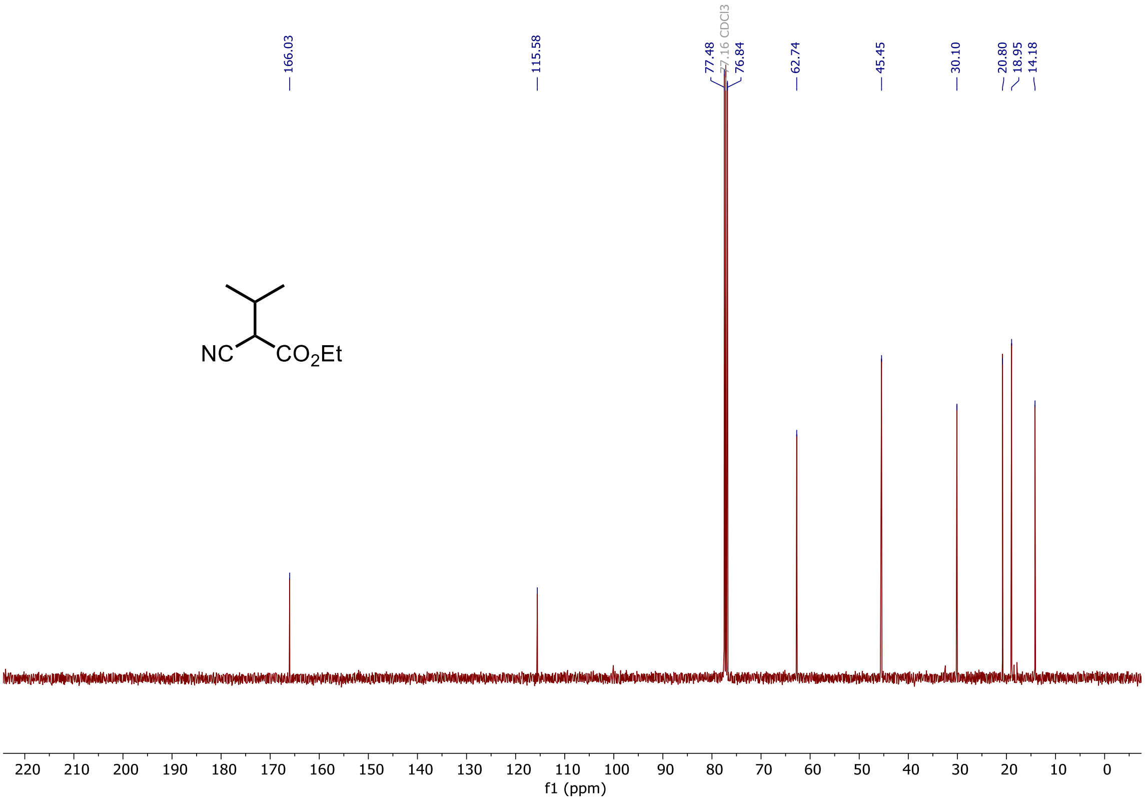

$^{13}C\{^{1}H\}$ NMR (101 MHz, $CDCl_3$) of **SM7**

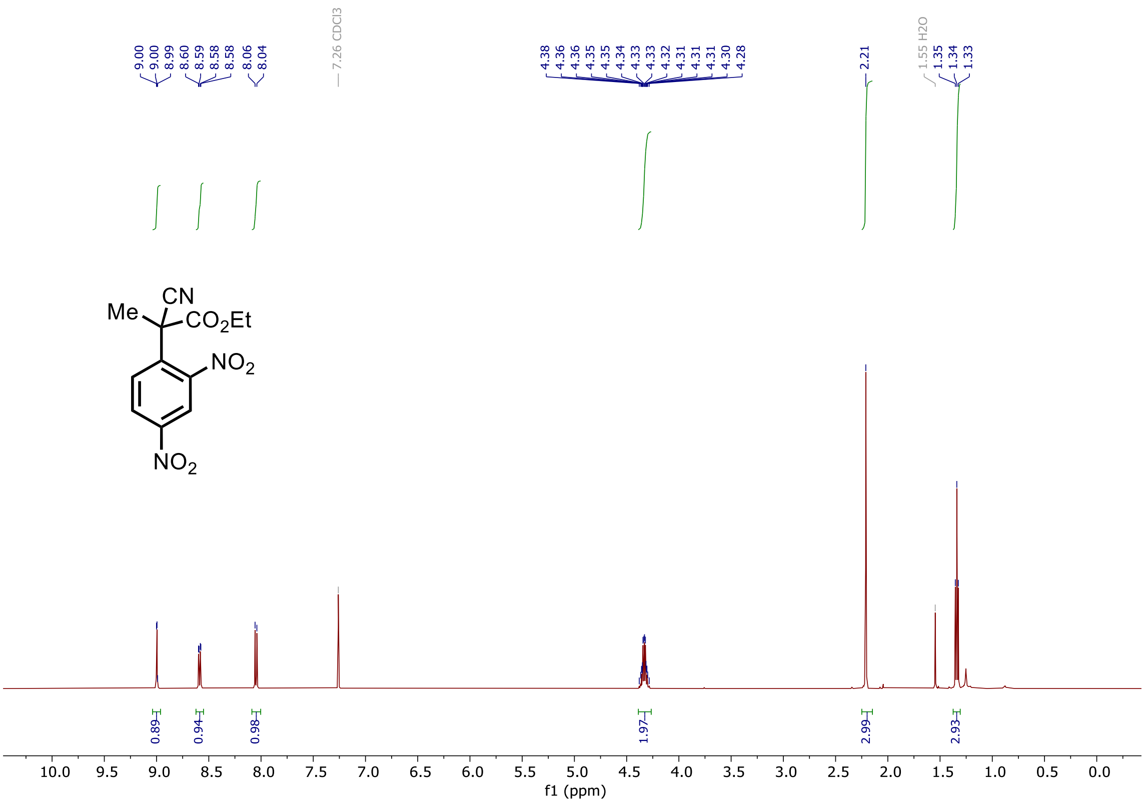

^{1}H NMR (500 MHz, $CDCl_3$) of **3**

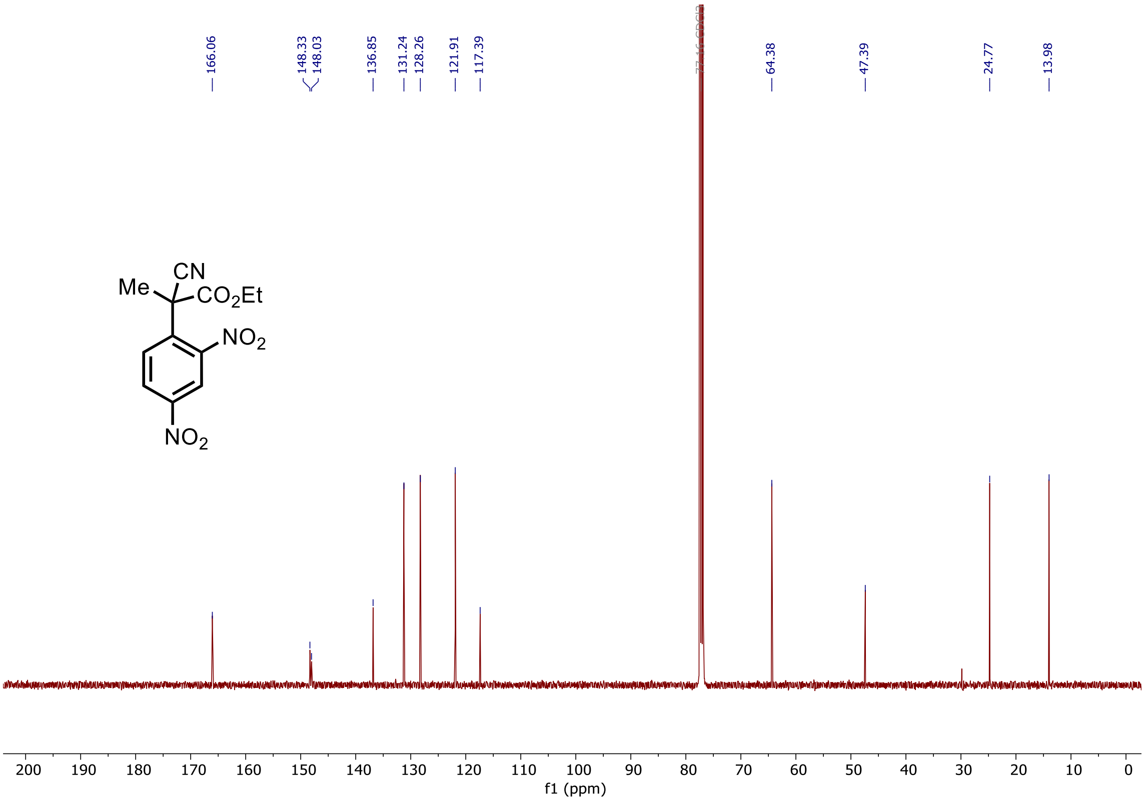

$^{13}C\{^{1}H\}$ NMR (126 MHz, $CDCl_3$) of **3**

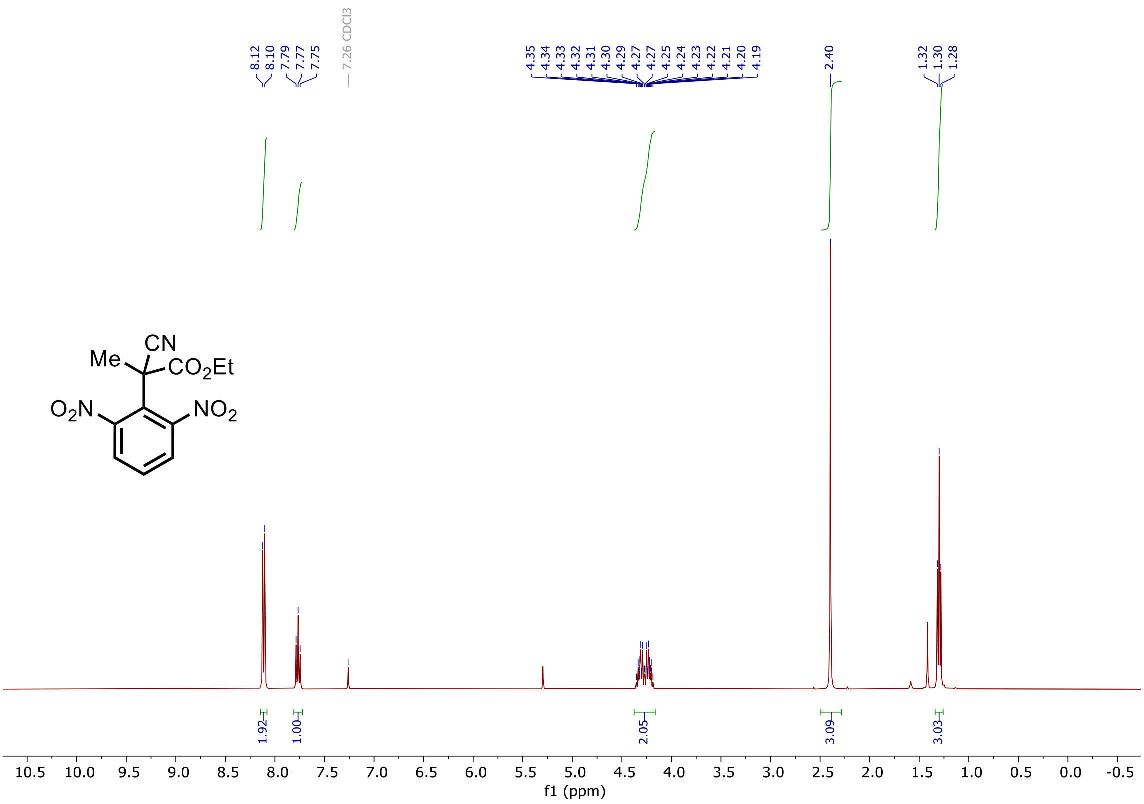

^{1}H NMR (400 MHz, $CDCl_3$) of **7**

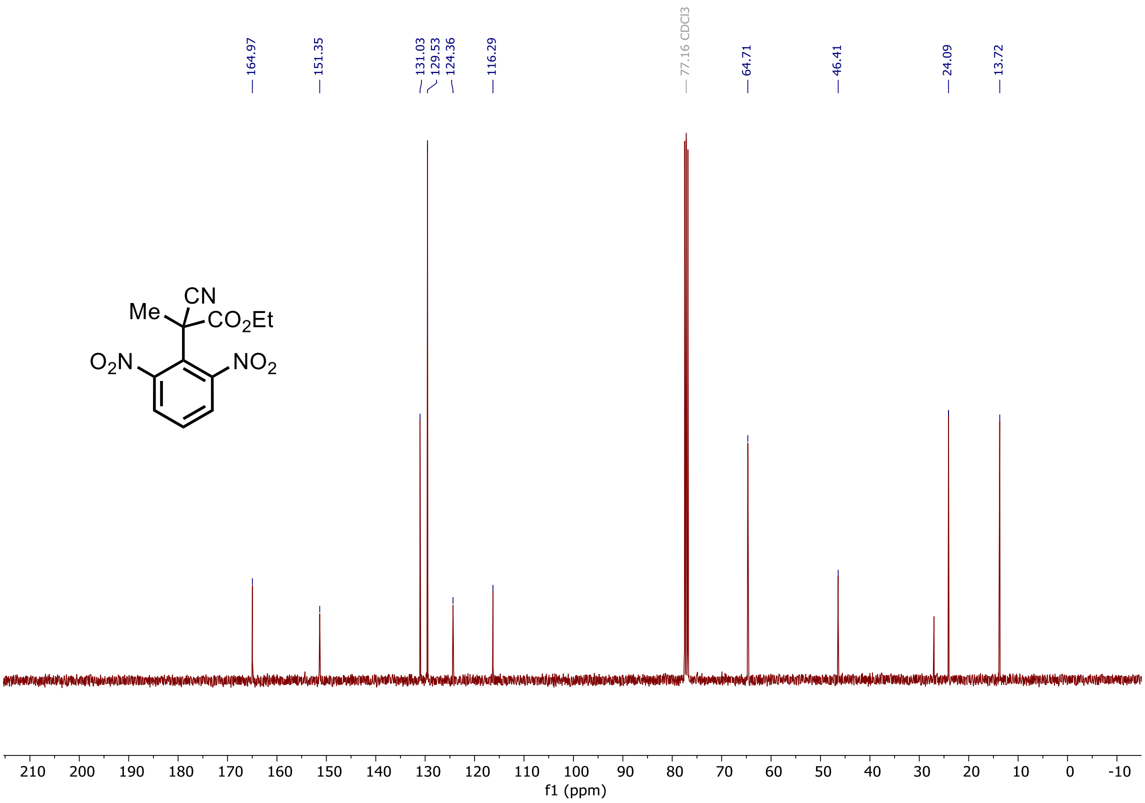

$^{13}C\{^{1}H\}$ NMR (101 MHz, $CDCl_3$) of **7**

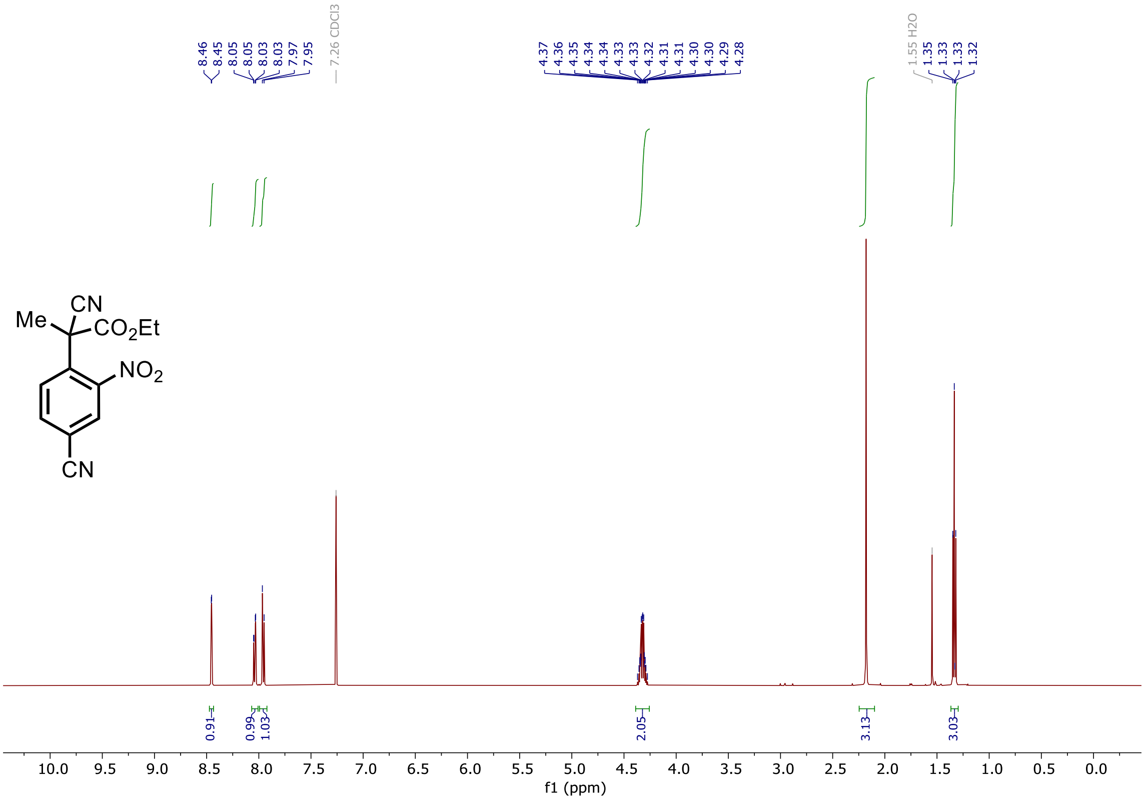

^{1}H NMR (500 MHz, $CDCl_3$) of **8**

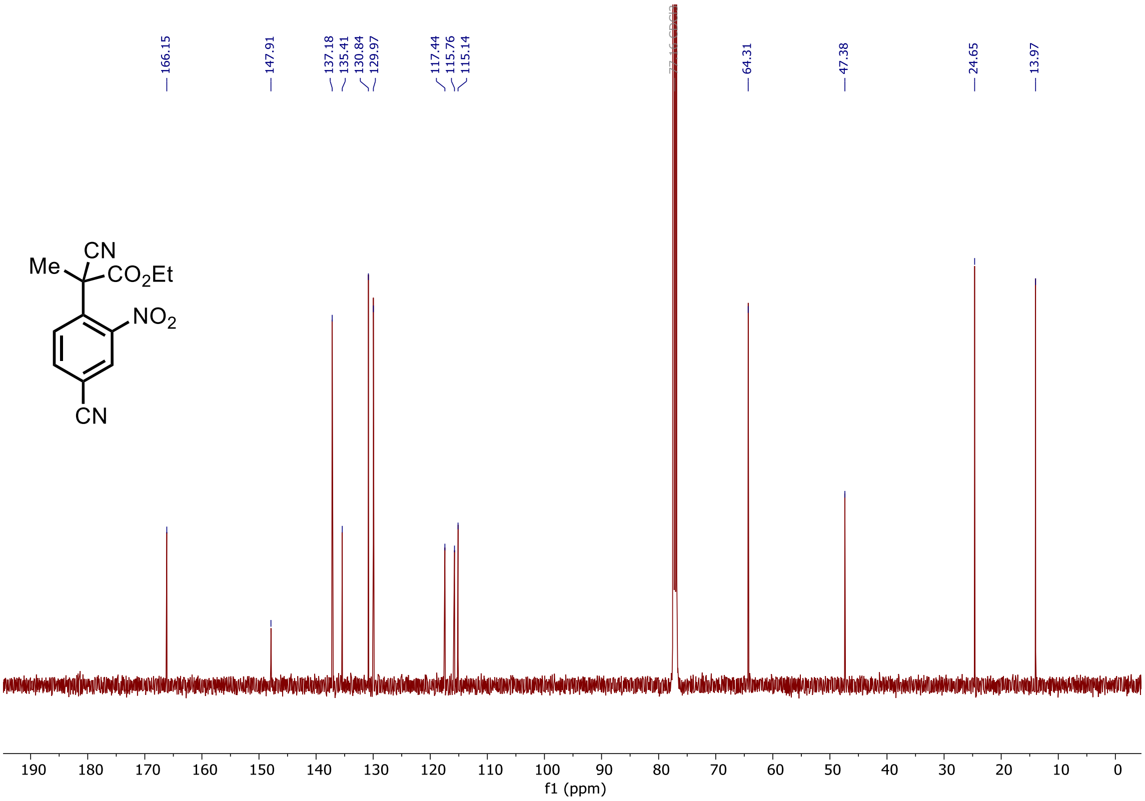

$^{13}C\{^{1}H\}$ NMR (126 MHz, $CDCl_3$) of **8**

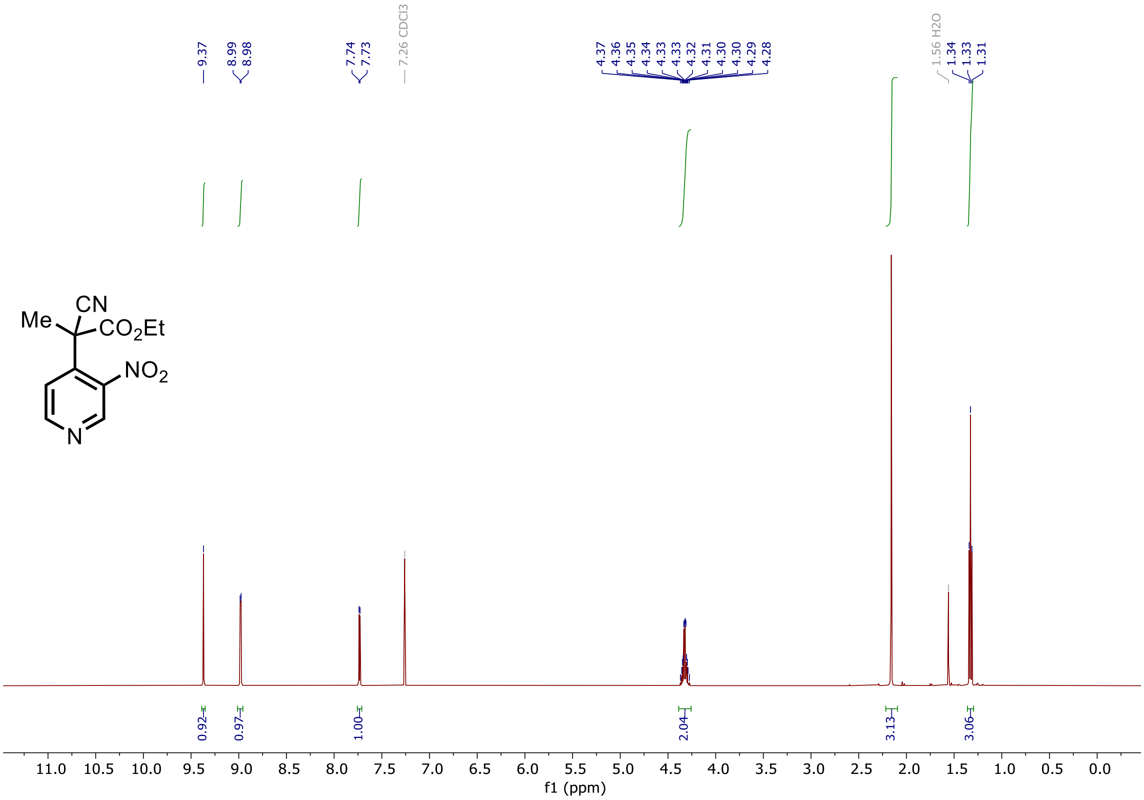

^1H NMR (500 MHz, $CDCl_3$) of **9**

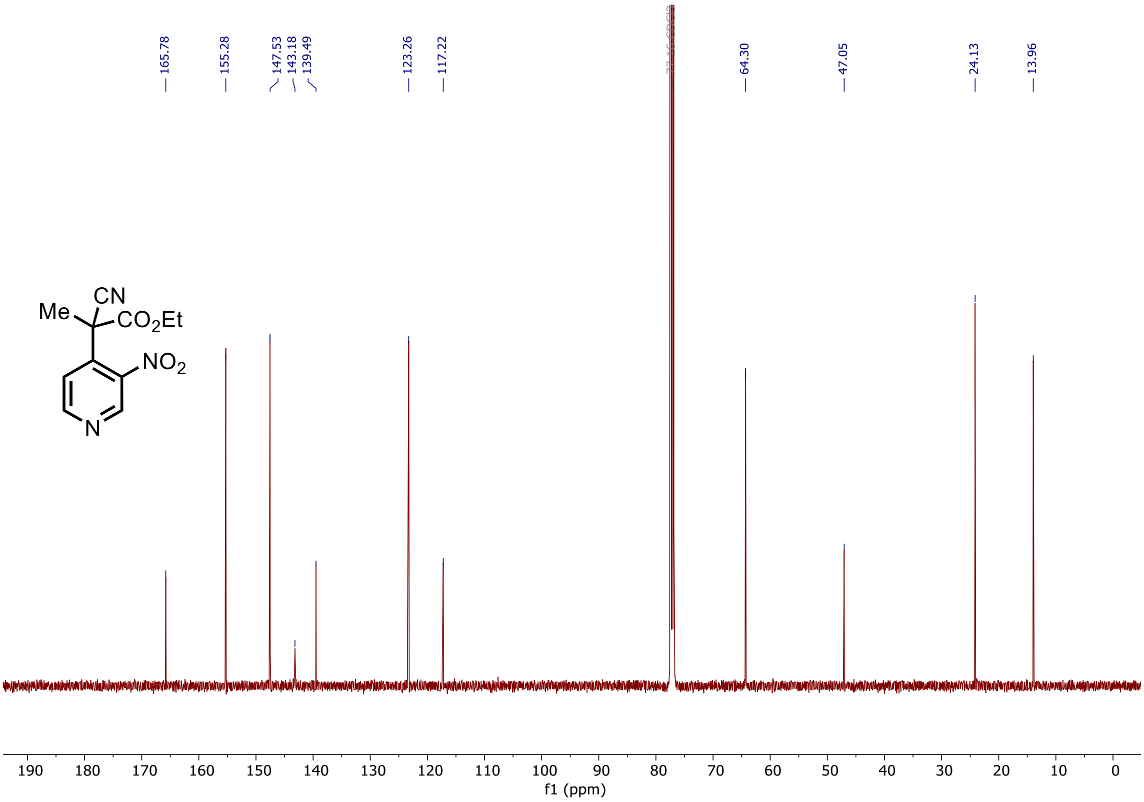

$^{13}C\{^1H\}$ NMR (126 MHz, $CDCl_3$) of **9**

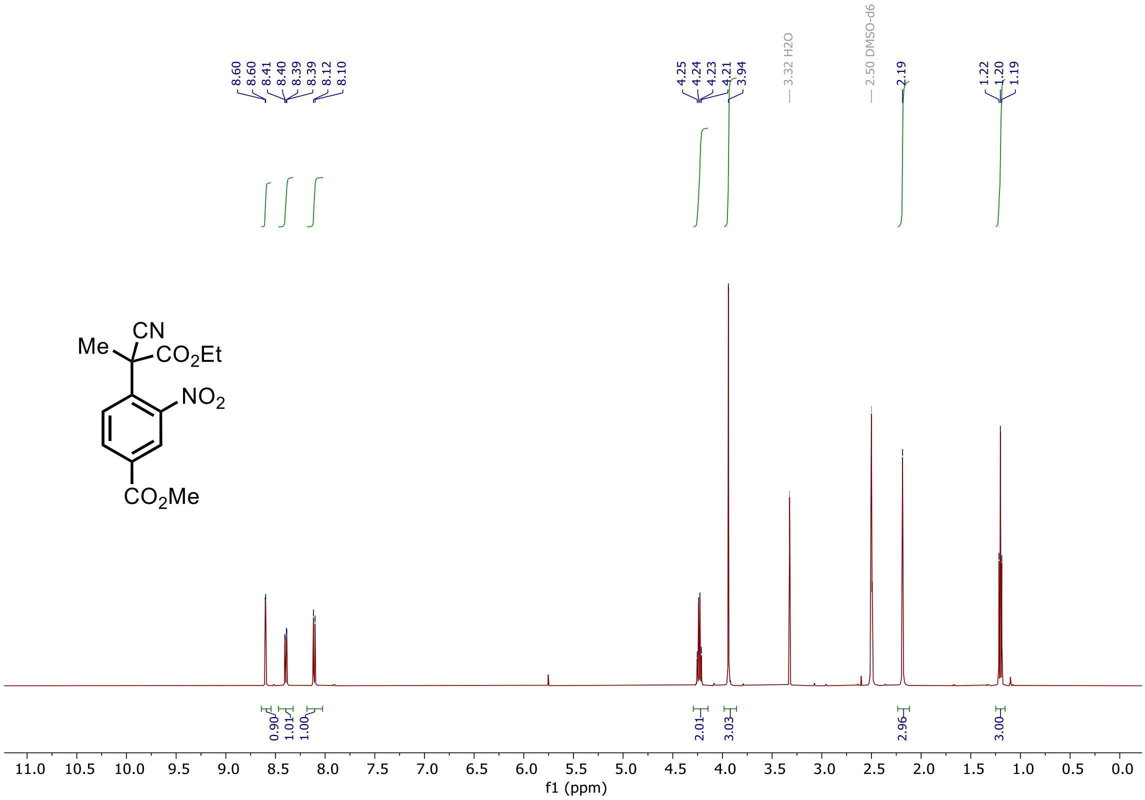

^{1}H NMR (500 MHz, DMSO-d_6) of **10**

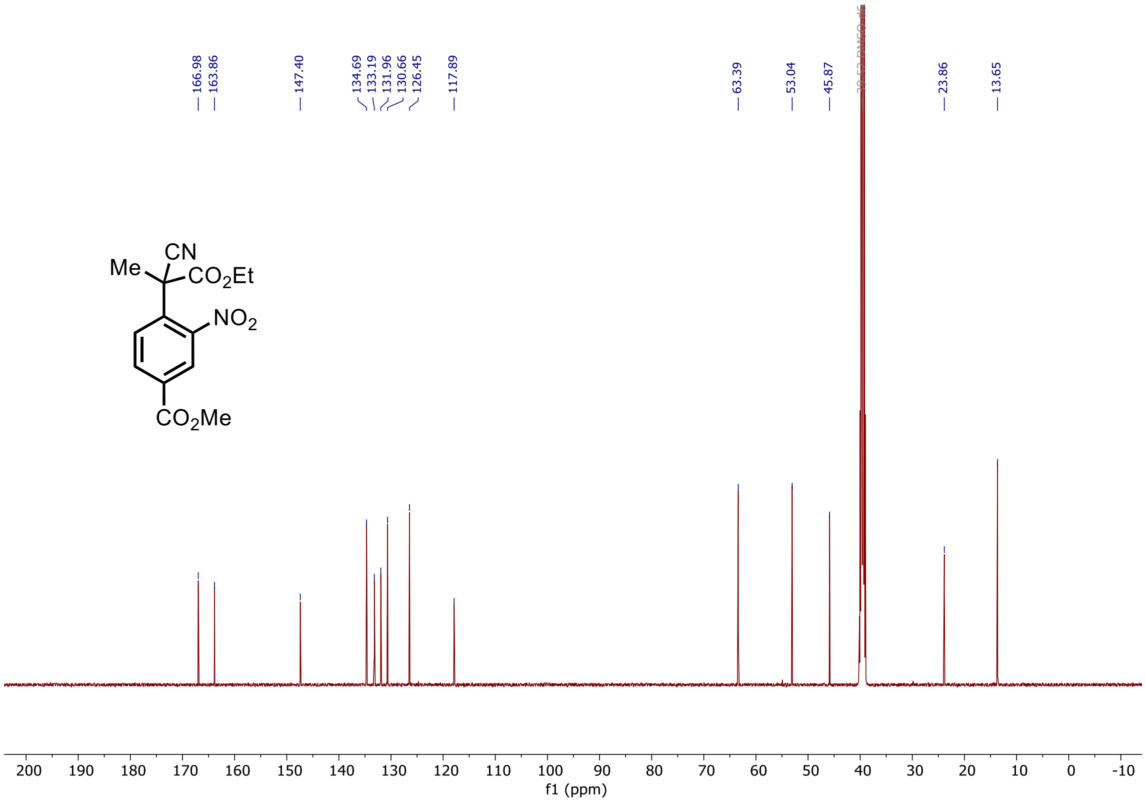

^{13}C{^{1}H} NMR (126 MHz, DMSO-d_6) of **10**

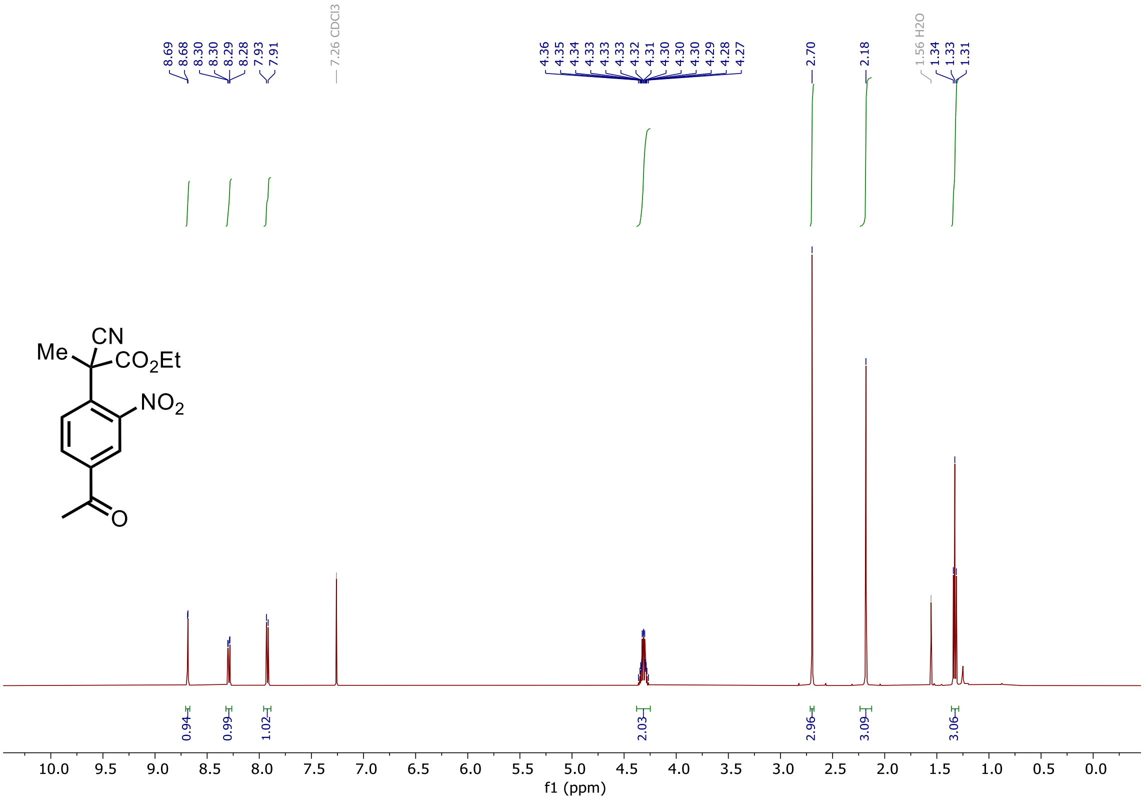

^{1}H NMR (500 MHz, $CDCl_3$) of **12**

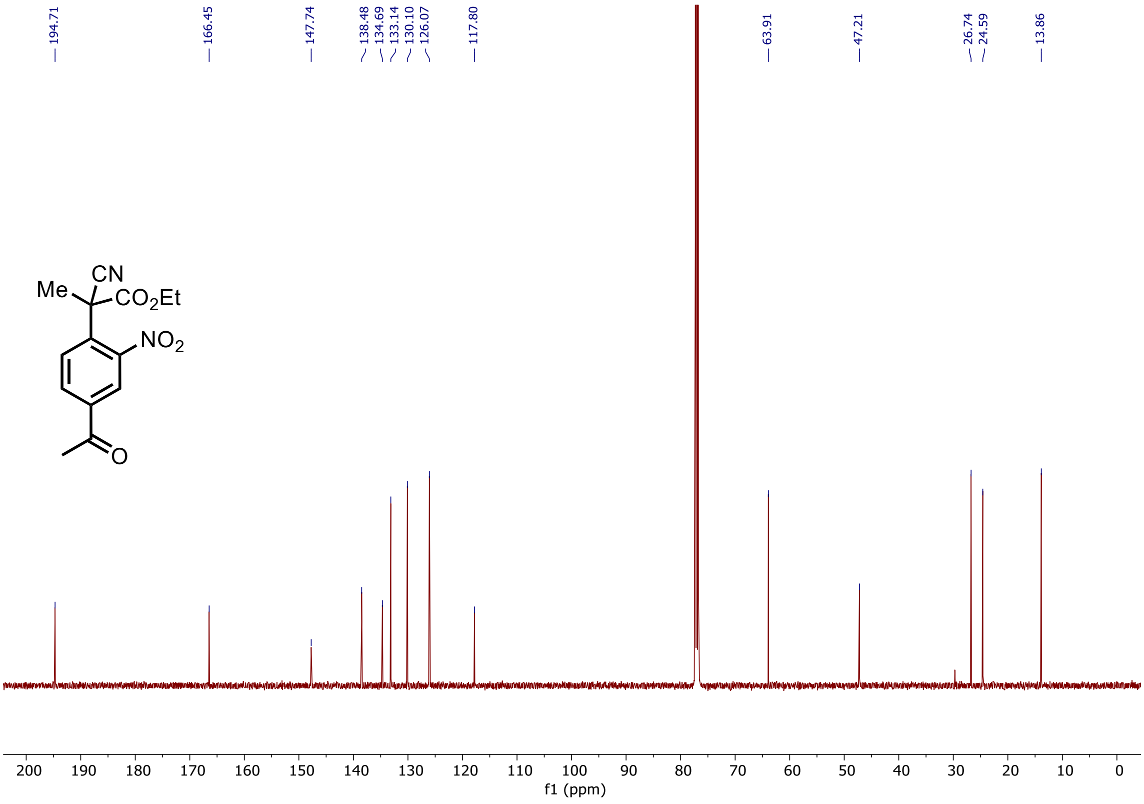

^{13}C{^{1}H} NMR (126 MHz, $CDCl_3$) of **12**

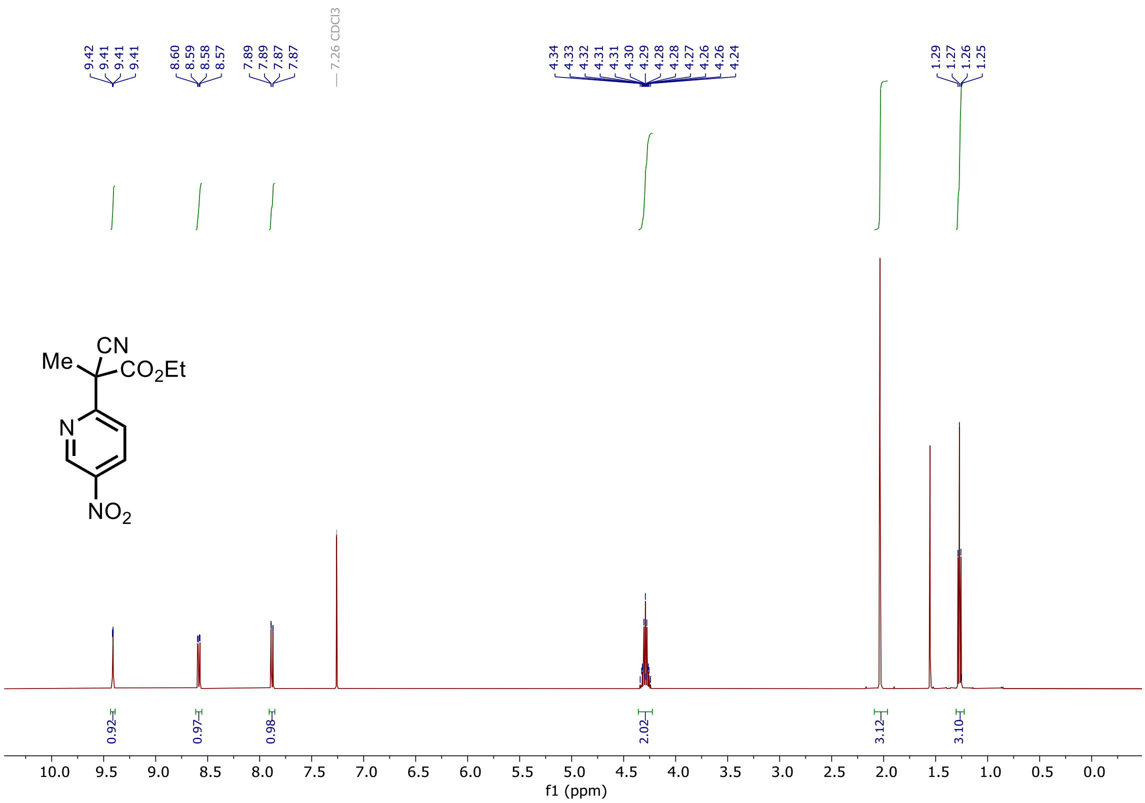

^{1}H NMR (500 MHz, $CDCl_3$) of **12**

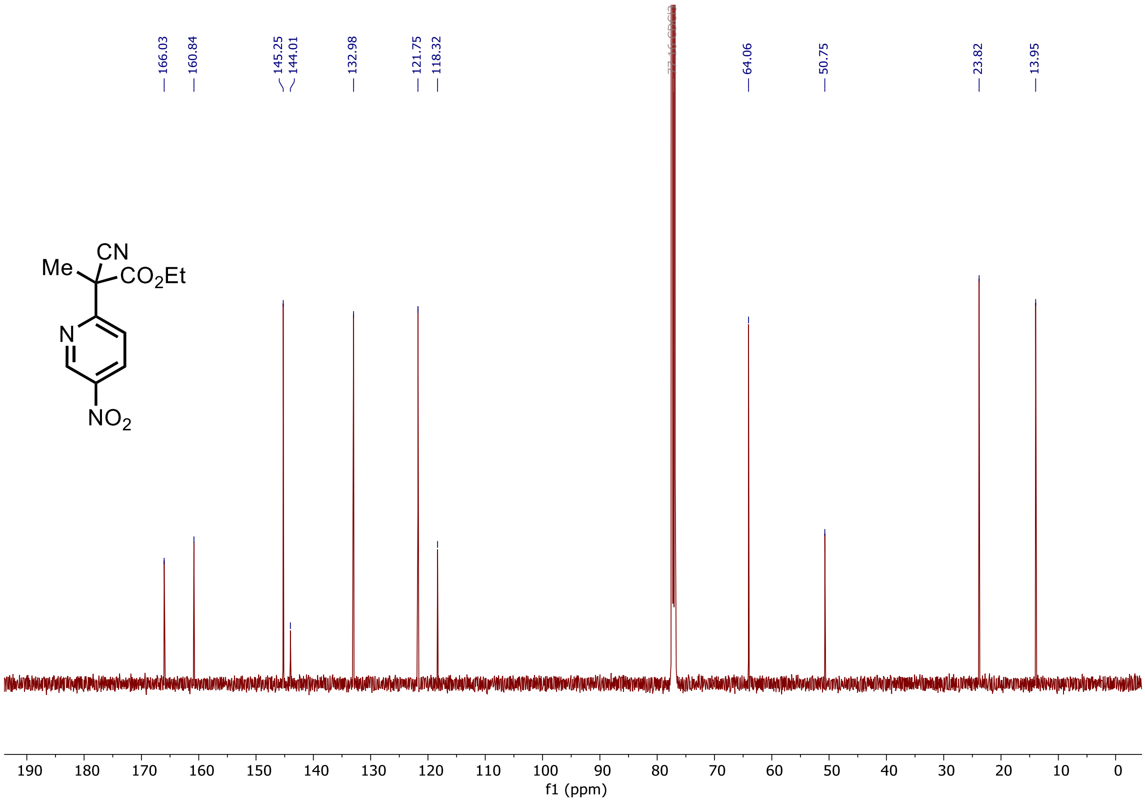

^{13}C{^{1}H} NMR (126 MHz, $CDCl_3$) of **12**

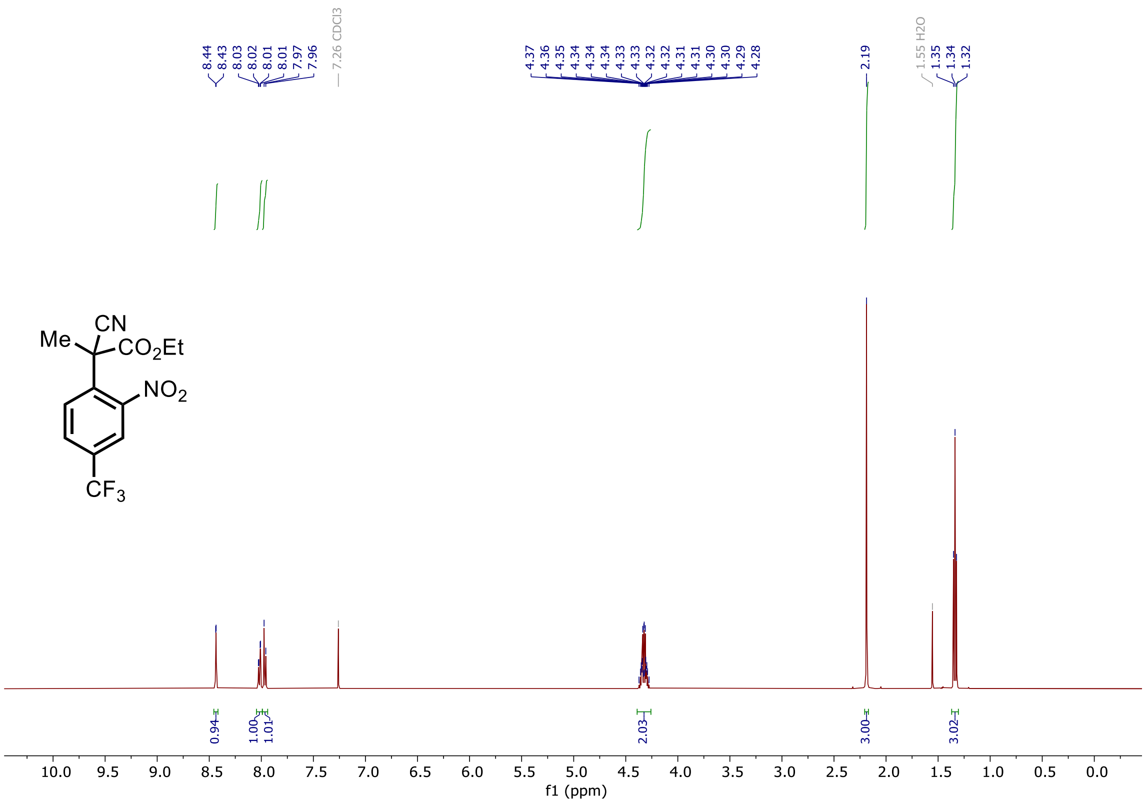

^{1}H NMR (500 MHz, $CDCl_3$) of **13**

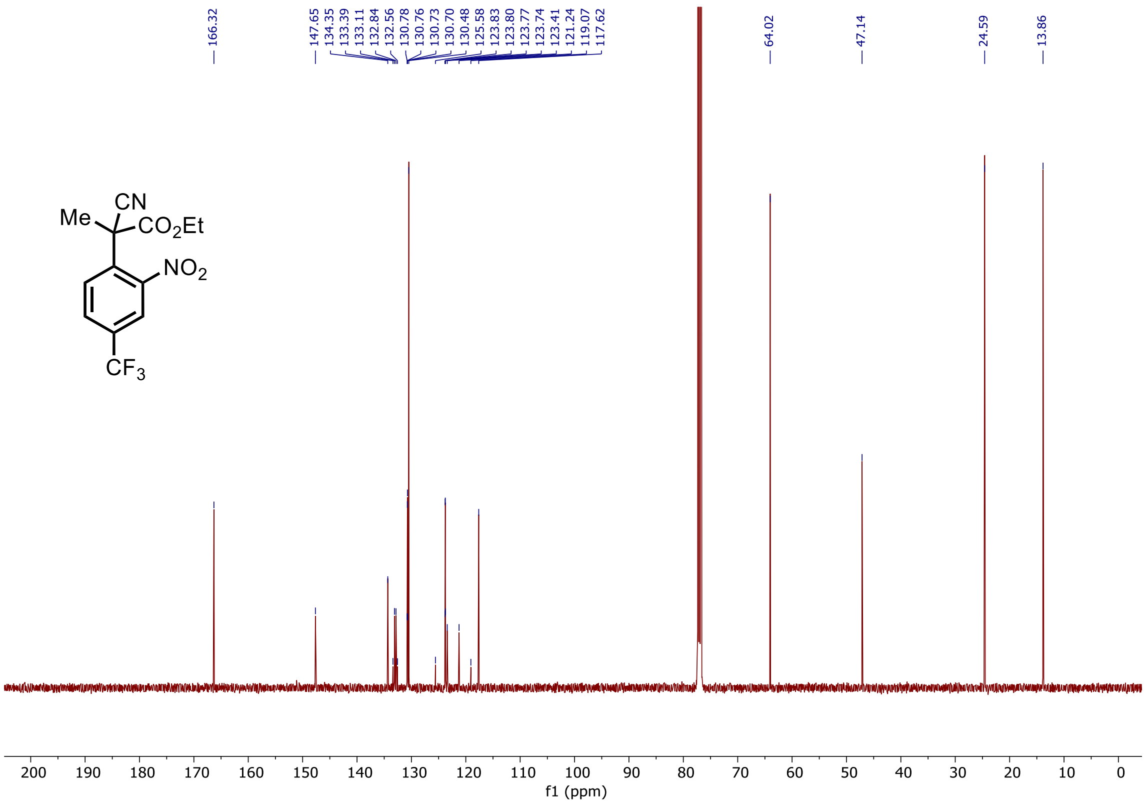

$^{13}C\{^{1}H\}$ NMR (126 MHz, $CDCl_3$) of **13**

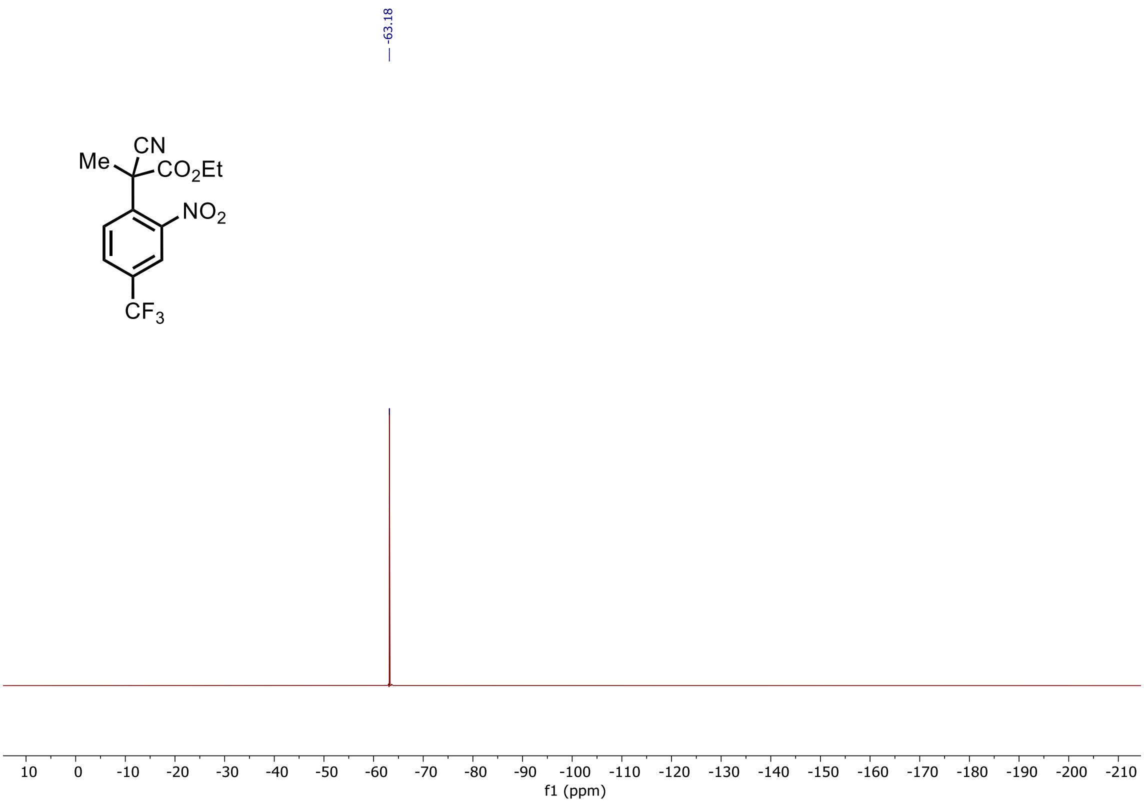

^{19}F NMR (471 MHz, $CDCl_3$) of **13**

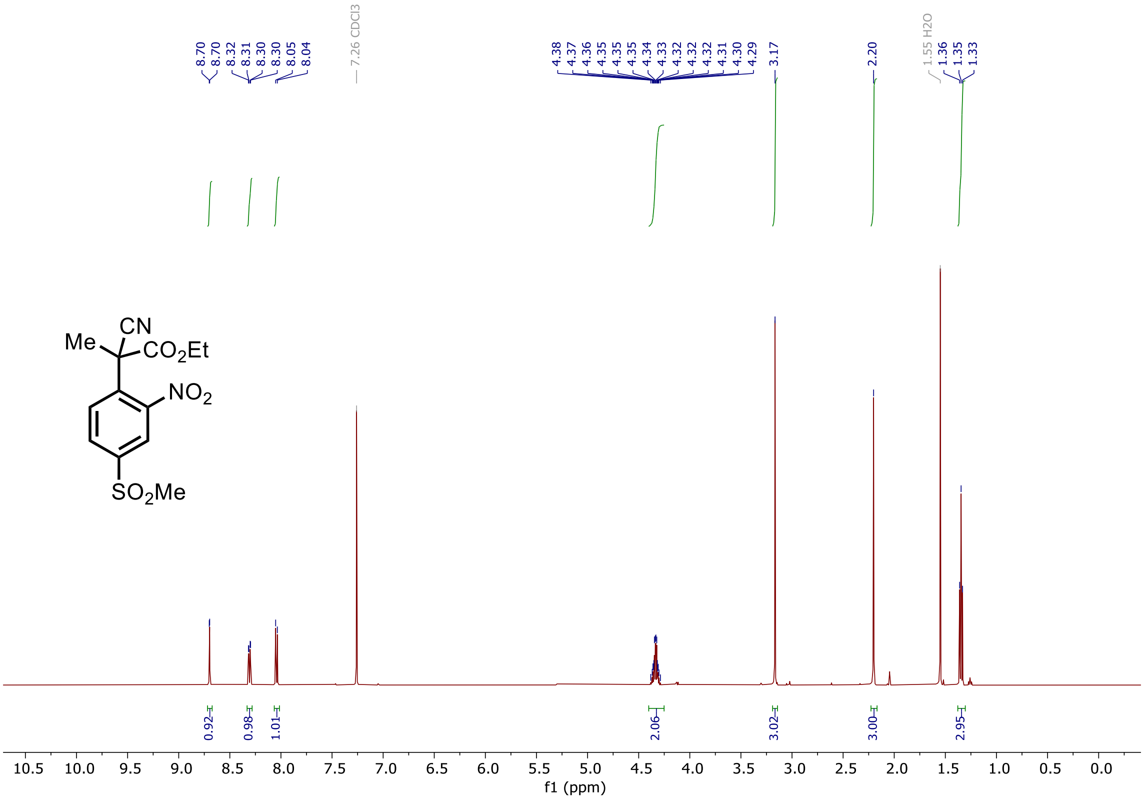

^{1}H NMR (500 MHz, $CDCl_3$) of **14**

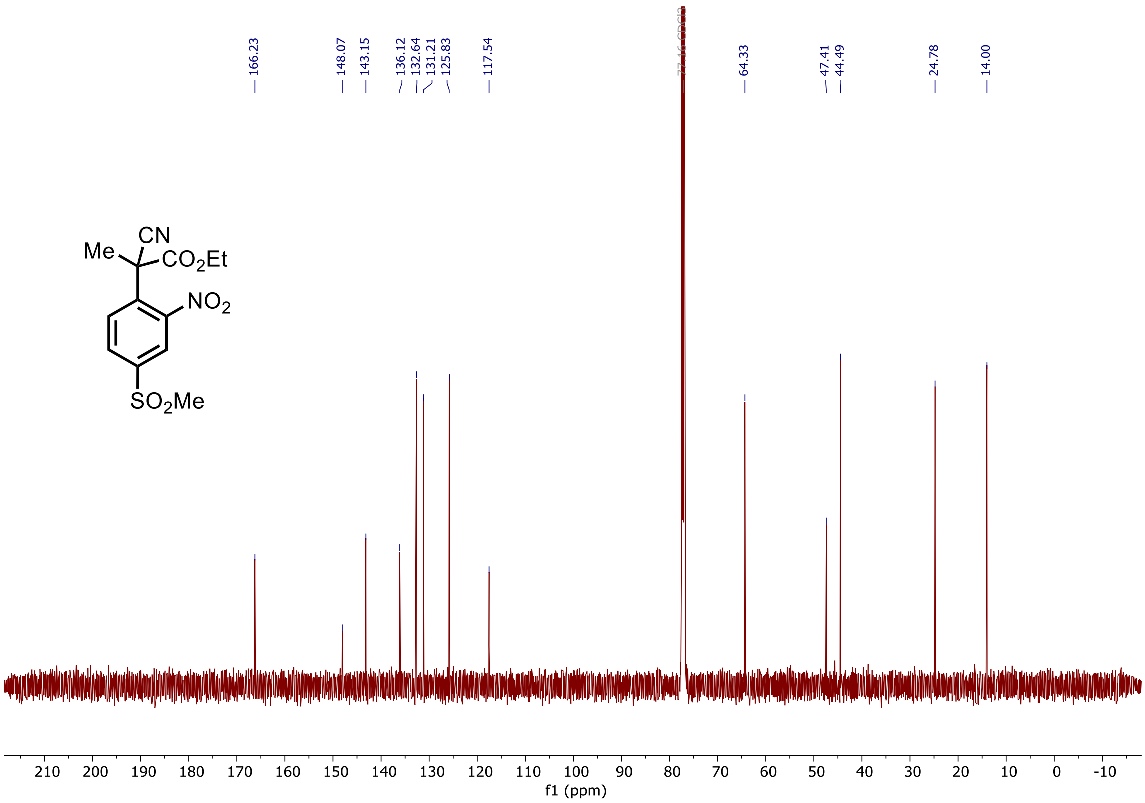

$^{13}C\{^1H\}$ NMR (126 MHz, $CDCl_3$) of **14**

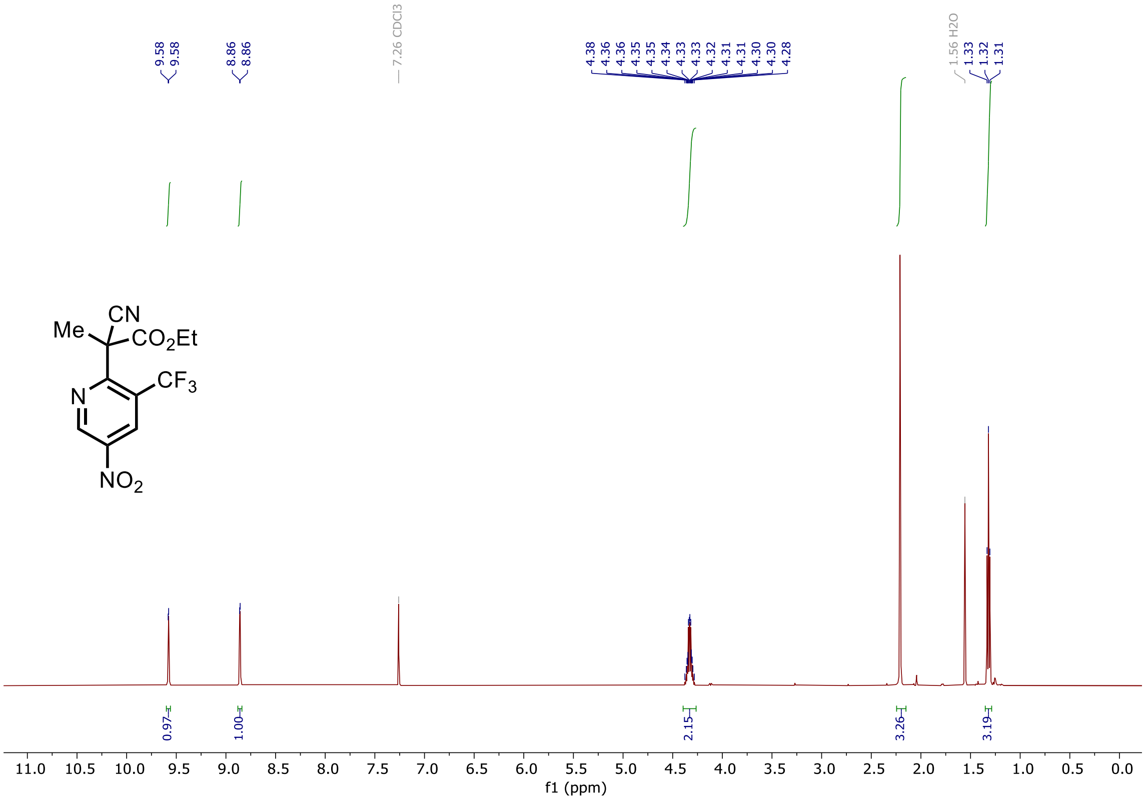

^1H NMR (500 MHz, $CDCl_3$) of **15**

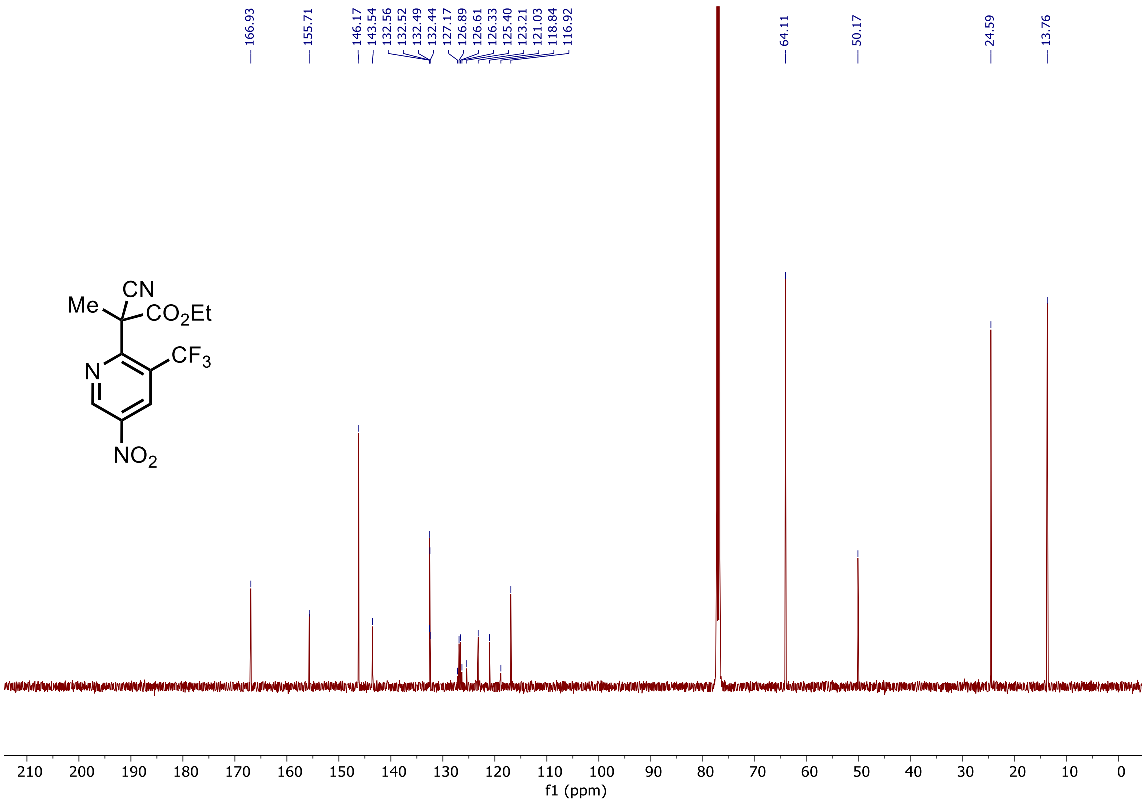

$^{13}C\{^{1}H\}$ NMR (126 MHz, $CDCl_3$) of **15**

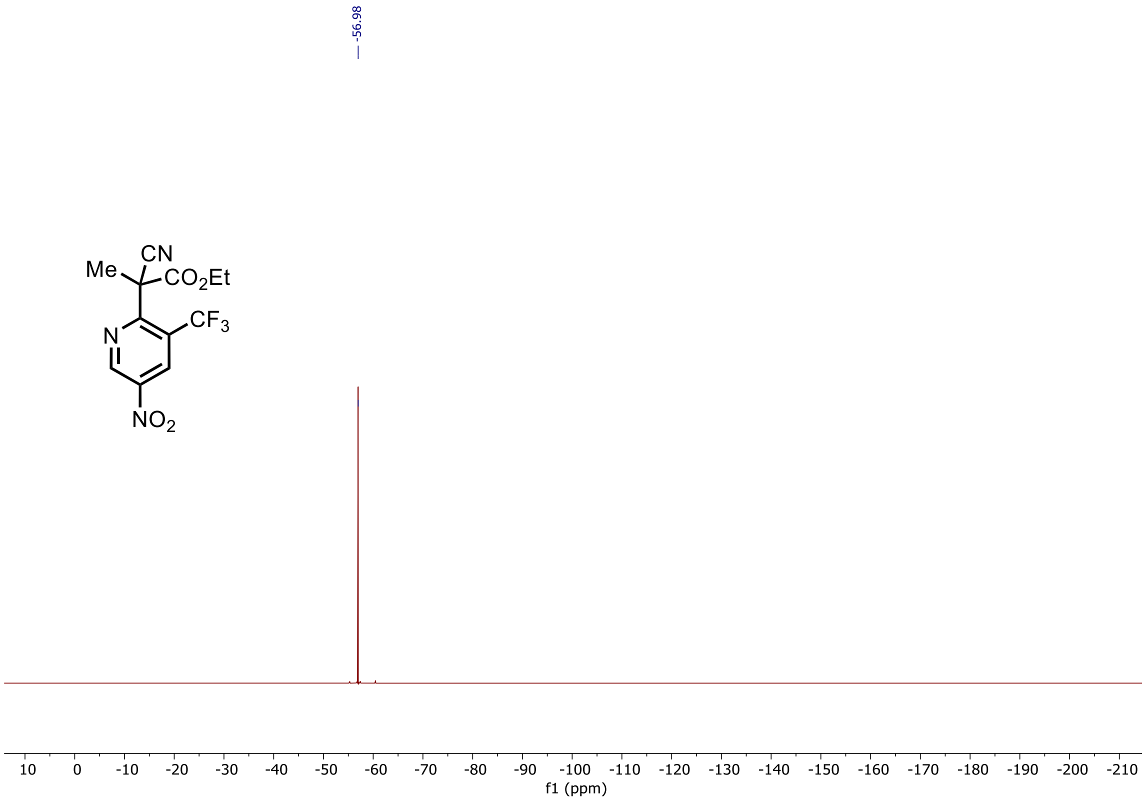

^{19}F NMR (471 MHz, $CDCl_3$) of **15**

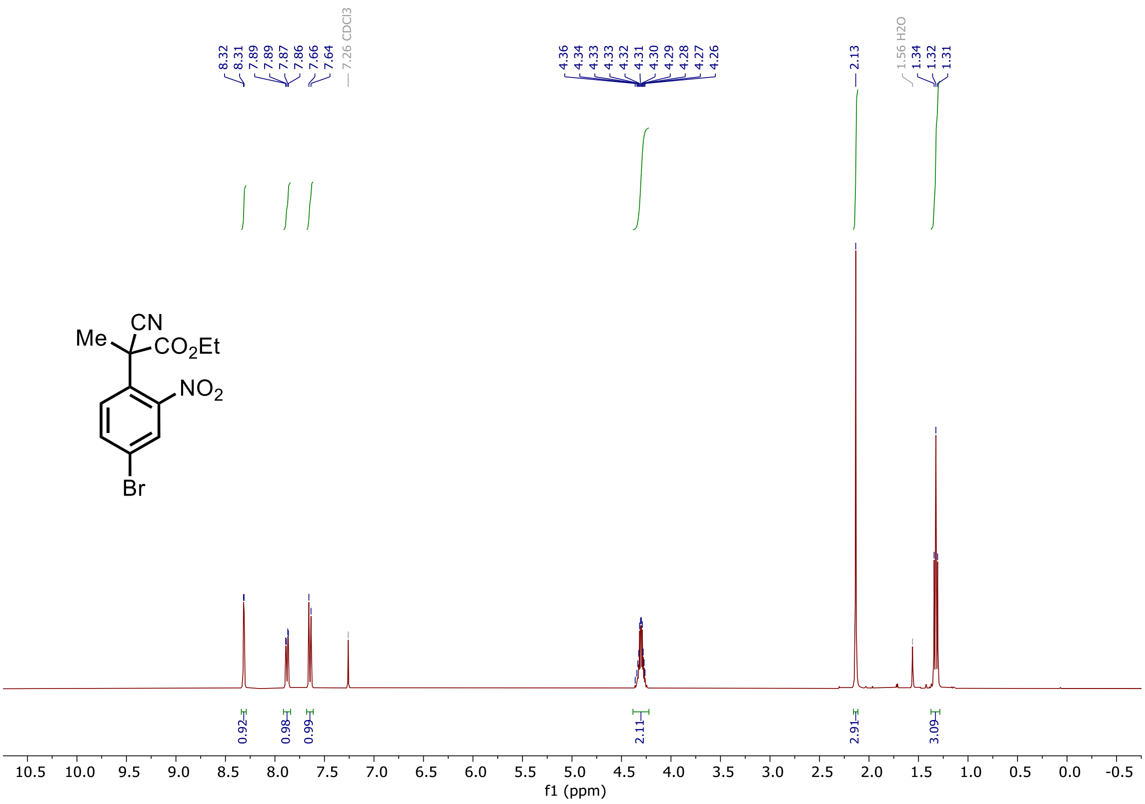

^{1}H NMR (400 MHz, $CDCl_3$) of **16**

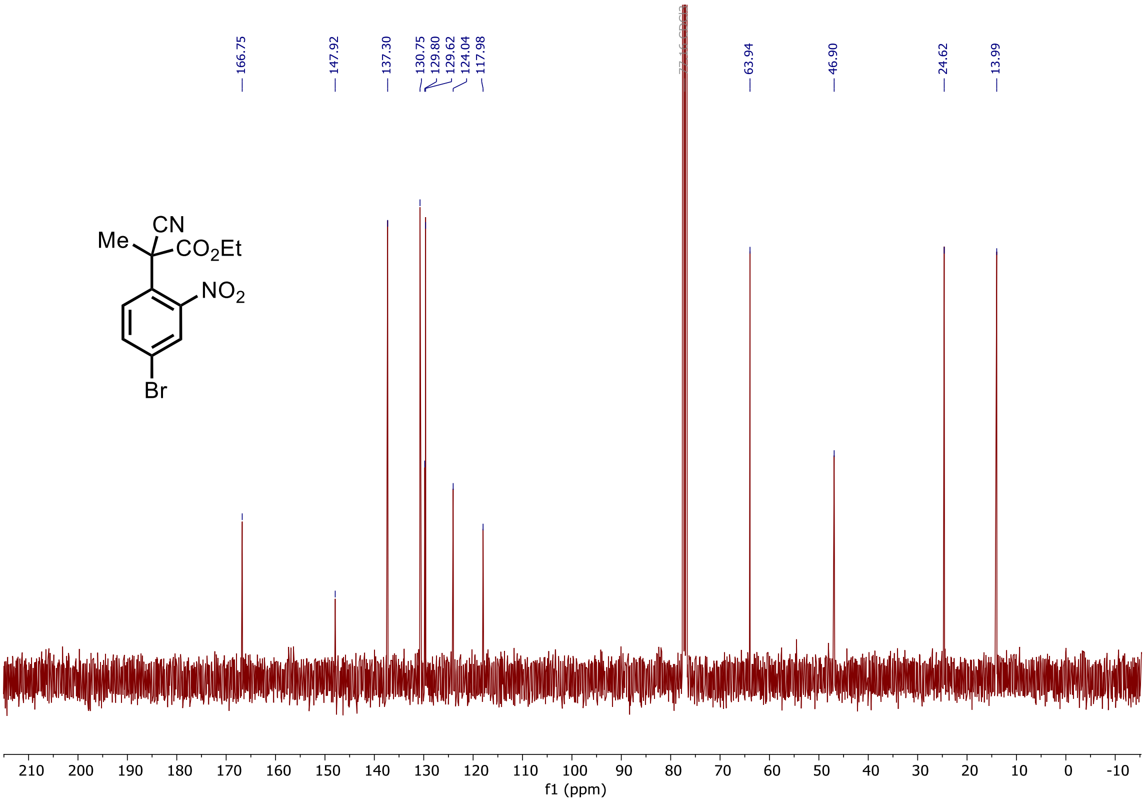

$^{13}C\{^{1}H\}$ NMR (101 MHz, $CDCl_3$) of **16**

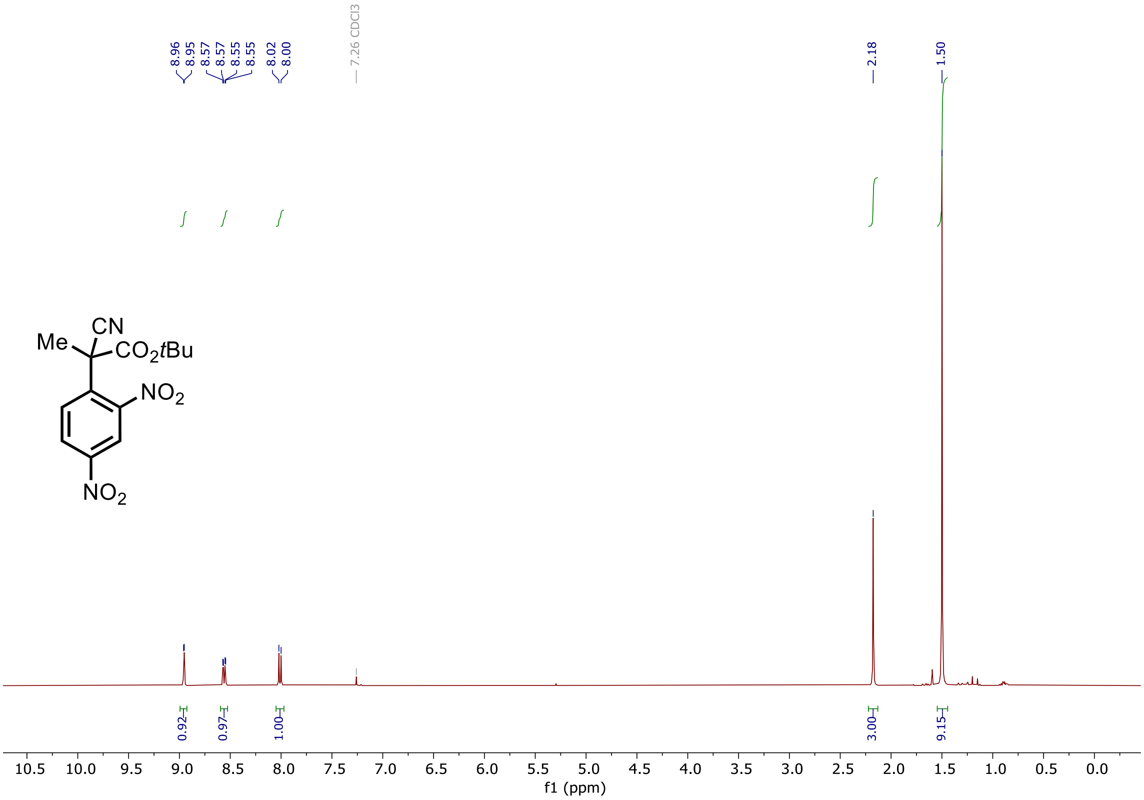

^{1}H NMR (500 MHz, CDCl$_3$) of **17**

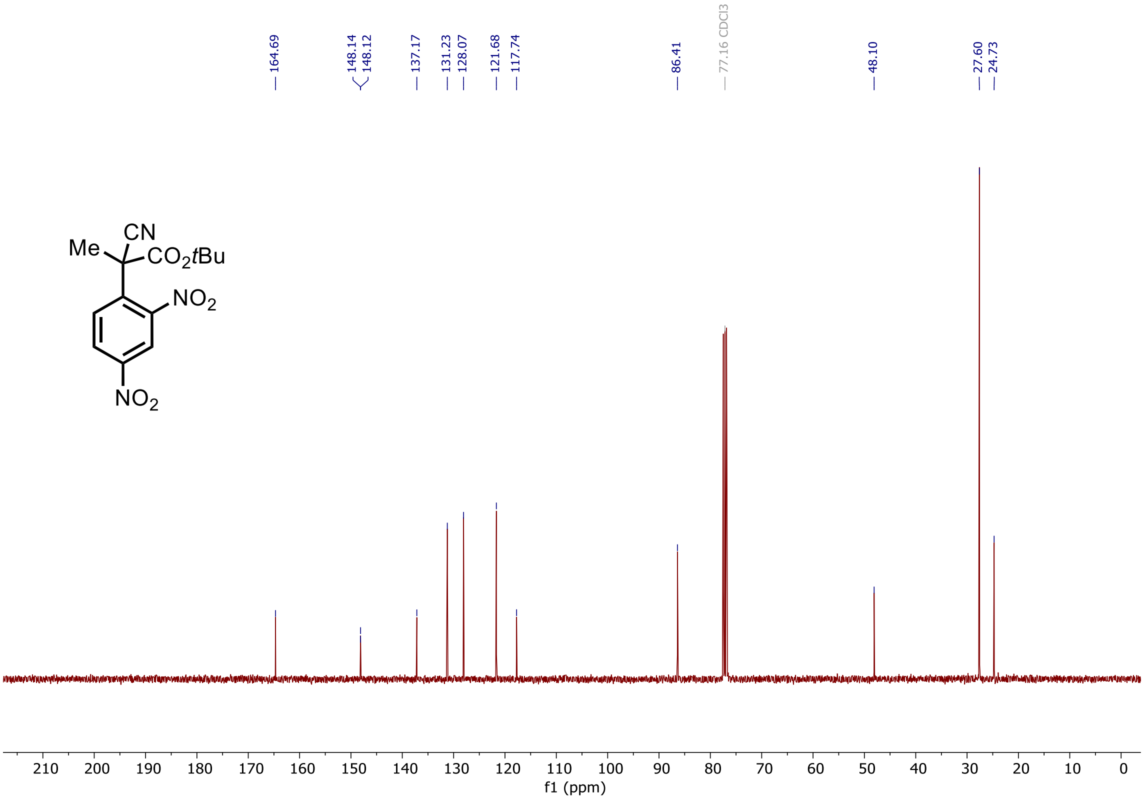

^{13}C{^{1}H} NMR (126 MHz, CDCl$_3$) of **17**

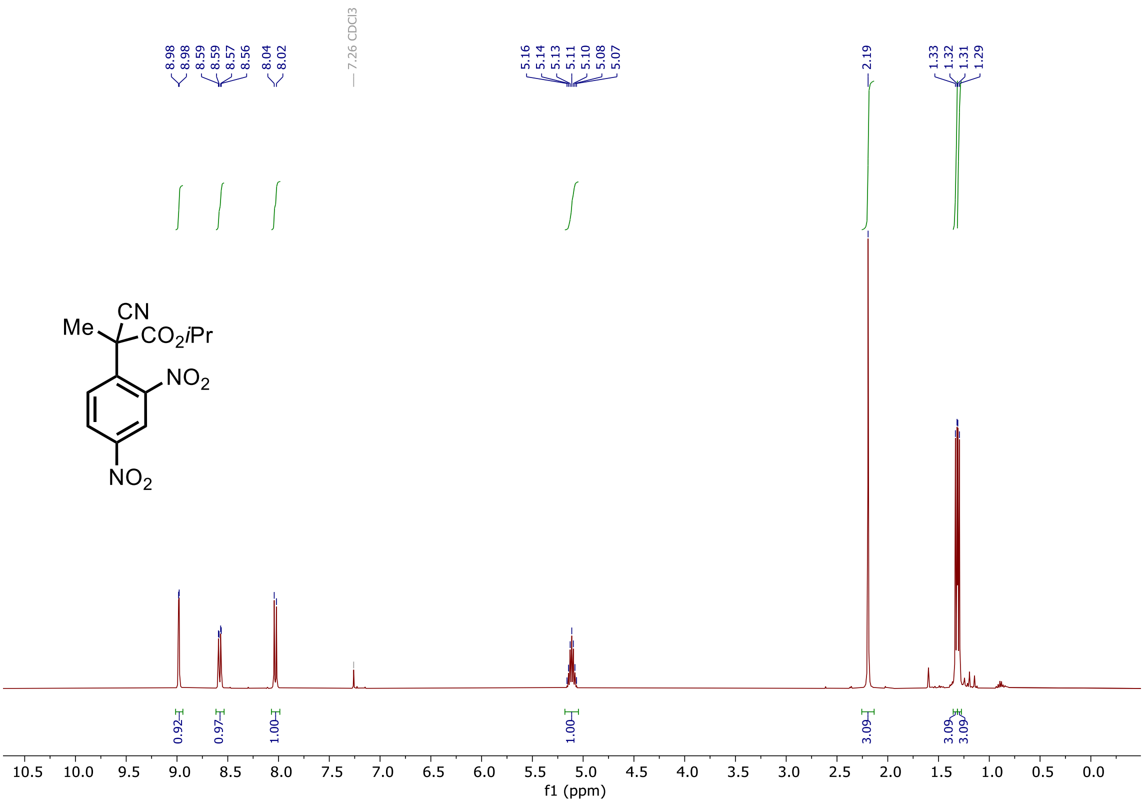

^{1}H NMR (400 MHz, $CDCl_3$) of **18**

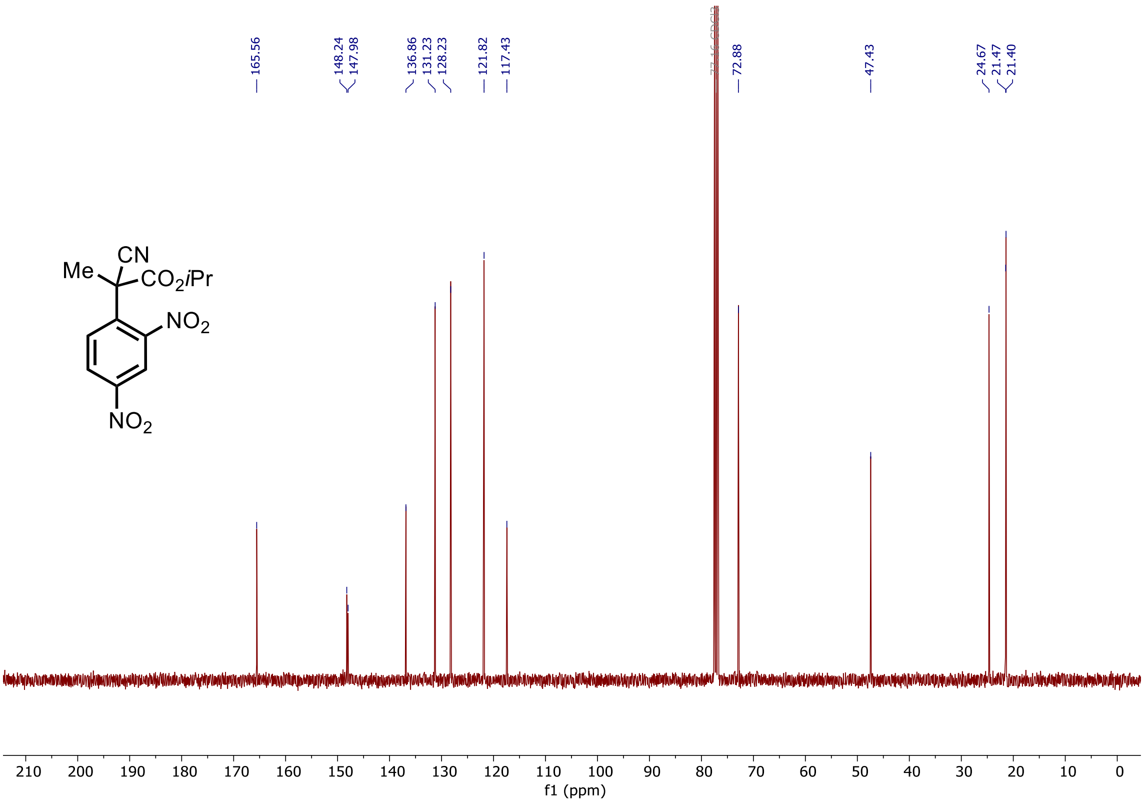

$^{13}C\{^1H\}$ NMR (101 MHz, $CDCl_3$) of **18**

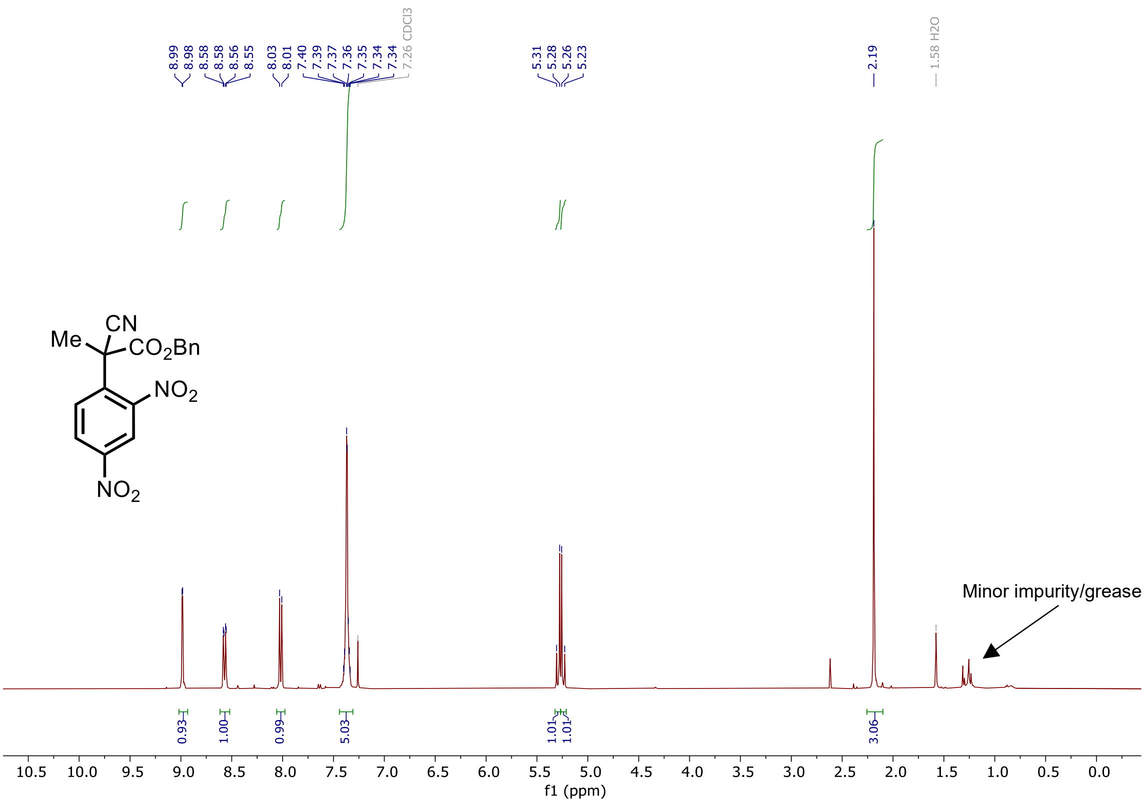

^{1}H NMR (400 MHz, $CDCl_3$) of **19**

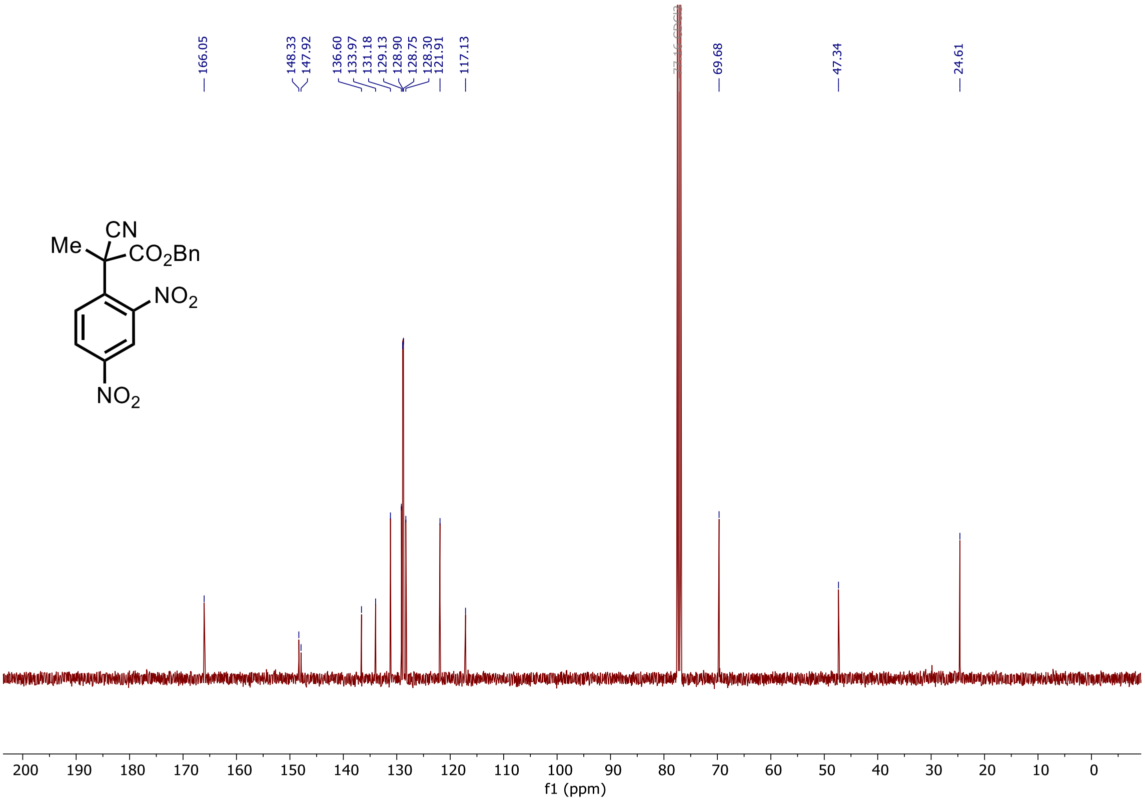

$^{13}C\{^1H\}$ NMR (101 MHz, $CDCl_3$) of **19**

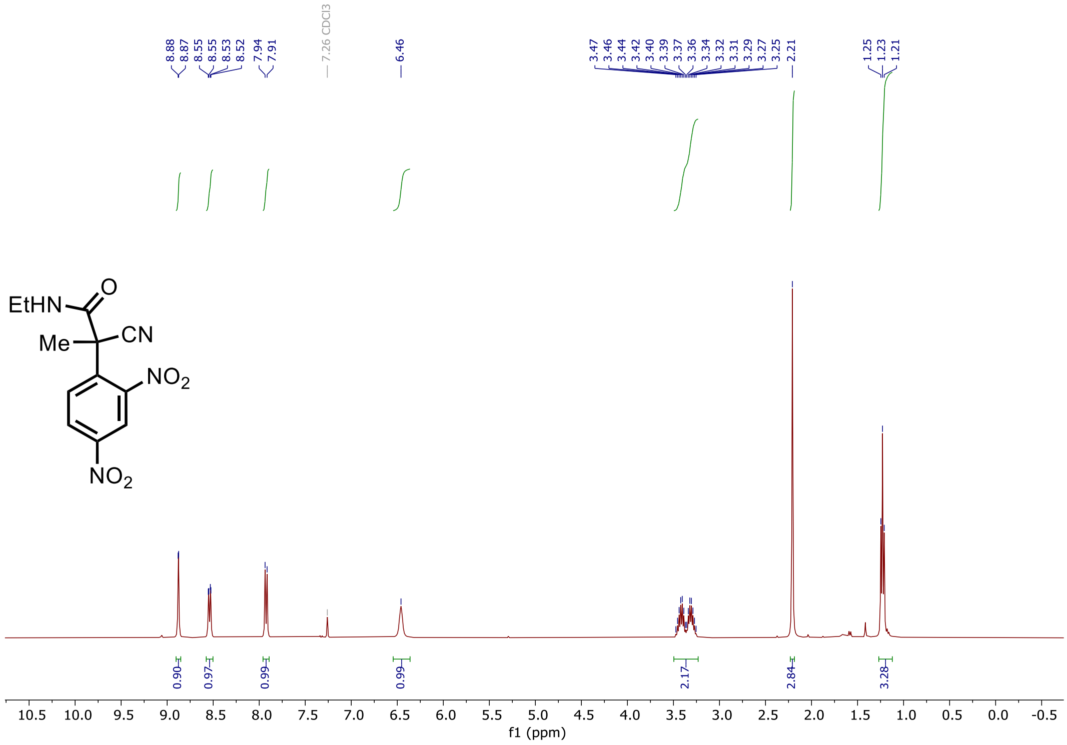

^{1}H NMR (400 MHz, $CDCl_3$) of **20**

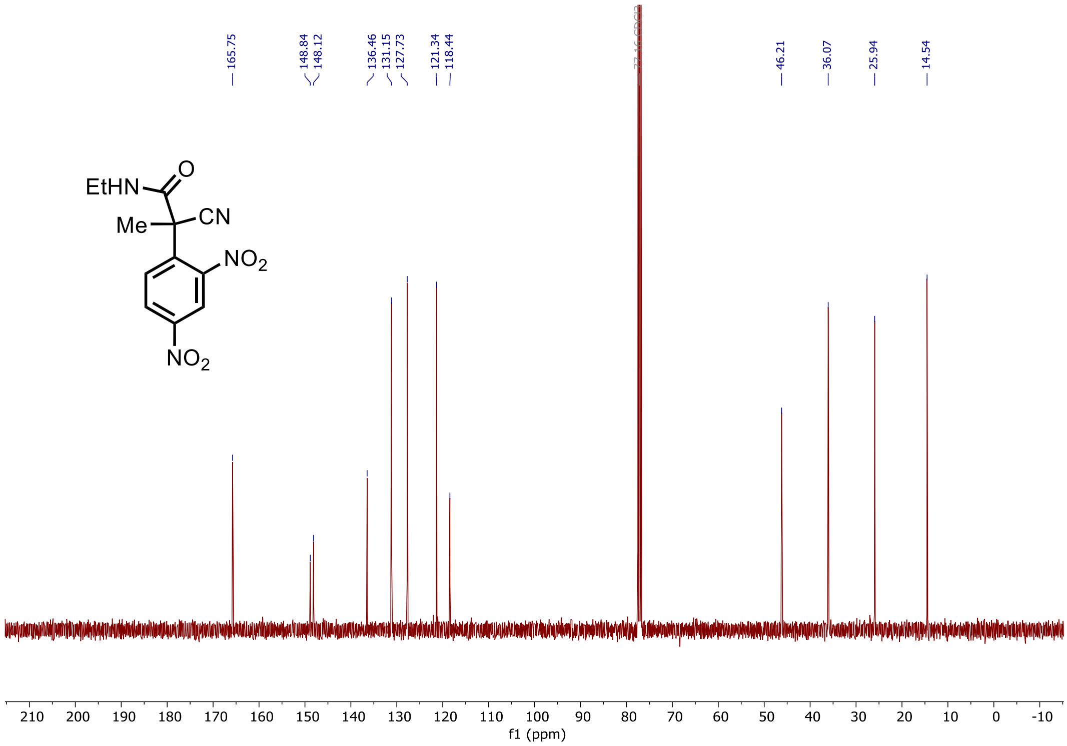

^{13}C NMR (101 MHz, $CDCl_3$) of **20**

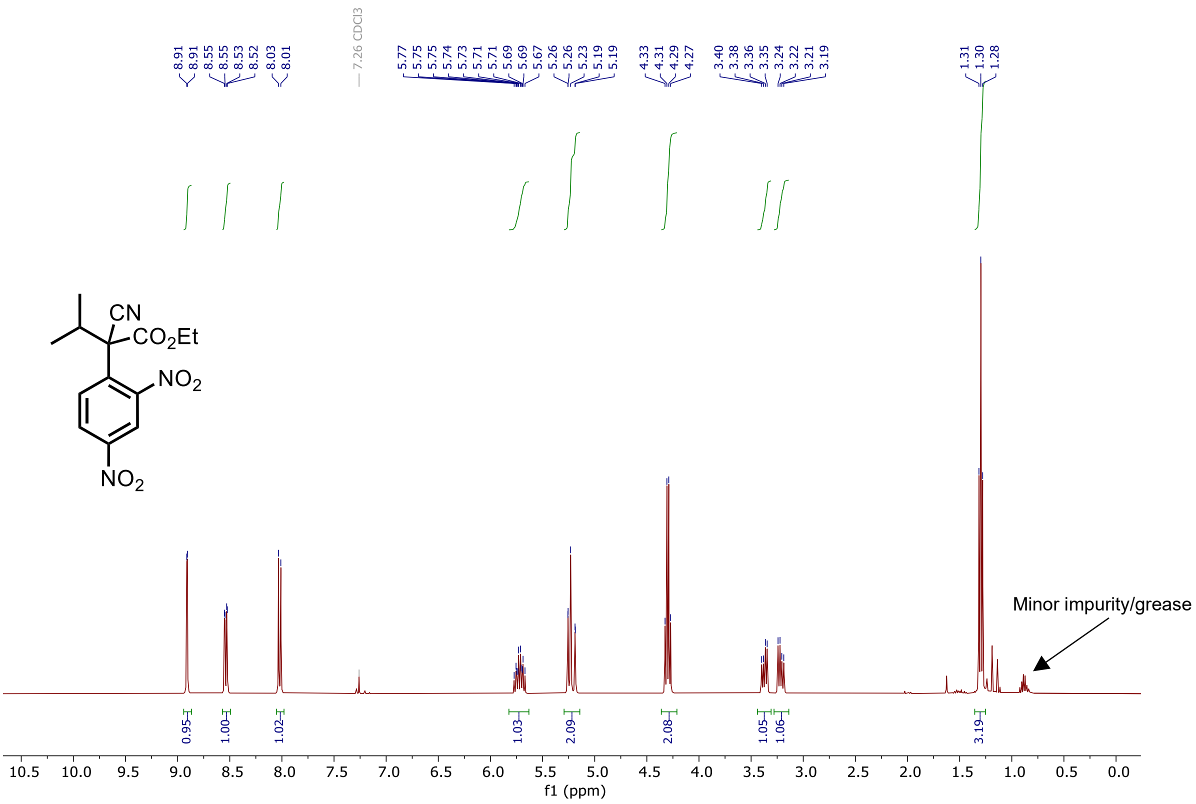

^{1}H NMR (400 MHz, $CDCl_3$) of **21**

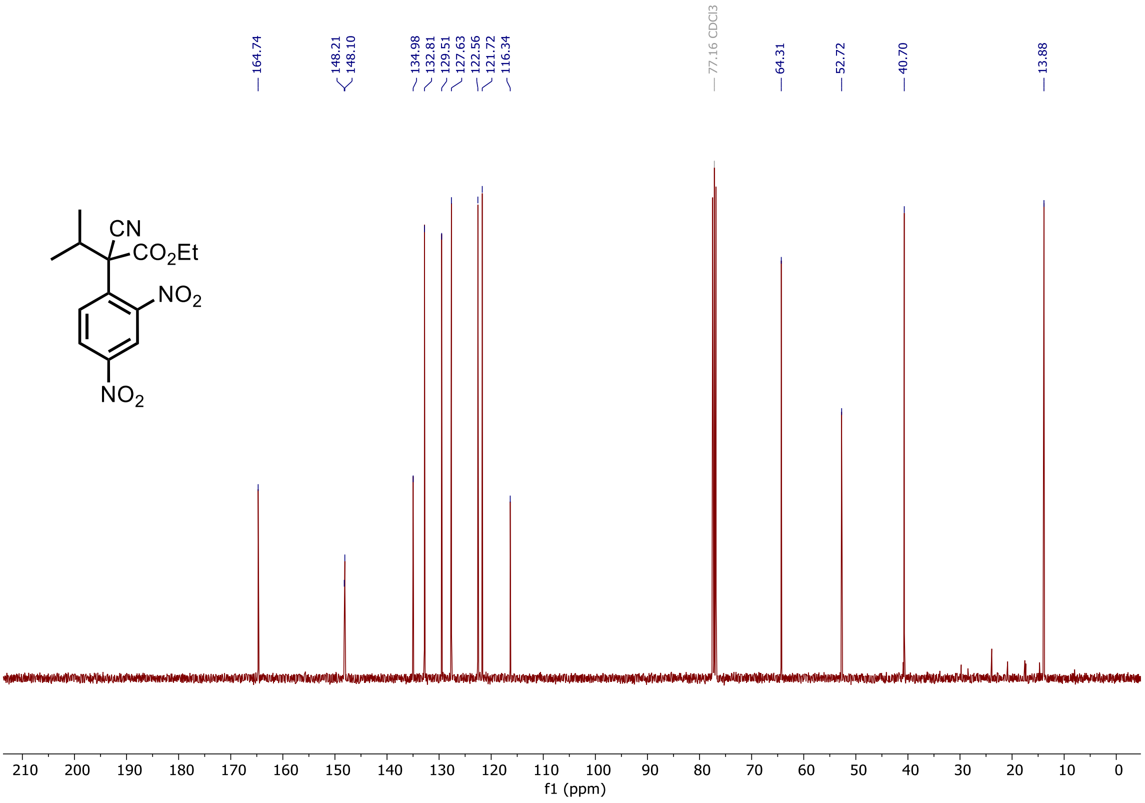

^{13}C{^{1}H} NMR (101 MHz, $CDCl_3$) of **21**

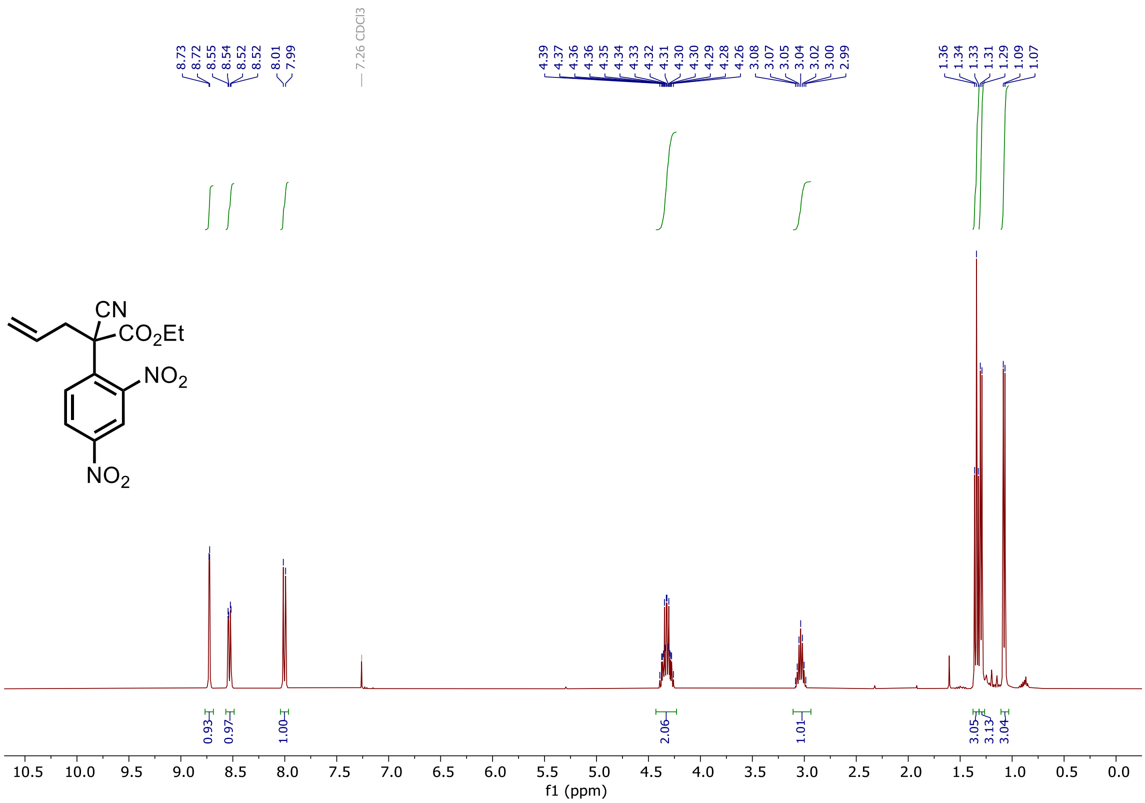

^{1}H NMR (400 MHz, $CDCl_3$) of **22**

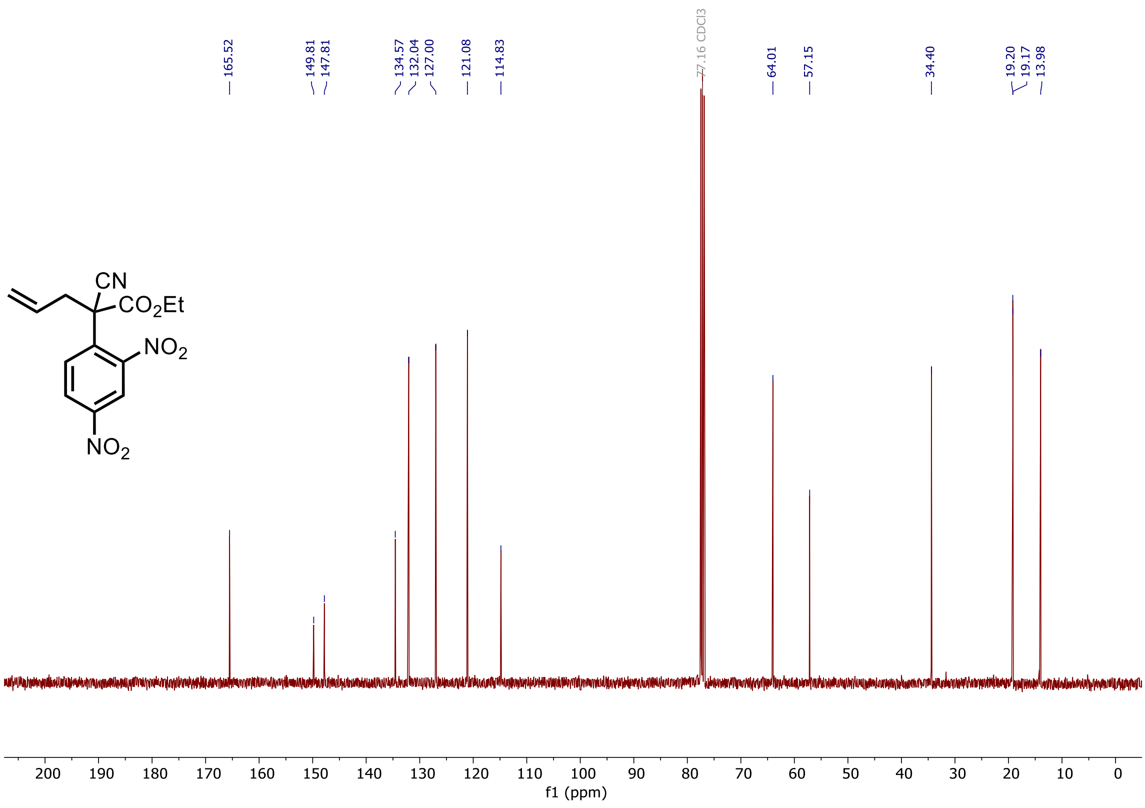

$^{13}C\{^1H\}$ NMR (101 MHz, $CDCl_3$) of **22**

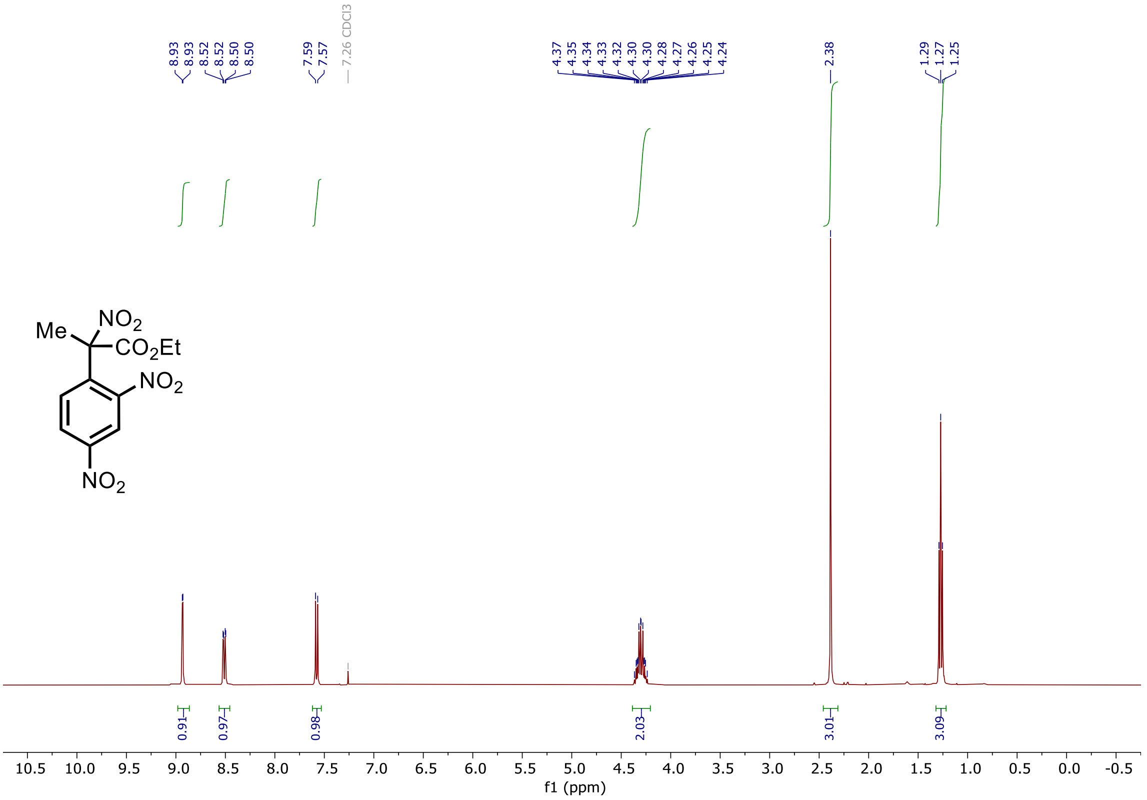

^{1}H NMR (400 MHz, $CDCl_3$) of **23**

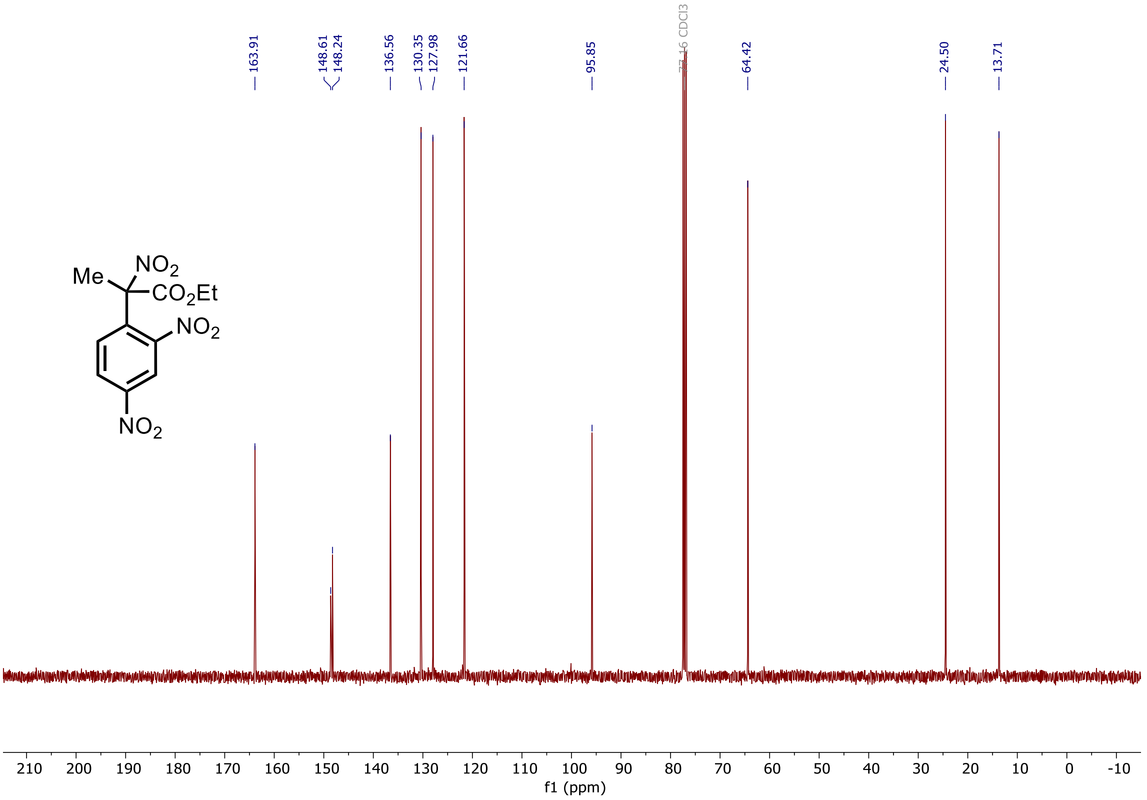

^{13}C{^{1}H} NMR (101 MHz, $CDCl_3$) of **23**

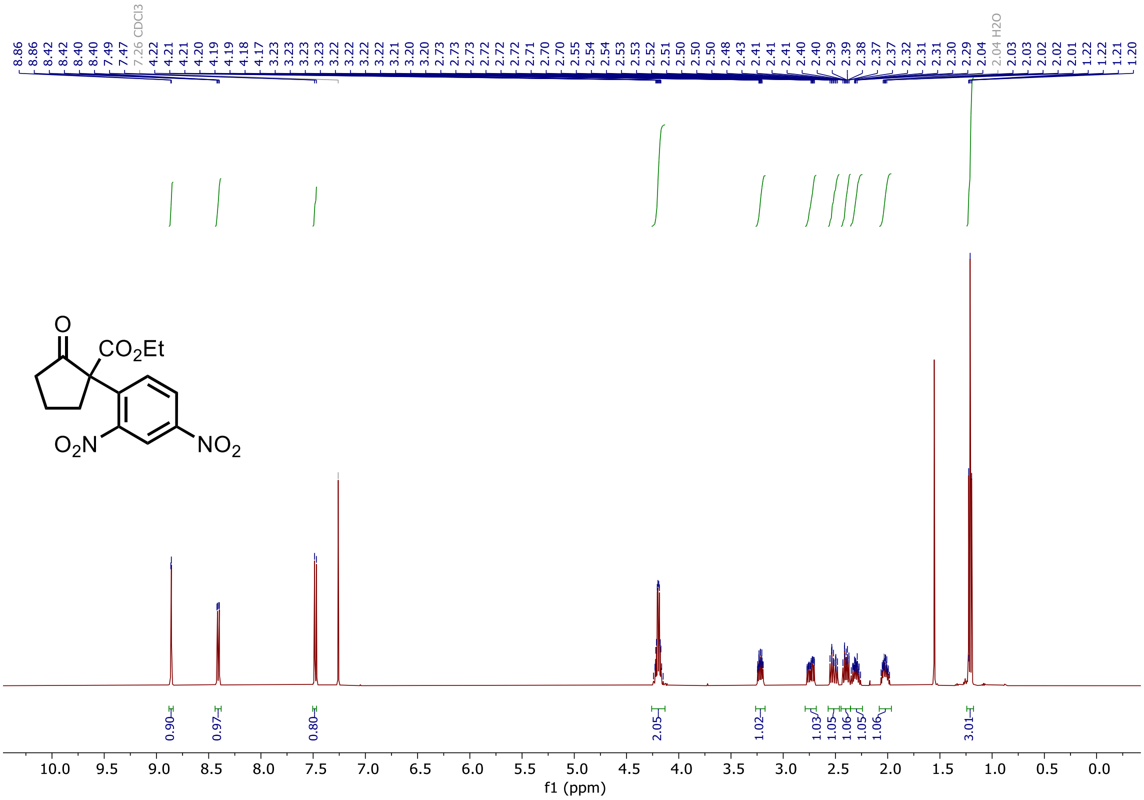

^{1}H NMR (500 MHz, $CDCl_3$) of **24a**

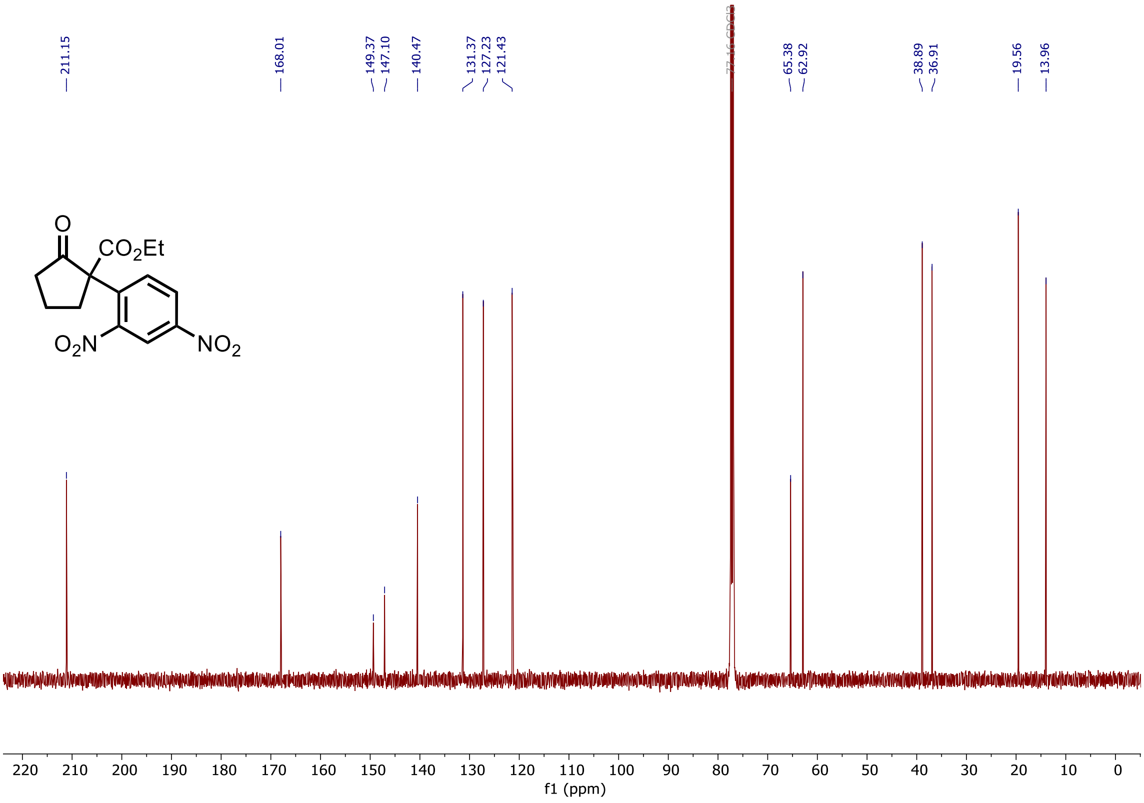

$^{13}C\{^{1}H\}$ NMR (101 MHz, $CDCl_3$) of **24a**

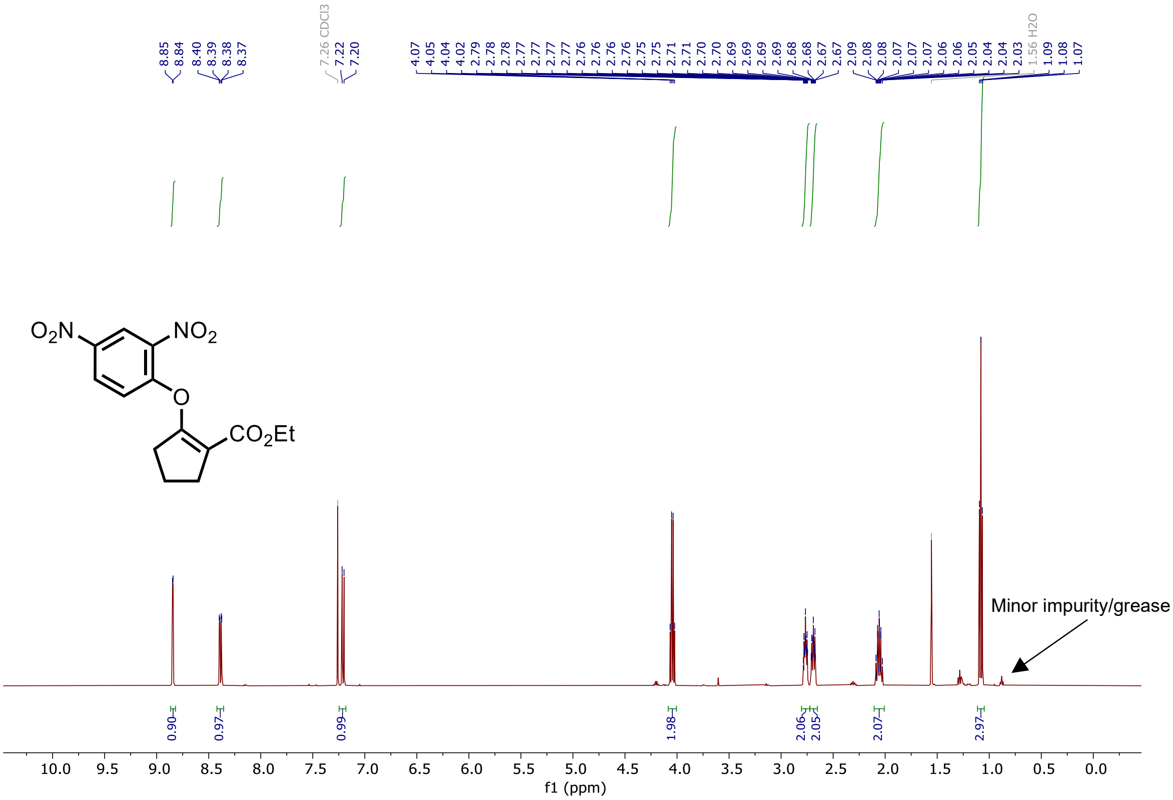

^{1}H NMR (500 MHz, $CDCl_3$) of **24b**

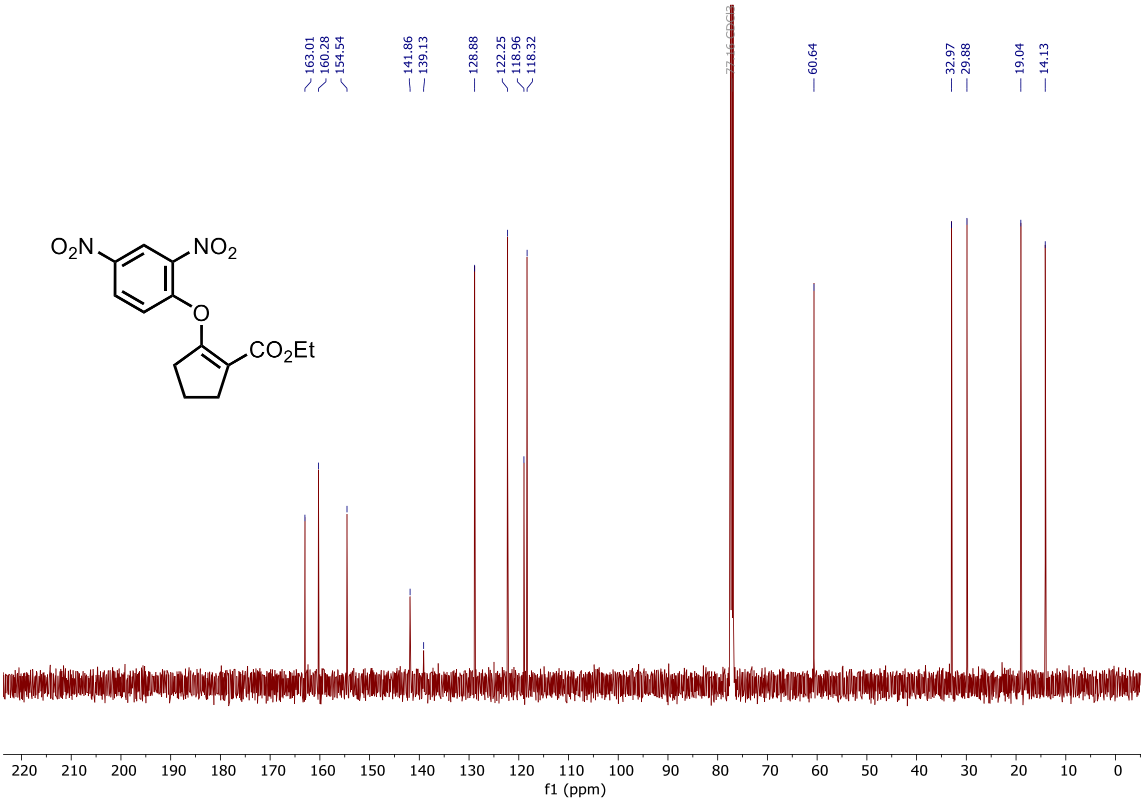

^{13}C{^{1}H} NMR (101 MHz, $CDCl_3$) of **24b**

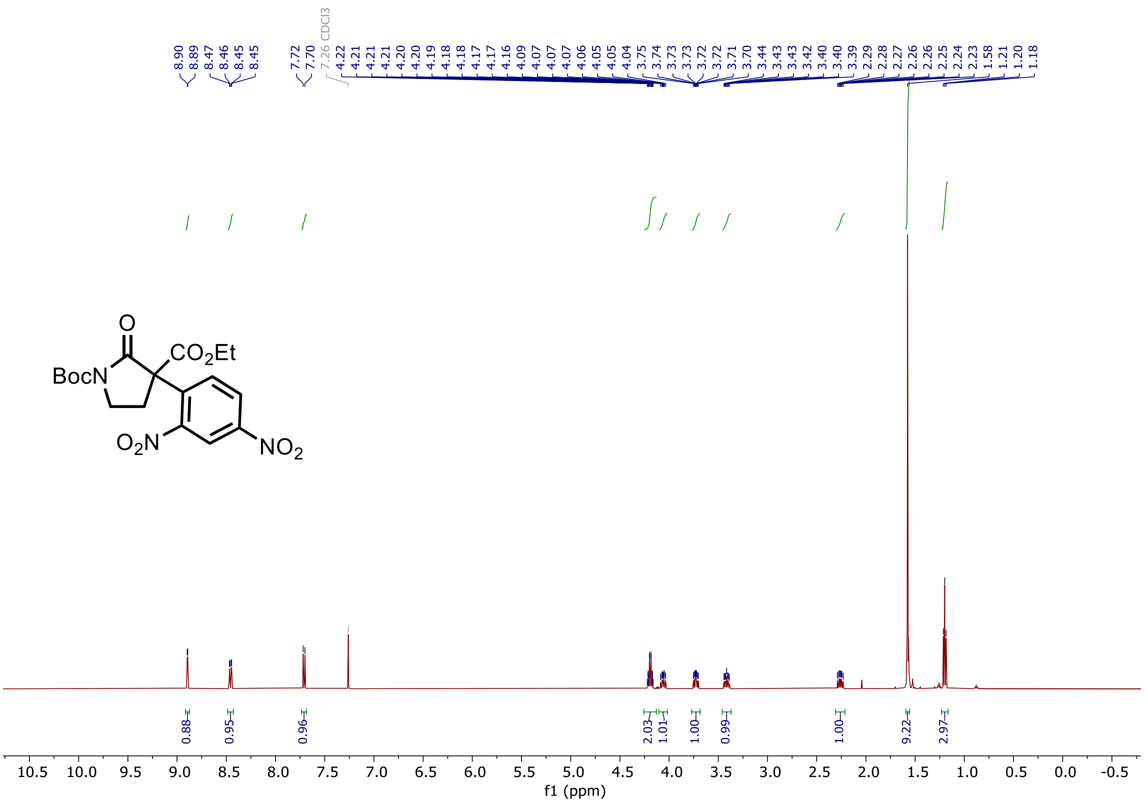

^{1}H NMR (500 MHz, $CDCl_3$) of **25**

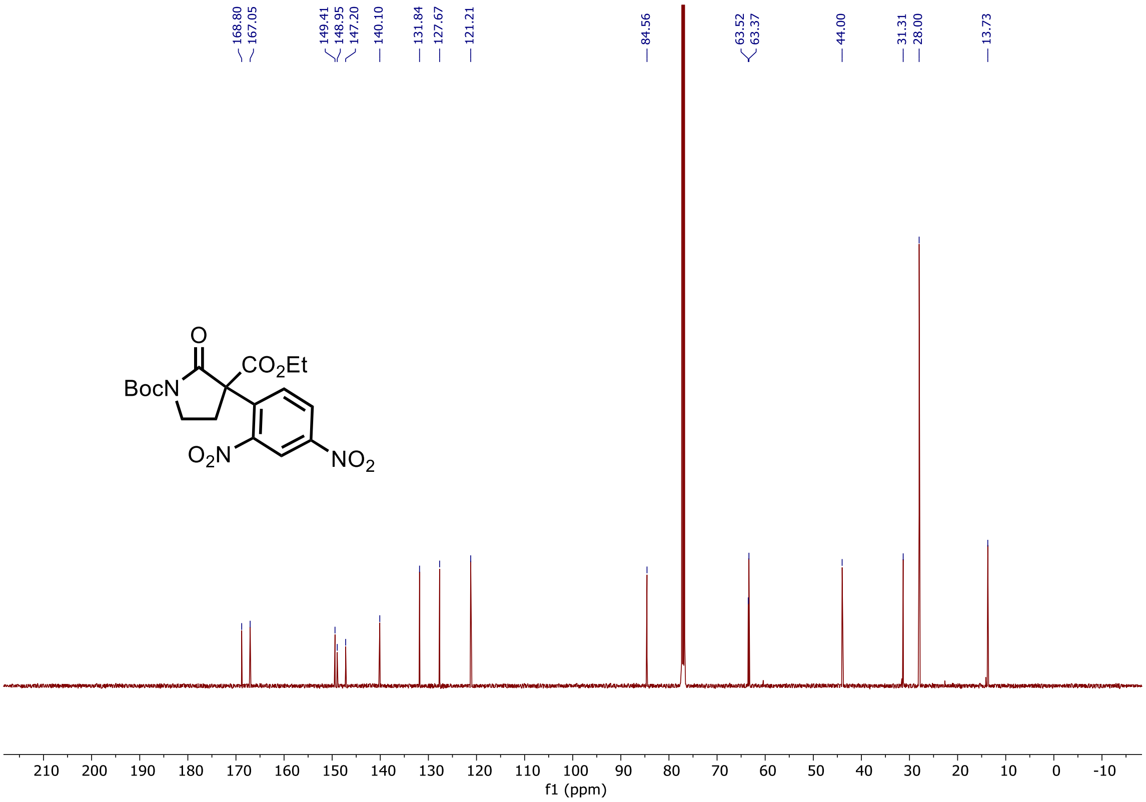

$^{13}C\{^{1}H\}$ NMR (126 MHz, $CDCl_3$) of **25**

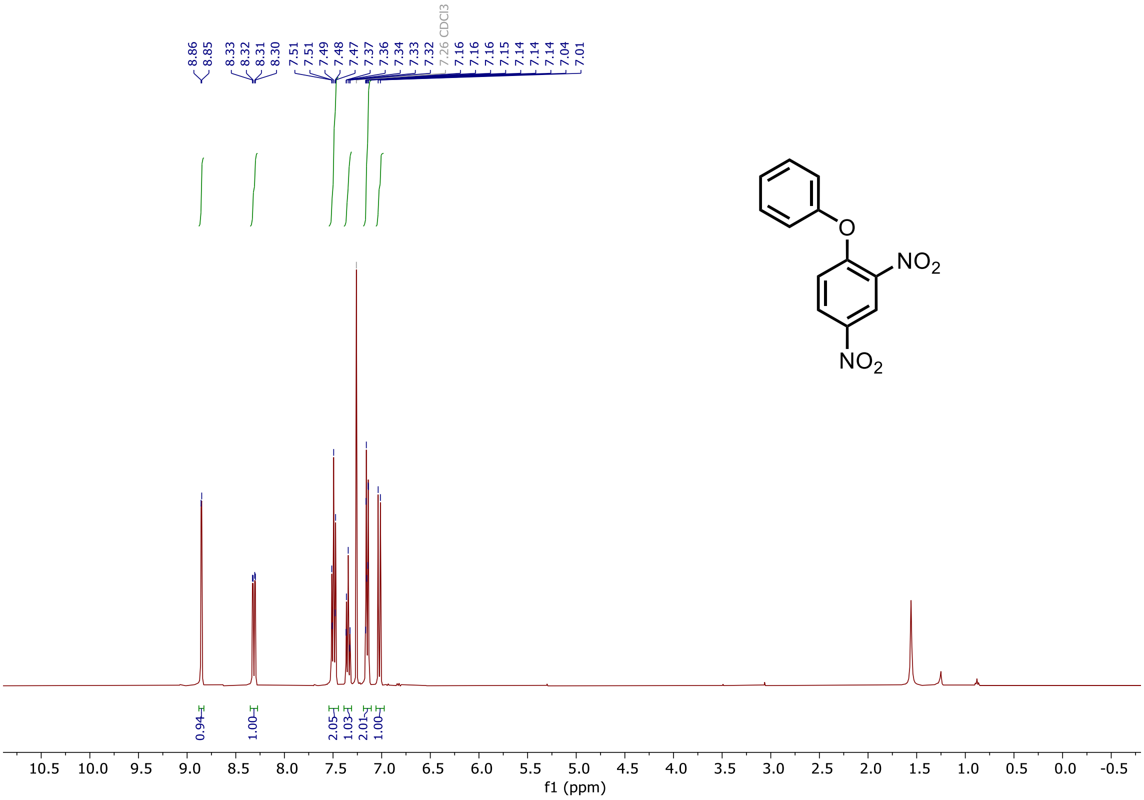

^{1}H NMR (400 MHz, $CDCl_3$) of **26**

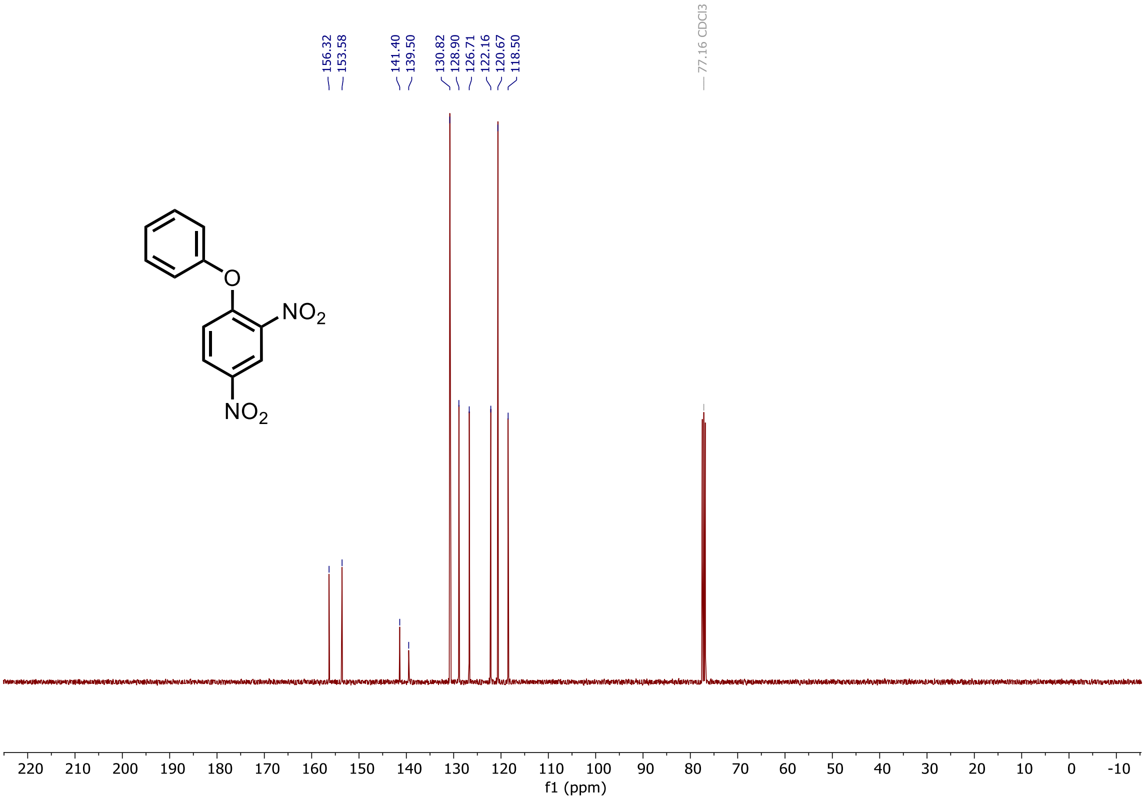

$^{13}C\{^{1}H\}$ NMR (101 MHz, $CDCl_3$) of **26**

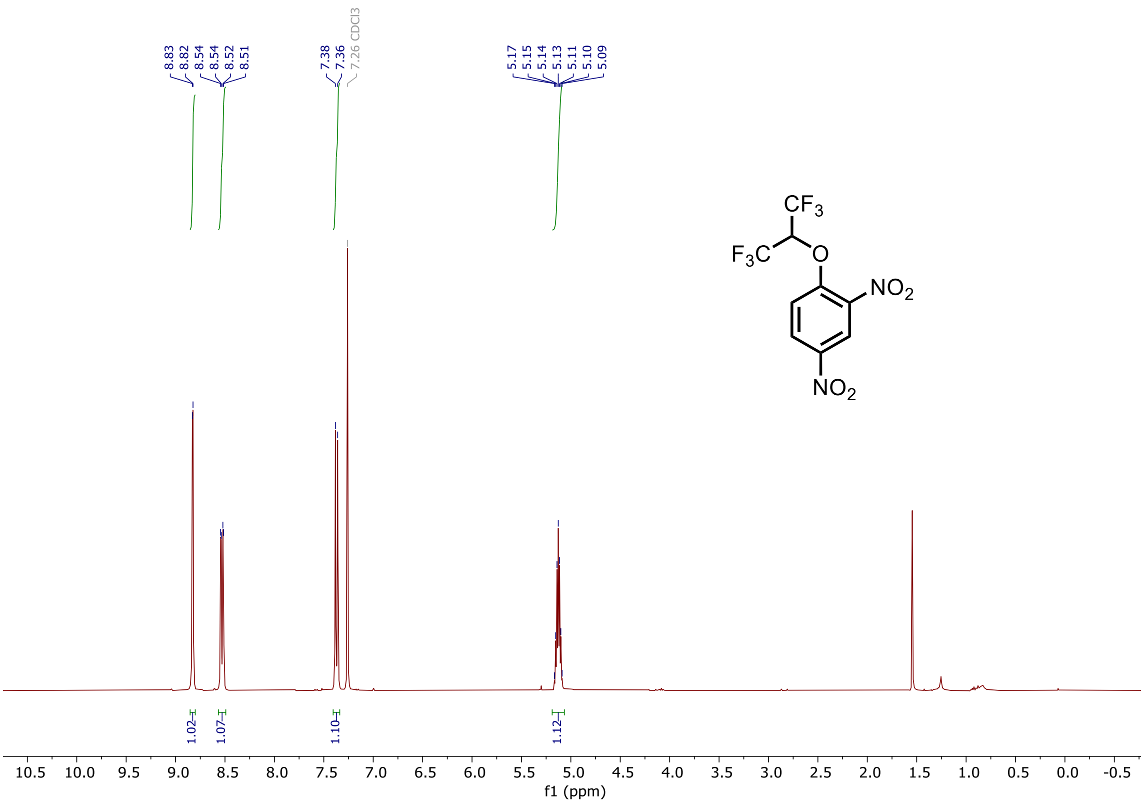

^{1}H NMR (400 MHz, $CDCl_3$) of **27**

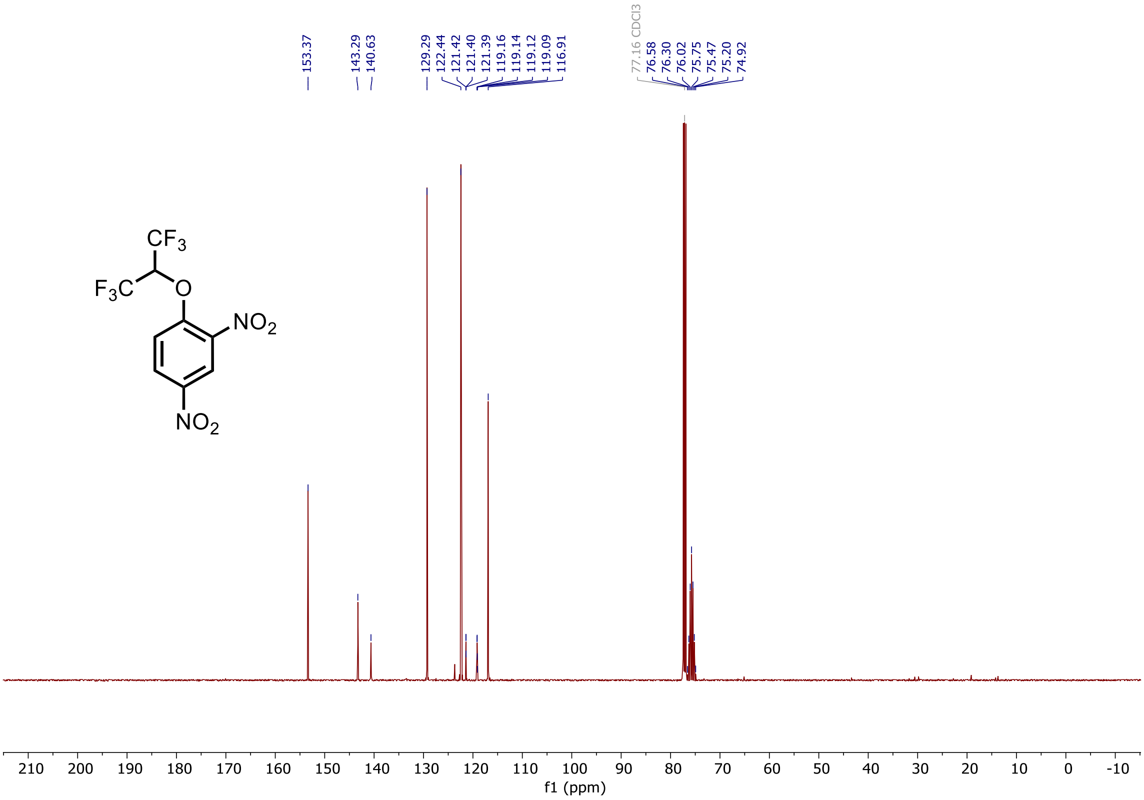

^{13}C{^{1}H} NMR (126 MHz, $CDCl_3$) of **27**

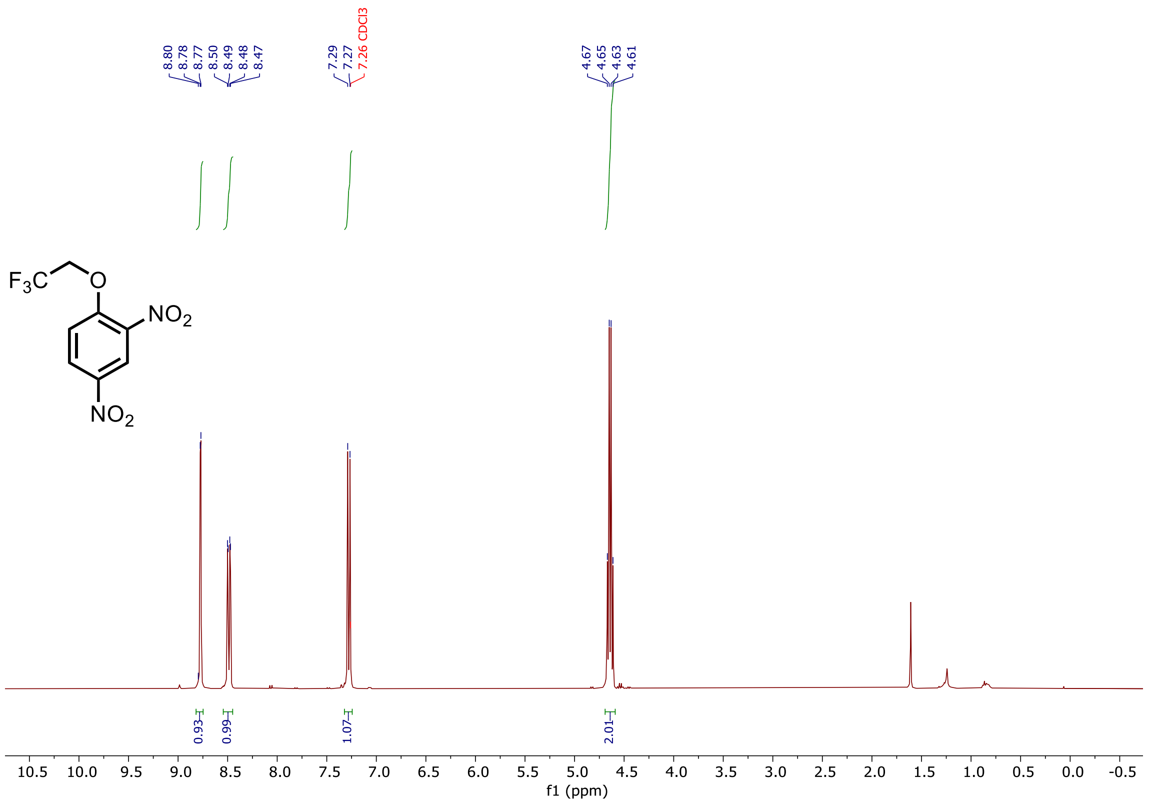

^{1}H NMR (400 MHz, $CDCl_3$) of **28**

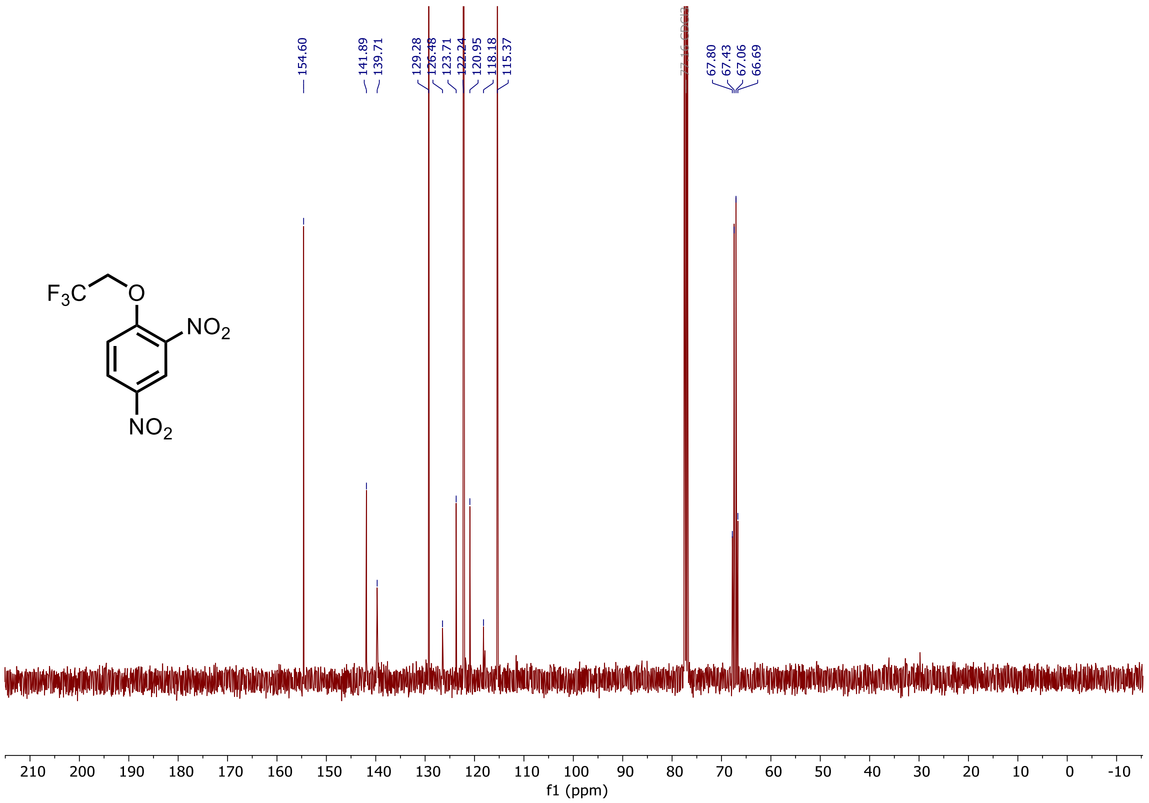

$^{13}C\{^{1}H\}$ NMR (126 MHz, $CDCl_3$) of **28**

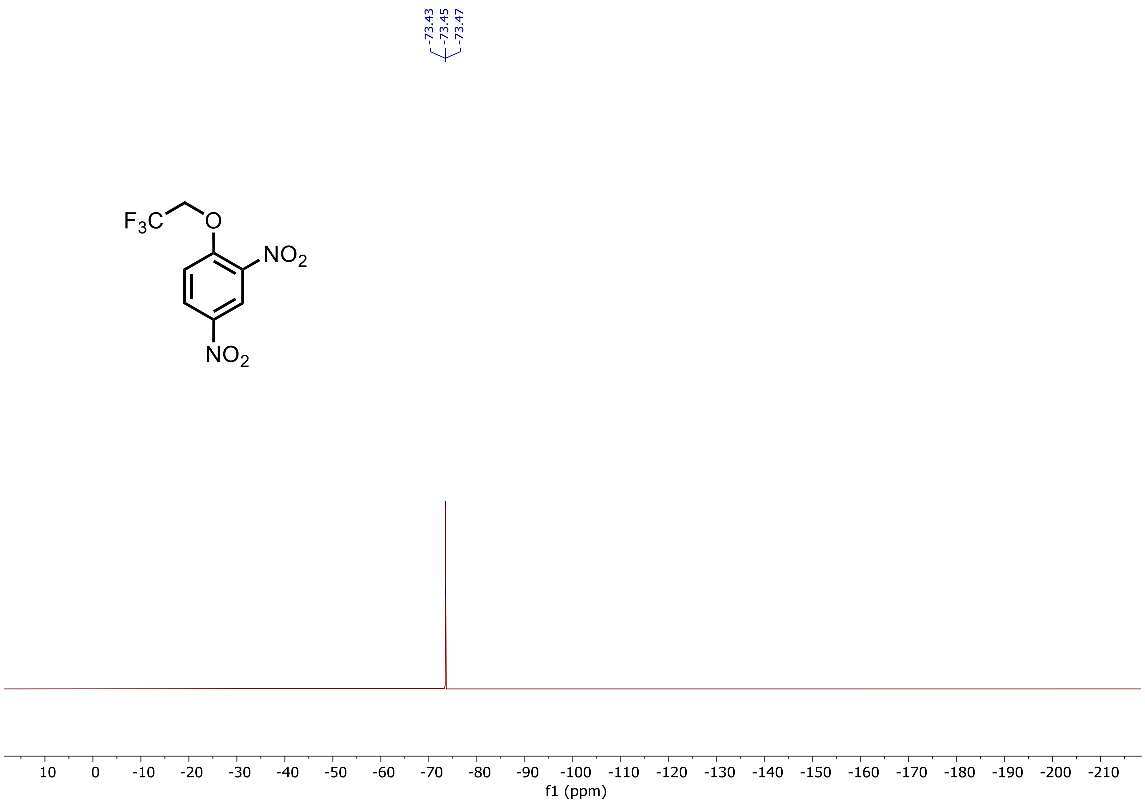

^{19}F NMR (376 MHz, $CDCl_3$) of **28**

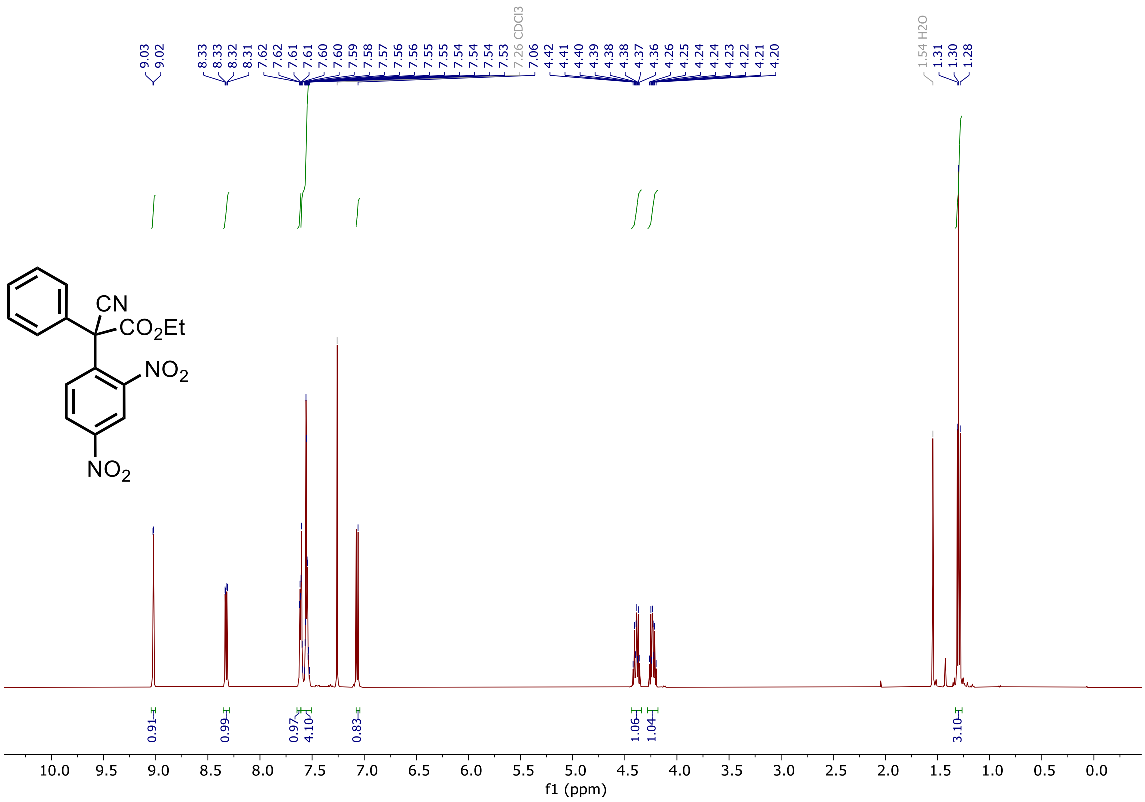

^{1}H NMR (500 MHz, $CDCl_3$) of **30**

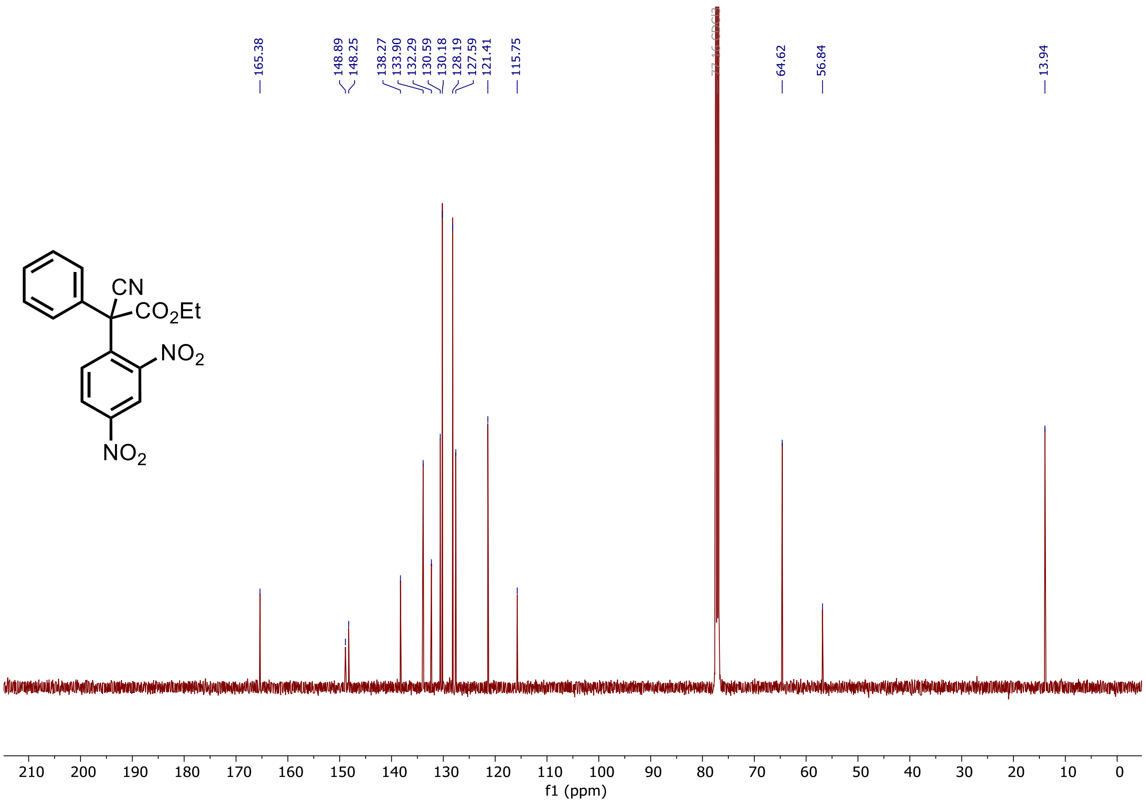

$^{13}C\{^1H\}$ NMR (101 MHz, $CDCl_3$) of **30**

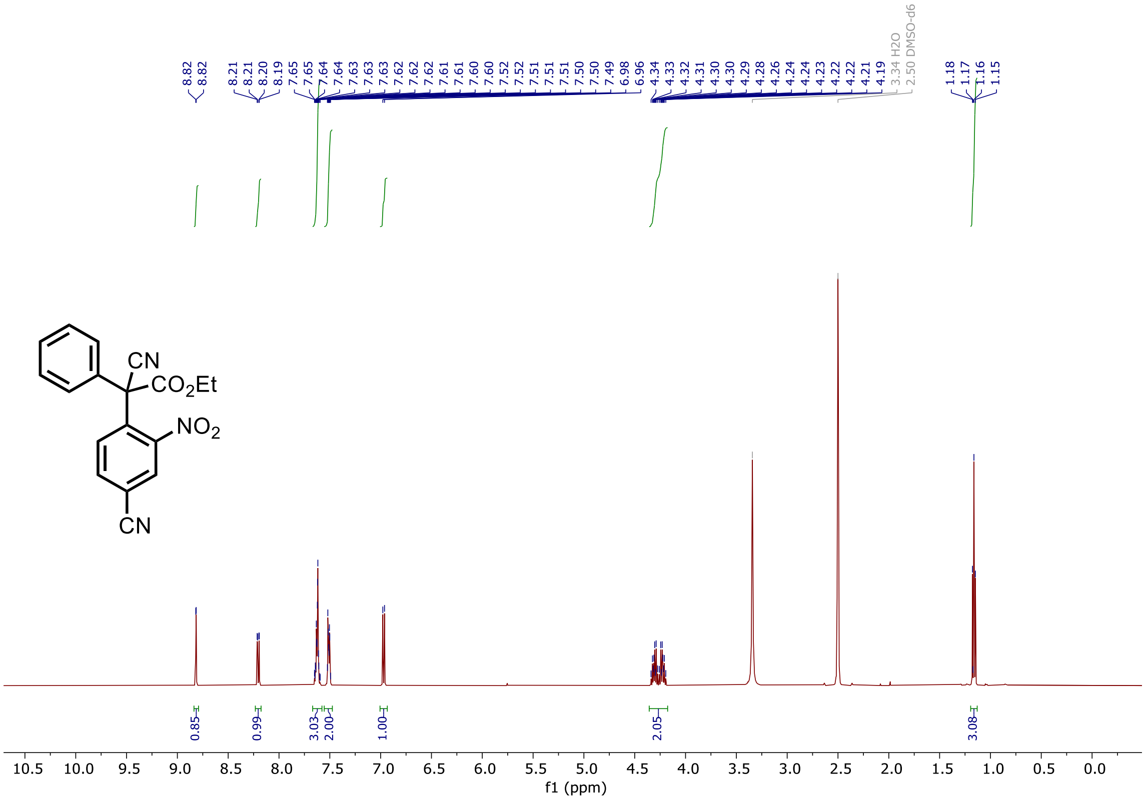

^1H NMR (500 MHz, $CDCl_3$) of **31**

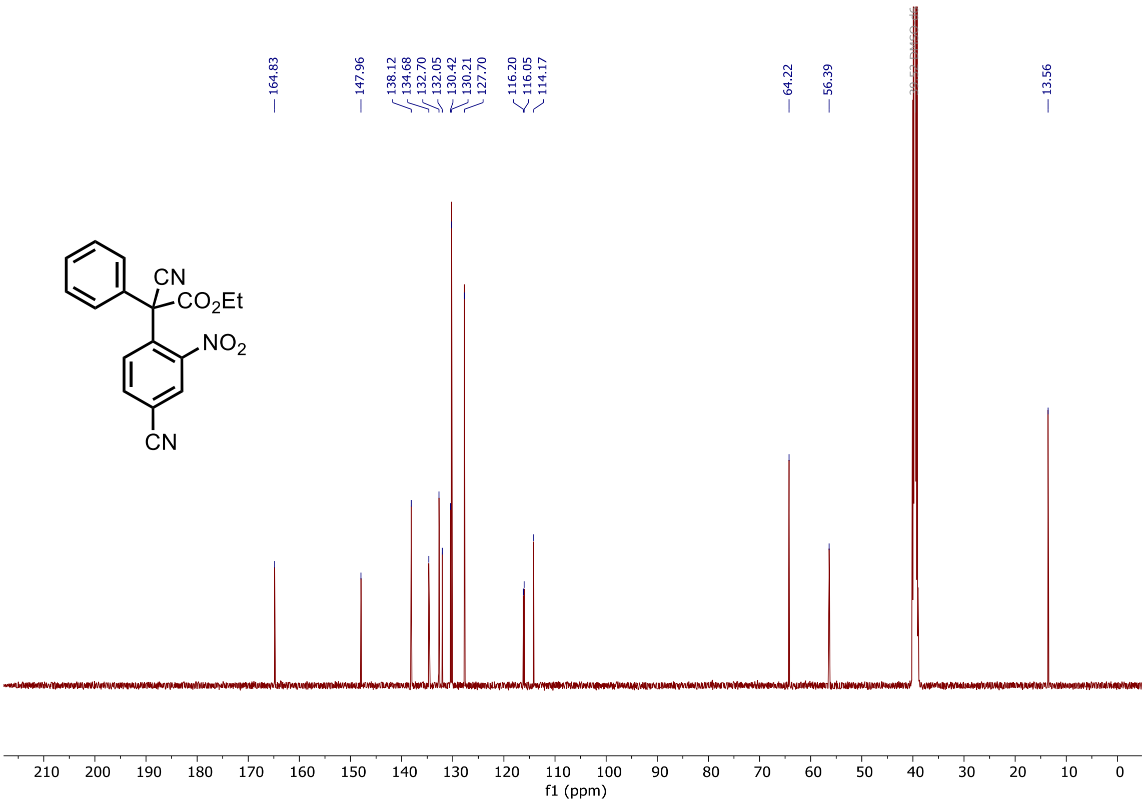

$^{13}C\{^1H\}$ NMR (101 MHz, $CDCl_3$) of **31**

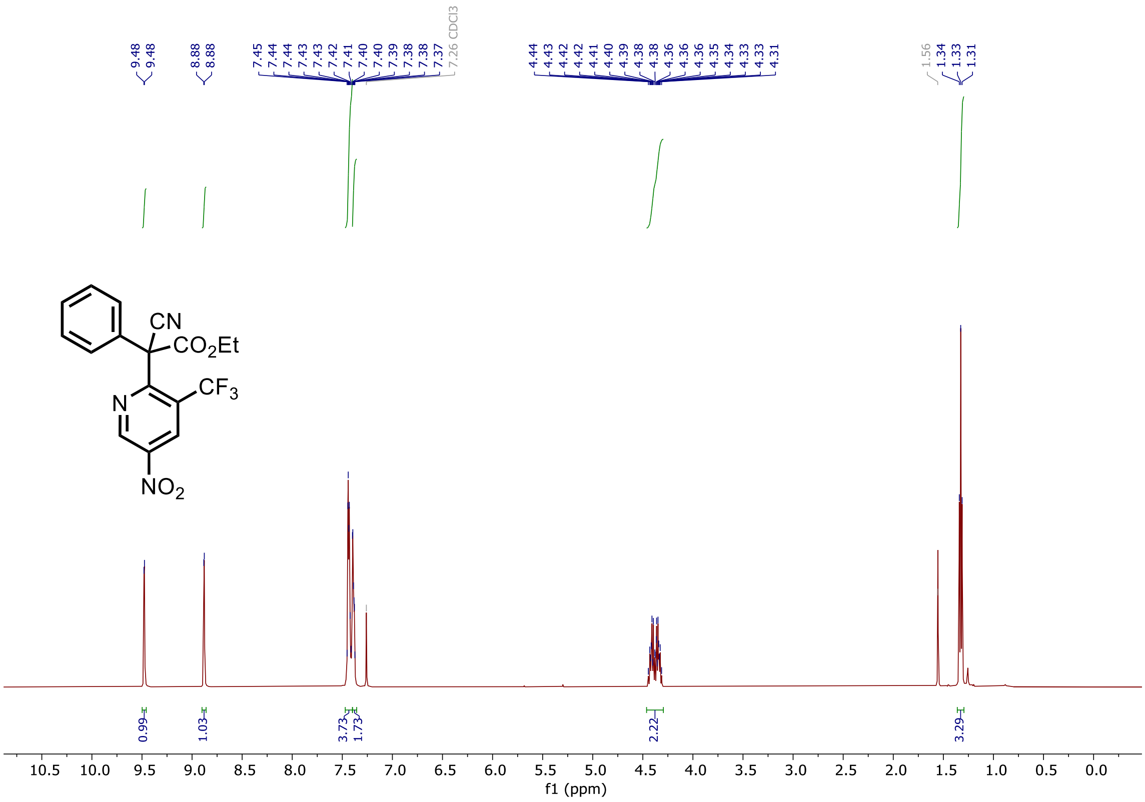

^1H NMR (500 MHz, $CDCl_3$) of **32**

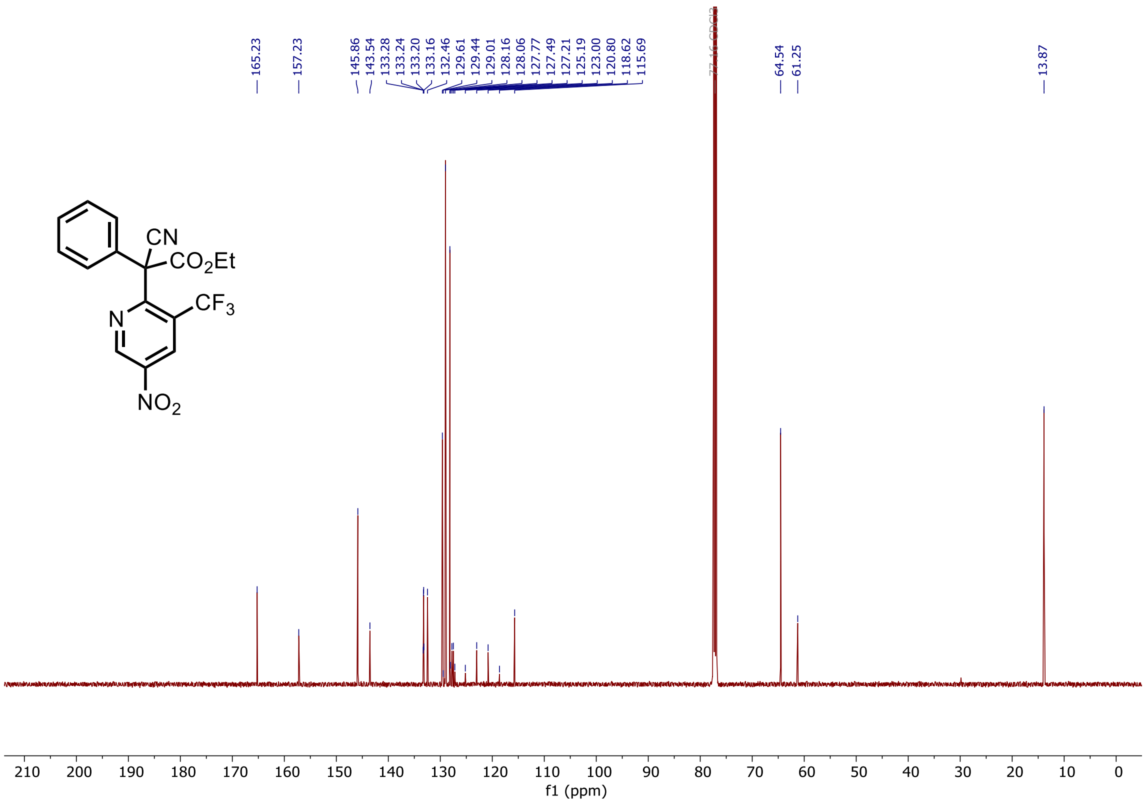

$^{13}C\{^{1}H\}$ NMR (101 MHz, $CDCl_3$) of **32**

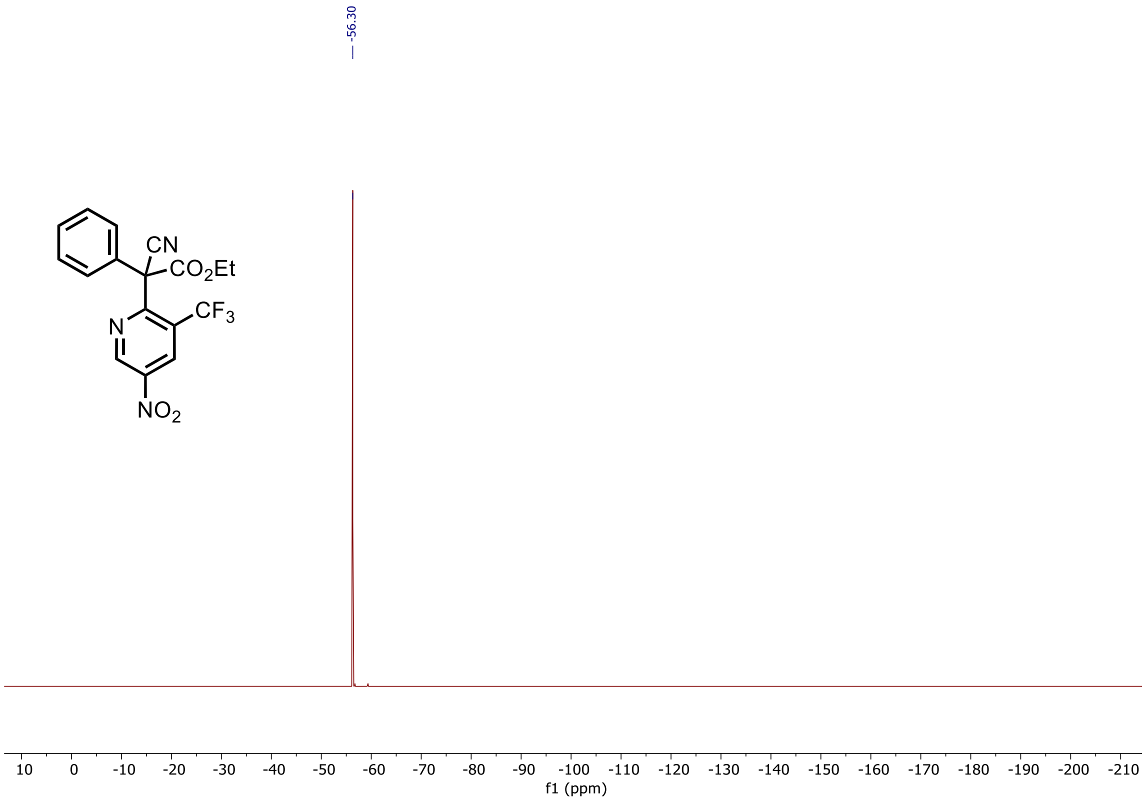

^{19}F NMR (471 MHz, $CDCl_3$) of **32**

Chiral HPLC chromatograms

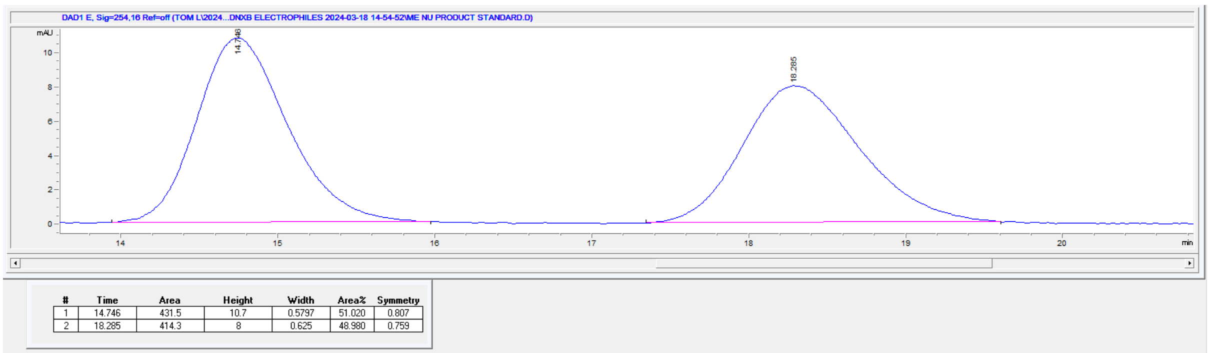

#	Time	Area	Height	Width	Area%	Symmetry
1	14.746	431.5	10.7	0.5797	51.020	0.807
2	18.285	414.3	8	0.625	48.980	0.759

Chiral HPLC chromatogram of (*rac*)-**3**

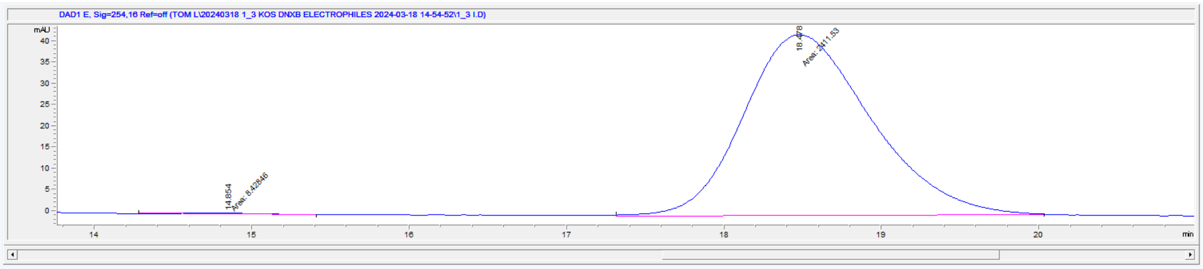

#	Time	Area	Height	Width	Area%	Symmetry
1	14.854	8.4	2.5E-1	0.5545	0.348	0.632
2	18.478	2411.5	42.6	0.9429	99.652	0.759

Chiral HPLC chromatogram of **(*R*)-3** from an analytical scale biotransformation

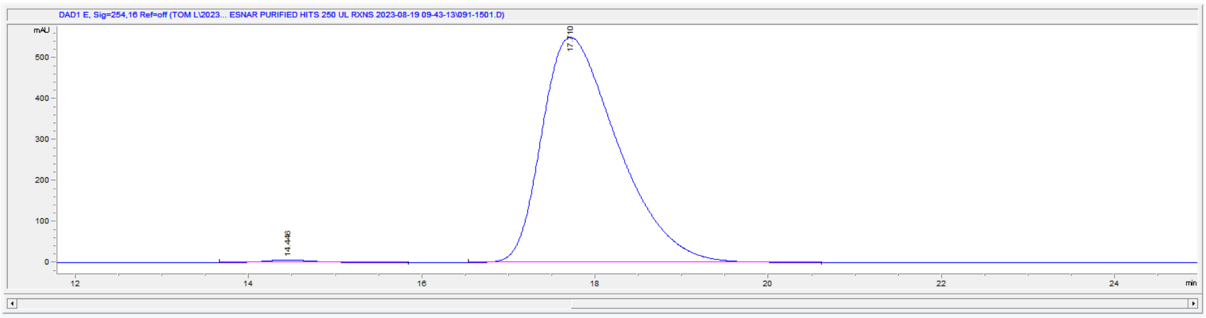

#	Time	Area	Height	Width	Area%	Symmetry
1	14.446	250.5	6.1	0.4976	0.742	0.825
2	17.71	33521.3	550.8	0.9381	99.258	0.563

Chiral HPLC chromatogram of **(*R*)-3** from a preparative scale biotransformation (following recrystallization) with S$_N$Ar1.3 using electrophile **2**

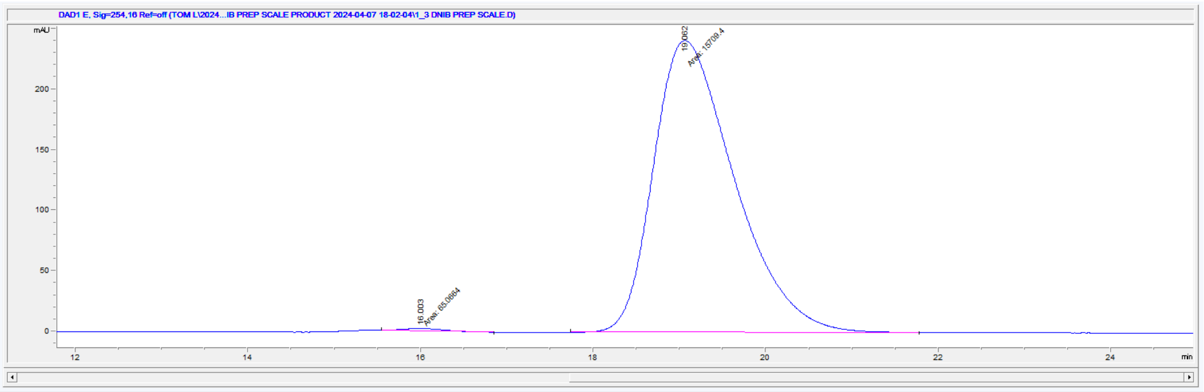

#	Time	Area	Height	Width	Area%	Symmetry
1	16.003	65.1	2	0.543	0.412	0.638
2	19.062	15709.4	241	1.0866	99.588	0.617

Chiral HPLC chromatogram of ***(R)*-3** from a preparative scale biotransformation with SNAr1.3 using electrophile **5**

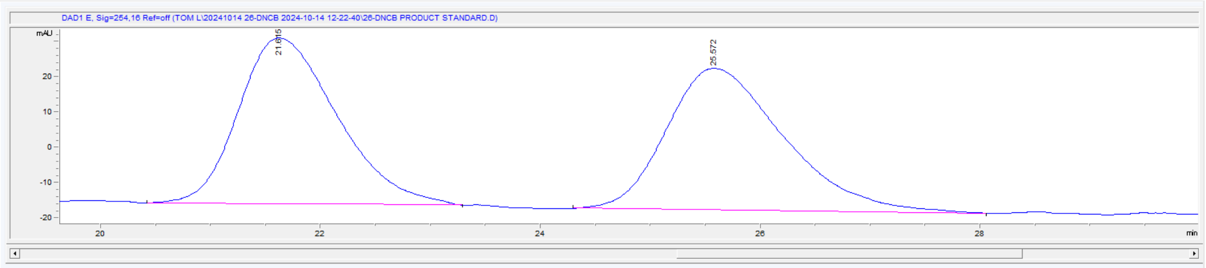

#	Time	Area	Height	Width	Area%	Symmetry
1	21.615	2945.3	47.1	0.743	49.562	0.668
2	25.572	2997.4	40.2	0.8742	50.438	0.65

Chiral HPLC chromatogram of (*rac*)-**7**

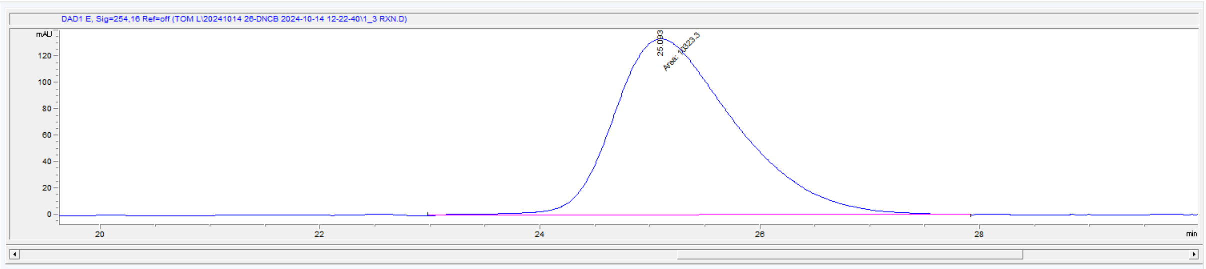

#	Time	Area	Height	Width	Area%	Symmetry
1	25.093	10323.3	133.8	1.286	100.000	0.617

Chiral HPLC chromatogram of **7** from a biotransformation

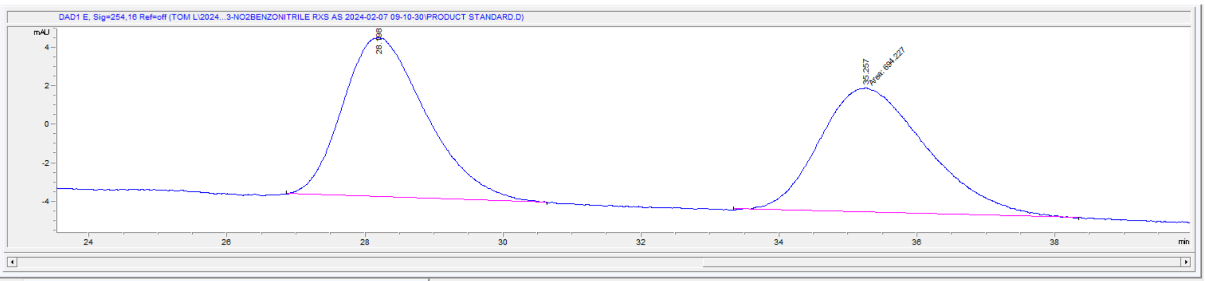

#	Time	Area	Height	Width	Area%	Symmetry
1	28.198	698	8.3	0.9918	50.136	0.74
2	35.257	694.2	6.5	1.7924	49.864	0.743

Chiral HPLC chromatogram of (*rac*)-**8**

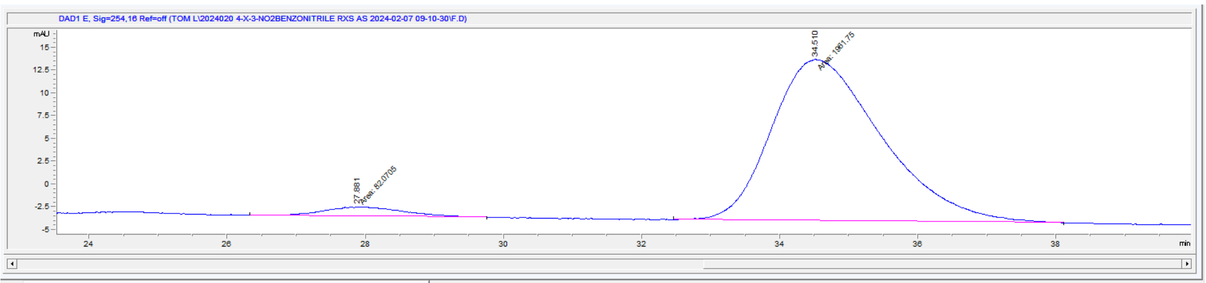

#	Time	Area	Height	Width	Area%	Symmetry
1	27.881	82.1	1	1.345	4.016	0.743
2	34.51	1961.7	17.7	1.847	95.984	0.64

Chiral HPLC chromatogram of **8** from a biotransformation

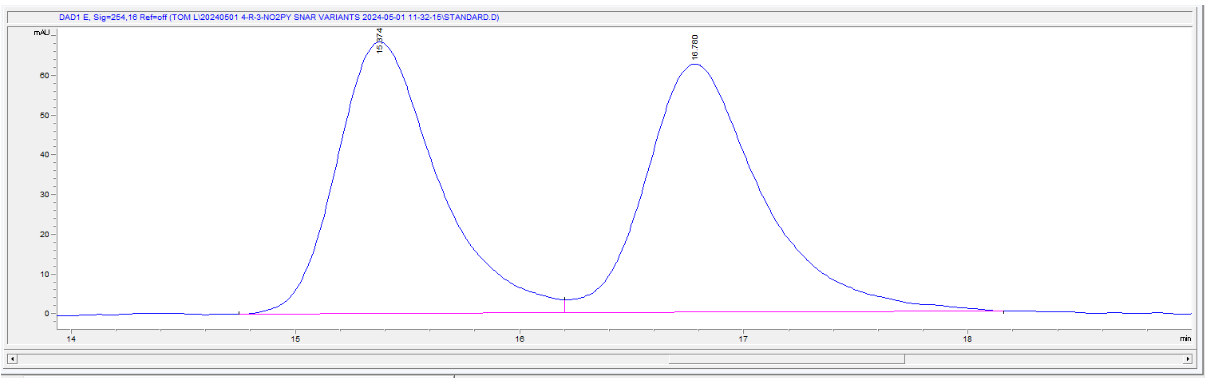

#	Time	Area	Height	Width	Area%	Symmetry
1	15.374	2154.8	68.4	0.4674	48.951	0.692
2	16.78	2247.2	62.5	0.5273	51.049	0.697

Chiral HPLC chromatogram of (*rac*)-**9**

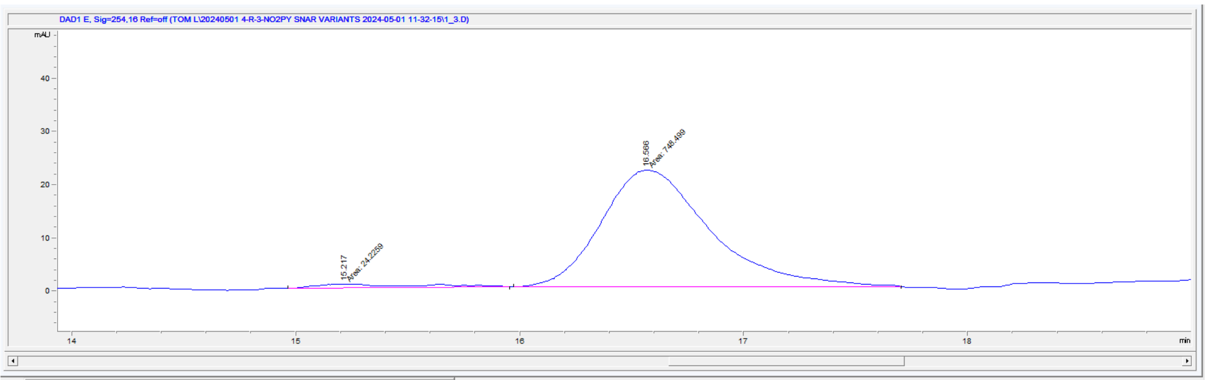

#	Time	Area	Height	Width	Area%	Symmetry
1	15.217	24.2	8E-1	0.5076	3.135	0.372
2	16.566	748.5	22	0.5672	96.865	0.696

Chiral HPLC chromatogram of **9** from a biotransformation

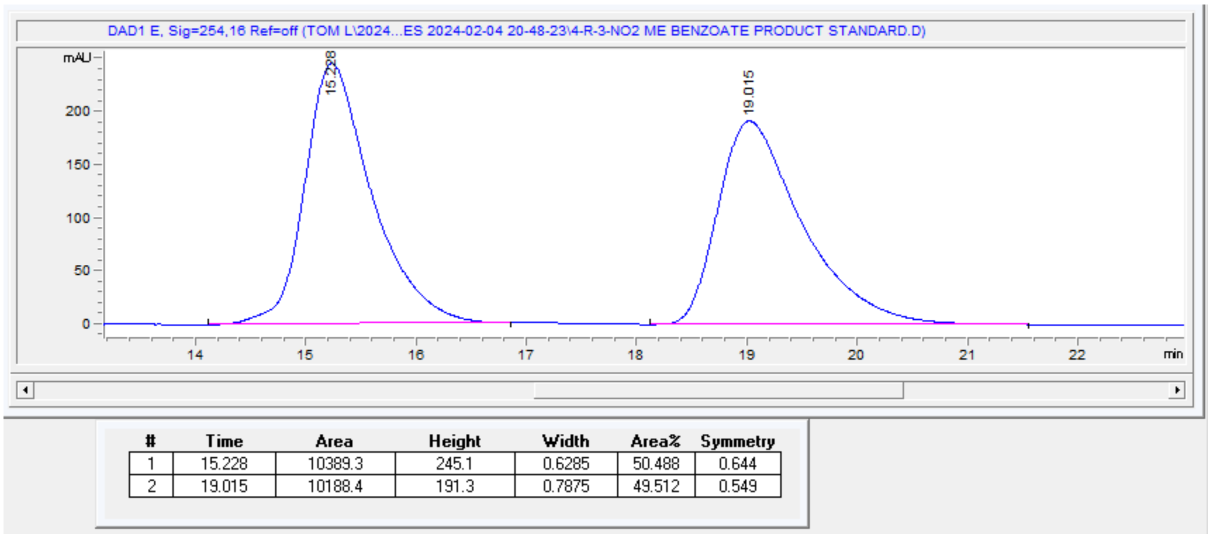

#	Time	Area	Height	Width	Area%	Symmetry
1	15.228	10389.3	245.1	0.6285	50.488	0.644
2	19.015	10188.4	191.3	0.7875	49.512	0.549

Chiral HPLC chromatogram of (*rac*)-**10**

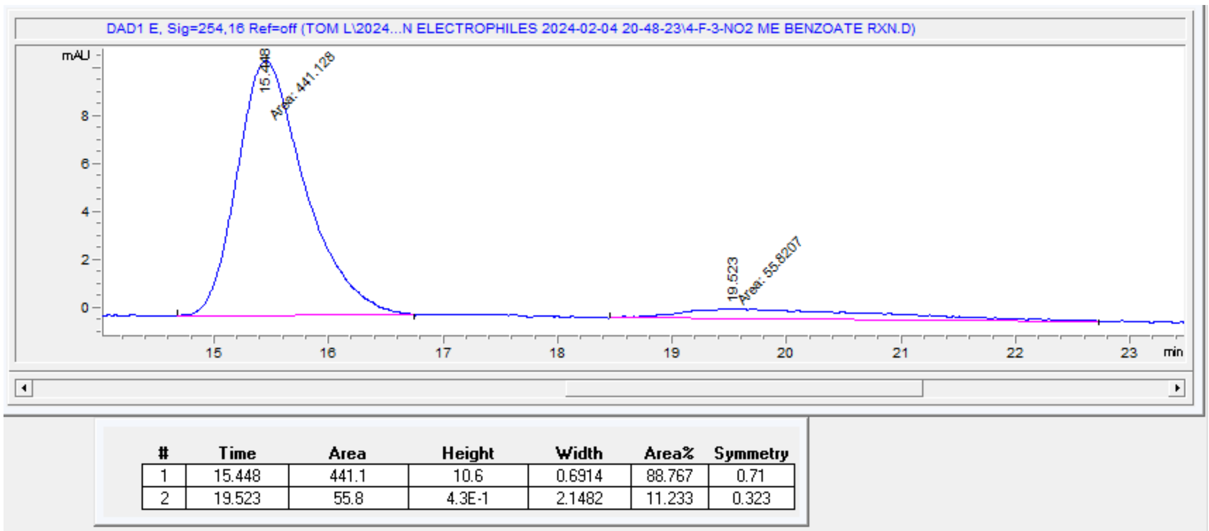

#	Time	Area	Height	Width	Area%	Symmetry
1	15.448	441.1	10.6	0.6914	88.767	0.71
2	19.523	55.8	4.3E-1	2.1482	11.233	0.323

Chiral HPLC chromatogram of **10** from a biotransformation

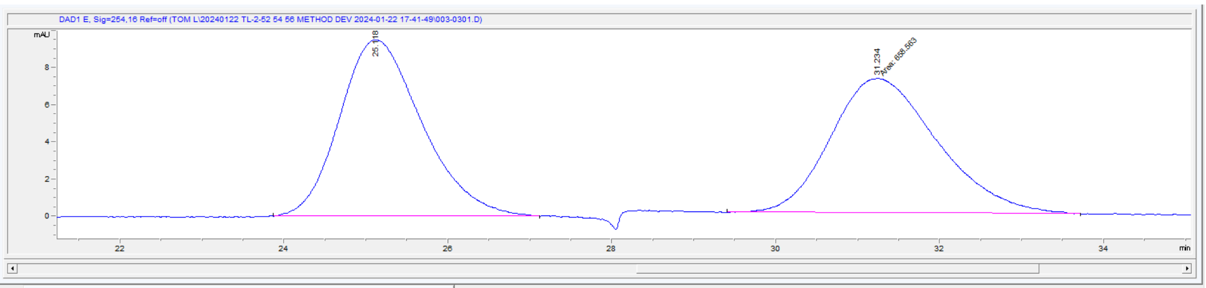

#	Time	Area	Height	Width	Area%	Symmetry
1	25.118	663	9.5	0.8278	50.168	0.774
2	31.234	658.6	7.2	1.5142	49.832	0.758

Chiral HPLC chromatogram of (*rac*)-**11**

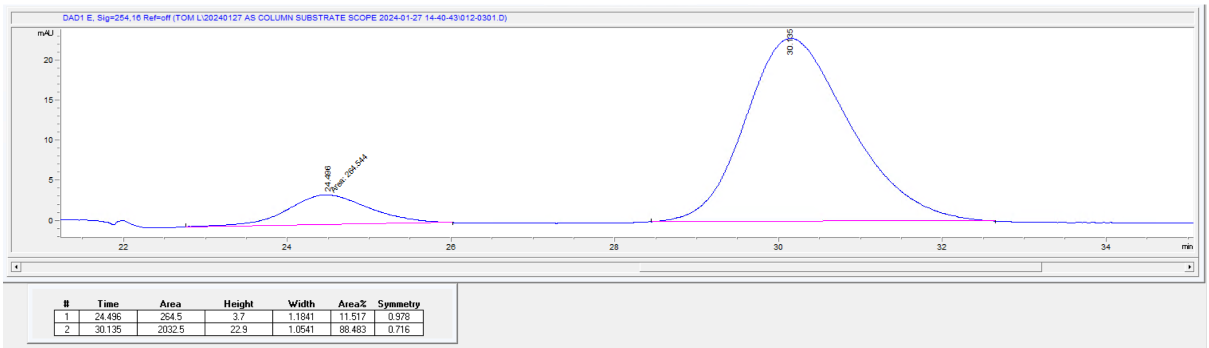

#	Time	Area	Height	Width	Area%	Symmetry
1	24.496	264.5	3.7	1.1841	11.517	0.978
2	30.135	2032.5	22.9	1.0541	88.483	0.716

Chiral HPLC chromatogram of **11** from a biotransformation

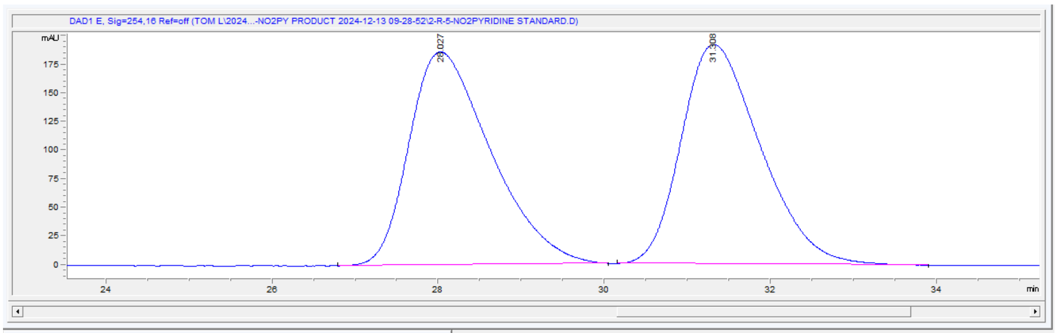

#	Time	Area	Height	Width	Area%	Symmetry
1	28.027	12723.7	186	0.9883	50.007	0.601
2	31.308	12719.9	191.6	0.9404	49.993	0.656

Chiral HPLC chromatogram of (*rac*)-**12**

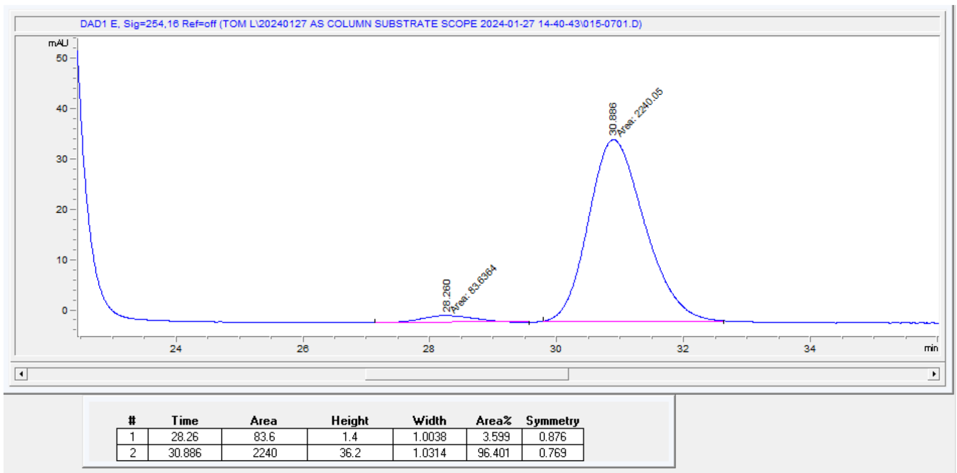

#	Time	Area	Height	Width	Area%	Symmetry
1	28.26	83.6	1.4	1.0038	3.599	0.876
2	30.886	2240	36.2	1.0314	96.401	0.769

Chiral HPLC chromatogram of **12** from a biotransformation

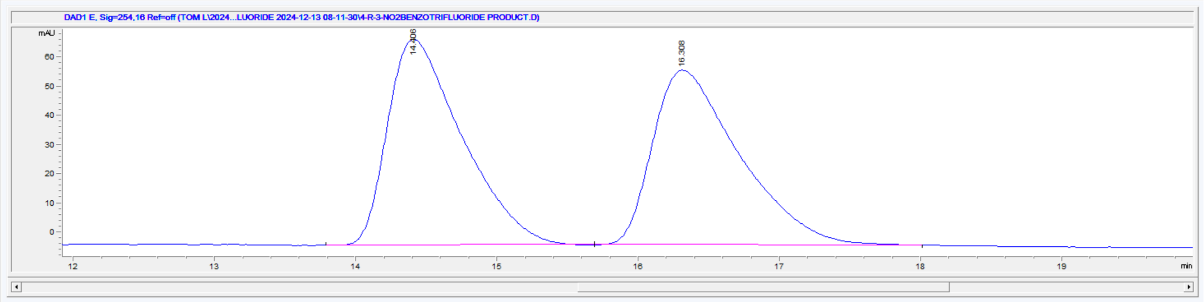

#	Time	Area	Height	Width	Area%	Symmetry
1	14.406	2587.4	70.6	0.5358	50.260	0.502
2	16.308	2560.6	59.9	0.6146	49.740	0.485

Chiral HPLC chromatogram of (*rac*)-**13**

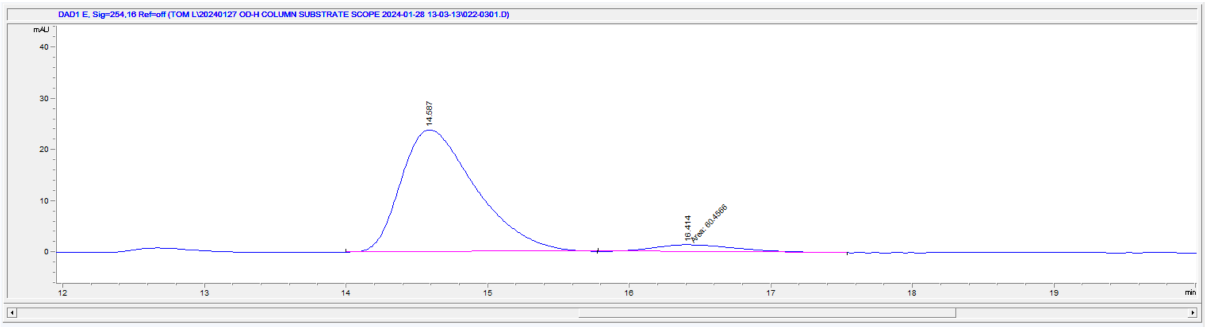

#	Time	Area	Height	Width	Area%	Symmetry
1	14.587	875.3	23.8	0.5584	93.539	0.576
2	16.414	60.5	1.4	0.7061	6.461	0.639

Chiral HPLC chromatogram of **13** from a biotransformation

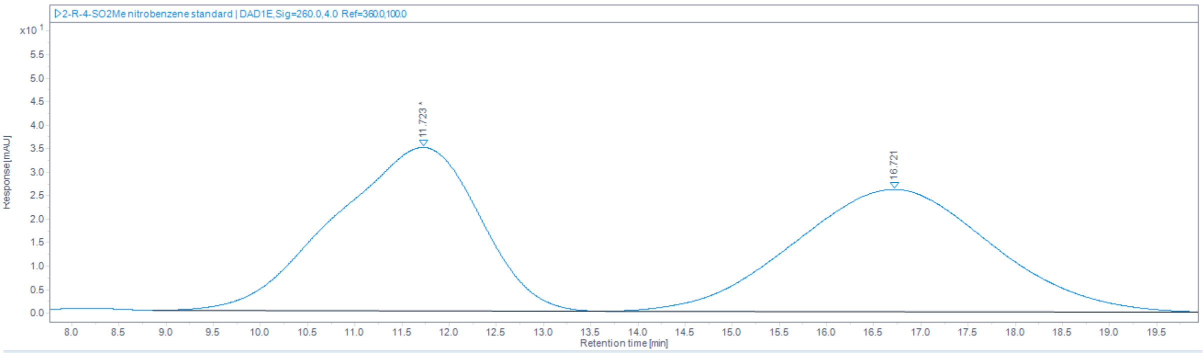

Injection Results

Peaks | Summary

#	Name	Signal description	RT (min)	Area (mAU·s)	Area%	Height (mAU)	Height%	Amount	Concentration	Start time (min)	End time (min)
1		DAD1E,Sig=260.0,4.0 Ref=360.0,100.0	11.723	3712.221	49.545	34.780	57.24			8.861	13.654
2		DAD1E,Sig=260.0,4.0 Ref=360.0,100.0	16.721	3780.353	50.455	25.983	42.76			13.654	19.994

Chiral HPLC chromatogram of (*rac*)-**14**

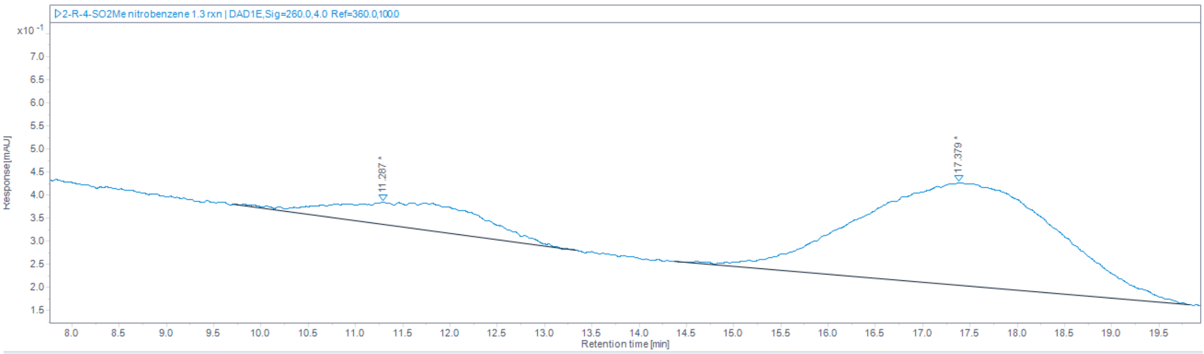

Injection Results

Peaks | Summary

#	Name	Signal description	RT (min)	Area (mAU·s)	Area%	Height (mAU)	Height%	Amount	Concentration	Start time (min)	End time (min)
1		DAD1E,Sig=260.0,4.0 Ref=360.0,100.0	11.287	6.037	15.660	0.048	17.68			9.705	13.333
2		DAD1E,Sig=260.0,4.0 Ref=360.0,100.0	17.379	32.515	84.340	0.222	82.32			14.372	19.822

Chiral HPLC chromatogram of **14** from a biotransformation

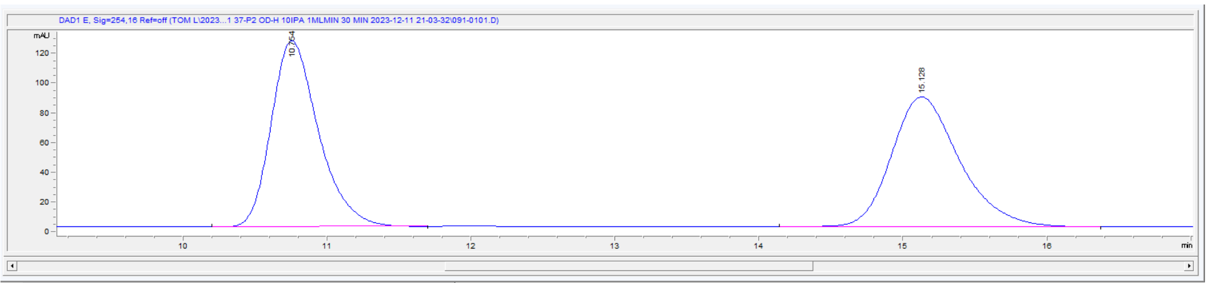

#	Time	Area	Height	Width	Area%	Symmetry
1	10.754	2857.4	125	0.3486	49.838	0.728
2	15.128	2875.9	87.3	0.4984	50.162	0.756

Chiral HPLC chromatogram of (*rac*)-**15**

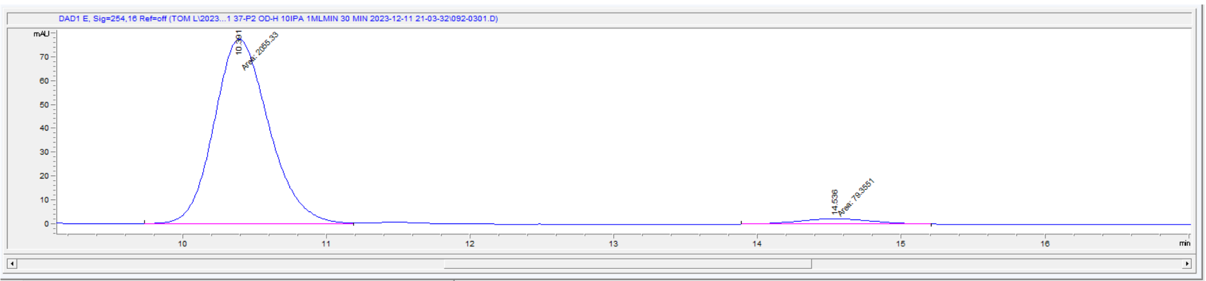

#	Time	Area	Height	Width	Area%	Symmetry
1	10.391	2055.3	77.5	0.4421	96.283	0.819
2	14.536	79.4	2.3	0.5812	3.717	0.978

Chiral HPLC chromatogram of **15** from a biotransformation

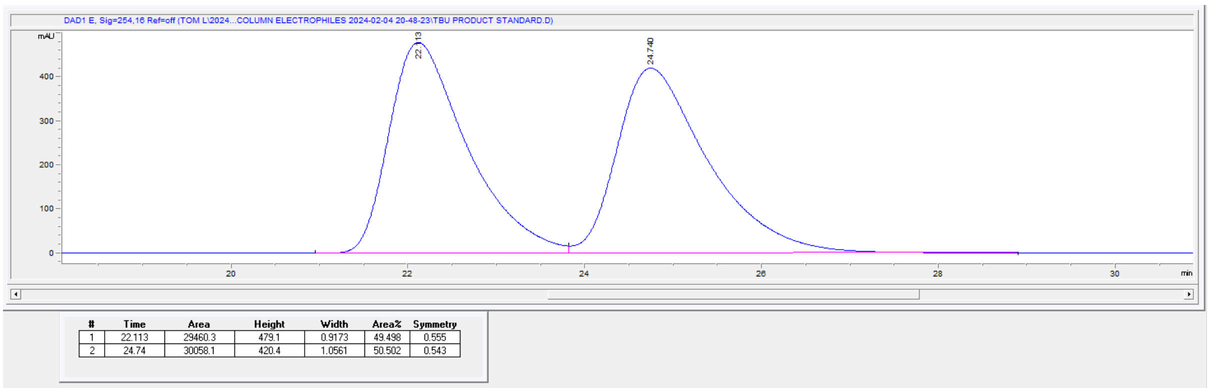

#	Time	Area	Height	Width	Area%	Symmetry
1	22.113	29460.3	479.1	0.9173	49.498	0.555
2	24.74	30058.1	420.4	1.0561	50.502	0.543

Chiral HPLC chromatogram of (*rac*)-**17**

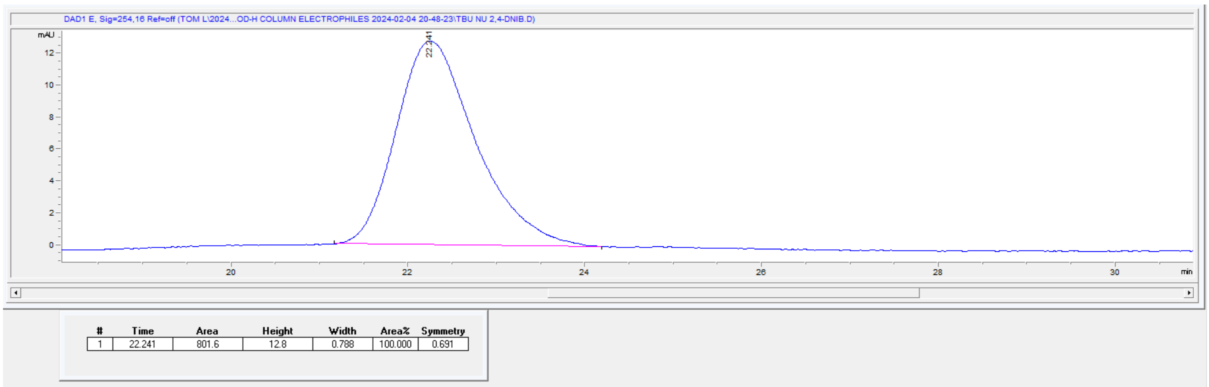

#	Time	Area	Height	Width	Area%	Symmetry
1	22.241	801.6	12.8	0.788	100.000	0.691

Chiral HPLC chromatogram of **17** from a biotransformation

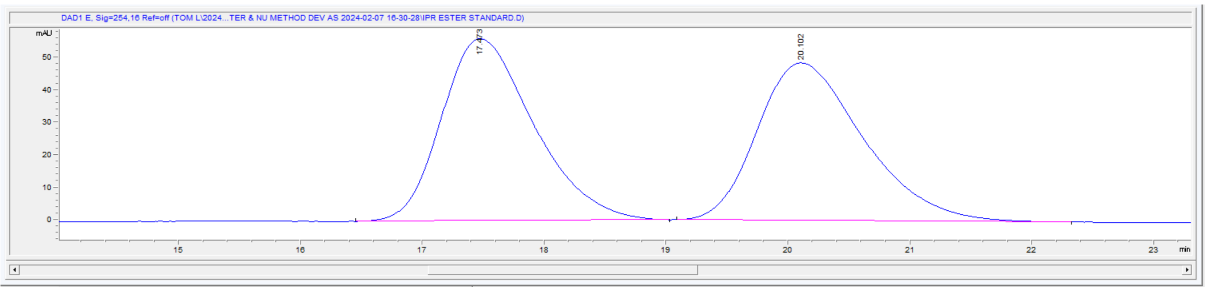

#	Time	Area	Height	Width	Area%	Symmetry
1	17.473	2945.9	56.1	0.7908	50.111	0.694
2	20.102	2932.9	48.5	0.8835	49.889	0.669

Chiral HPLC chromatogram of (*rac*)-**18**

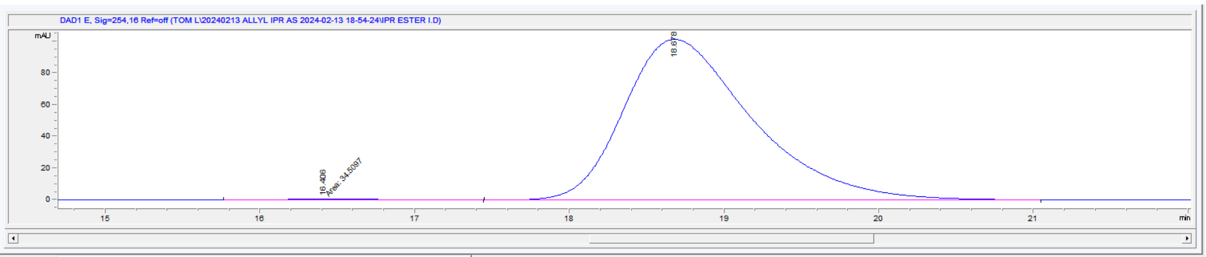

#	Time	Area	Height	Width	Area%	Symmetry
1	16.406	34.5	7.6E-1	0.7552	0.586	0.579
2	18.678	5857.7	101.4	0.856	99.414	0.623

Chiral HPLC chromatogram of **18** from a biotransformation

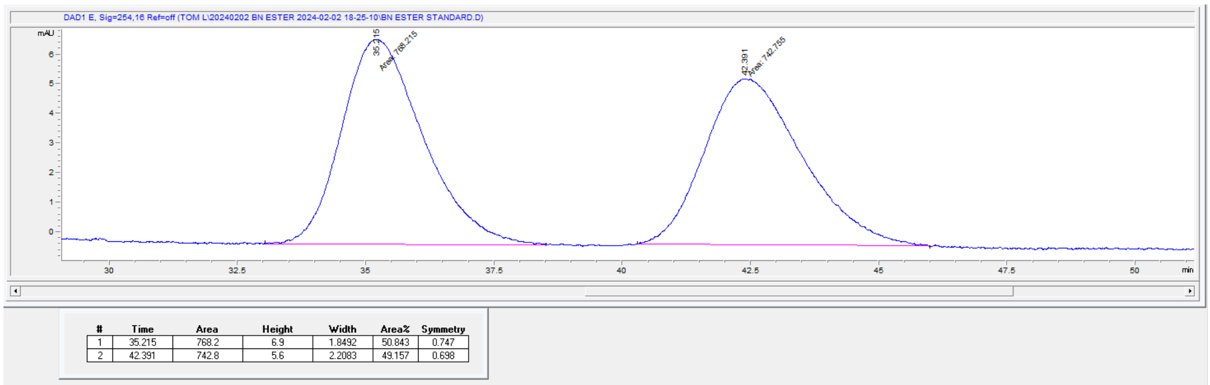

#	Time	Area	Height	Width	Area%	Symmetry
1	35.215	768.2	6.9	1.8492	50.843	0.747
2	42.391	742.8	5.6	2.2083	49.157	0.698

Chiral HPLC chromatogram of (*rac*)-**19**

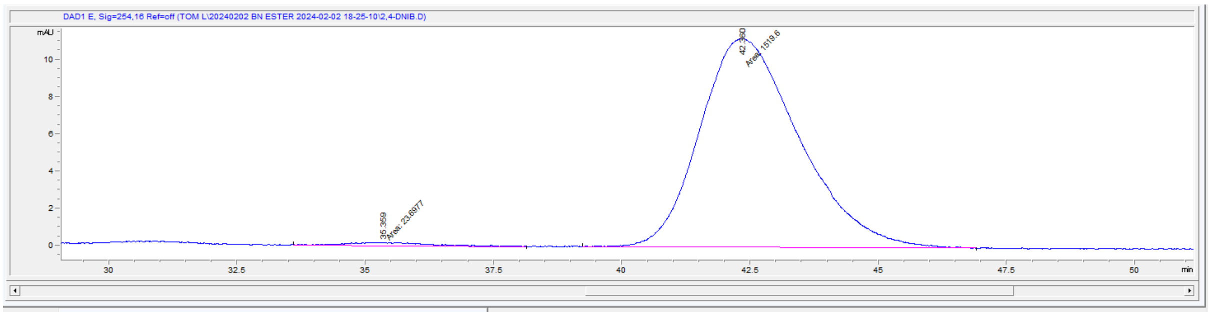

#	Time	Area	Height	Width	Area%	Symmetry
1	35.359	23.7	1.9E-1	2.0447	1.536	0.744
2	42.36	1519.6	11.2	2.2548	98.464	0.752

Chiral HPLC chromatogram of **19** from a biotransformation

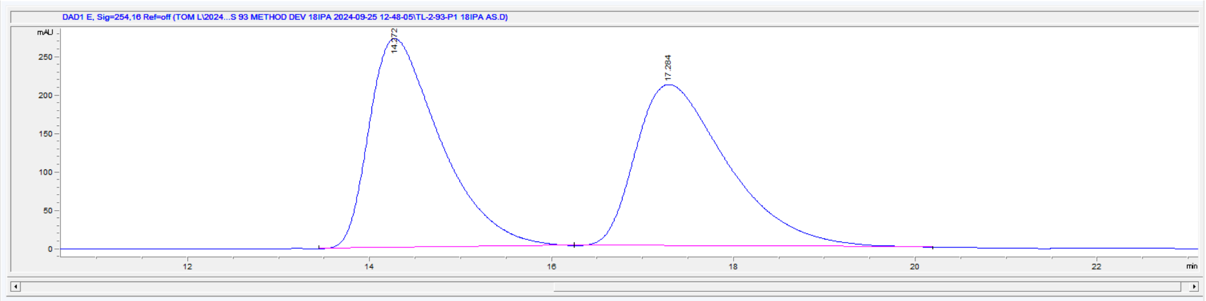

#	Time	Area	Height	Width	Area%	Symmetry
1	14.272	14846.6	272.4	0.8159	50.097	0.519
2	17.284	14789.1	210.2	1.0108	49.903	0.531

Chiral HPLC chromatogram of (*rac*)-**20**

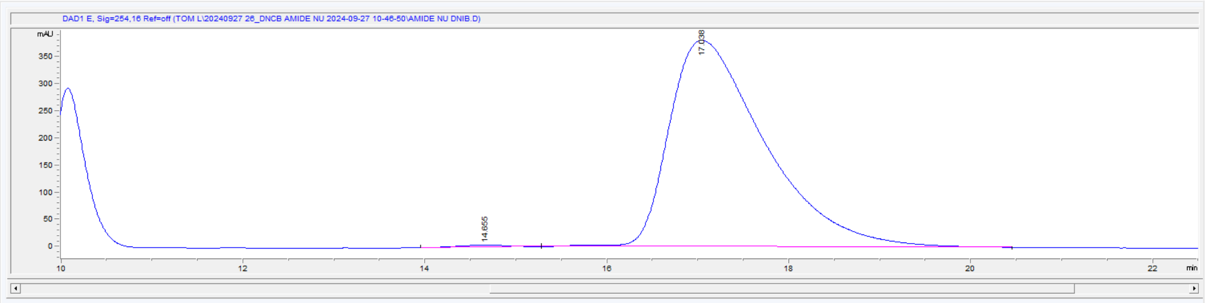

#	Time	Area	Height	Width	Area%	Symmetry
1	14.655	135.6	3.4	0.4759	0.485	1.169
2	17.038	27834	381.1	1.0601	99.515	0.486

Chiral HPLC chromatogram of **20** from a biotransformation

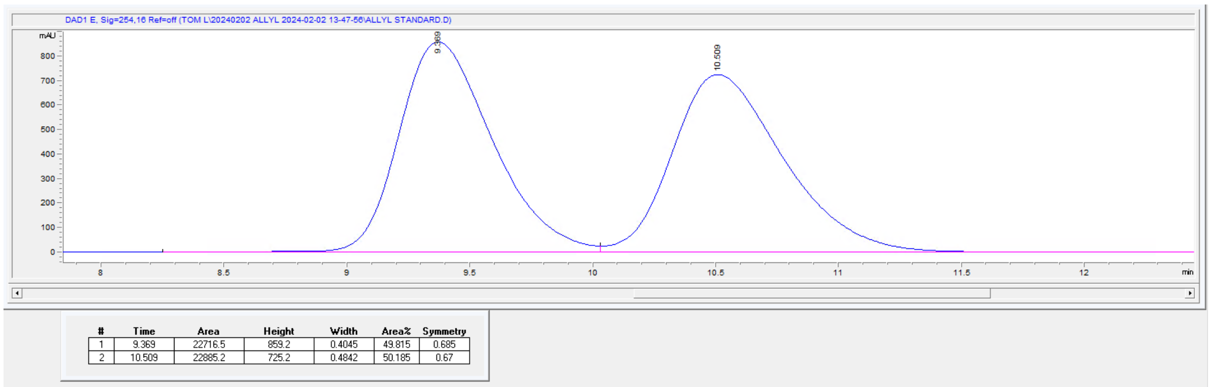

#	Time	Area	Height	Width	Area%	Symmetry
1	9.369	22716.5	859.2	0.4045	49.815	0.685
2	10.509	22885.2	725.2	0.4842	50.185	0.67

Chiral HPLC chromatogram of (*rac*)-**21**

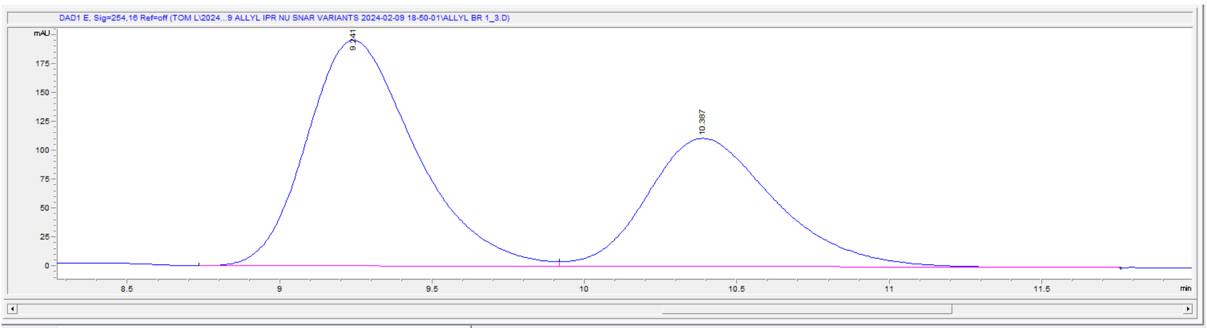

#	Time	Area	Height	Width	Area%	Symmetry
1	9.241	4867.9	195.5	0.3805	59.974	0.71
2	10.387	3248.8	111.1	0.443	40.026	0.73

Chiral HPLC chromatogram of **21** from a biotransformation

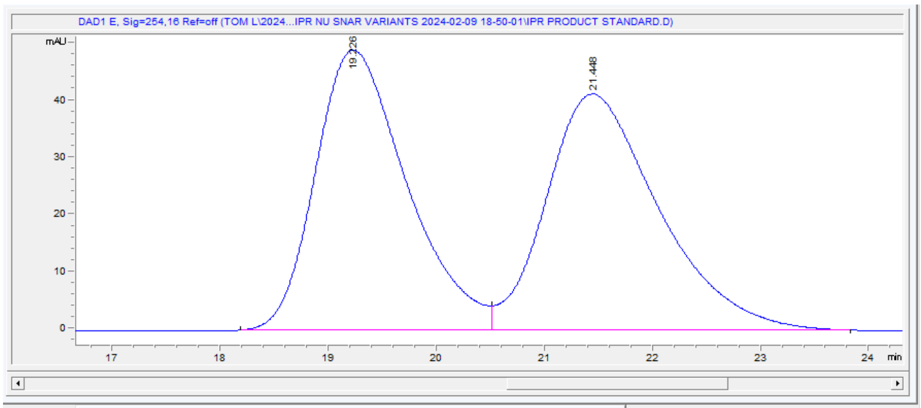

#	Time	Area	Height	Width	Area%	Symmetry
1	19.226	2888.3	49	0.8715	49.167	0.672
2	21.448	2986.2	41.3	0.9491	50.833	0.668

Chiral HPLC chromatogram of (*rac*)-**22**

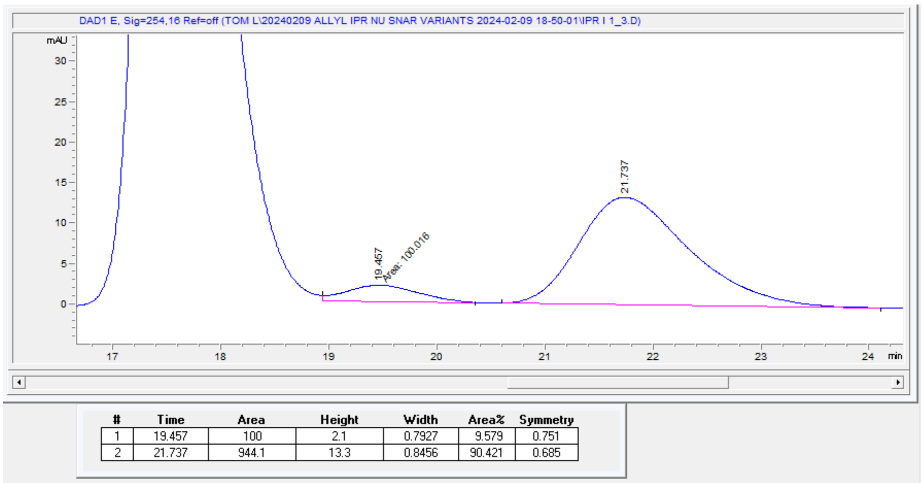

#	Time	Area	Height	Width	Area%	Symmetry
1	19.457	100	2.1	0.7927	9.579	0.751
2	21.737	944.1	13.3	0.8456	90.421	0.685

Chiral HPLC chromatogram of **22** from a biotransformation

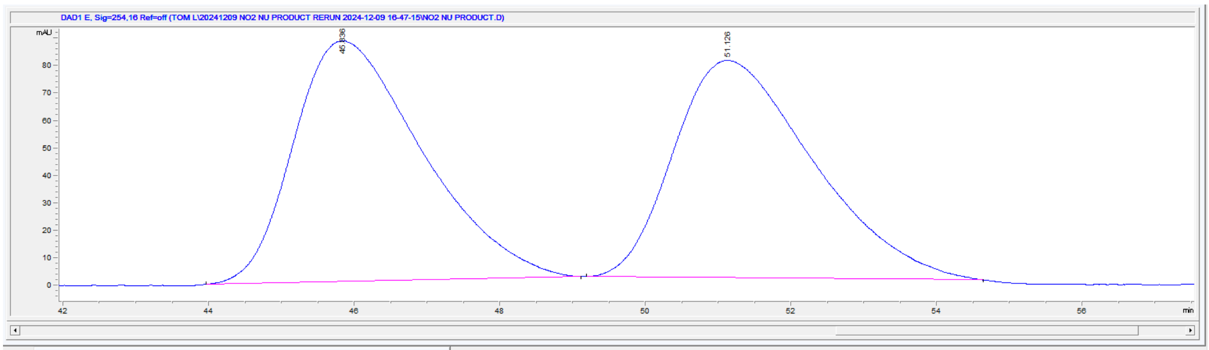

#	Time	Area	Height	Width	Area%	Symmetry
1	45.836	10774.1	87.9	1.4361	50.480	0.603
2	51.126	10569.2	79.4	1.563	49.520	0.616

Chiral HPLC chromatogram of (*rac*)-**23**

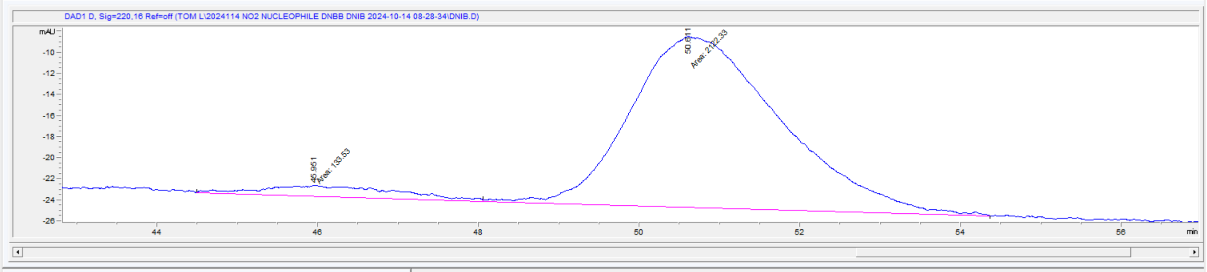

#	Time	Area	Height	Width	Area%	Symmetry
1	45.951	133.5	1.1	2.085	5.919	0.586
2	50.611	2122.3	16.3	2.1767	94.081	0.626

Chiral HPLC chromatogram of **23** from a biotransformation

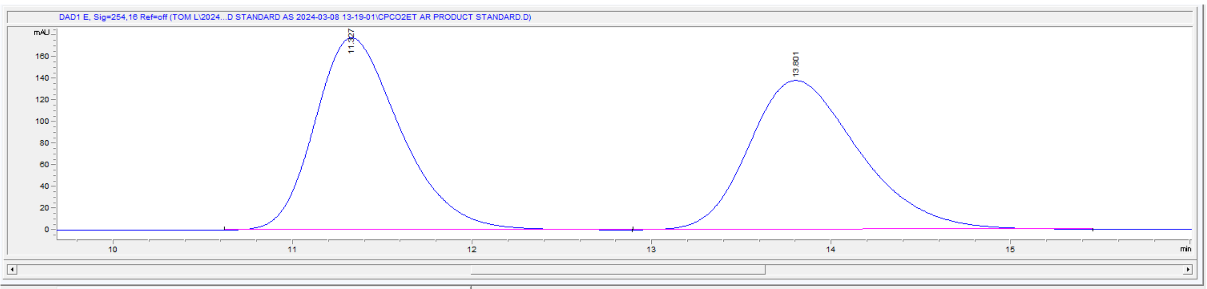

#	Time	Area	Height	Width	Area%	Symmetry
1	11.327	5899.1	177.6	0.5036	50.098	0.716
2	13.801	5875.9	137.6	0.656	49.902	0.705

Chiral HPLC chromatogram of (*rac*)-**24a**

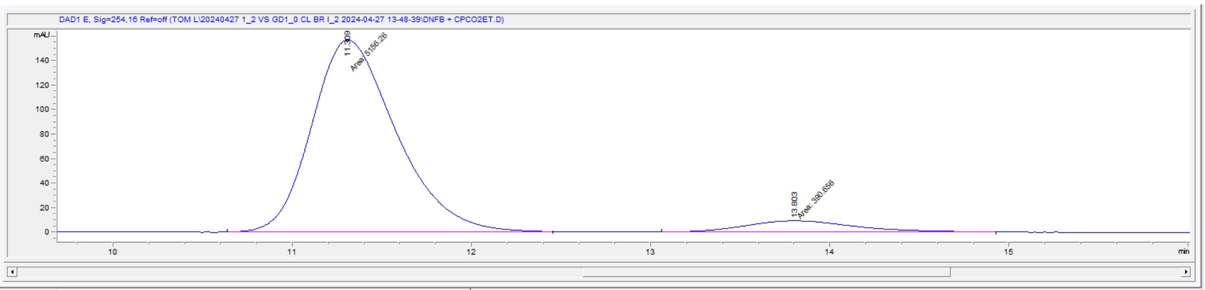

#	Time	Area	Height	Width	Area%	Symmetry
1	11.309	5156.3	156.7	0.5485	92.957	0.727
2	13.803	390.7	9.2	0.7104	7.043	0.822

Chiral HPLC chromatogram of **24a** from a biotransformation

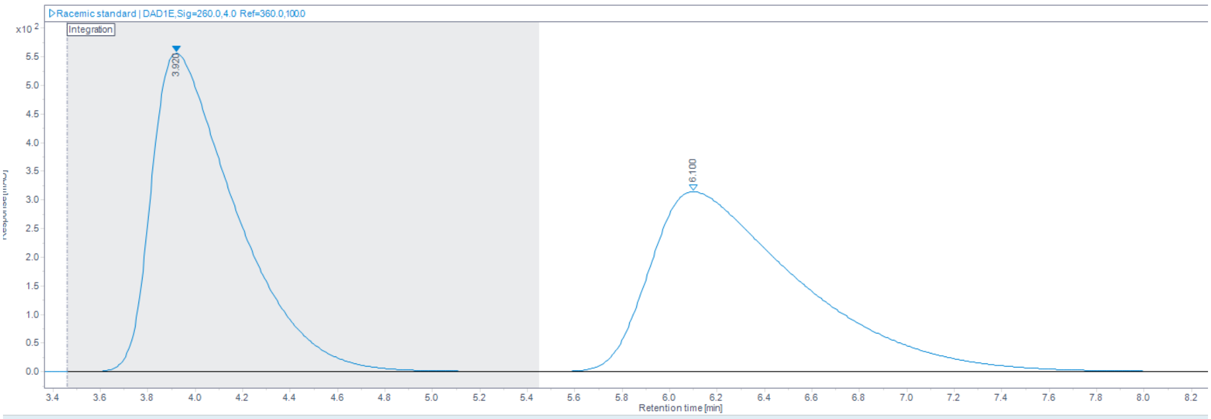

Injection Results

Peaks Summary

#	Name	Signal description	RT (min)	Area (mAU·s)	Area%	Height (mAU)	Height%	Amount	Concentration	Start time (min)	End time (min)
1		DAD1E,Sig=260.0,4.0 Ref=360.0,100.0	3.920	13764.782	49.980	556.220	63.92			3.465	5.450
2		DAD1E,Sig=260.0,4.0 Ref=360.0,100.0	6.100	13775.835	50.020	313.947	36.08			5.452	8.378

Chiral HPLC chromatogram of (*rac*)-**25**

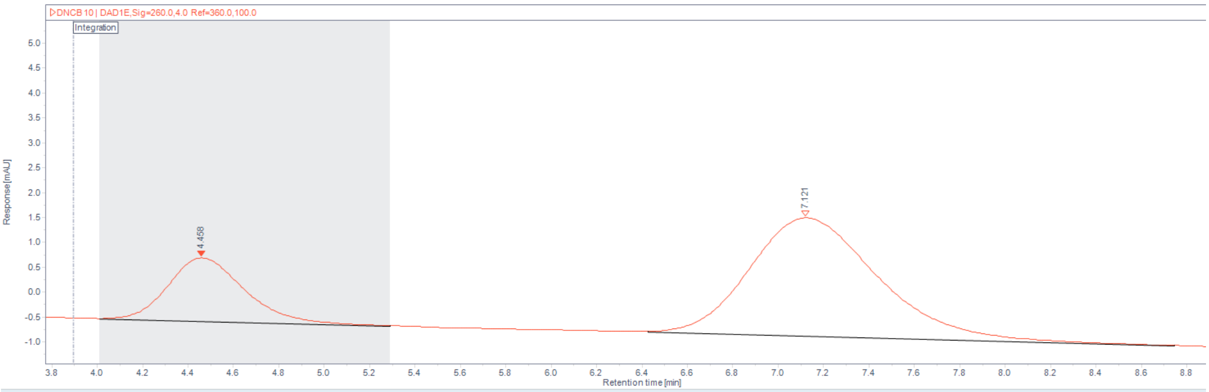

Injection Results

Peaks Summary

#	Name	Signal description	RT (min)	Area (mAU·s)	Area%	Height (mAU)	Height%	Amount	Concentration	Start time (min)	End time (min)
1		DAD1E,Sig=260.0,4.0 Ref=360.0,100.0	4.458	30.110	24.363	1.274	34.90			4.011	5.291
2		DAD1E,Sig=260.0,4.0 Ref=360.0,100.0	7.121	93.478	75.637	2.376	65.10			6.428	8.746

Chiral HPLC chromatogram of **25** from a biotransformation

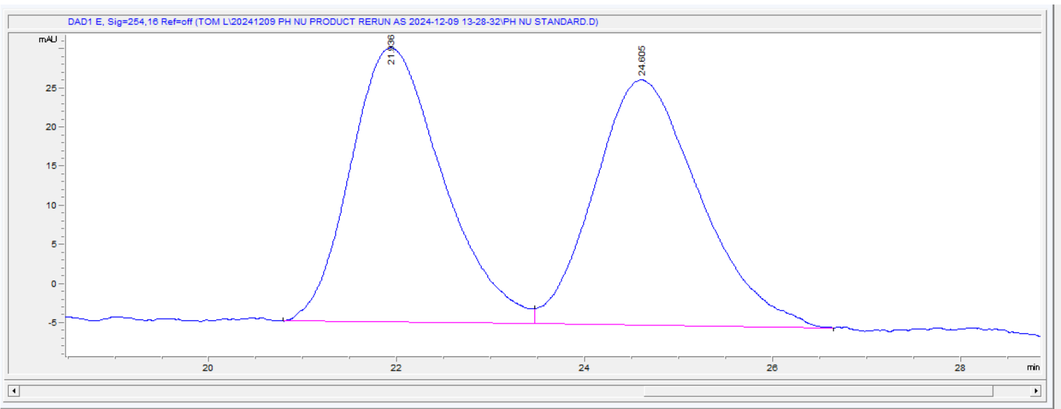

#	Time	Area	Height	Width	Area%	Symmetry
1	21.936	2414.2	35.1	0.8155	49.576	0.761
2	24.605	2455.6	31.5	0.9222	50.424	0.791

Chiral HPLC chromatogram of (*rac*)-**30**

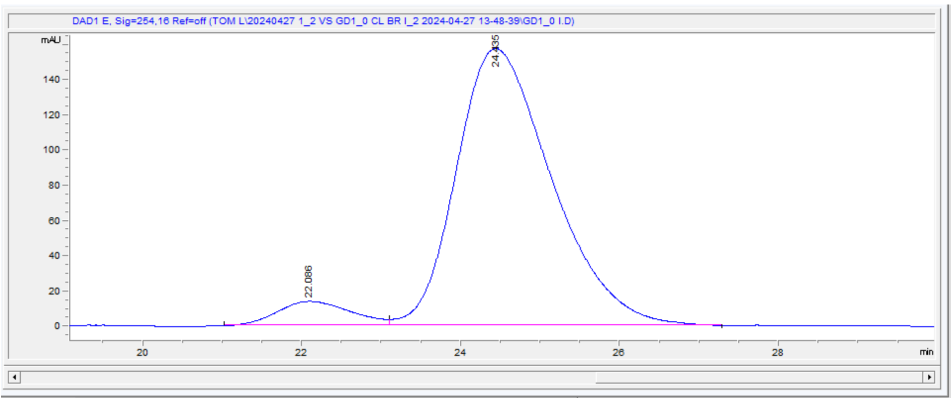

#	Time	Area	Height	Width	Area%	Symmetry
1	22.086	896.7	13.8	0.7651	6.415	0.737
2	24.435	13079.9	157.1	1.1571	93.585	0.667

Chiral HPLC chromatogram of **30** from a biotransformation

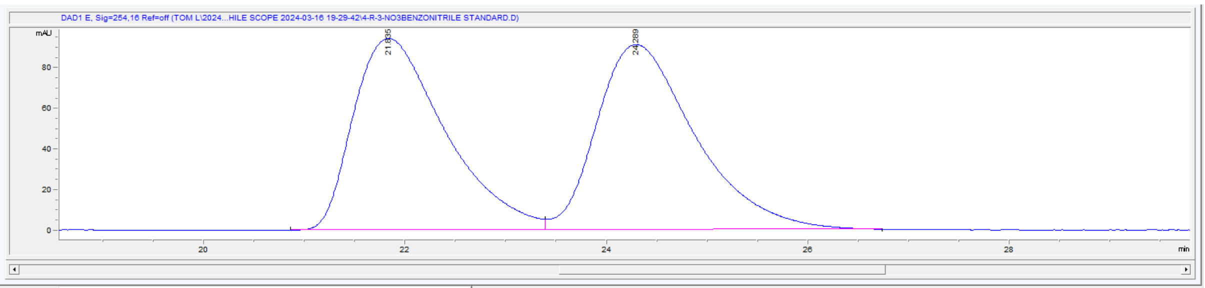

#	Time	Area	Height	Width	Area%	Symmetry
1	21.835	5981.5	94.4	0.9653	49.083	0.585
2	24.289	6204.9	91.2	1.0155	50.917	0.624

Chiral HPLC chromatogram of (*rac*)-**31**

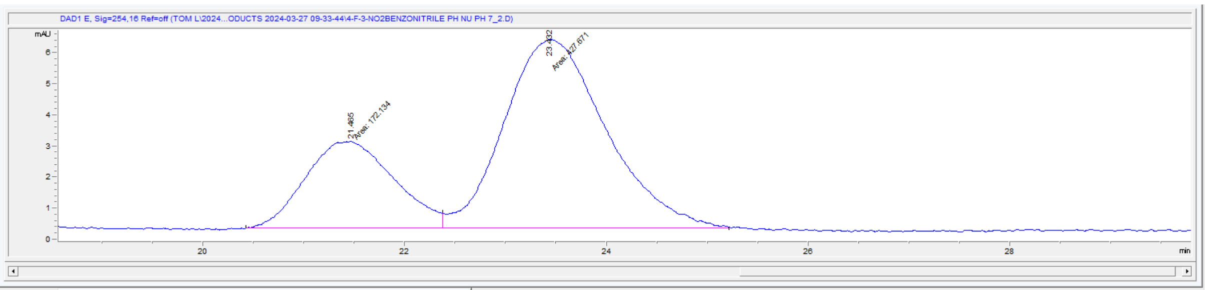

#	Time	Area	Height	Width	Area%	Symmetry
1	21.465	172.1	2.8	1.0242	28.698	1.007
2	23.432	427.7	6.1	1.1714	71.302	0.778

Chiral HPLC chromatogram of **31** from a biotransformation

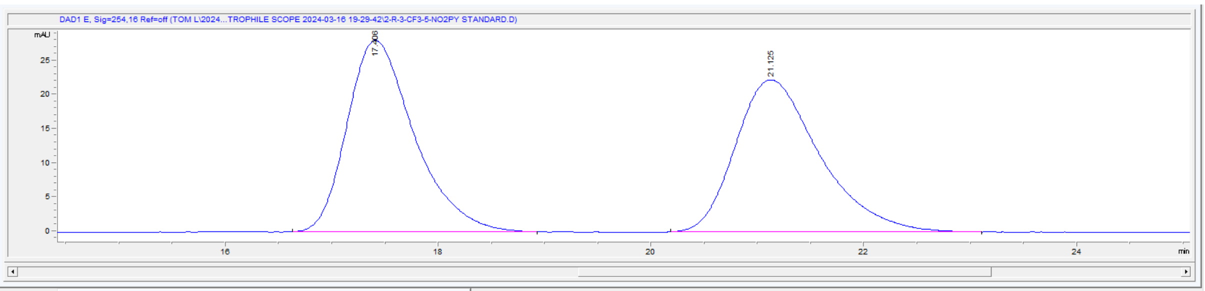

#	Time	Area	Height	Width	Area%	Symmetry
1	17.406	1226.1	28	0.6401	49.974	0.684
2	21.125	1227.4	22.3	0.763	50.026	0.688

Chiral HPLC chromatogram of (*rac*)-**32**

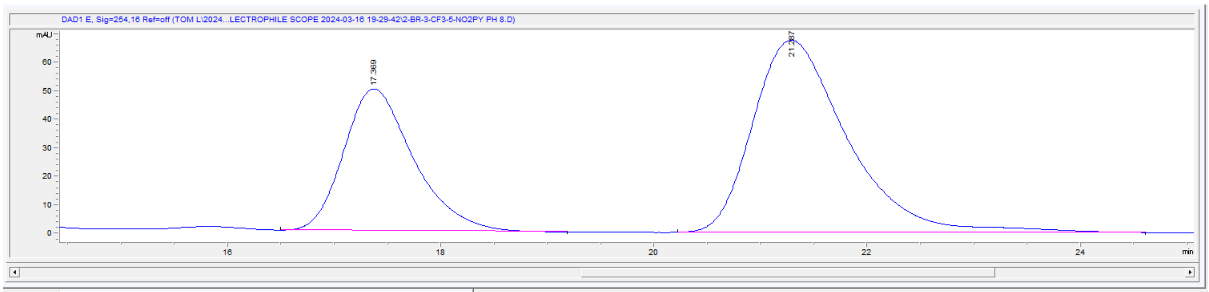

#	Time	Area	Height	Width	Area%	Symmetry
1	17.369	2272.4	49.7	0.6872	35.341	0.717
2	21.287	4157.6	67.3	0.9082	64.659	0.646

Chiral HPLC chromatogram of **32** from a biotransformation

References

1 Sheldrick, G. Crystal structure refinement with SHELXL. *Acta Cryst. C* **71**, 3-8 (2015). https://doi.org/doi:10.1107/S2053229614024218

2 Dolomanov, O. V., Bourhis, L. J., Gildea, R. J., Howard, J. A. K. & Puschmann, H. OLEX2: a complete structure solution, refinement and analysis program. *J. Appl. Crystallogr.* **42**, 339-341 (2009). https://doi.org/doi:10.1107/S0021889808042726

3 Yan, Q., Gin, E., Banwell, M. G., Willis, A. C. & Carr, P. D. A Unified Approach to the Isomeric α-, β-, γ-, and δ-Carbolines via their 6,7,8,9-Tetrahydro Counterparts. *J. Org. Chem.* **82**, 4328–4335 (2017).

4 Rao, M. N. *et al.* Asymmetric Base-Free Michael Addition at Room Temperature with Nickel-Based Bifunctional Amido-Functionalized N-Heterocyclic Carbene Catalysts. *Eur. J. Inorg. Chem.* **2015**, 1604–1615 (2015).

5 Reddy, M. D. & Watkins, E. B. Palladium-Catalyzed Direct Arylation of C(sp3)–H Bonds of α-Cyano Aliphatic Amides. *J. Org. Chem.* **80**, 11447–11459 (2015).

6 Aoyama, T., Nakano, T., Marumo, K., Uno, Y. & Shioiri, T. Reaction of Lithium Trimethylsilyldiazomethanide with Ketenimines Bearing Electron-Withdrawing Groups. *Synthesis* **1991**, 1163–1167 (1991).

7 Ohmura, T., Sasaki, I. & Suginome, M. Catalytic Generation of Rhodium Silylenoid for Alkene–Alkyne–Silylene [2 + 2 + 1] Cycloaddition. *Org. Lett.* **21**, 1649–1653 (2019).

8 Mallia, C. J., Englert, L., Walter, G. C. & Baxendale, I. R. Thiazole formation through a modified Gewald reaction. *Beilstein J. Org. Chem.* **11**, 875–883 (2015).

9 Bella, M., Kobbelgaard, S. & Jørgensen, K. A. Organocatalytic Regio- and Asymmetric C-Selective SNAr Reactions Stereoselective Synthesis of Optically Active Spiro-pyrrolidone-3,3′-oxoindoles. *J. Am. Chem. Soc.* **127**, 3670–3671 (2005).

10 Bandna, Guha, N. R., Shil, A. K., Sharma, D. & Das, P. Ligand-free solid supported palladium(0) nano/microparticles promoted C–O, C–S, and C–N cross coupling reaction. Tetrahedron Lett. 53, 5318–5322 (2012).
